# Consecutive β,β′‐Selective C(sp^3^)−H Silylation of Tertiary Amines with Dihydrosilanes Catalyzed by B(C_6_F_5_)_3_

**DOI:** 10.1002/anie.202016664

**Published:** 2021-03-03

**Authors:** Huaquan Fang, Kaixue Xie, Sebastian Kemper, Martin Oestreich

**Affiliations:** ^1^ Institut für Chemie Technische Universität Berlin Strasse des 17. Juni 115 10623 Berlin Germany

**Keywords:** amines, boron, C−H activation, Si−H activation, silicon

## Abstract

Tris(pentafluorophenyl)borane has been found to catalyze the two‐fold C(sp^3^)−H silylation of various trialkylamine derivatives with dihydrosilanes, furnishing the corresponding 4‐silapiperidines in decent yields. The multi‐step reaction cascade involves amine‐to‐enamine dehydrogenation at two alkyl residues and two electrophilic silylation reactions of those enamines, one inter‐ and one intramolecular.

## Graphical abstract

Acyclic tertiary amines with alkyl substitution undergo two consecutive C(sp^3^)−H silylation reactions with dihydrosilanes to form 4‐silapiperidines. Bond formation occurs β to the nitrogen atom at two of the alkyl residues. The reaction is catalyzed by the strong boron Lewis acid B(C_6_F_5_)_3_ and involves enamine intermediates generated by dehydrogenation.

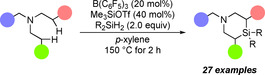

Selective functionalization of C(sp^3^)−H bonds is an important goal in synthetic chemistry.^[1]^ One way to achieve this is by transition‐metal‐catalyzed C(sp^3^)−H silylation,[^2^, ^3^] and recently selected boron Lewis acids also emerged as catalysts for this purpose.^[4]^ For example, B(C_6_F_5_)_3_ has been shown to abstract hydride from α‐C(sp^3^)−H bonds of amines to result in the formation of iminium ions and the borohydride;^[5]^ that iminium ion is C−H acidic and can be deprotonated by another molecule of the amine, affording the corresponding enamine along with the ammonium borohydride[^6^, ^7^] (Scheme 1, gray box). The net reaction is a dehydrogenation that enables subsequent bond formation with electrophiles in the β‐position to the nitrogen atom, thereby representing a formal activation of the β‐C(sp^3^)−H bond. This process has already been employed for silylation,^[8]^ alkylation,^[9]^ deuteration,^[10]^ and olefination^[11]^ of the β‐carbon atom of various (a)cyclic tertiary amines (Scheme 1, top). Of note, Park and Chang merged the C(sp^3^)−H silylation with a B(C_6_F_5_)_3_‐catalyzed intramolecular Friedel–Crafts‐type silylation^[12]^ for the synthesis of bridged silicon‐containing nitrogen heterocycles starting from N‐arylated piperidines.^[8a]^ However, the undirected silylation of acyclic tertiary amines^[3c]^ as well as their challenging two‐fold C(sp^3^)−H silylation are unprecedented. We disclose here a β,β′‐selective C(sp^3^)−H silylation of acyclic tertiary amines and dihydrosilanes catalyzed by B(C_6_F_5_)_3_ to directly arrive at sila analogues of piperidines (Scheme 1, bottom left). These are valuable building blocks in medicinal chemistry,^[13]^ for example, for the dopamine receptor antagonist sila‐haloperidol (Scheme 1, bottom right).^[14]^ Different from our approach, established syntheses typically start from divinyl‐substituted silanes employing a sequence of hydrobromination or hydroboration–oxidation–sulfonylation followed by dialkylation of a primary amine.^[15]^

**Scheme 1:**
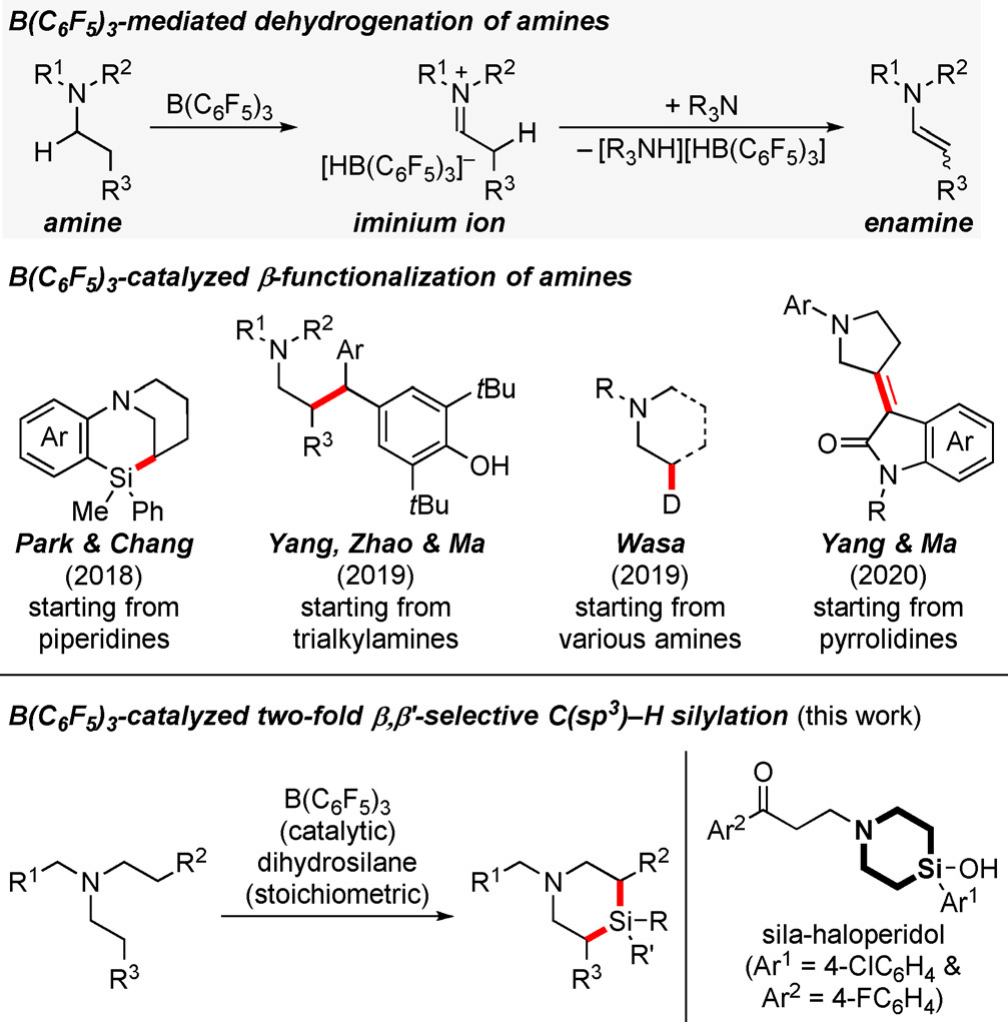
B(C_6_F_5_)_3_‐catalyzed β‐C(sp^3^)−H functionalization of tertiary amines. R groups=various aryl and alkyl groups as well as H; Ar=aryl group.

We began our investigation with optimizing the two‐fold C(sp^3^)−H silylation of benzyldiethylamine (**1 a**→**3 aa**; Table 1). Treatment of **1 a** and Ph_2_SiH_2_ (**2 a**, 2.0 equiv) with 20 mol % of B(C_6_F_5_)_3_ in *p*‐xylene at 150 °C afforded **3 aa** after 15 h in 56 % yield (Table 1, entry 1). Previous reports had indicated that the use of a metal oxide^[8a]^ or a silyl triflate^[5c]^ as an additive could improve the reactivity.^[16]^ However, substoichiometric amounts of CaO or SrO decreased the yield (Table 1, entries 2 and 3). The addition of 40 mol % of a silyl triflate improved the reactivity (Table 1, entries 4–6), and a 75 % yield of **3 aa** was obtained with Me_3_SiOTf as the additive. That yield was somewhat lower when using less and more Me_3_SiOTf, respectively (Table 1, entries 7 and 8). The reaction was completed within 2 h while a further shortened reaction time to 1 h resulted in a lower yield (Table 1, entries 9 and 10). Other arene solvents were tested but none provided a better outcome (Table 1, entries 11–13). A control experiment verified that Me_3_SiOTf is unable to mediate the reaction in the absence of B(C_6_F_5_)_3_ (Table 1, entry 14). Less B(C_6_F_5_)_3_ or Ph_2_SiH_2_ (**2 a**) as well as lowering the temperature to 120 °C led to a decreased reactivity (Table 1, entries 15–17). The volume of the reaction vessel was also examined, and the results indicate that vessels smaller than 10 mL are detrimental (Table 1, entries 18 and 19). We ascribe this to catalyst inhibition by dihydrogen at high pressure.^[7]^ A good yield was restored on a 5.0 mmol scale when performing the two‐fold C(sp^3^)−H silylation in an open system with a continuous flow of nitrogen gas (Table 1, entry 20).

**Table 1:** Selected examples of the optimization of B(C_6_F_5_)_3_‐catalyzed two‐fold C(sp^3^)−H silylation.^[a]^ 
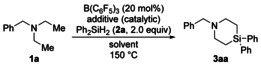

Entry	Additive (mol %)	Solvent	*t* [h]	Yield [%]^[b]^
1	–	*p*‐xylene	15	56
2	CaO (50)	*p*‐xylene	15	48
3	SrO (50)	*p*‐xylene	15	50
4	Me_3_SiOTf (40)	*p*‐xylene	15	75
5	*t*BuMe_2_SiOTf (40)	*p*‐xylene	15	66
6	*i*Pr_3_SiOTf (40)	*p*‐xylene	15	62
7	Me_3_SiOTf (20)	*p*‐xylene	15	67
8	Me_3_SiOTf (80)	*p*‐xylene	15	60
9	Me_3_SiOTf (40)	*p*‐xylene	2	75 (73)
10	Me_3_SiOTf (40)	*p*‐xylene	1	42
11	Me_3_SiOTf (40)	toluene	2	74
12	Me_3_SiOTf (40)	benzene	2	62
13	Me_3_SiOTf (40)	C_6_H_5_Cl	2	55
14^[c]^	Me_3_SiOTf (40)	*p*‐xylene	2	0
15^[d]^	Me_3_SiOTf (40)	*p*‐xylene	2	49
16^[e]^	Me_3_SiOTf (40)	*p*‐xylene	2	68
17^[f]^	Me_3_SiOTf (40)	*p*‐xylene	15	61
18^[g]^	Me_3_SiOTf (40)	*p*‐xylene	2	60
19^[h]^	Me_3_SiOTf (40)	*p*‐xylene	2	34
20^[i,j]^	Me_3_SiOTf (40)	*p*‐xylene	12	(65)

[a] All reactions were performed on a 0.050 mmol scale in a 10 mL sealed tube. [b] Yields determined by ^1^H NMR spectroscopy using mesitylene as an internal standard; isolated yields in parentheses. [c] Without B(C_6_F_5_)_3_. [d] 10 mol % B(C_6_F_5_)_3_ used. [e] 1.5 equiv Ph_2_SiH_2_ (**2 a**) used. [f] Run at 120 °C. [g] 5.0 mL sealed tube used. [h] 1.0 mL sealed tube used. [i] Open system with a continuous flow of nitrogen gas. [j] 5.0 mmol scale.

We continued exploring the scope under the optimized reaction setup (Scheme 2; cf. Table 1, entry 9). It must be noted that reductive C(sp^3^)−N bond cleavage^[17]^ is competing in any of the reactions summarized in Scheme 2, and secondary amines are the major byproducts (not quantified because of their volatility). N‐Benzylated diethylamine derivatives bearing various electron‐donating or ‐withdrawing substituents on the aryl moiety reacted with Ph_2_SiH_2_ (**2 a**) to furnish the corresponding 4‐silapiperidines in moderate to good yields (**1 b**–**l**→**3 ba**–**la**; gray box). All halo groups (**1 g**–**j**) and a trifluoromethyl group (**1 k**) were compatible. Tertiary amine **1 l** containing a methyl ether underwent demethylation/silylation, and the free phenol was isolated in 50 % yield after purification by flash chromatography on silica gel (**1 l**→**3 la**). A lower yield was obtained for a naphth‐2‐ylmethyl instead of the benzyl group (**1 m**→**3 ma**). The bis(4‐silapiperidine) **3 na** was formed in 47 % yield by four‐fold C(sp^3^)−H silylation of **1 n**. Replacing the benzyl group by an alkyl group was feasible (**1 o**‐**q**→**3 oa**‐**qa**). Notably, the two‐fold C(sp^3^)−H silylation of substrate **1 o** bearing two ethyl groups and one cyclohexyl group proceeded chemoselectively at the ethyl groups to form **3 oa**. Substituted 4‐silapiperidine derivatives were obtained from tertiary amines with groups other than ethyl (**1 r**–**t**→**3 ra**–**ta**). As expected, **1 t** gave **3 ta** with essentially no diastereoselectivity (*cis*/*trans*=58:42). Attempted but failed cyclizations included tertiary benzylamines as precursors having two isopropyl, cyclohexyl, isobutyl, or phenethyl groups as well as 1‐benzylazepane (see the Supporting Information for details).

**Scheme 2:**
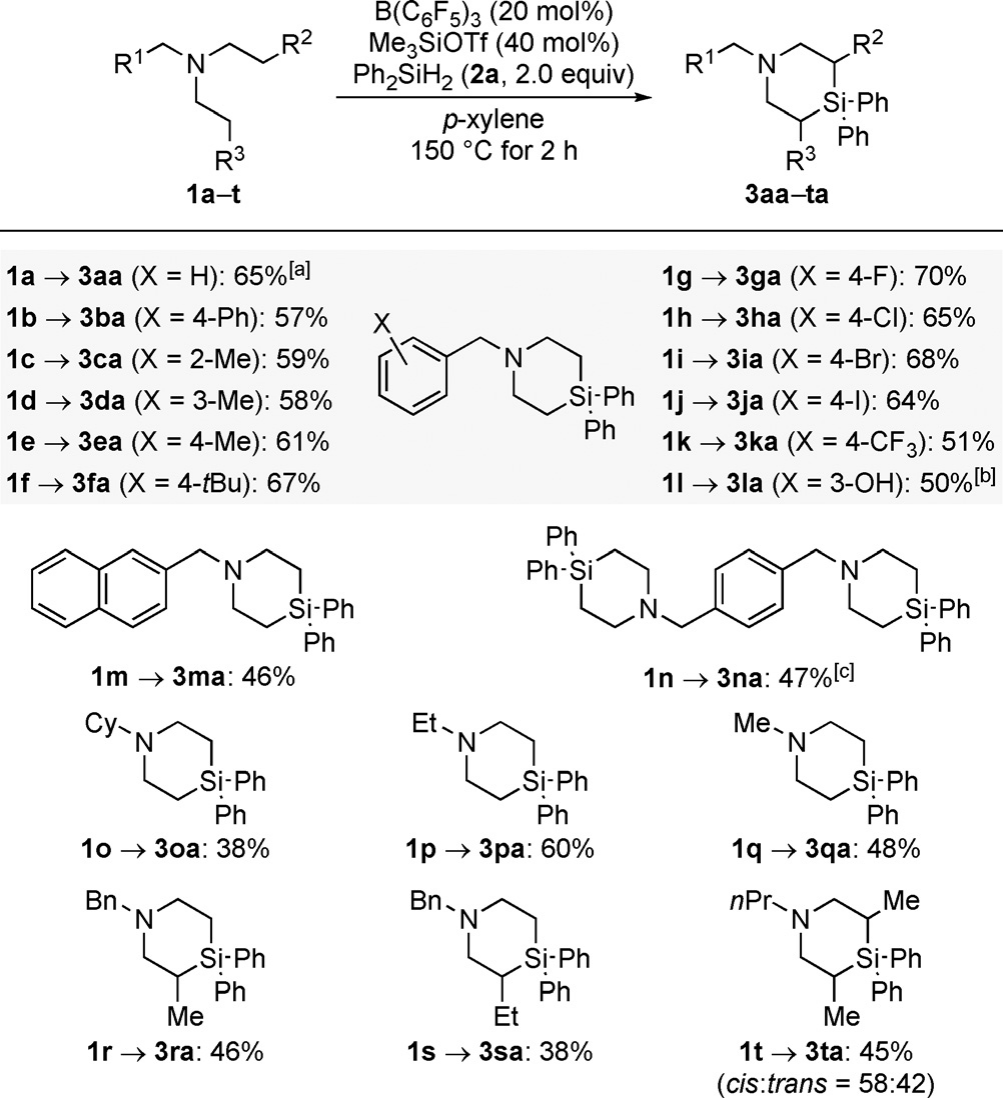
Scope I: Variation of the tertiary amine. Reaction conditions (0.10 mmol scale): B(C_6_F_5_)_3_ (20 mol %), Me_3_SiOTf (40 mol %), Ph_2_SiH_2_ (**2 a**, 2.0 equiv), and *p*‐xylene (0.80 mL) at 150 °C for 2 h. Yields are isolated yields. [a] See Table 1, entry 20. [b] Starting from *N*‐ethyl‐*N*‐(3‐methoxybenzyl)ethanamine (**1 l**). [c] 40 mol % of B(C_6_F_5_)_3_, 80 mol % of Me_3_SiOTf, and 4.0 equiv of Ph_2_SiH_2_ (**2 a**) used. Bn=benzyl, Cy=cyclohexyl.

We also tried the silylation of the tertiary aniline derivative **1 u** which did not react under Park's and Chang's catalytic system (Scheme 3).^[8a]^ Bicyclic **4 ua** and tricyclic **5 ua** formed in yields of 50 % and 8 %, respectively. The proportion of **5 ua** increased at longer reactions times, for example, 44 % yield of **4 ua** and 25 % yield of **5 ua** after 3 h. As for the aforementioned method,^[8a]^ intramolecular Friedel–Crafts C(sp^2^)−H silylation^[12]^ is favored over intramolecular C(sp^3^)−H silylation.

**Scheme 3:**
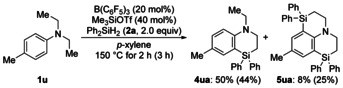
Consecutive C(sp^3^)−H/C(sp^2^)−H silylation of an aniline derivative.

We next assessed the dihydrosilane scope in the reaction of model substrate **1 a** (Table 2). Diarylsilanes **2 b**–**e** exhibited good reactivity, furnishing the corresponding products in the same yield range as compared to **2 a** (**1 a**→**3 ab**–**ae**; Table 2, entries 1–4). No reaction was seen with sterically hindered dimesitylsilane (**2 f**; Table 2, entry 5). Modest yield was obtained with MePhSiH_2_ (**2 g** in **1 a**→**3 ag**; Table 2, entry 6) but the synthesis of a spirocyclic derivative with 1‐silaindane (**2 h**) was low yielding (Table 2, entry 7).^[18]^ The dialkylsilane Et_2_SiH_2_ (**2 i**) afforded desired **3 ai** in moderate yield (Table 2, entry 8), but again, there was no reaction with bulky *t*Bu_2_SiH_2_ (**2 j**; Table 2, entry 9). The reaction of the primary hydrosilane PhSiH_3_ yielded only trace amounts of the 4‐silapiperidine (not shown).

**Table 2:** Scope II: Variation of the hydrosilane.^[a]^ 
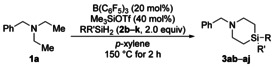

Entry	Hydrosilane	R	R′	Yield [%]^[b]^
1	**2 b**	4‐MeC_6_H_4_	4‐MeC_6_H_4_	65 (**3 ab**)
2	**2 c**	4‐*t*BuC_6_H_4_	4‐*t*BuC_6_H_4_	65 (**3 ac**)
3	**2 d**	4‐FC_6_H_4_	4‐FC_6_H_4_	67 (**3 ad**)
4	**2 e**	Ph	Naphth‐1‐yl	68 (**3 ae**)
5	**2 f**	Mes	Mes	no reaction (**3 af**)
6	**2 g**	Ph	Me	40 (**3 ag**)
7	**2 h**	1‐silaindan‐1,1‐diyl		traces (**3 ah**)
8	**2 i**	Et	Et	42 (**3 ai**)
9	**2 j**	*t*Bu	*t*Bu	no reaction (**3 aj**)

[a] Reaction conditions (0.10 mmol scale): B(C_6_F_5_)_3_ (20 mol %), Me_3_SiOTf (40 mol %), hydrosilane **2** (2.0 equiv), and *p*‐xylene (0.80 mL) at 150 °C for 2 h. [b] Isolated yield. Mes=mesityl.

The benzyl group in 4‐silapiperidines such as **3 aa** serves as a linchpin for further manipulations (Scheme 4). Debenzylation was achieved by treatment with 1‐chloroethyl chloroformate followed by the reaction of the resulting carbamate with MeOH (**3 aa**→**6**). The benzyl group can also be converted into a benzoyl group by oxidation with KMnO_4_ in the presence of BnNEt_3_Cl (**3 aa**→**7**).

**Scheme 4:**
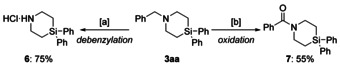
Elaboration of an N‐benzylated 4‐silapiperidine. [a] 1) 1‐chloroethyl chloroformate (1.2 equiv), CH_2_Cl_2_, 0 °C to Δ, 1 h; RT, 20 h; 2) MeOH, Δ, 1 h; [b] KMnO_4_ (3.0 equiv), BnNEt_3_Cl (3.0 equiv), CH_2_Cl_2_, Δ, 3 h.

To gain insight into the reaction mechanism of this two‐fold C(sp^3^)−H silylation, deuterium‐labeling experiments and stoichiometric experiments were performed (Scheme 5). The reaction of **1 a** with Ph_2_SiD_2_ (**2 a**‐*d*_2_) under standard conditions gave **3 aa**‐*d*_3_ in the expected yield with 41 % deuterium incorporation in the benzylic position as well as at the α carbon atoms (Scheme 5, top). This result confirms the known reversible hydride abstraction from C(sp^3^)−H bonds α to an amine nitrogen atom.^[6]^ Importantly, 6 % deuterium incorporation was also detected for the β carbon atoms, which is evidence for hydrogenation of the enamine intermediate. In the case of diethyl‐substituted **1 a**, silylation is faster than this backward reaction. Conversely, di‐*n*‐butyl‐substituted **1 v** shows a different outcome (Scheme 5, top). None of the hypothetical 4‐silapiperidine **3 va**‐*d*_3_ was found (not shown) but instead **1 v**‐*d*_3_ with the usual deuteration in the α‐positions. However, the deuteration grade in the β‐positions was 24 %, demonstrating that enamine hydrogenation is now a competitive if not the only reaction pathway for more hindered alkyl chains. To inspect the influence of the Me_3_SiOTf additive, we mixed **1 a** and B(C_6_F_5_)_3_ in an equimolar ratio (Scheme 5, bottom). This known reaction^[6]^ led to the formation of the three boron species **8 a**–**10 a** in 52 %, 30 %, and 17 % yield, respectively, and this product distribution was not affected by the addition of 1.0 equiv of Me_3_SiOTf.

**Scheme 5:**
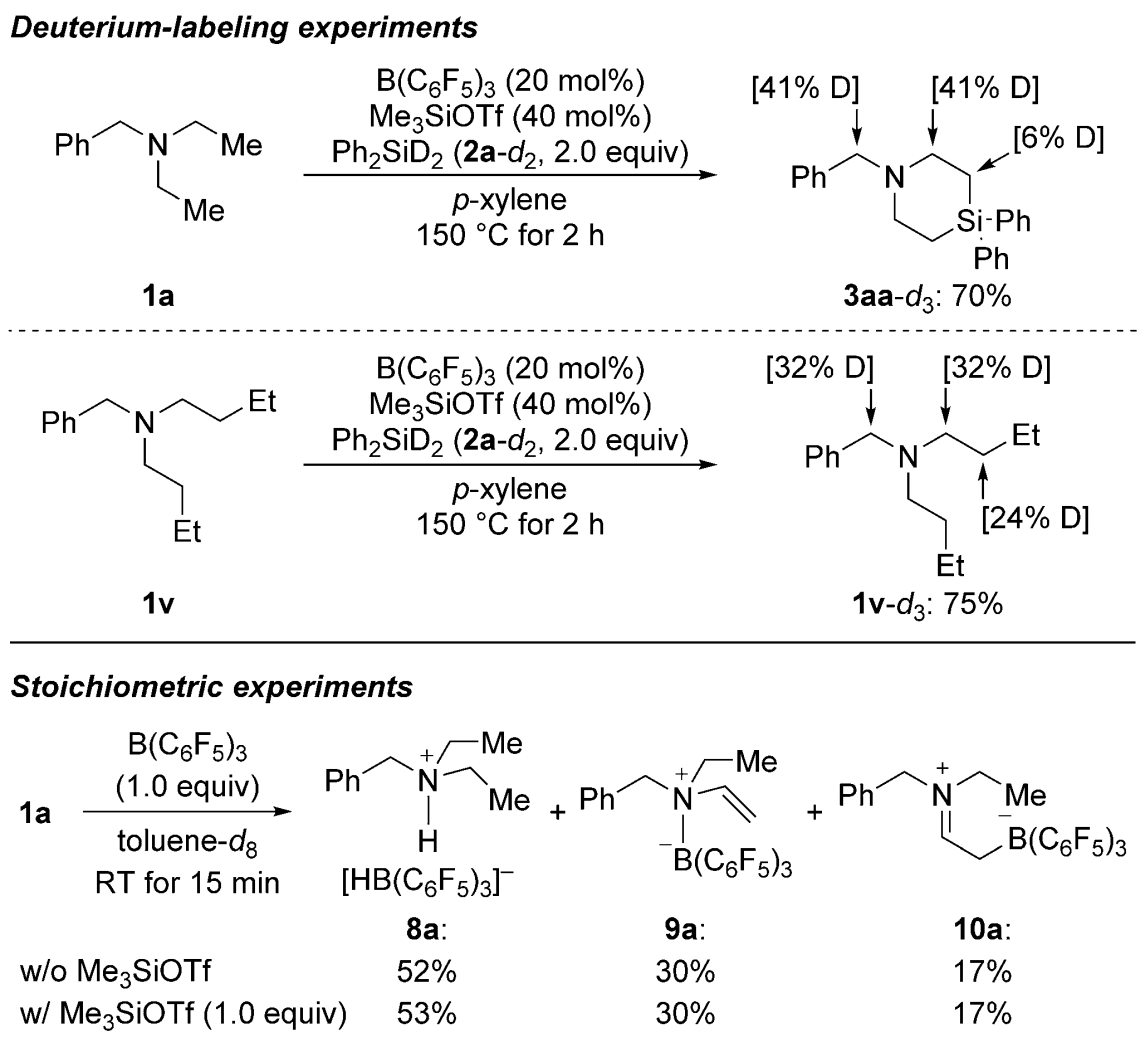
Deuterium‐labeling and stoichiometric experiments. Individual deuteration grades were estimated by ^1^H NMR spectroscopy. The overall deuteration grades of 2.87 D for **3 aa**‐*d*_3_ and 2.98 D for **1 v**‐*d*_3_ were determined by mass spectrometry.

On the basis of the above experimental results and the literature precedent[^6^, ^8^] as well as DFT calculations by Park and Dang,^[8b]^ a plausible reaction mechanism is proposed (Scheme 6). B(C_6_F_5_)_3_ promotes hydride abstraction from the tertiary amine **1 a** to generate the iminium borohydrides **11 a** and **11 a′** in equilibrium. Their subsequent deprotonation by unreacted **1 a** yields enamine **12 a** and FLP‐type dihydrogen adduct **8 a**; these can regenerate the free amine **1 a** and the catalyst B(C_6_F_5_)_3_ along with release of dihydrogen.[^7^, ^19^] The thus‐formed enamine **12 a** then engages in the rate‐determining B(C_6_F_5_)_3_‐catalyzed *inter*molecular hydrosilylation^[8b]^ through the Piers mechanism^[20]^ with **12 a** as a carbon nucleophile (B(C_6_F_5_)_3_→**13 a**→**15 aa**). Alternatively, B(C_6_F_5_)_3_‐activated hydrosilane **13 a** can also react with the amine nitrogen nucleophile **1 a** to equilibrate with silylammonium borohydride **14 aa**, the resting species of the overall process.[^8a^, ^8b^] Initially formed **15 aa** stands in equilibrium with regioisomeric **15 aa′** and **15 aa′′**, and **15 aa′′** can undergo another deprotonation affording enamine **16 aa**. That enamine again enters the catalytic cycle of the B(C_6_F_5_)_3_‐promoted, now *intra*molecular hydrosilylation to eventually arrive at the title compound **3 aa**.

**Scheme 6:**
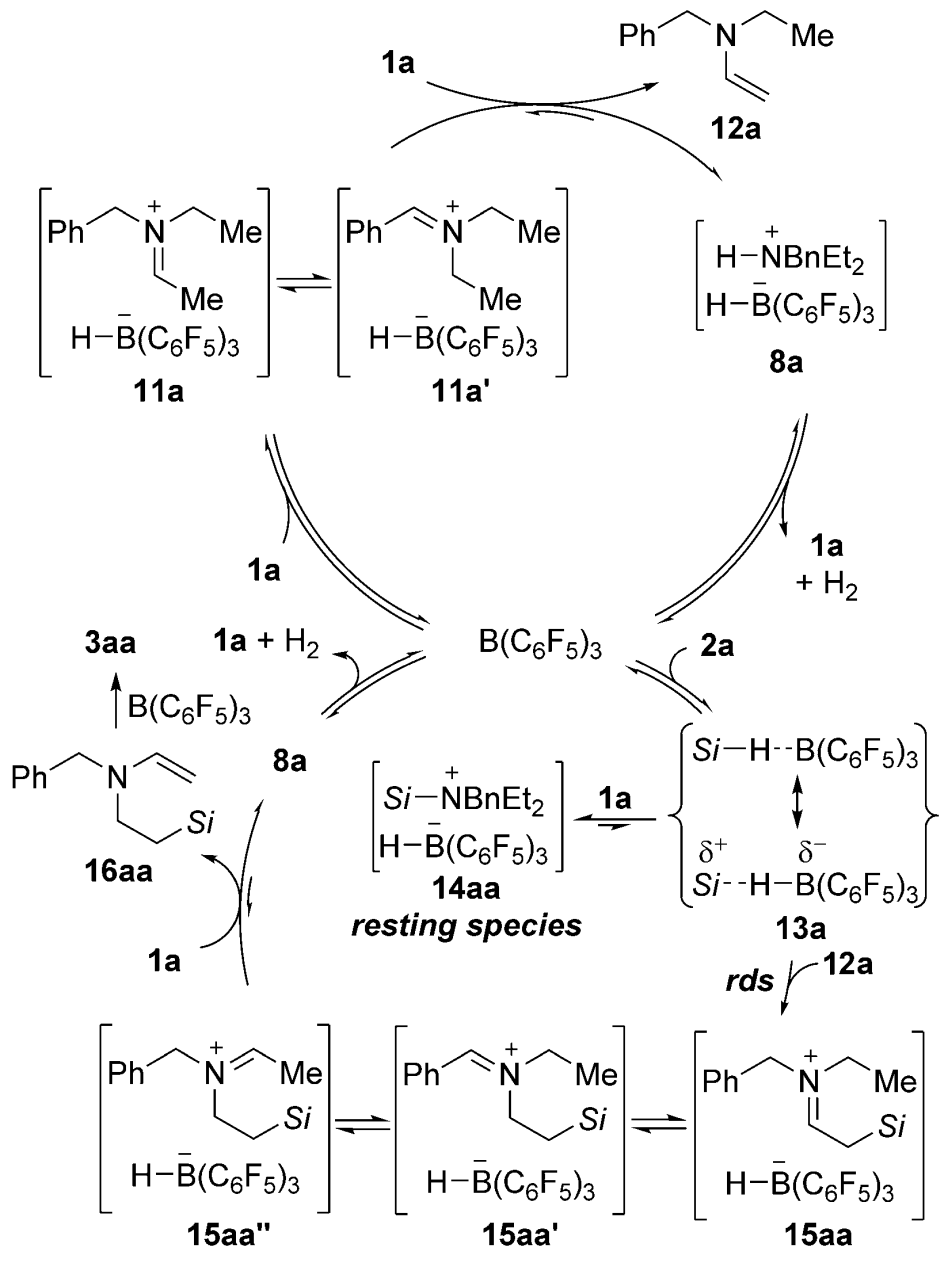
Plausible mechanism for the formation of **3 aa** from **1 a** and **2 a** (*Si*=HPh_2_Si). rds=rate‐determining step.

In summary, we have developed a B(C_6_F_5_)_3_‐catalyzed two‐fold β,β′‐selective (formal) C(sp^3^)−H silylation of acyclic tertiary amines with dihydrosilanes to construct 4‐silapiperidines and its derivatives. The reaction involves two amine‐to‐enamine dehydrogenation reactions each followed by an inter‐ and an intramolecular electrophilic enamine silylation, respectively.

## Conflict of interest

The authors declare no conflict of interest.

## Supporting information

As a service to our authors and readers, this journal provides supporting information supplied by the authors. Such materials are peer reviewed and may be re‐organized for online delivery, but are not copy‐edited or typeset. Technical support issues arising from supporting information (other than missing files) should be addressed to the authors.

Supplementary
