# Supplementary material for: Consecutive β,β′‐Selective C(sp3)−H Silylation of Tertiary Amines with Dihydrosilanes Catalyzed by B(C6F5)3
Source: Angew Chem Int Ed Engl. 2021 Mar 3;60(15):8542–6. doi: 10.1002/anie.202016664 (PMC8048813; doi:10.1002/anie.202016664)
Supplement: Supplementary file 1 — Supplementary [file ANIE-60-8542-s001.pdf]

Angewandte Chemie
Eine Zeitschrift der Gesellschaft Deutscher Chemiker

Supporting Information

# Consecutive β,β′-Selective C(sp$^3$)−H Silylation of Tertiary Amines with Dihydrosilanes Catalyzed by $B(C_6F_5)_3$

*Huaquan Fang, Kaixue Xie, Sebastian Kemper, and Martin Oestreich**

anie_202016664_sm_miscellaneous_information.pdf

# Supporting Information

## Table of Contents

**1 General Experimental Information ... S3**

**2 Experimental Details for the Preparation of *N,N*-Dialkyl Benzylamines ... S5**

2.1 General Procedure for the Preparation of *N*,*N*-Dialkyl Benzylamines ... S5

2.2 Charaterization Data of the *N*,*N*-Dialkyl Benzylamines ... S6

**3 Experiment Details for the Two-Fold C($sp^3$)–H Silylation ... S7**

3.1 General Procedure for the Two-Fold C($sp^3$)–H Silylation ... S7

3.2 Characterization Data of the 4-Silapiperidines ... S8

3.3 Unsuccessful Substrates ... S23

**4 Scale-Up Experiment ... S24**

**5 Elaboration of an *N*-Benzylated 4-Silapiperidine ... S25**

5.1 Debenzylation of Product **3aa** ... S25

5.2 Oxidation of Product **3aa** ... S25

**6 Mechanistic Investigations ... S27**

6.1 $^2$H-Labeling Experiments ... S27

6.2 Stoichiometric Experiments ... S30

**7 NMR Spectra ... S31**

**8 References ... S139**

# 1 General Experimental Information

### Reagents and Solvents

Toluene, benzene, chlorobenzene, chlorobenzene, and *p*-xylene were purified by distillation over $LiAlH_4$ and freshly distilled prior to use. $CH_2Cl_2$ was dried over $CaH_2$ and freshly distilled prior to use. $B(C_6F_5)_3$ was purchased from Boulder Scientific Company, sublimed under vacuum at 130 °C prior to use, and stored in a nitrogen-filled glovebox. Di-*p*-tolylsilane (**2b**),[1] bis(4-(*tert*-butyl)phenyl)silane (**2c**),[2] bis(4-fluorophenyl)silane (**2d**),[2] 2,3-dihydro-1*H*-benzo[*b*]silole (**2g**),[3] and dimesitylsilane (**2k**)[4] were prepared according to literature procedures. $Ph_2SiD_2$[5] was prepared according to literature procedures. All other reagents were purchased from commercial sources and used as received unless specified otherwise.

### Reactions

All manipulations were carried out in a nitrogen-filled glovebox or under an atmosphere of dry nitrogen using standard Schlenk techniques, unless otherwise stated.

### Chromatography

Analytical thin layer chromatography (TLC) was performed on silica gel 60 F254 glass plates by *Merck*. Flash column chromatography was performed on silica gel 60 (40–63 µm, 230–400 mesh, ASTM) by *Grace* using the indicated solvents.

### Nuclear Magnetic Resonance (NMR) Spectroscopy

$^{1}H$, $^{13}C$, and $^{19}F$ NMR spectra were recorded in $CDCl_3$ on Bruker AV500 instruments. Chemical shifts are reported in parts per million (ppm) and are referenced to the residual solvent resonance as the internal standard ($CHCl_3$: δ = 7.26 ppm for $^{1}H$ NMR and $CDCl_3$: δ = 77.16 ppm for $^{13}C$ NMR; toluene: δ = 7.09, 7.01, 6.97, 2.08 ppm for $^{1}H$ NMR). Data are reported as follows: chemical shift, multiplicity (br = broad signal, s = singlet, d = doublet, t = triplet, q = quartet, m = multiplet), coupling constants (Hz), and integration.

### Gas Chromatography (GC)

Gas chromatography (GC) was performed on an *Agilent Technologies* 7820A gas chromatograph equipped with a HP-5 capillary column (30 m × 0.32 mm, 0.25 *µ*m film

thickness) by *Agilent Technologies/ CS-Chromatographie Service* using the following program: nitrogen carrier gas, injection temperature 250 °C, detector temperature 300 °C, flow rate: 1.7 mL/min; temperature program: start temperature 40 °C, heating rate 10 °C/min, end temperature 280 °C for 10 min.

**Gas Chromatography–Mass Spectrometry (GC-MS)**

Gas chromatography–mass spectrometry (GC-MS) was performed on an *Agilent Technologies* 5975C gas chromatograph equipped with an *Agilent Technologies* HP-5 column (30 m × 0.32 mm, 0.25 μm film thickness) using the following program: nitrogen carrier gas, injection temperature 280 °C, detector temperature 280 °C, flow rate: 0.8 mL/min; temperature program: start temperature 40 °C, heating rate 10 °C/min, end temperature 280 °C for 10 min.

**Infrared Spectroscopy**

Infrared (IR) spectra were recorded on an Agilent Technologies Cary 630 FT-IR spectrometer equipped with an ATR unit or a Jasco FT/IR-4100 spectrometer, and the bands are reported in wavenumbers ($cm^{-1}$).

**Mass Spectrometry**

High resolution mass spectrometry (HRMS) analysis was performed by the Analytical Facility at the *Institut für Chemie, Technische Universität Berlin*.

**Compound Nomenclature**

The compound names were generated by the computer program *ChemDraw* according to the guidelines specified by the *International Union of Pure and Applied Chemistry* (IUPAC).

# 2 Experimental Details for the Preparation of *N*,*N*-Dialkyl Benzylamines

## 2.1 General Procedure for the Preparation of *N*,*N*-Dialkyl Benzylamines

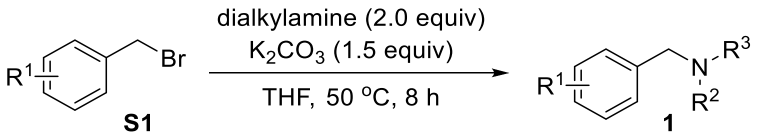


A mixture of benzyl bromides **S1** (10.0 mmol), dialkylamine (20.0 mmol), $K_2CO_3$ (15.0 mmol) in THF (30 mL) was heated at 50 °C for 8 h. The mixture was cooled to room temperature and diluted with methyl *tert*-butyl ether (20 mL) and $H_2O$ (20 mL). Then the mixture was extracted with methyl *tert*-butyl ether (10 mL×3). The combined organic layers were washed with brine (15 mL), dried over $Na_2SO_4$ and the solvent was removed under reduced pressure. The residue was purified by flash column chromatography on silica gel with methyl *tert*-butyl ether / cyclohexane to afford *N*,*N*-dialkyl benzylamines as a colorless liquid.

*N*,*N*-Dialkyl Benzylamines **1a**, **1o–q**, **1t**, and **1u** were purchased from commercial sources and used as received unless specified otherwise.

*N*,*N*-Dialkyl Benzylamines **1b**,[6] **1c**,[7] **1d–e**,[8] **1f**,[6] **1g**,[8] **1h**,[6] **1i–j**,[8] **1k**,[7] **1l**,[6] **1m**,[7] **1n**[6], and **1v**[9] were prepared according to the general procedure and data were consistent with that reported.

## 2.2 Charaterization Data of New *N*,*N*-Dialkyl Benzylamines

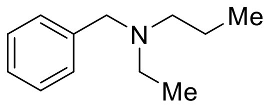

***N*-Benzyl-*N*-ethylpropan-1-amine (1r).** The general procedure was followed with benzyl bromide (1.19 mL, 10.0 mmol), *N*-ethylpropan-1-amine (2.42 mL, 20.0 mmol), $K_2CO_3$ (2.10 g, 15.0 mmol) in THF (30 mL) at 50 °C for 8 h. The residue was purified by flash column chromatography on silica gel with methyl *tert*-butyl ether / cyclohexane = 1 : 10 as the eluent to afford **1r** as a colorless liquid (1.60 g, 90% yield). **$^1$H NMR** (500 MHz, $CDCl_3$): δ 7.36 – 7.30 (m, 4H), 7.24 (t, *J* = 7.0 Hz, 1H), 3.58 (s, 2H), 2.52 (q, *J* = 7.2 Hz, 2H), 2.41 (t, *J* = 7.4 Hz, 2H), 1.54 – 1.47 (m, 2H), 1.05 (t, *J* = 7.2 Hz, 3H), 0.89 (t, *J* = 7.4 Hz, 3H) ppm. **$^{13}$C NMR** (125 MHz, $CDCl_3$): δ 140.3, 129.0, 128.2, 126.7, 58.2, 55.4, 47.4, 20.3, 12.0, 11.9 ppm. **HRMS** (APCI): Calculated for $C_{12}H_{20}N^+$ $[M+H]^+$: 178.1596; Found: 178.1586. **IR** (ATR): ṽ 2961, 2931, 2871, 2794, 1492, 1452, 1368, 1193, 1163, 1073, 1026, 727, 696.

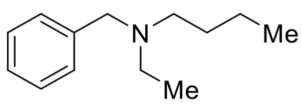

***N*-Benzyl-*N*-ethylbutan-1-amine (1s).** The general procedure was followed with benzyl bromide (1.19 mL, 10.0 mmol), *N*-ethylbutan-1-amine (2.73 mL, 20.0 mmol), $K_2CO_3$ (2.10 g, 15.0 mmol) in THF (30 mL) at 50 °C for 8 h. The residue was purified by flash column chromatography on silica gel with methyl *tert*-butyl ether / cyclohexane = 1 : 10 as the eluent to afford **1s** as a colorless liquid (1.80 g, 94% yield). **$^1$H NMR** (500 MHz, $CDCl_3$): δ 7.35 – 7.33 (m, 4H), 7.25 – 7.21 (m, 1H), 3.57 (s, 2H), 2.51 (q, *J* = 7.1 Hz, 2H), 2.43 (t, *J* = 7.3 Hz, 2H), 1.50 – 1.44 (m, 2H), 1.35 – 1.27 (m, 2H), 1.04 (t, *J* = 7.1 Hz, 3H), 0.89 (t, *J* = 7.4 Hz, 3H) ppm. **$^{13}$C NMR** (125 MHz, $CDCl_3$): δ 140.3, 129.0, 128.2, 126.8, 58.2, 53.1, 47.4, 29.4, 20.8, 14.2, 11.9 ppm. **HRMS** (APCI): Calculated for $C_{13}H_{22}N^+$ $[M+H]^+$: 192.1752; Found: 192.1743. **IR** (ATR): ṽ 2957, 2929, 2869, 2793, 1493, 1452, 1368, 1187, 1160, 1071, 1027, 728, 696.

# 3 Experiment Details for the Two-Fold C(sp$^3$)–H Silylation

## 3.1 General Procedure for the Two-Fold C(sp$^3$)–H Silylation

In a nitrogen-filled glovebox, a 10-mL sealed tube equipped with a magnetic stir bar was charged with the desired amount of amine, hydrosilane, solvent, additive, and $B(C_6F_5)_3$. The sealed tube was fitted with a cap, and the reaction stirred at required temperature for indicated time in a preheated oil bath. After the resulting reaction mixture was cooled to room temperature, the volatile were removed under reduced pressure. *In the case of $R_3$SiOTf as an additive, the solution was neutralized upon stirring with NaOH (5 mL, 10% aq.) for 30 min. The mixture was extracted with methyl tert-butyl ether (10 mL×3). The combined organic layers were washed with brine (15 mL), dried over $Na_2SO_4$, and the solvent was removed under reduced pressure.* Mesitylene (0.500 equiv) was added as an internal standard, and the yield was determined by $^1$H NMR spectroscopy. The residue was purified by a sequence of flash column chromatography on silica gel with methyl *tert*-butyl ether / cyclohexane for *N*-benzyl-substituted 4-silapiperidines or MeOH / methyl *tert*-butyl ether for *N*-alkyl-substituted 4-sila-piperidines as the eluent and Kugelrohr distillation.

### 3.2 Characterization Data of the 4-Silapiperidines

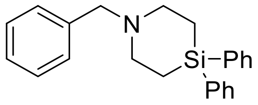


**1-Benzyl-4,4-diphenyl-1,4-azasilinane (3aa)**. The general procedure was followed with *N*-benzyl-*N*-ethylethanamine (**1a**, 8.20 mg, 0.0500 mmol), $Ph_2SiH_2$ (**2a**, 18.4 mg, 0.100 mmol), *p*-xylene (0.800 mL), $Me_3SiOTf$ (3.60 µL, 20.0 µmol) and $B(C_6F_5)_3$ (5.10 mg, 10.0 µmol) at 150 °C for 2 h. The residue was purified by a sequence of flash column chromatography on silica gel with methyl *tert*-butyl ether / cyclohexane = 1 : 10 as the eluent and Kugelrohr distillation (200 °C, 2 mbar) to afford the 4-silapiperidine product as a colorless viscous liquid (12.5 mg, 73% yield). **$^1$H NMR** (500 MHz, $CDCl_3$): δ 7.60 – 7.57 (m, 4H), 7.44 – 7.33 (m, 10H), 7.29 – 7.25 (m, 1H), 3.62 (s, 2H), 2.86 (t, *J* = 6.3 Hz, 4H), 1.40 (t, *J* = 6.3 Hz, 4H) ppm. **$^{13}$C NMR** (125 MHz, $CDCl_3$): δ 139.6, 136.0, 134.8, 129.5, 128.9, 128.3, 128.0, 126.9, 62.8, 52.4, 11.5 ppm. **$^1$H/$^{29}$Si HMQC NMR** (500/99 MHz, $CDCl_3$, 298 K, optimized for *J* = 7 Hz): δ 7.60 – 7.57/–15.4, 2.86/–15.4, 1.40/–15.4 ppm. **HRMS** (APCI): Calculated for $C_{23}H_{26}NSi^+$ $[M+H]^+$: 344.1835; Found: 344.1828. **IR** (ATR): ṽ 2920, 2891, 2794, 2758, 1452, 1226, 1185, 1109, 968, 864, 724, 694. Spectral data is in agreement with published data.[10]

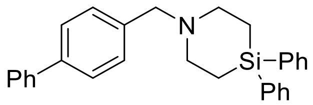


**1-([1,1'-Biphenyl]-4-ylmethyl)-4,4-diphenyl-1,4-azasilinane (3ba)**. The general procedure was followed with *N*-([1,1'-biphenyl]-4-ylmethyl)-*N*-ethylethanamine (**1b**, 23.9 mg, 0.100 mmol), $Ph_2SiH_2$ (**2a**, 36.8 mg, 0.200 mmol), *p*-xylene (0.800 mL), $Me_3SiOTf$ (7.20 µL, 40.0 µmol) and $B(C_6F_5)_3$ (10.2 mg, 20.0 µmol) at 150 °C for 2 h. The residue was purified by a sequence of flash column chromatography on silica gel with methyl *tert*-butyl ether / cyclohexane = 1 : 10 as the eluent and Kugelrohr distillation (240 °C, 2 mbar) to afford the 4-silapiperidine product as a colorless viscous liquid (23.9 mg, 57% yield). **$^1$H NMR** (500 MHz, $CDCl_3$): δ 7.65 – 7.58 (m, 8H), 7.48 – 7.35 (m, 11H), 3.66 (s, 2H), 2.89 (t, *J* = 6.5 Hz, 4H), 1.43 (t, *J* = 6.5 Hz, 4H) ppm. **$^{13}$C NMR** (125 MHz, $CDCl_3$): δ 141.2, 139.9, 138.7, 136.0, 134.8, 129.5, 129.3, 128.9, 128.1, 127.2, 127.2, 127.1, 62.5, 52.5, 11.5 ppm. **$^1$H/$^{29}$Si HMQC NMR** (500/99 MHz, $CDCl_3$, 298 K, optimized for *J* = 7 Hz): δ 7.58/–15.5, 2.89/–15.5, 1.43/–15.5 ppm. **HRMS** (APCI): Calculated for $C_{29}H_{30}NSi^+$ $[M+H]^+$: 420.2148; Found: 420.2138. **IR** (ATR): ṽ 2920, 2892, 2792, 2758, 1485, 1426, 1226, 1185, 1110, 1007, 970, 867, 727, 695.

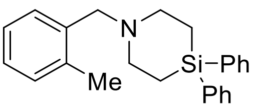


**1-(2-Methylbenzyl)-4,4-diphenyl-1,4-azasilinane (3ca)**. The general procedure was followed with *N*-ethyl-*N*-(2-methylbenzyl)ethanamine (**1c**, 17.7 mg, 0.100 mmol), $Ph_2SiH_2$ (**2a**, 36.8 mg, 0.200 mmol), *p*-xylene (0.800 mL), $Me_3SiOTf$ (7.20 µL, 40.0 µmol) and $B(C_6F_5)_3$ (10.2 mg, 20.0 µmol) at 150 °C for 2 h. The residue was purified by a sequence of flash column chromatography on silica gel with methyl *tert*-butyl ether / cyclohexane = 1 : 10 as the eluent and Kugelrohr distillation (220 °C, 2 mbar) to afford the 4-silapiperidine product as a colorless viscous liquid (21.1 mg, 59% yield). **$^1$H NMR** (500 MHz, $CDCl_3$): δ 7.58 – 7.55 (m, 4H), 7.42 – 7.33 (m, 7H), 7.18 – 7.14 (m, 3H), 3.52 (s, 2H), 2.83 (t, *J* = 5.2 Hz, 4H), 2.37 (s, 3H), 1.36 (t, *J* = 5.2 Hz, 4H) ppm. **$^{13}$C NMR** (125 MHz, $CDCl_3$): δ 137.8, 137.5, 136.1, 134.8, 130.3, 129.5, 128.1, 126.9, 125.6, 60.7, 52.6, 19.4, 11.6 ppm. **$^1$H/$^{29}$Si HMQC NMR** (500/99 MHz, $CDCl_3$, 298 K, optimized for *J* = 7 Hz): δ 7.58 – 7.55/–15.4, 2.83/–15.4, 1.36/–15.4 ppm. **HRMS** (APCI): Calculated for $C_{24}H_{28}NSi^+$ [M+H]$^+$: 358.1991; Found: 358.1984. **IR** (ATR): ṽ 2920, 2889, 2795, 2758, 1459, 1426, 1226, 1185, 1110, 969, 865, 728, 697.

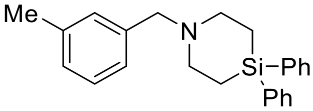


**1-(3-Methylbenzyl)-4,4-diphenyl-1,4-azasilinane (3da)**. The general procedure was followed with *N*-ethyl-*N*-(3-methylbenzyl)ethanamine (**1d**, 17.7 mg, 0.100 mmol), $Ph_2SiH_2$ (**2a**, 36.8 mg, 0.200 mmol), *p*-xylene (0.800 mL), $Me_3SiOTf$ (7.20 µL, 40.0 µmol) and $B(C_6F_5)_3$ (10.2 mg, 20.0 µmol) at 150 °C for 2 h. The residue was purified by a sequence of flash column chromatography on silica gel with methyl *tert*-butyl ether / cyclohexane = 1 : 10 as the eluent and Kugelrohr distillation (220 °C, 2 mbar) to afford the 4-silapiperidine product as a colorless viscous liquid (20.8 mg, 58% yield). **$^1$H NMR** (500 MHz, $CDCl_3$): δ 7.58 – 7.56 (m, 4H), 7.42 – 7.36 (m, 6H), 7.24 – 7.16 (m, 3H), 7.08 (d, *J* = 7.3 Hz, 1H), 3.58 (s, 2H), 2.85 (t, *J* = 6.0 Hz, 4H), 2.37 (s, 3H), 1.40 (t, *J* = 6.1 Hz, 4H) ppm. **$^{13}$C NMR** (125 MHz, $CDCl_3$): δ 137.9, 136.0, 134.8, 129.8, 129.5, 128.2, 128.1, 127.8, 126.1, 62.7, 52.4, 21.6, 11.3 ppm. **$^1$H/$^{29}$Si HMQC NMR** (500/99 MHz, $CDCl_3$, 298 K, optimized for *J* = 7 Hz): δ 7.58 – 7.56/–15.8, 2.85/–15.8, 1.40/–15.8 ppm. **HRMS** (APCI): Calculated for $C_{24}H_{28}NSi^+$ [M+H]$^+$: 358.1991; Found: 358.1983. **IR** (ATR): ṽ 3045, 2921, 2794, 1427, 1389, 1227, 1112, 970, 865, 728, 698.

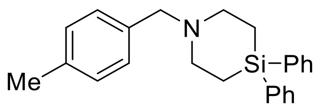


**1-(4-Methylbenzyl)-4,4-diphenyl-1,4-azasilinane (3ea)**. The general procedure was followed with *N*-ethyl-*N*-(4-methylbenzyl)ethanamine (**1e**, 17.7 mg, 0.100 mmol), $Ph_2SiH_2$ (**2a**, 36.8 mg, 0.200 mmol), *p*-xylene (0.800 mL), $Me_3SiOTf$ (7.20 µL, 40.0 µmol) and $B(C_6F_5)_3$ (10.2 mg, 20.0 µmol) at 150 °C for 2 h. The residue was purified by a sequence of flash column chromatography on silica gel with methyl *tert*-butyl ether / cyclohexane = 1 : 10 as the eluent and Kugelrohr distillation (220 °C, 2 mbar) to afford the 4-silapiperidine product as a colorless viscous liquid (21.8 mg, 61% yield). **$^1$H NMR** (500 MHz, $CDCl_3$): δ 7.58 – 7.56 (m, 4H), 7.42 – 7.36 (m, 6H), 7.26 (d, *J* = 8.0 Hz, 2H), 7.14 (d, *J* = 7.9 Hz, 2H), 3.58 (s, 2H), 2.84 (t, *J* = 6.1 Hz, 4H), 2.36 (s, 3H), 1.39 (t, *J* = 6.2 Hz, 4H) ppm. **$^{13}$C NMR** (125 MHz, $CDCl_3$): δ 136.6, 136.1, 135.9, 134.8, 129.5, 129.0 (2C), 128.1, 62.5, 52.3, 21.2, 11.4 ppm. **$^1$H/$^{29}$Si HMQC NMR** (500/99 MHz, $CDCl_3$, 298 K, optimized for *J* = 7 Hz): δ 7.58 – 7.56/–15.6, 2.84/–15.6, 1.39/–15.6 ppm. **HRMS** (APCI): Calculated for $C_{24}H_{28}NSi^+$ $[M+H]^+$: 358.1991; Found: 358.1985. **IR** (ATR): ṽ 3045, 2920, 2792, 1426, 1186, 1111, 970, 867, 728, 698.

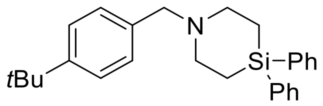


**1-(4-(*tert*-Butyl)benzyl)-4,4-diphenyl-1,4-azasilinane (3fa)**. The general procedure was followed with *N*-(4-(*tert*-butyl)benzyl)-*N*-ethylethanamine (**1f**, 21.9 mg, 0.100 mmol), $Ph_2SiH_2$ (**2a**, 36.8 mg, 0.200 mmol), *p*-xylene (0.800 mL), $Me_3SiOTf$ (7.20 µL, 40.0 µmol) and $B(C_6F_5)_3$ (10.2 mg, 20.0 µmol) at 150 °C for 2 h. The residue was purified by a sequence of flash column chromatography on silica gel with methyl *tert*-butyl ether / cyclohexane = 1 : 10 as the eluent and Kugelrohr distillation (230 °C, 2 mbar) to afford the 4-silapiperidine product as a colorless viscous liquid (26.7 mg, 67% yield). **$^1$H NMR** (500 MHz, $CDCl_3$): δ 7.59 – 7.57 (m, 4H), 7.43 – 7.35 (m, 8H), 7.30 (d, *J* = 8.0 Hz, 2H), 3.60 (s, 2H), 2.86 (t, *J* = 5.7 Hz, 4H), 1.41 (t, *J* = 5.7 Hz, 4H), 1.35 (s, 9H) ppm. **$^{13}$C NMR** (125 MHz, $CDCl_3$): δ 149.9, 136.0, 135.9, 134.8, 129.5, 128.7, 128.1, 125.2, 62.4, 52.4, 34.6, 31.5, 11.3 ppm. **$^1$H/$^{29}$Si HMQC NMR** (500/99 MHz, $CDCl_3$, 298 K, optimized for *J* = 7 Hz): δ 7.59 – 7.57/–15.5, 2.86/–15.5, 1.41/–15.5 ppm. **HRMS** (APCI): Calculated for $C_{27}H_{34}NSi^+$ $[M+H]^+$: 400.2461; Found: 400.2451. **IR** (ATR): ṽ 3066, 2958, 2792, 1465, 1426, 1389, 1226, 1109, 970, 906, 867, 726, 696.

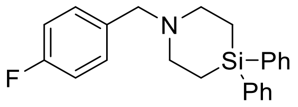

**1-(4-Fluorobenzyl)-4,4-diphenyl-1,4-azasilinane (3ga)**. The general procedure was followed with *N*-ethyl-*N*-(4-fluorobenzyl)ethanamine (**1g**, 18.1 mg, 0.100 mmol), $Ph_2SiH_2$ (**2a**, 36.8 mg, 0.200 mmol), *p*-xylene (0.800 mL), $Me_3SiOTf$ (7.20 µL, 40.0 µmol) and $B(C_6F_5)_3$ (10.2 mg, 20.0 µmol) at 150 °C for 2 h. The residue was purified by a sequence of flash column chromatography on silica gel with methyl *tert*-butyl ether / cyclohexane = 1 : 10 as the eluent and Kugelrohr distillation (220 °C, 2 mbar) to afford the 4-silapiperidine product as a colorless viscous liquid (25.3 mg, 70% yield). $^1$**H NMR** (500 MHz, $CDCl_3$): δ 7.56 – 7.54 (m, 4H), 7.41 – 7.35 (m, 6H), 7.32 – 7.29 (m, 2H), 7.02 – 6.98 (m, 2H), 3.55 (s, 2H), 2.81 (t, *J* = 6.1 Hz, 4H), 1.37 (t, *J* = 5.9 Hz, 4H) ppm. $^{13}$**C NMR** (125 MHz, $CDCl_3$): δ 162.0 (d, *J* = 244.6 Hz), 135.9, 135.3, 134.8, 130.3 (d, *J* = 7.9 Hz), 129.5, 128.1, 115.0 (d, *J* = 21.1 Hz), 62.0, 52.3, 11.5 ppm. $^{19}$**F NMR** (471 MHz, $CDCl_3$): δ –116.2 ppm. $^1$**H/**$^{29}$**Si HMQC NMR** (500/99 MHz, $CDCl_3$, 298 K, optimized for *J* = 7 Hz): δ 7.56 – 7.54/–15.6, 2.81/–15.6, 1.37/–15.6 ppm. **HRMS** (APCI): Calculated for $C_{23}H_{25}FNSi^+$ $[M+H]^+$: 362.1740; Found: 362.1727. **IR** (ATR): ṽ 3066, 2922, 2793, 1602, 1506, 1427, 1221, 1112, 971, 866, 729, 700.

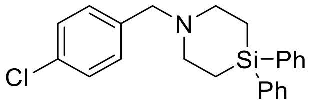

**1-(4-Chlorobenzyl)-4,4-diphenyl-1,4-azasilinane (3ha)**. The general procedure was followed with *N*-ethyl-*N*-(4-chlorobenzyl)ethanamine (**1h**, 19.7 mg, 0.100 mmol), $Ph_2SiH_2$ (**2a**, 36.8 mg, 0.200 mmol), *p*-xylene (0.800 mL), $Me_3SiOTf$ (7.20 µL, 40.0 µmol) and $B(C_6F_5)_3$ (10.2 mg, 20.0 µmol) at 150 °C for 2 h. The residue was purified by a sequence of flash column chromatography on silica gel with methyl *tert*-butyl ether / cyclohexane = 1 : 10 as the eluent and Kugelrohr distillation (230 °C, 2 mbar) to afford the 4-silapiperidine product as a colorless viscous liquid (24.6 mg, 65% yield). $^1$**H NMR** (500 MHz, $CDCl_3$): δ 7.56 – 7.54 (m, 4H), 7.42 – 7.35 (m, 6H), 7.30 – 7.27 (m, 4H), 3.56 (s, 2H), 2.82 (t, *J* = 6.0 Hz, 4H), 1.38 (t, *J* = 6.0 Hz, 4H) ppm. $^{13}$**C NMR** (125 MHz, $CDCl_3$): δ 137.8, 135.7, 134.8, 132.7, 130.3, 129.6, 128.5, 128.1, 62.0, 52.4, 11.4 ppm. $^1$**H/**$^{29}$**Si HMQC NMR** (500/99 MHz, $CDCl_3$, 298 K, optimized for *J* = 7 Hz): δ 7.56 – 7.54/–16.0, 2.82/–16.0, 1.38/–16.0 ppm. **HRMS** (APCI): Calculated for $C_{23}H_{25}ClNSi^+$ $[M+H]^+$: 378.1445; Found: 378.1440. **IR** (ATR): ṽ 3066, 2922, 2796, 1488, 1427, 1112, 1015, 971, 868, 699.

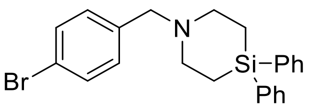

**1-(4-Bromobenzyl)-4,4-diphenyl-1,4-azasilinane (3ia)**. The general procedure was followed with *N*-ethyl-*N*-(4-bromobenzyl)ethanamine (**1i**, 24.2 mg, 0.100 mmol), $Ph_2SiH_2$ (**2a**, 36.8 mg, 0.200 mmol), *p*-xylene (0.800 mL), $Me_3SiOTf$ (7.20 μL, 40.0 μmol) and $B(C_6F_5)_3$ (10.2 mg, 20.0 μmol) at 150 °C for 2 h. The residue was purified by a sequence of flash column chromatography on silica gel with methyl *tert*-butyl ether / cyclohexane = 1 : 10 as the eluent and Kugelrohr distillation (230 °C, 2 mbar) to afford the 4-silapiperidine product as a colorless viscous liquid (28.8 mg, 68% yield). **$^1$H NMR** (500 MHz, $CDCl_3$): δ 7.57 – 7.54 (m, 4H), 7.45 – 7.36 (m, 8H), 7.24 (d, *J* = 8.3 Hz, 2H), 3.54 (s, 2H), 2.82 (t, *J* = 6.2 Hz, 4H), 1.38 (t, *J* = 6.2 Hz, 4H) ppm. **$^{13}$C NMR** (125 MHz, $CDCl_3$): δ 138.3, 135.7, 134.8, 131.4, 130.6, 129.6, 128.1, 120.8, 62.1, 52.4, 11.4 ppm. **$^1$H/$^{29}$Si HMQC NMR** (500/99 MHz, $CDCl_3$, 298 K, optimized for *J* = 7 Hz): δ 7.57 – 7.54/–15.7, 2.82/–15.5, 1.38/–15.5 ppm. **HRMS** (APCI): Calculated for $C_{23}H_{25}BrNSi^+$ [M+H]$^+$: 422.0940; Found: 422.0933. **IR** (ATR): ṽ 3066, 2922, 2795, 1485, 1427, 1388, 1112, 1011, 971, 699.

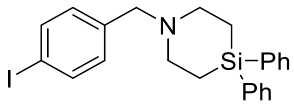

**1-(4-Iodobenzyl)-4,4-diphenyl-1,4-azasilinane (3ja)**. The general procedure was followed with *N*-ethyl-*N*-(4-iodobenzyl)ethanamine (**1j**, 28.9 mg, 0.100 mmol), $Ph_2SiH_2$ (**2a**, 36.8 mg, 0.200 mmol), *p*-xylene (0.800 mL), $Me_3SiOTf$ (7.20 μL, 40.0 μmol) and $B(C_6F_5)_3$ (10.2 mg, 20.0 μmol) at 150 °C for 2 h. The residue was purified by a sequence of flash column chromatography on silica gel with methyl *tert*-butyl ether / cyclohexane = 1 : 10 as the eluent and Kugelrohr distillation (220 °C, 2 mbar) to afford the 4-silapiperidine product as a colorless viscous liquid (30.1 mg, 64% yield). **$^1$H NMR** (500 MHz, $CDCl_3$): δ 7.65 (d, *J* = 8.3 Hz, 2H), 7.57 – 7.55 (m, 4H), 7.43 – 7.36 (m, 6H), 7.12 (d, *J* = 8.3 Hz, 2H), 3.53 (s, 2H), 2.82 (t, *J* = 6.1 Hz, 4H), 1.38 (t, *J* = 6.2 Hz, 4H) ppm. **$^{13}$C NMR** (125 MHz, $CDCl_3$): δ 139.3, 137.4, 135.8, 134.8, 130.9, 129.5, 128.1, 92.3, 62.2, 52.4, 11.5 ppm. **$^1$H/$^{29}$Si HMQC NMR** (500/99 MHz, $CDCl_3$, 298 K, optimized for *J* = 7 Hz): δ 7.57 – 7.55/–15.8, 2.82/–15.8, 1.38/–15.8 ppm. **HRMS** (APCI): Calculated for $C_{23}H_{25}INSi^+$ [M+H]$^+$: 470.0801; Found: 470.0782. **IR** (ATR): ṽ 3065, 2920, 2792, 1586, 1480, 1425, 1386, 1110, 969, 906, 865, 726, 695.

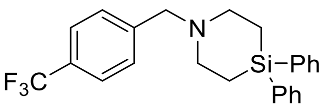


**4,4-Diphenyl-1-(4-(trifluoromethyl)benzyl)-1,4-azasilinane (3ka)**. The general procedure was followed with *N*-ethyl-*N*-(4-(trifluoromethyl)benzyl)ethanamine (**1k**, 23.1 mg, 0.100 mmol), $Ph_2SiH_2$ (**2a**, 36.8 mg, 0.200 mmol), *p*-xylene (0.800 mL), $Me_3SiOTf$ (7.20 µL, 40.0 µmol) and $B(C_6F_5)_3$ (10.2 mg, 20.0 µmol) at 150 °C for 2 h. The residue was purified by a sequence of flash column chromatography on silica gel with methyl *tert*-butyl ether / cyclohexane = 1 : 10 as the eluent and Kugelrohr distillation (220 °C, 2 mbar) to afford the 4-silapiperidine product as a colorless viscous liquid (21.1 mg, 51% yield). **$^1$H NMR** (500 MHz, $CDCl_3$): δ 7.60 – 7.57 (m, 6H), 7.50 (d, *J* = 8.1 Hz, 2H), 7.44 – 7.38 (m, 6H), 3.65 (s, 2H), 2.84 (t, *J* = 6.2 Hz, 4H), 1.41 (t, *J* = 6.2 Hz, 4H) ppm. **$^{13}$C NMR** (125 MHz, $CDCl_3$): δ 143.9, 135.7, 134.8, 129.6, 129.2 (q, *J* = 32.4 Hz), 128.9, 128.1, 125.3 (q, *J* = 3.6 Hz), 124.5 (q, *J* = 271.9 Hz), 62.3, 52.6, 11.5 ppm. **$^{19}$F NMR** (471 MHz, $CDCl_3$): δ –62.3 ppm. **$^1$H/$^{29}$Si HMQC NMR** (500/99 MHz, $CDCl_3$, 298 K, optimized for *J* = 7 Hz): δ 7.60 – 7.57/–15.8, 2.84/–15.8, 1.41/–15.8 ppm. **HRMS** (APCI): Calculated for $C_{24}H_{25}F_3NSi^+$ $[M+H]^+$: 412.1708; Found: 412.1694. **IR** (ATR): ṽ 3067, 2922, 2795, 1426, 1321, 1159, 1110, 1063, 1017, 970, 867, 697.

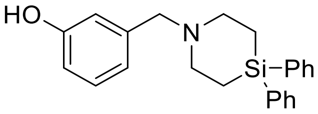


**3-((4,4-Diphenyl-1,4-azasilinan-1-yl)methyl)phenol (3la)**. The general procedure was followed with *N*-ethyl-*N*-(3-methoxybenzyl)ethanamine (**1l**, 19.3 mg, 0.100 mmol), $Ph_2SiH_2$ (**2a**, 36.8 mg, 0.200 mmol), *p*-xylene (0.800 mL), $Me_3SiOTf$ (7.20 µL, 40.0 µmol) and $B(C_6F_5)_3$ (10.2 mg, 20.0 µmol) at 150 °C for 2 h. The residue was purified by a sequence of flash column chromatography on silica gel with methyl *tert*-butyl ether / cyclohexane = 1 : 1 as the eluent and Kugelrohr distillation (220 °C, 2 mbar) to afford the 4-silapiperidine product as a colorless viscous liquid (18.1 mg, 50% yield). **$^1$H NMR** (500 MHz, $CDCl_3$): δ 7.53 – 7.51 (m, 4H), 7.41 – 7.34 (m, 6H), 7.15 (t, *J* = 7.8 Hz, 1H), 6.84 – 6.81 (m, 2H), 6.76 (dd, *J* = 8.0, 1.8 Hz, 1H), 6.07 (br, 1H), 3.56 (s, 2H), 2.87 (t, *J* = 6.2 Hz, 4H), 1.39 (t, *J* = 6.2 Hz, 4H) ppm. **$^{13}$C NMR** (125 MHz, $CDCl_3$): δ 156.7, 139.3, 135.3, 134.9, 129.6, 129.5, 128.1, 121.5, 117.0, 115.2, 62.6, 52.4, 10.8 ppm. **$^1$H/$^{29}$Si HMQC NMR** (500/99 MHz, $CDCl_3$, 298 K, optimized for *J* = 7 Hz): δ 7.53 – 7.51/–16.0, 2.87/–16.0, 1.39/–16.0 ppm. **HRMS** (APCI): Calculated for $C_{23}H_{26}NOSi^+$

[M+H]$^+$: 360.1784; Found: 360.1778. **IR** (ATR): ṽ 3295, 3045, 2924, 2084, 1588, 1454, 1265, 1112, 972, 908, 861, 699.

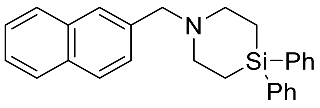


**1-(Naphthalen-2-ylmethyl)-4,4-diphenyl-1,4-azasilinane (3ma)**. The general procedure was followed with *N*-ethyl-*N*-(naphthalen-2-ylmethyl)ethanamine (**1m**, 21.3 mg, 0.100 mmol), $Ph_2SiH_2$ (**2a**, 36.8 mg, 0.200 mmol), *p*-xylene (0.800 mL), $Me_3SiOTf$ (7.20 µL, 40.0 µmol) and $B(C_6F_5)_3$ (10.2 mg, 20.0 µmol) at 150 °C for 2 h. The residue was purified by a sequence of flash column chromatography on silica gel with methyl *tert*-butyl ether / cyclohexane = 1 : 10 as the eluent and Kugelrohr distillation (240 °C, 2 mbar) to afford the 4-silapiperidine product as a colorless viscous liquid (18.2 mg, 46% yield). **$^1$H NMR** (500 MHz, $CDCl_3$): δ 7.86 – 7.83 (m, 3H), 7.78 (s, 1H), 7.60 – 7.56 (m, 5H), 7.51 – 7.45 (m, 2H), 7.44 – 7.38 (m, 6H), 3.78 (s, 2H), 2.91 (t, *J* = 6.2 Hz, 4H), 1.43 (t, *J* = 6.3 Hz, 4H) ppm. **$^{13}$C NMR** (125 MHz, $CDCl_3$): δ 137.1, 136.0, 134.8, 133.5, 132.9, 129.5, 128.1, 128.0, 127.8, 127.8, 127.4, 127.4, 126.0, 125.6, 62.9, 52.5, 11.4 ppm. **$^1$H/$^{29}$Si HMQC NMR** (500/99 MHz, $CDCl_3$, 298 K, optimized for *J* = 7 Hz): δ 7.60 – 7.56/–15.7, 2.91/–15.7, 1.43/–15.7 ppm. **HRMS** (APCI): Calculated for $C_{27}H_{28}NSi^+$ [M+H]$^+$: 394.1991; Found: 394.1982. **IR** (ATR): ṽ 3046, 2920, 2798, 1426, 1328, 1110, 971, 864, 727, 698.

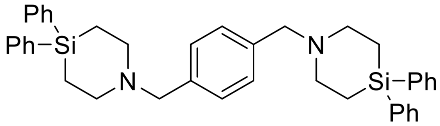


**1,4-Bis((4,4-diphenyl-1,4-azasilinan-1-yl)methyl)benzene (3na)**. The general procedure was followed with *N,N'*-(1,4-phenylenebis(methylene))bis(*N*-ethylethanamine) (**1n**, 24.8 mg, 0.100 mmol), $Ph_2SiH_2$ (**2a**, 73.6 mg, 0.400 mmol), *p*-xylene (0.800 mL), $Me_3SiOTf$ (14.4 µL, 80.0 µmol) and $B(C_6F_5)_3$ (20.4 mg, 40.0 µmol) at 150 °C for 2 h. The residue was purified by a sequence of flash column chromatography on silica gel with methyl *tert*-butyl ether / cyclohexane = 1 : 1 as the eluent and Kugelrohr distillation (250 °C, 0.2 mbar) to afford the 4-silapiperidine product as a colorless viscous liquid (28.7 mg, 47% yield). **$^1$H NMR** (500 MHz, $CDCl_3$): δ 7.56 (d, *J* = 7.4 Hz, 8H), 7.41 – 7.36 (m, 12H), 7.29 (s, 4H), 3.59 (s, 4H), 2.84 (t, *J* = 5.5 Hz, 8H), 1.38 (t, *J* = 5.5 Hz, 8H) ppm. **$^{13}$C NMR** (125 MHz, $CDCl_3$): δ 138.0, 136.0, 134.8, 129.5, 128.8, 128.1, 62.5, 52.4, 11.4 ppm. **$^1$H/$^{29}$Si HMQC NMR** (500/99 MHz, $CDCl_3$, 298 K,

optimized for $J$ = 7 Hz): δ 7.56/–15.8, 2.84/–15.8, 1.38/–15.8 ppm. **HRMS** (APCI): Calculated for $C_{40}H_{45}N_2Si_2^+$ [M+H]$^+$: 609.3121; Found: 609.3119. **IR** (ATR): ṽ 2921, 2893, 2792, 2757, 1426, 1387, 1226, 1185, 1110, 969, 906, 866, 725, 696.

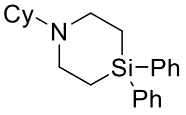


**1-Cyclohexyl-4,4-diphenyl-1,4-azasilinane (3oa)**. The general procedure was followed with *N,N*-diethylcyclohexanamine (**1o**, 15.5 mg, 0.100 mmol), $Ph_2SiH_2$ (**2a**, 36.8 mg, 0.200 mmol), *p*-xylene (0.800 mL), $Me_3SiOTf$ (7.20 μL, 40.0 μmol) and $B(C_6F_5)_3$ (10.2 mg, 20.0 μmol) at 150 °C for 2 h. The residue was purified by a sequence of flash column chromatography on silica gel with MeOH / methyl *tert*-butyl ether = 1 : 10 as the eluent and Kugelrohr distillation (180 °C, 2 mbar) to afford the 4-silapiperidine product as a colorless viscous liquid (12.8 mg, 38% yield). **$^1$H NMR** (500 MHz, $CDCl_3$): δ 7.56 – 7.54 (m, 4H), 7.40 – 7.34 (m, 6H), 2.97 (t, $J$ = 6.5 Hz, 4H), 2.52 (t, $J$ = 7.7 Hz, 1H), 1.86 – 1.77 (m, 4H), 1.62 (d, $J$ = 13.8 Hz, 1H), 1.38 (t, $J$ = 6.5 Hz, 4H), 1.28 – 1.17 (m, 4H), 1.11 – 1.03 (m, 1H) ppm. **$^{13}$C NMR** (125 MHz, $CDCl_3$): δ 136.0, 134.9, 129.5, 128.1, 64.4, 48.8, 29.1, 26.5, 26.3, 11.8 ppm. **$^1$H/$^{29}$Si HMQC NMR** (500/99 MHz, $CDCl_3$, 298 K, optimized for $J$ = 7 Hz): δ 7.56 – 7.54/–14.6, 2.97/–14.6, 1.38/–14.6 ppm. **HRMS** (APCI): Calculated for $C_{22}H_{30}NSi^+$ [M+H]$^+$: 336.2148; Found: 336.2142. **IR** (ATR): ṽ 3065, 2922, 2850, 2795, 1374, 1227, 1110, 987, 863, 728, 698.

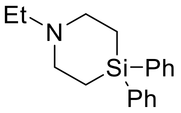


**1-Ethyl-4,4-diphenyl-1,4-azasilinane (3pa)**. The general procedure was followed with triethylamine (**1p**, 10.1 mg, 0.100 mmol), $Ph_2SiH_2$ (**2a**, 36.8 mg, 0.200 mmol), *p*-xylene (0.800 mL), $Me_3SiOTf$ (7.20 μL, 40.0 μmol) and $B(C_6F_5)_3$ (10.2 mg, 20.0 μmol) at 150 °C for 2 h. The residue was purified by a sequence of flash column chromatography on silica gel with MeOH / methyl *tert*-butyl ether = 1 : 10 as the eluent and Kugelrohr distillation (150 °C, 2 mbar) to afford th e 4-silapiperidine product as a colorless viscous liquid (16.9 mg, 60% yield). **$^1$H NMR** (500 MHz, $CDCl_3$): δ 7.56 – 7.54 (m, 4H), 7.42 – 7.35 (m, 6H), 2.93 (t, $J$ = 6.1 Hz, 4H), 2.64 (q, $J$ = 7.2 Hz, 2H), 1.47 (t, $J$ = 6.1 Hz, 4H), 1.14 (t, $J$ = 7.2 Hz, 3H) ppm. **$^{13}$C NMR** (125 MHz, $CDCl_3$): δ 135.0, 134.9, 129.7, 128.2, 52.0, 51.9, 11.7, 10.8 ppm. **$^1$H/$^{29}$Si HMQC NMR** (500/99 MHz, $CDCl_3$, 298 K, optimized for $J$ = 7 Hz): δ 7.56 – 7.54/–15.6, 2.93/–15.6, 1.47/–15.6 ppm.

**HRMS** (APCI): Calculated for $C_{18}H_{24}NSi^+$ $[M+H]^+$: 282.1678; Found: 282.1669. **IR** (ATR): ṽ 3066, 2923, 2799, 1427, 1229, 1111, 984, 867, 707.

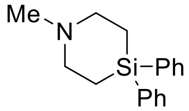

**1-Methyl-4,4-diphenyl-1,4-azasilinane (3qa)**. The general procedure was followed with *N*-ethyl-*N*-methylethanamine (**1q**, 8.70 mg, 0.100 mmol), $Ph_2SiH_2$ (**2a**, 36.8 mg, 0.200 mmol), *p*-xylene (0.800 mL), $Me_3SiOTf$ (7.20 µL, 40.0 µmol) and $B(C_6F_5)_3$ (10.2 mg, 20.0 µmol) at 150 °C for 2 h. The residue was purified by a sequence of flash column chromatography on silica gel with MeOH / methyl *tert*-butyl ether = 1 : 10 as the eluent and Kugelrohr distillation (150 °C, 2 mbar) to afford the 4-silapiperidine product as a colorless viscous liquid (12.9 mg, 48% yield). **$^1$H NMR** (500 MHz, $CDCl_3$): δ 7.56 – 7.53 (m, 4H), 7.42 – 7.35 (m, 6H), 2.77 (t, *J* = 6.2 Hz, 4H), 2.34 (s, 3H), 1.42 (t, *J* = 6.2 Hz, 4H) ppm. **$^{13}$C NMR** (125 MHz, $CDCl_3$): δ 135.3, 134.9, 129.6, 128.1, 54.9, 47.2, 11.7 ppm. **$^1$H/$^{29}$Si HMQC NMR** (500/99 MHz, $CDCl_3$, 298 K, optimized for *J* = 7 Hz): δ 7.56 – 7.53/–16.5, 2.77/–16.5, 1.42/–16.5 ppm. **HRMS** (APCI): Calculated for $C_{17}H_{22}NSi^+$ $[M+H]^+$: 268.1522; Found: 268.1514. **IR** (ATR): ṽ 3066, 2924, 2781, 1464, 1374, 1246, 1181, 1112, 968, 729, 707.

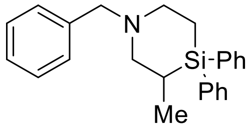

**1-Benzyl-3-methyl-4,4-diphenyl-1,4-azasilinane (3ra).** The general procedure was followed with *N*-benzyl-*N*-ethylpropan-1-amine (**1r**, 17.7 mg, 0.100 mmol), $Ph_2SiH_2$ (**2a**, 36.8 mg, 0.200 mmol), *p*-xylene (0.800 mL), $Me_3SiOTf$ (7.20 µL, 40.0 µmol) and $B(C_6F_5)_3$ (10.2 mg, 20.0 µmol) at 150 °C for 2 h. The residue was purified by a sequence of flash column chromatography on silica gel with methyl *tert*-butyl ether / cyclohexane = 1 : 10 as the eluent and Kugelrohr distillation (220 °C, 2 mbar) to afford the 4-silapiperidine product as a colorless viscous liquid (16.5 mg, 46% yield). **$^1$H NMR** (500 MHz, $CDCl_3$): δ 7.62 – 7.57 (m, 4H), 7.44 – 7.34 (m, 10H), 7.29 – 7.26 (m, 1H), 3.63 (q, *J* = 15.7 Hz, 2H), 3.04 – 2.99 (m, 1H), 2.92 – 2.88 (m, 1H), 2.86 – 2.81 (m, 1H), 2.52 – 2.47 (m, 1H), 1.72 – 1.68 (m, 1H), 1.49 – 1.44 (m, 1H), 1.37 – 1.31 (m, 1H), 1.11 (dd, *J* = 7.6, 2.3 Hz, 3H) ppm. **$^{13}$C NMR** (125 MHz, $CDCl_3$): δ 139.8, 136.0, 135.9, 135.0, 134.6, 129.5, 129.4, 128.9, 128.3, 128.0, 127.8, 126.9, 63.3, 61.1, 53.2, 17.8, 14.9, 11.1 ppm. **$^1$H/$^{29}$Si HMQC NMR** (500/99 MHz, $CDCl_3$, 298 K, optimized for *J* = 7 Hz):

δ 7.62 – 7.57/–13.7, 3.04 – 2.99/–13.7, 2.92 – 2.88/–13.7, 1.49 – 1.44/–13.7, 1.37 – 1.31/–13.7, 1.11/–13.7 ppm. **HRMS** (APCI): Calculated for $C_{24}H_{28}NSi^+$ [M+H]$^+$: 358.1991; Found: 358.1982. **IR** (ATR): ṽ 2921, 2863, 2794, 2756, 1452, 1426, 1314, 1186, 1107, 993, 960, 886, 695.

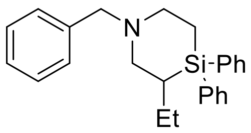


**1-Benzyl-3-ethyl-4,4-diphenyl-1,4-azasilinane (3sa).** The general procedure was followed with *N*-benzyl-*N*-ethylbutan-1-amine (**1s**, 19.1 mg, 0.100 mmol), $Ph_2SiH_2$ (**2a**, 36.8 mg, 0.200 mmol), *p*-xylene (0.800 mL), $Me_3SiOTf$ (7.20 µL, 40.0 µmol) and $B(C_6F_5)_3$ (10.2 mg, 20.0 µmol) at 150 °C for 2 h. The residue was purified by a sequence of flash column chromatography on silica gel with methyl *tert*-butyl ether / cyclohexane = 1 : 10 as the eluent and Kugelrohr distillation (220 °C, 2 mbar) to afford the 4-silapiperidine product as a colorless viscous liquid (14.2 mg, 38% yield). **$^1$H NMR** (500 MHz, $CDCl_3$): δ 7.57 – 7.53 (m, 4H), 7.41 – 7.30 (m, 10H), 7.26 – 7.23 (m, 1H), 3.64 – 3.54 (m, 2H), 2.90 – 2.78 (m, 3H), 2.53 (t, *J* = 8.0 Hz, 1H), 1.60 – 1.53 (m, 1H), 1.44 – 1.40 (m, 3H), 1.28 (s, 1H), 0.79 (t, *J* = 7.0 Hz, 3H) ppm. **$^{13}$C NMR** (125 MHz, $CDCl_3$): δ 139.8, 136.1, 135.8, 135.1, 134.6, 129.4, 129.0, 128.3, 128.0, 127.9, 127.0, 63.4, 57.5, 53.2, 25.7, 22.2, 13.9, 11.4 ppm. **$^1$H/$^{29}$Si HMQC NMR** (500/99 MHz, $CDCl_3$, 298 K, optimized for *J* = 7 Hz): δ 7.57 – 7.53/–14.5, 2.90 – 2.78/–14.5, 2.53/–14.5, 1.60 – 1.53/–14.5, 1.44 – 1.40/–14.5 ppm. **HRMS** (APCI): Calculated for $C_{25}H_{30}NSi^+$ [M+H]$^+$: 372.2148; Found: 372.2137. **IR** (ATR): ṽ 2955, 2922, 2869, 2796, 1453, 1427, 1109, 973, 865, 699.

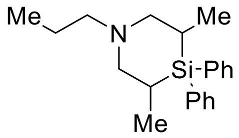


**3,5-Dimethyl-4,4-diphenyl-1-propyl-1,4-azasilinane (3ta, *cis*:*trans* = 58:42).** The general procedure was followed with tripropylamine (**1t**, 14.3 mg, 0.100 mmol), $Ph_2SiH_2$ (**2a**, 36.8 mg, 0.200 mmol), *p*-xylene (0.800 mL), $Me_3SiOTf$ (7.20 µL, 40.0 µmol) and $B(C_6F_5)_3$ (10.2 mg, 20.0 µmol) at 150 °C for 2 h. The residue was purified by a sequence of flash column chromatography on silica gel with MeOH / methyl *tert*-butyl ether = 1 : 30 as the eluent and Kugelrohr distillation (180 °C, 2 mbar) to afford the 4-silapiperidine product as a colorless

viscous liquid (13.3 mg, 41% yield, *cis*:*trans* = 58:42). The stereochemistry of **3ta** was determined by 2D-NOESY. **$^1$H NMR** (500 MHz, $CDCl_3$): δ 7.62 – 7.51 (m, 4H), 7.44 – 7.33 (m, 6H), 3.07 – 2.83 (m, 2H), 2.58 – 2.49 (m, 3H), 2.45 – 2.32 (m, 1H), 1.76 – 1.69 (m, 1H), 1.64 – 1.56 (m, 2H), 1.55 – 1.48 (m, 1H), 1.03 – 0.98 (m, 6H), 0.94 – 0.90 (m, 3H) ppm. **$^{13}$C NMR** (125 MHz, $CDCl_3$): δ 137.0, 136.0, 135.3, 134.9, 134.5, 132.4, 129.6, 129.4, 129.3, 128.0, 127.8, 127.7, 61.9, 61.7, 61.5, 60.7, 20.6, 20.3, 18.3, 15.9, 15.4, 13.9, 12.2, 12.1 ppm. **$^1$H/$^{29}$Si HMQC NMR** (500/99 MHz, $CDCl_3$, 298 K, optimized for $J$ = 7 Hz): δ 7.62 – 7.51/–10.6, 3.07 – 2.83/–10.6, 2.58 – 2.49/–10.6, 1.76 – 1.69/–10.6, 1.03 – 0.98/–10.6 ppm. **HRMS** (APCI): Calculated for $C_{21}H_{30}NSi^+$ [M+H]$^+$: 324.2148; Found: 324.2137. **IR** (ATR): ṽ 2935, 2863, 2766, 1457, 1425, 1376, 1195, 1108, 997, 885, 696.

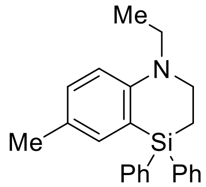


**1-Ethyl-6-methyl-4,4-diphenyl-1,2,3,4-tetrahydrobenzo[*b*][1,4]azasiline (4ua).** The general procedure was followed with *N,N*-diethyl-4-methylaniline (**1u**, 16.3 mg, 0.100 mmol), $Ph_2SiH_2$ (**2a**, 36.8 mg, 0.200 mmol), *p*-xylene (0.800 mL), $Me_3SiOTf$ (7.20 µL, 40.0 µmol) and $B(C_6F_5)_3$ (10.2 mg, 20.0 µmol) at 150 °C for 2 h. The residue was purified by a sequence of flash column chromatography on silica gel with methyl *tert*-butyl ether / cyclohexane = 1 : 100 as the eluent and Kugelrohr distillation (220 °C, 2 mbar) to afford the 4-silapiperidine product as a colorless viscous liquid (19.0 mg, 55% yield). **$^1$H NMR** (500 MHz, $CDCl_3$): δ 7.66 – 7.64 (m, 4H), 7.46 – 7.39 (m, 6H), 7.22 (s, 1H), 7.13 (dd, $J$ = 8.5, 2.2 Hz, 1H), 6.77 (d, $J$ = 8.6 Hz, 1H), 3.53 – 3.46 (m, 4H), 2.24 (s, 3H), 1.53 (t, $J$ = 6.3 Hz, 2H), 1.20 (t, $J$ = 7.1 Hz, 3H) ppm. **$^{13}$C NMR** (125 MHz, $CDCl_3$): δ 153.7, 137.7, 136.2, 135.7, 131.8, 129.5, 127.9, 125.3, 116.5, 112.9, 48.7, 47.4, 20.4, 12.6, 11.4 ppm. **$^1$H/$^{29}$Si HMQC NMR** (500/99 MHz, $CDCl_3$, 298 K, optimized for $J$ = 7 Hz): δ 7.66 – 7.64/–20.7, 7.22/–20.7, 6.77/–20.7, 3.53 – 3.46/–20.7, 1.53/–20.7 ppm. **HRMS** (APCI): Calculated for $C_{23}H_{26}NSi^+$ [M+H]$^+$: 344.1835; Found: 344.1828. **IR** (ATR): ṽ 3065, 2969, 2926, 1604, 1487, 1426, 1332, 1277, 1108, 1068, 802, 699.

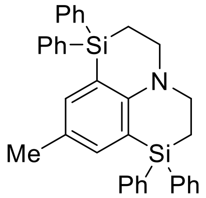

**8-Methyl-1,1,6,6-tetraphenyl-2,3,5,6-tetrahydro-1*H*,4*H*-3a-aza-1,6-disilaphenalene (5ua).** The following residue was further purified by Kugelrohr distillation (250 °C, 2 mbar) to afford the 4-silapiperidine product as a colorless viscous liquid (4.30 mg, 8% yield). **$^{1}$H NMR** (500 MHz, $CDCl_3$): δ 7.61 – 7.59 (m, 8H), 7.39 – 7.34 (m, 12H), 7.22 (s, 2H), 3.49 – 3.45 (m, 4H), 2.12 (s, 3H), 1.51 – 1.49 (m, 4H). **$^{13}$C NMR** (125 MHz, $CDCl_3$): δ162.2, 139.6, 136.3, 135.7, 129.5, 128.0, 125.7, 117.3, 52.8, 20.5, 12.6 ppm. **$^{1}$H/$^{29}$Si HMQC NMR** (500/99 MHz, $CDCl_3$, 298 K, optimized for *J* = 7 Hz): δ 7.61 – 7.59/–19.5, 7.39 – 7.34/–19.5, 7.22/–19.5, 3.49 – 3.45/–19.5, 1.51 – 1.49/–19.5 ppm. **HRMS** (APCI): Calculated for $C_{35}H_{34}NSi_2^+$ $[M+H]^+$: 524.2230; Found: 524.2215. **IR** (ATR): ṽ 3066, 2934, 2809, 2134, 1536, 1483, 1427, 1262, 1109, 827, 733, 698.

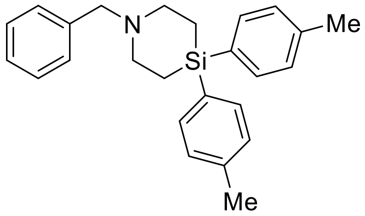

**1-Benzyl-4,4-di-*p*-tolyl-1,4-azasilinane (3ab)**. The general procedure was followed with *N*-benzyl-*N*-ethylethanamine (**1a**, 16.3 mg, 0.100 mmol), di-*p*-tolylsilane (**2b**, 42.5 mg, 0.200 mmol), *p*-xylene (0.800 mL), $Me_3SiOTf$ (7.20 µL, 40.0 µmol) and $B(C_6F_5)_3$ (10.2 mg, 20.0 µmol) at 150 °C for 2 h. The residue was purified by a sequence of flash column chromatography on silica gel with methyl *tert*-butyl ether / cyclohexane = 1 : 10 as the eluent and Kugelrohr distillation (220 °C, 2 mbar) to afford the 4-silapiperidine product as a colorless viscous liquid (24.1 mg, 65% yield). **$^{1}$H NMR** (500 MHz, $CDCl_3$): δ 7.45 (d, *J* = 7.9 Hz, 4H), 7.37 – 7.30 (m, 4H), 7.26 – 7.23 (m, 1H), 7.19 (d, *J* = 7.5 Hz, 4H), 3.60 (s, 2H), 2.83 (t, *J* = 5.8 Hz, 4H), 2.37 (s, 6H), 1.35 (t, *J* = 5.7 Hz, 4H) ppm. **$^{13}$C NMR** (125 MHz, $CDCl_3$): δ 139.5, 139.3, 134.9, 132.4, 129.0, 128.9, 128.3, 127.0, 62.8, 52.5, 21.6, 11.6 ppm. **$^{1}$H/$^{29}$Si HMQC NMR** (500/99 MHz, $CDCl_3$, 298 K, optimized for *J* = 7 Hz): δ 7.45/–16.0, 2.83/–16.0, 1.35/–16.0 ppm. **HRMS** (APCI): Calculated for $C_{25}H_{30}NSi^+$ $[M+H]^+$: 372.2148; Found: 372.2135. **IR** (ATR): ṽ 3027, 2920, 2796, 1601, 1452, 1391, 1188, 1107, 970, 866, 799, 727.

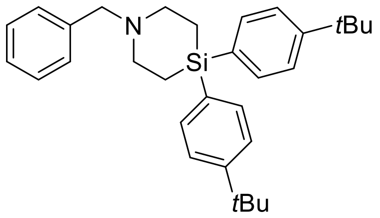

**1-Benzyl-4,4-bis(4-(*tert*-butyl)phenyl)-1,4-azasilinane (3ac)**. The general procedure was followed with *N*-benzyl-*N*-ethylethanamine (**1a**, 16.3 mg, 0.100 mmol), bis(4-(*tert*-butyl)phenyl)silane (**2c**, 59.3 mg, 0.200 mmol), *p*-xylene (0.800 mL), $Me_3SiOTf$ (7.20 µL, 40.0 µmol) and $B(C_6F_5)_3$ (10.2 mg, 20.0 µmol) at 150 °C for 2 h. The residue was purified by a sequence of flash column chromatography on silica gel with methyl *tert*-butyl ether / cyclohexane = 1 : 10 as the eluent and Kugelrohr distillation (240 °C, 2 mbar) to afford the 4-silapiperidine product as a colorless viscous liquid (29.6 mg, 65% yield). **$^1$H NMR** (500 MHz, $CDCl_3$): δ 7.52 (d, *J* = 8.2 Hz, 4H), 7.41 (d, *J* = 8.2 Hz, 4H), 7.37 (d, *J* = 7.4 Hz, 2H), 7.33 (t, *J* = 7.3 Hz, 2H), 7.26 (t, *J* = 7.2 Hz, 1H), 3.62 (s, 2H), 2.85 (t, *J* = 6.2 Hz, 4H), 1.37 (t, *J* = 6.1 Hz, 4H), 1.35 (s, 18H) ppm. **$^{13}$C NMR** (125 MHz, $CDCl_3$): δ 152.3, 139.8, 134.7, 132.6, 128.9, 128.3, 126.9, 125.0, 62.7, 52.5, 34.8, 31.4, 11.6 ppm. **$^1$H/$^{29}$Si HMQC NMR** (500/99 MHz, $CDCl_3$, 298 K, optimized for *J* = 7 Hz): δ 7.52/–16.8, 2.85/–16.8, 1.37/–16.8 ppm. **HRMS** (APCI): Calculated for $C_{31}H_{42}NSi^+$ $[M+H]^+$: 456.3087; Found: 456.3071. **IR** (ATR): ṽ 3066, 2959, 2797, 1598, 1458, 1387, 1267, 1136, 1086, 970, 866, 820, 724.

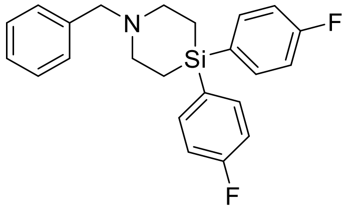

**1-Benzyl-4,4-bis(4-fluorophenyl)-1,4-azasilinane (3ad)**. The general procedure was followed with *N*-benzyl-*N*-ethylethanamine (**1a**, 16.3 mg, 0.100 mmol), bis(4-fluorophenyl)silane (**2d**, 44.1 mg, 0.200 mmol), *p*-xylene (0.800 mL), $Me_3SiOTf$ (7.20 µL, 40.0 µmol) and $B(C_6F_5)_3$ (10.2 mg, 20.0 µmol) at 150 °C for 2 h. The residue was purified by a sequence of flash column chromatography on silica gel with methyl *tert*-butyl ether / cyclohexane = 1 : 10 as the eluent and Kugelrohr distillation (220 °C, 2 mbar) to afford the 4-silapiperidine product as a colorless viscous liquid (25.5 mg, 67% yield). **$^1$H NMR** (500 MHz, $CDCl_3$): δ 7.52 – 7.48 (m, 4H), 7.36 – 7.30 (m, 4H), 7.27 – 7.24 (m, 1H), 7.10 – 7.05 (m, 4H),

3.59 (s, 2H), 2.81 (t, *J* = 6.3 Hz, 4H), 1.34 (t, *J* = 6.1 Hz, 4H) ppm. **$^{13}$C NMR** (125 MHz, $CDCl_3$): δ 164.1 (d, *J* = 248.8 Hz), 139.4, 136.7 (d, *J* = 7.3 Hz), 131.2, 128.9, 128.3, 127.1, 115.4 (d, *J* = 19.8 Hz), 62.9, 52.3, 11.8 ppm. **$^{19}$F NMR** (471 MHz, $CDCl_3$): δ –111.1 ppm. **$^{1}$H/$^{29}$Si HMQC NMR** (500/99 MHz, $CDCl_3$, 298 K, optimized for *J* = 7 Hz): δ 7.52 – 7.48/–15.0, 2.81/–15.0,1.34/–15.0 ppm. **HRMS** (APCI): Calculated for $C_{23}H_{24}F_2NSi^+$ $[M+H]^+$: 380.1646; Found: 380.1635. **IR** (ATR): ṽ 3026, 2922, 2797, 1585, 1497, 1388, 1230, 1161, 1105, 971, 867, 821, 728.

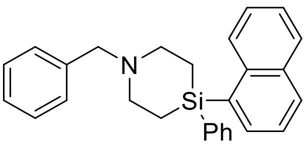


**1-Benzyl-4-(naphthalen-1-yl)-4-phenyl-1,4-azasilinane (3ae)**. The general procedure was followed with *N*-benzyl-*N*-ethylethanamine (**1a**, 16.3 mg, 0.100 mmol), naphthalen-1-yl(phenyl)silane (**2e**, 46.9 mg, 0.200 mmol), *p*-xylene (0.800 mL), $Me_3SiOTf$ (7.20 µL, 40.0 µmol) and $B(C_6F_5)_3$ (10.2 mg, 20.0 µmol) at 150 °C for 2 h. The residue was purified by a sequence of flash column chromatography on silica gel with methyl *tert*-butyl ether / cyclohexane = 1 : 10 as the eluent and Kugelrohr distillation (230 °C, 2 mbar) to afford the 4-silapiperidine product as a colorless viscous liquid (26.8 mg, 68% yield). **$^{1}$H NMR** (500 MHz, $CDCl_3$): δ 7.97 (d, *J* = 8.4 Hz, 1H), 7.93 (d, *J* = 8.2 Hz, 1H), 7.88 (d, *J* = 8.2 Hz, 1H), 7.81 (dd, *J* = 6.8, 1.2 Hz, 1H), 7.60 (dd, *J* = 7.7, 1.5 Hz, 2H), 7.53 (dd, *J* = 8.2, 6.9 Hz, 1H), 7.47 – 7.44 (m, 1H), 7.39 – 7.31 (m, 8H), 7.27 – 7.24 (m, 1H), 3.63 (s, 2H), 2.95 – 2.85 (m, 4H), 1.65 – 1.54 (m, 4H) ppm. **$^{13}$C NMR** (125 MHz, $CDCl_3$): δ 139.6, 137.3, 136.9, 135.2, 134.8, 133.7, 133.5, 130.5, 129.4, 129.1, 128.9, 128.6, 128.3, 128.1, 127.0, 125.9, 125.6, 125.2, 62.9, 52.6, 12.6 ppm. **$^{1}$H/$^{29}$Si HMQC NMR** (500/99 MHz, $CDCl_3$, 298 K, optimized for *J* = 7 Hz): δ 7.81/–15.8, 7.60/–15.8, 2.95 – 2.85/–15.8, 1.65 – 1.54/–15.8, ppm. **HRMS** (APCI): Calculated for $C_{27}H_{28}NSi^+$ $[M+H]^+$: 394.1991; Found: 394.1978. **IR** (ATR): ṽ 2923, 2895, 2796, 2759, 1426, 1389, 1317, 1226, 1106, 969, 905, 867, 795, 776, 723, 696.

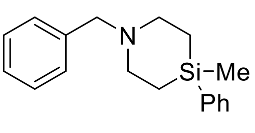


**1-Benzyl-4-methyl-4-phenyl-1,4-azasilinane (3ag)**. The general procedure was followed with *N*-benzyl-*N*-ethylethanamine (**1a**, 16.3 mg, 0.100 mmol), methyl(phenyl)silane (**2g**, 24.4 mg, 0.200 mmol), *p*-xylene (0.800 mL), $Me_3SiOTf$ (7.20 µL, 40.0 µmol) and $B(C_6F_5)_3$ (10.2 mg,

20.0 µmol) at 150 °C for 2 h. The residue was purified by a sequence of flash column chromatography on silica gel with methyl *tert*-butyl ether / cyclohexane = 1 : 10 as the eluent and Kugelrohr distillation (180 °C, 2 mbar) to afford the 4-silapiperidine product as a colorless viscous liquid (11.3 mg, 40% yield). **$^{1}$H NMR** (500 MHz, $CDCl_3$): δ 7.57 – 7.56 (m, 2H), 7.38 – 7.31 (m, 7H), 7.27 – 7.24 (m, 1H), 3.62 (s, 2H), 2.87 – 2.82 (m, 2H), 2.79 – 2.74 (m, 2H), 1.21 – 1.16 (m, 2H), 0.99 – 0.96 (m, 2H), 0.32 (s, 3H) ppm. **$^{13}$C NMR** (125 MHz, $CDCl_3$): δ 139.1, 138.1, 134.0, 129.3, 129.1, 128.3, 128.0, 127.0, 62.8, 52.5, 12.6, –4.0 ppm. **$^{1}$H/$^{29}$Si HMQC NMR** (500/99 MHz, $CDCl_3$, 298 K, optimized for $J$ = 7 Hz): δ 7.57 – 7.56/–11.5, 2.87 – 2.82/–11.5, 1.21 – 1.16/–11.5, 0.99 – 0.96/–11.5, 0.32/–11.5, ppm. **HRMS** (APCI): Calculated for $C_{18}H_{24}NSi^+$ $[M+H]^+$: 282.1678; Found: 282.1673. **IR** (ATR): ṽ 2919, 2794, 2756, 1391, 1250, 1110, 972, 868, 788, 730, 697.

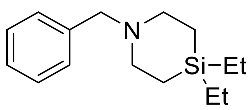


**1-Benzyl-4,4-diethyl-1,4-azasilinane (3ai)**. The general procedure was followed with *N*-benzyl-*N*-ethylethanamine (**1a**, 16.3 mg, 0.100 mmol), diethylsilane (**2i**, 17.6 mg, 0.200 mmol), *p*-xylene (0.800 mL), $Me_3SiOTf$ (7.20 µL, 40.0 µmol) and $B(C_6F_5)_3$ (10.2 mg, 20.0 µmol) at 150 °C for 2 h. The residue was purified by a sequence of flash column chromatography on silica gel with methyl *tert*-butyl ether / cyclohexane = 1 : 10 as the eluent and Kugelrohr distillation (140 °C, 2 mbar) to afford the 4-silapiperidine product as a colorless viscous liquid (10.5 mg, 42% yield). **$^{1}$H NMR** (500 MHz, $CDCl_3$): δ 7.35 – 7.30 (m, 4H), 7.26 – 7.23 (m, 1H), 3.60 (s, 2H), 2.73 (t, $J$ = 6.3 Hz, 4H), 0.96 (t, $J$ = 8.0 Hz, 6H), 0.79 (t, $J$ = 6.4 Hz, 4H), 0.57 (t, $J$ = 8.0 Hz, 4H) ppm. **$^{13}$C NMR** (125 MHz, $CDCl_3$): δ 138.9, 129.2, 128.3, 127.1, 62.7, 52.6, 9.9, 7.4, 3.7 ppm. **$^{1}$H/$^{29}$Si HMQC NMR** (500/99 MHz, $CDCl_3$, 298 K, optimized for $J$ = 7 Hz): δ 2.73/–3.1, 0.96/–3.1, 0.79/–3.1, 0.57/–3.1, ppm. **HRMS** (APCI): Calculated for $C_{15}H_{26}NSi^+$ $[M+H]^+$: 248.1835; Found: 248.1831. **IR** (ATR): ṽ 2950, 2910, 2794, 1465, 1391, 1230, 1010, 976, 865, 730, 697.

## 3.3 Unsuccessful Substrates

Further investigation of the substrate scope revealed that tertiary benzylamines having two isopropyl, cyclohexyl, isobutyl, or phenethyl groups as well as 1-benzylazepane failed to furnish the corresponding 4-silapiperidine derivative.

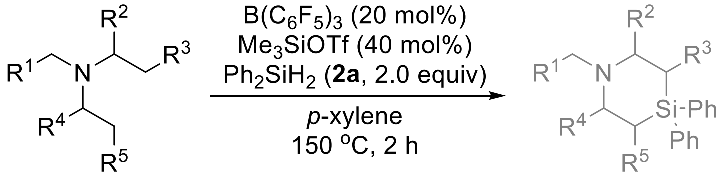


**not detected**

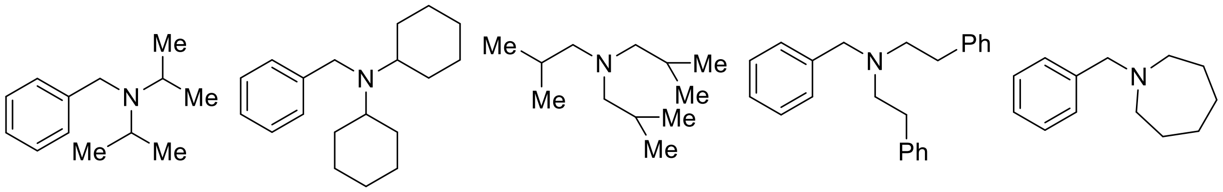

## 4 Scale-Up Experiment

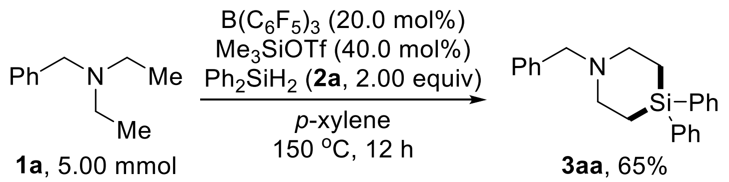


In a nitrogen-filled glovebox, a 50-mL single-neck round bottom flask equipped with a magnetic stir bar was charged with *N*-benzyl-*N*-ethylethanamine (**1a**, 0.820 g, 5.00 mmol), $Ph_2SiH_2$ (**2a**, 1.84 g, 10.0 mmol), *p*-xylene (20.0 mL), $Me_3SiOTf$ (362 μL, 2.00 mmol) and $B(C_6F_5)_3$ (512 mg, 1.00 mmol). The single-neck round bottom flask was fitted with a cap, and the reaction was then removed from the glovebox. After a condenser was attached to the flask, the mixture was stirred at 150 °C for 12 h in a preheated oil bath with a continous flow of nitrogen gas. After the resulting reaction mixture was cooled to room temperature, NaOH (20.0 mL, 10% aq.) was added and the mixture stirred for 30 min. The mixture was extracted with methyl *tert*-butyl ether (20 mL×3). The combined organic layers were washed with brine (15 mL), dried over $Na_2SO_4$, and the solvent was removed under reduced pressure. The residue was purified by a sequence of flash column chromatography on silica gel with methyl *tert*-butyl ether : cyclohexane = 1 : 10 as the eluent and Kugelrohr distillation (200 °C, 2 mbar) to afford **3aa** as a colorless viscous liquid (1.12 g, 65% yield).

# 5 Elaboration of an *N*-Benzylated 4-Silapiperidine

## 5.1 Debenzylation of Product 3aa[11]

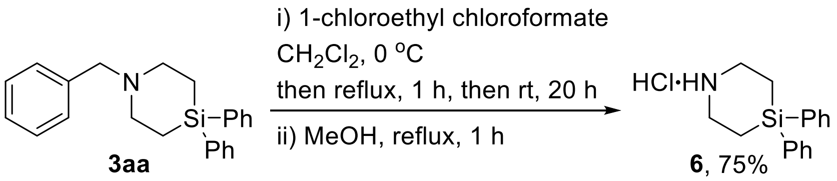


A flame-dried 50-mL two-neck flask equipped with a magnetic stir bar and a reflux condenser was charged with **3aa** (0.340 g, 1.00 mmol) and $CH_2Cl_2$ (15 mL) under an atmosphere of nitrogen. Then, 1-chloroethyl chloroformate (0.135 mL, 1.25 mmol) was added dropwise at 0 °C within 5 min. The mixture was heated at reflux for 1 h and was then stirred at room temperature for a further 20 h. After that, the solvent was removed under reduced pressure. The resluting oily residue was dissolved in MeOH (10 mL), and the mixture was heated at reflux for 1 h. After the reaction mixture was cooled to room temperature, the solvent was removed under reduced pressure to give viscous oil which was triturated with diethyl ether to obtain the product **6** as a white solid (217 mg, 75% yield). **$^1$H NMR** (500 MHz, $CDCl_3$): δ 9.63 (br, 2H), 7.56 – 7.55 (m, 4H), 7.46 – 7.38 (m, 6H), 3.44 (t, *J* = 6.2 Hz, 4H), 1.74 (t, *J* = 6.3 Hz, 4H) ppm. **$^{13}$C NMR** (125 MHz, $CDCl_3$): δ 134.8, 132.0, 130.5, 128.6, 44.3, 9.1 ppm. **$^1$H/$^{29}$Si HMQC NMR** (500/99 MHz, $CDCl_3$, 298 K, optimized for *J* = 7 Hz): δ 7.56 – 7.55/–16.5, 3.44/–16.5, 1.74/–16.5 ppm. Spectral data is in agreement with published data.[12]

## 5.2 Oxidation of Product 3aa[13]

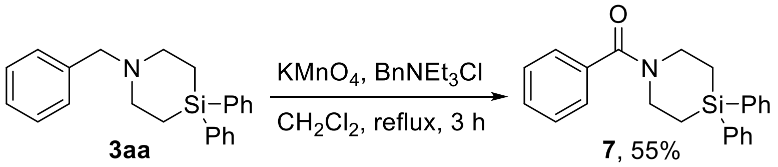


A flame-dried 50-mL two-neck flask equipped with a magnetic stir bar and a reflux condenser was charged with **3aa** (0.340 g, 1.00 mmol) and $CH_2Cl_2$ (20 mL) under a atmosphere of nitrogen. Then benzyltriethylammoniumchlorid (0.680 g, 3.00 mmol) and $KMnO_4$ (0.470 g, 3.00 mmol) was added, and the reaction mixture was heated at reflux for 3 h. After the reaction mixture was cooled to room temperature, the suspension was quenched by aq. sodium thiosulphate and filtered. The resulting solution was extracted with $CH_2Cl_2$ (20 mL×3),

and the combined organic layers were washed with brine (15 mL), dried over $Na_2SO_4$, and the solvent was removed under reduced pressure. The product was purified by flash column chromatography on silica gel with methyl *tert*-butyl ether : cyclohexane = 1 : 3 as the eluent to afford **7** as a white solid (197 mg, 55% yield). **$^1$H NMR** (500 MHz, $CDCl_3$): δ 7.56 – 7.54 (m, 4H), 7.45 – 7.38 (m, 11H), 4.00 (br, 2H), 3.64 (br, 2H), 1.56 (br, 2H), 1.27 (br, 2H) ppm. **$^{13}$C NMR** (125 MHz, $CDCl_3$): δ 171.1, 136.9, 134.6, 134.5, 130.0, 129.4, 128.5, 128.3, 126.5, 47.1, 42.0, 13.0, 11.3 ppm. **$^1$H/$^{29}$Si HMQC NMR** (500/99 MHz, $CDCl_3$, 298 K, optimized for $J$ = 7 Hz): δ 7.56 – 7.54/–14.2 ppm. **HRMS** (APCI): Calculated for $C_{23}H_{24}NOSi^+$ $[M+H]^+$: 358.1627; Found: 358.1618. **IR** (ATR): ṽ 2927, 2894, 1626, 1426, 1296, 1112, 969, 831, 731, 701.

# 6 Mechanistic Investigations

## 6.1 $^2$H-Labeling Experiments

### 6.1.1 Reaction of **1a** with $Ph_2SiD_2$ (**2a**-$d_2$) Under Standard Conditions

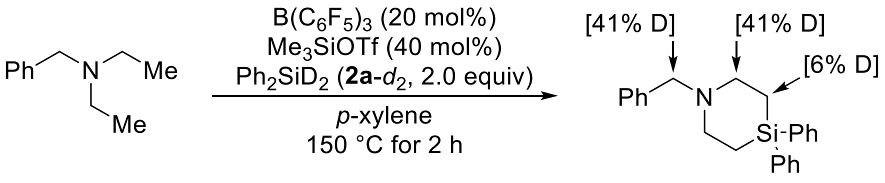


**1a** **3aa**-$d_3$: 70%

In a nitrogen-filled glovebox, a 10-mL sealed tube equipped with a magnetic stir bar was charged with *N*-benzyl-*N*-ethylethanamine (**1a**, 16.3 mg, 0.100 mmol), $Ph_2SiH_2$ (**2a**-$d_2$, 37.2 mg, 0.200 mmol), *p*-xylene (0.800 mL), $Me_3SiOTf$ (7.20 µL, 40.0 µmol), and $B(C_6F_5)_3$ (10.2 mg, 20.0 µmol). The sealed tube was fitted with a cap, and the reaction stirred at 150 $^o$C for 2 h in a preheated oil bath. After the resulting reaction mixture was cooled to room temperature, NaOH (5.00 mL, 10% aq.) was added and the mixture stirred for 30 min. The mixture was extracted with methyl *tert*-butyl ether (20 mL×3). The combined organic layers were washed with brine (15 mL), dried over $Na_2SO_4$, and the solvent was removed under reduced pressure. The residue was purified by a sequence of flash column chromatography on silica gel with methyl *tert*-butyl ether : cyclohexane = 1 : 10 as the eluent and Kugelrohr distillation (200 $^o$C, 2 mbar) to afford **3aa**-$d_3$ as a colorless viscous liquid (24.2 mg, 70% yield). Deuterium incorporation: 2.70 D/molecule ($^1$H NMR). **$^1$H NMR** (500 MHz, $CDCl_3$): δ 7.57 – 7.56 (m, 4H), 7.42 – 7.31 (m, 10H), 7.27 – 7.24 (m, 1H), 3.60 (d, *J* = 13.8 Hz, 1.18H, 41%D), 2.89 – 2.78 (m, 2.36H, 41%D), 1.40 – 1.35 (m, 3.74H, 6%D) ppm. **$^{13}$C NMR** (125 MHz, $CDCl_3$): δ 139.4, 135.9, 134.8, 129.5, 129.0, 128.3, 128.1, 127.0, 62.7 –62.2 (m), 52.4 – 51.8 (m), 11.2 (t, *J* = 16.1 Hz) ppm. **$^1$H/$^{29}$Si HMQC NMR** (500/99 MHz, $CDCl_3$, 298 K, optimized for *J* = 7 Hz): δ 7.57 – 7.56/–15.8, 2.89 – 2.78/–15.8, 1.40 – 1.35/–15.8 ppm. **HRMS** (APCI): Calculated for $C_{23}H_{23}D_3NSi^+$ $[M+H]^+$: 347.2023; Found: 347.2009. **IR** (ATR): ṽ 3064, 3021, 2919. 2786, 1491, 1427, 1214, 1110, 962, 874, 698.

**Table S1.** Ratio of different deuterated products **3aa**-$d_n$ (n = 0~6) determined by mass spectroscopy.

| **3aa**-$d_n$ | Compared to each other in %(intensity : sum of all intensity * 100%) |
|---|---|
| **3aa** | 2 |
| **3aa**-$d_1$ | 10 |
| **3aa**-$d_2$ | 22 |
| **3aa**-$d_3$ | 28 |
| **3aa**-$d_4$ | 22 |
| **3aa**-$d_5$ | 11 |
| **3aa**-$d_6$ | 1 |

The overall deuteration grades of **3aa**-$d_3$ determined by mass spectrometry is 2.87 D.

6.1.2 Reaction of **1v** with $Ph_2SiD_2$ (**2a**-$d_2$) Under Standard Conditions

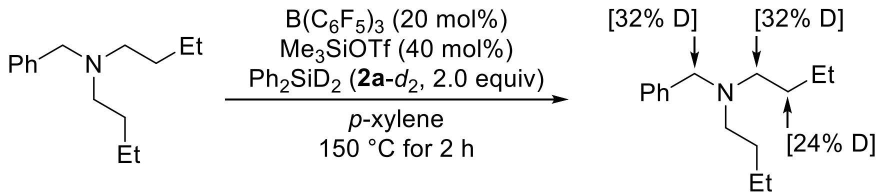


**1v** **1v**-$d_3$: 75%

In a nitrogen-filled glovebox, a 10-mL sealed tube equipped with a magnetic stir bar was charged with *N*-benzyl-*N*-butylbutan-1-amine (**1v**, 21.9 mg, 0.100 mmol), $Ph_2SiH_2$ (**2a**-$d_2$, 37.2 mg, 0.200 mmol), *p*-xylene (0.800 mL), $Me_3SiOTf$ (7.20 μL, 40.0 μmol) and $B(C_6F_5)_3$ (10.2 mg, 20.0 μmol). The sealed tube was fitted with a cap, and the reaction stirred at 150 °C for 2 h in a preheated oil bath. After the resulting reaction mixture was cooled to room temperature, NaOH (5.00 mL, 10% aq.) was added and the mixture stirred for 30 min. The mixture was extracted with methyl *tert*-butyl ether (20 mL×3). The combined organic layers were washed with brine (15 mL), dried over $Na_2SO_4$, and the solvent was removed under reduced pressure. The residue was purified by a sequence of flash column chromatography on silica gel with methyl *tert*-butyl ether : cyclohexane = 1 : 5 as the eluent to afford **1v**-$d_3$ as a colorless liquid (16.8 mg, 75% yield). Deuterium incorporation: 2.85 D/molecule ($^1$H NMR). **$^1$H NMR** (500 MHz, $CDCl_3$): δ 7.33 – 7.28 (m, 4H), 7.22 (t, *J* = 7.0 Hz, 1H), 3.58 – 3.50 (m, 1.37H, 32%D), 2.42 – 2.38 (m, 2.71H, 32%D), 1.48 – 1.42 (m, 4H), 1.33 – 1.26 (m, 3.06H, 24%D), 0.88 (t, *J* = 7.4 Hz, 6H) ppm. **$^{13}$C NMR** (125 MHz, $CDCl_3$): δ 140.2, 129.0, 128.2, 126.8, 58.7 –58.2 (m), 53.6 – 53.1 (m), 29.3 – 29.0 (m), 20.7 – 20.2 (m), 14.2 – 14.0 (m) ppm. **HRMS** (APCI): Calculated for

$C_{15}H_{23}D_3N^+$ $[M+H]^+$: 223.2254; Found: 223.2244. **IR** (ATR): ṽ 2955, 2928, 2869, 2795, 1453, 1376, 1170, 731, 697.

**Table S2.** Ratio of different deuterated products **1v**-$d_n$ (n = 0~7) determined by mass spectroscopy.

| **1v**-$d_n$ | Compared to each other in %(intensity : sum of all intensity * 100%) |
|---|---|
| **1v** | 3 |
| **1v**-$d_1$ | 12 |
| **1v**-$d_2$ | 23 |
| **1v**-$d_3$ | 27 |
| **1v**-$d_4$ | 21 |
| **1v**-$d_5$ | 10 |
| **1v**-$d_6$ | 3 |
| **1v**-$d_7$ | 1 |

The overall deuteration grades of **1v**-$d_3$ determined by mass spectrometry is 2.98 D.

## 6.2 Stoichiometric Experiments

### 6.2.1 Stoichiometric Reaction of **1a** and $B(C_6F_5)_3$ (1:1)

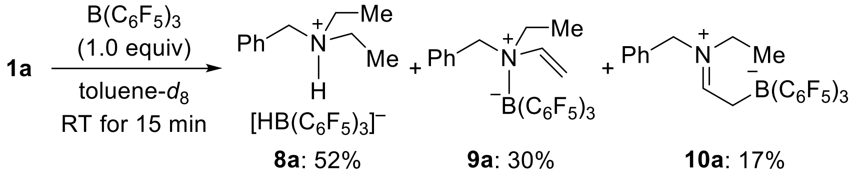


In a nitrogen-filled glovebox, a J-Young tube was charged with **1a** (8.20 mg, 0.0500 mmol), mesitylene (3.50 μL, 0.0250 mmol), toluene-*d*$_8$ (0.5 mL), and $B(C_6F_5)_3$ (25.6 mg, 0.0500 mmol). After shaking 15 min at RT, the reaction mixture was analyzed by NMR spectroscopy (internal standard material: mesitylene, 3.50 μL, 0.0250 mmol). *Crude NMR data of **8a***: **$^{11}$B NMR** (161 MHz, toluene-*d*$_8$; selected data of **8a**): δ –23.8 (d, *J* = 81.2 Hz) ppm; **$^{19}$F NMR** (471 MHz, toluene-*d*$_8$; selected data of **8a**): δ –133.8 (d, *J* = 30.0 Hz), –161.2 (t, *J* = 20.8 Hz), –165.4 – –165.5 (m) ppm. *Crude NMR data of **9a***: **$^{11}$B NMR** (161 MHz, toluene-*d*$_8$; selected data of **9a**): δ –13.9 ppm; **$^{19}$F NMR** (471 MHz, toluene-*d*$_8$; selected data of **9a**): δ –132.5 (d, *J* = 28.0 Hz), –159.4 (t, *J* = 21.4 Hz), –164.2 – –164.4 (m) ppm. *Crude NMR data of **10a***: **$^{11}$B NMR** (161 MHz, toluene-*d*$_8$; selected data of **10a**): δ –13.5 ppm; **$^{19}$F NMR** (471 MHz, toluene-*d*$_8$; selected data of **10a**): δ –132.1 (d, *J* = 28.6 Hz), –159.2 (t, *J* = 21.4 Hz), –164.1 – –164.2 (m) ppm.

### 6.2.2 Stoichiometric Reaction of **1a**, $Me_3SiOTf$, and $B(C_6F_5)_3$ (1:1:1)

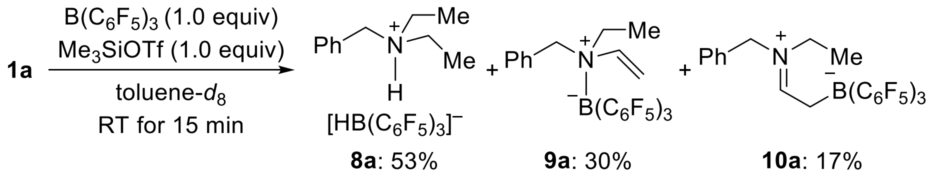


In a nitrogen-filled glovebox, a J-Young tube was charged with **1a** (8.20 mg, 0.0500 mmol), mesitylene (3.50 μL, 0.0250 mmol), toluene-*d*$_8$ (0.5 mL), and $B(C_6F_5)_3$ (25.6 mg, 0.0500 mmol). After shaking 15 min at RT, the reaction mixture was analyzed by NMR spectroscopy (internal standard material: mesitylene, 3.50 μL, 0.0250 mmol).

# 7 NMR Spectra

**Figure S1.** $^{1}H$ NMR spectrum (500 MHz, $CDCl_3$, 298 K) of ***N*-benzyl-*N*-ethylpropan-1-amine (1r)**.

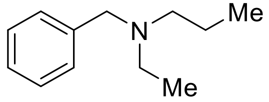


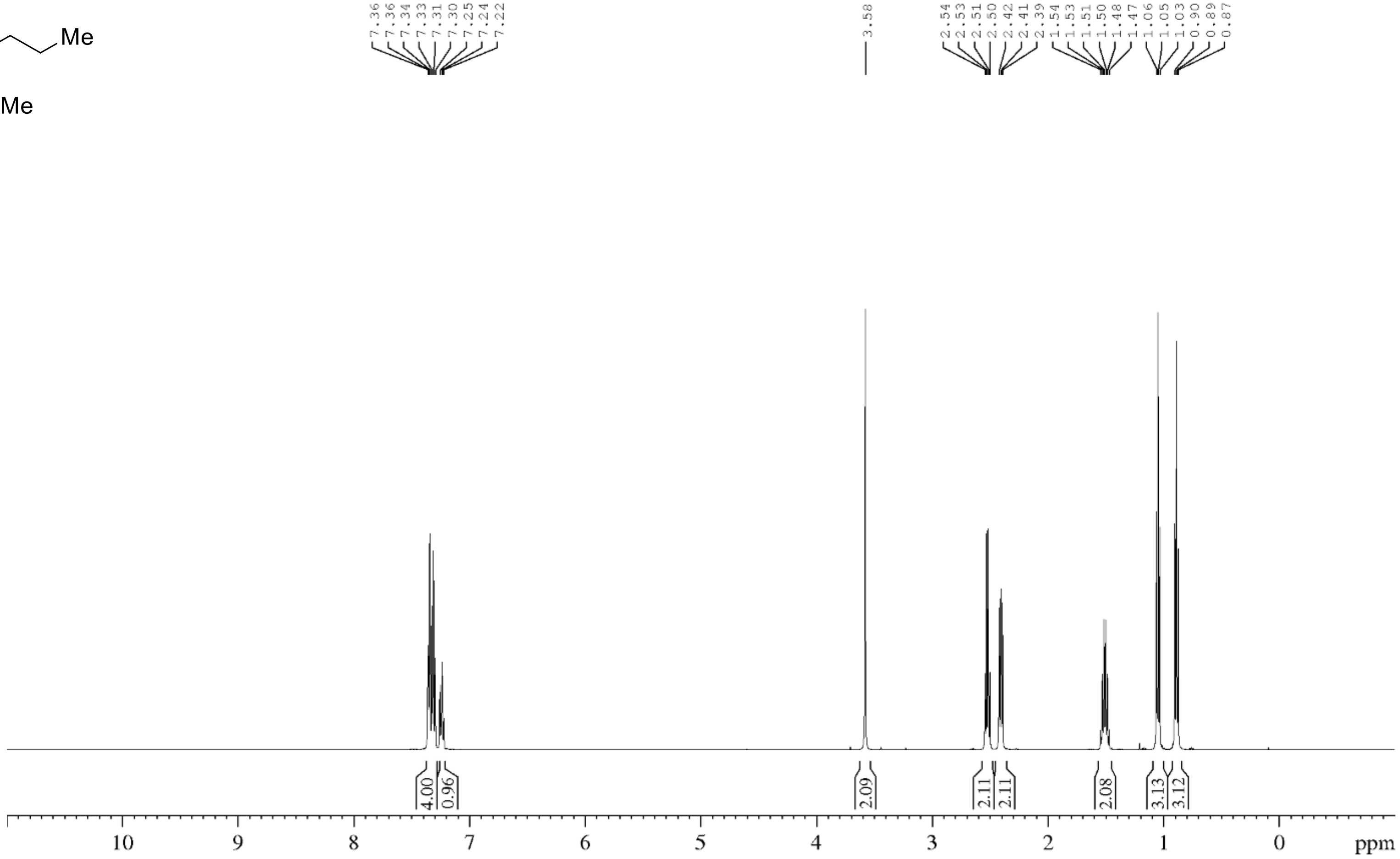

**Figure S2.** $^{13}$C NMR spectrum (125 MHz, $CDCl_3$, 298 K) of ***N*-benzyl-*N*-ethylpropan-1-amine (1r)**.

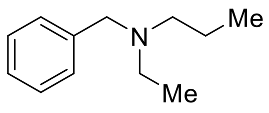


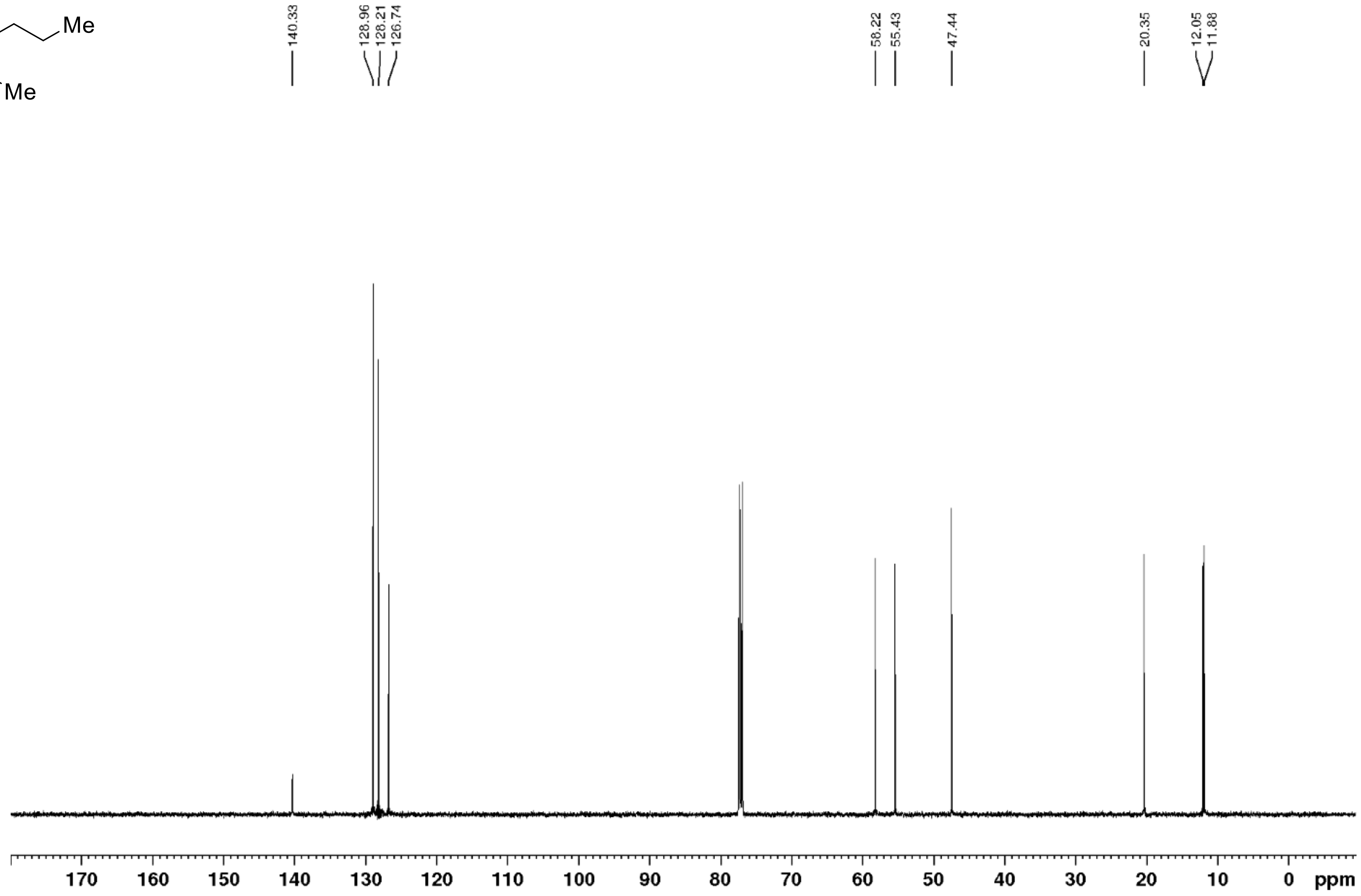

**Figure S3.** $^{1}$H NMR spectrum (500 MHz, $CDCl_3$, 298 K) of ***N*-benzyl-*N*-ethylbutan-1-amine (1s)**.

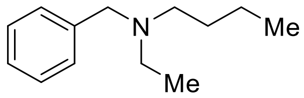


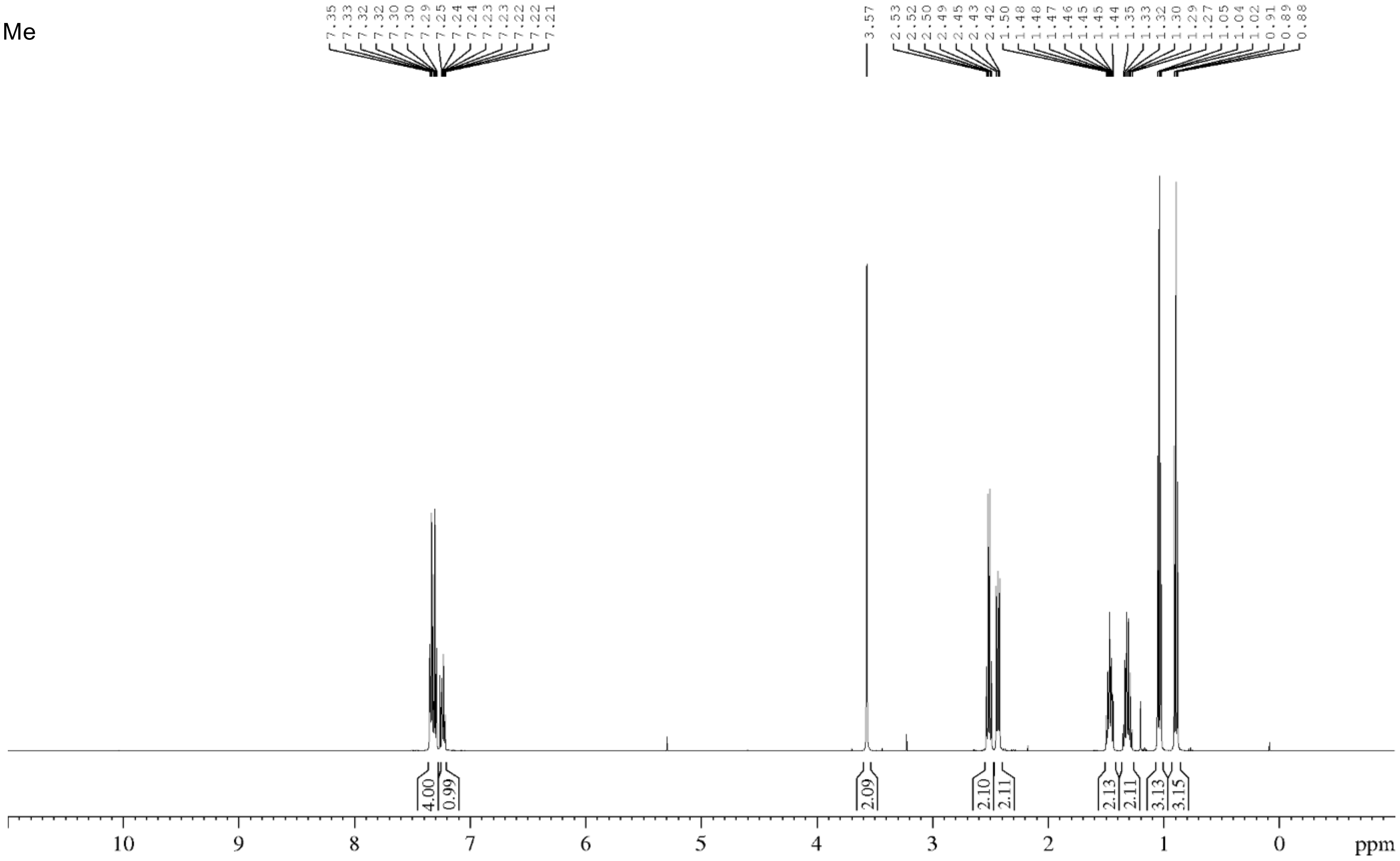

**Figure S4.** $^{13}C$ NMR spectrum (125 MHz, $CDCl_3$, 298 K) of ***N*-benzyl-*N*-ethylbutan-1-amine (1s)**.

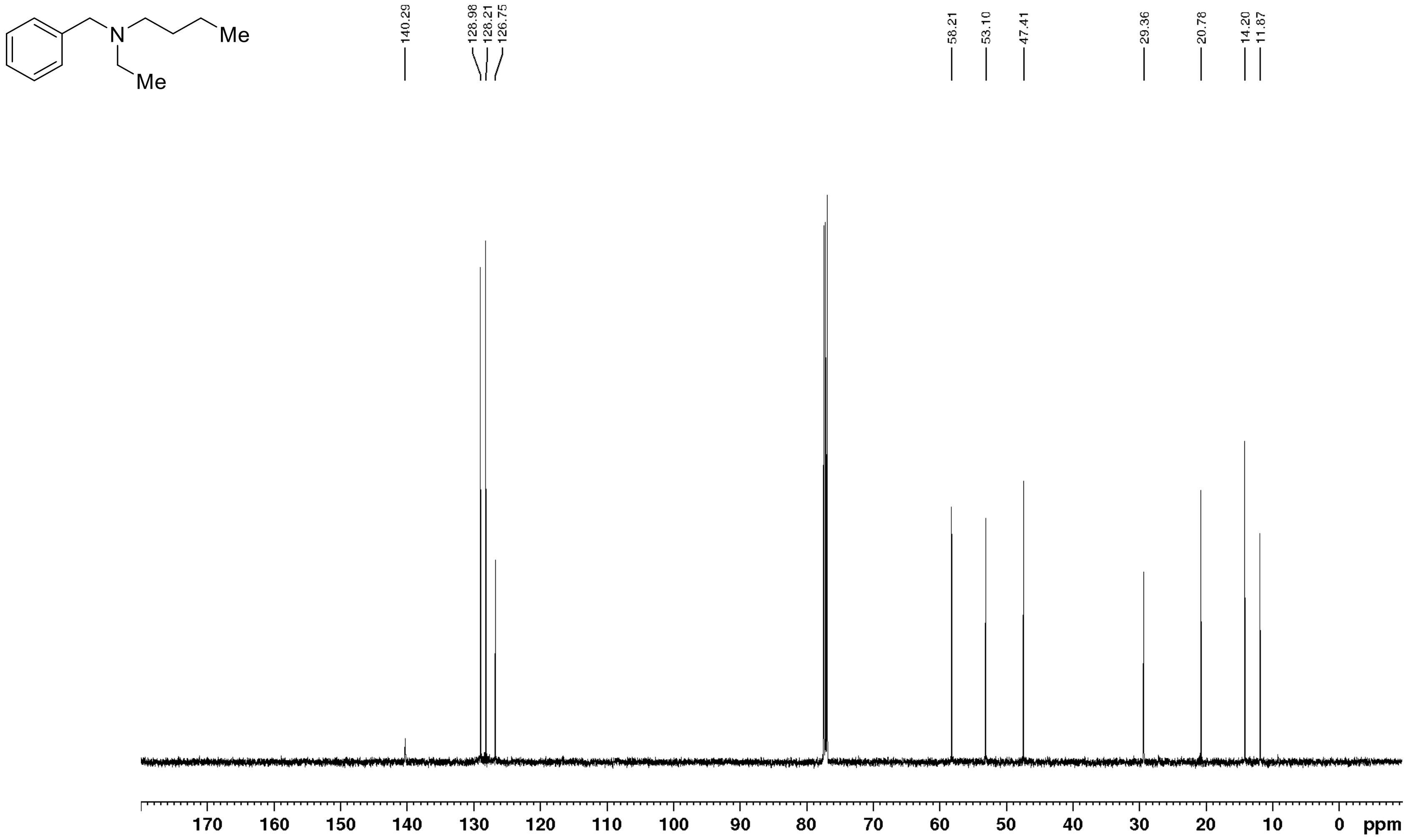

**Figure S5.** $^1$H NMR spectrum (500 MHz, $CDCl_3$, 298 K) of **1-benzyl-4,4-diphenyl-1,4-azasilinane (3aa)**.

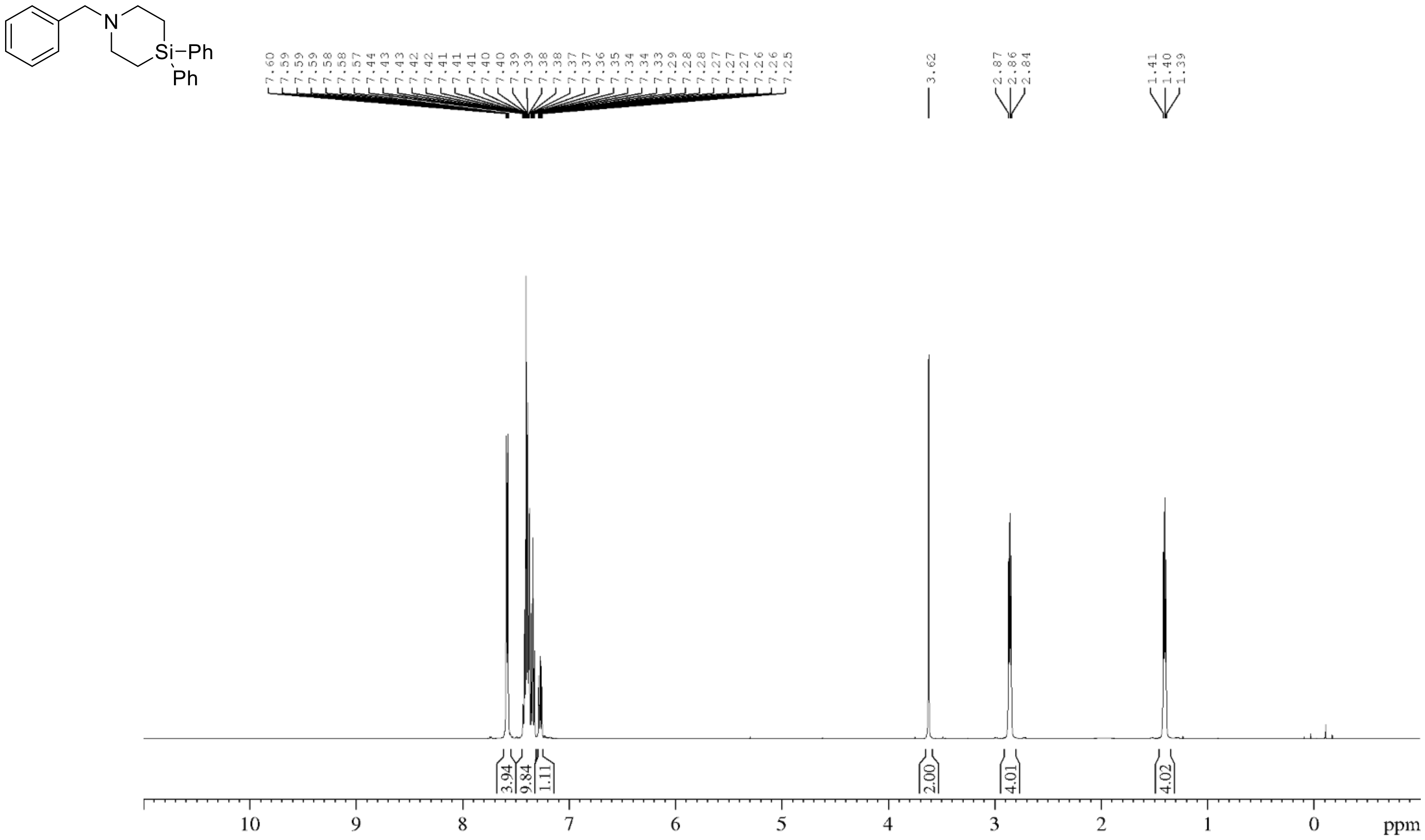

**Figure S6.** $^{13}$C NMR spectrum (125 MHz, $CDCl_3$, 298 K) of **1-benzyl-4,4-diphenyl-1,4-azasilinane (3aa)**.

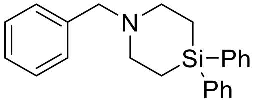


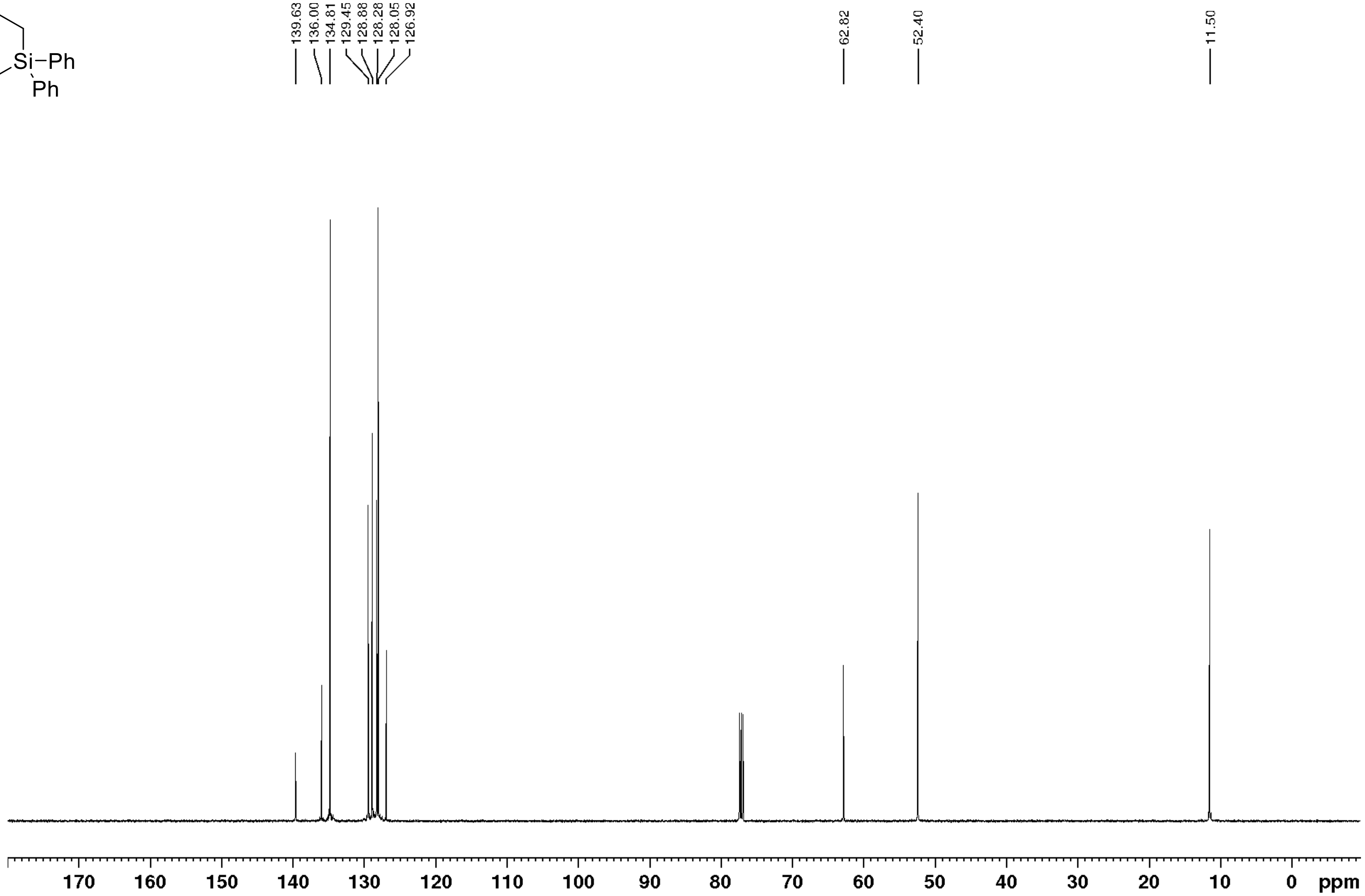

**Figure S7.** $^{1}H/^{29}Si$ HMQC NMR (500/99 MHz, $CDCl_3$, 298 K, optimized for $J$ = 7 Hz) of **1-benzyl-4,4-diphenyl-1,4-azasilinane (3aa)**.

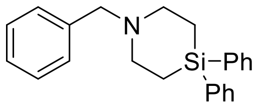


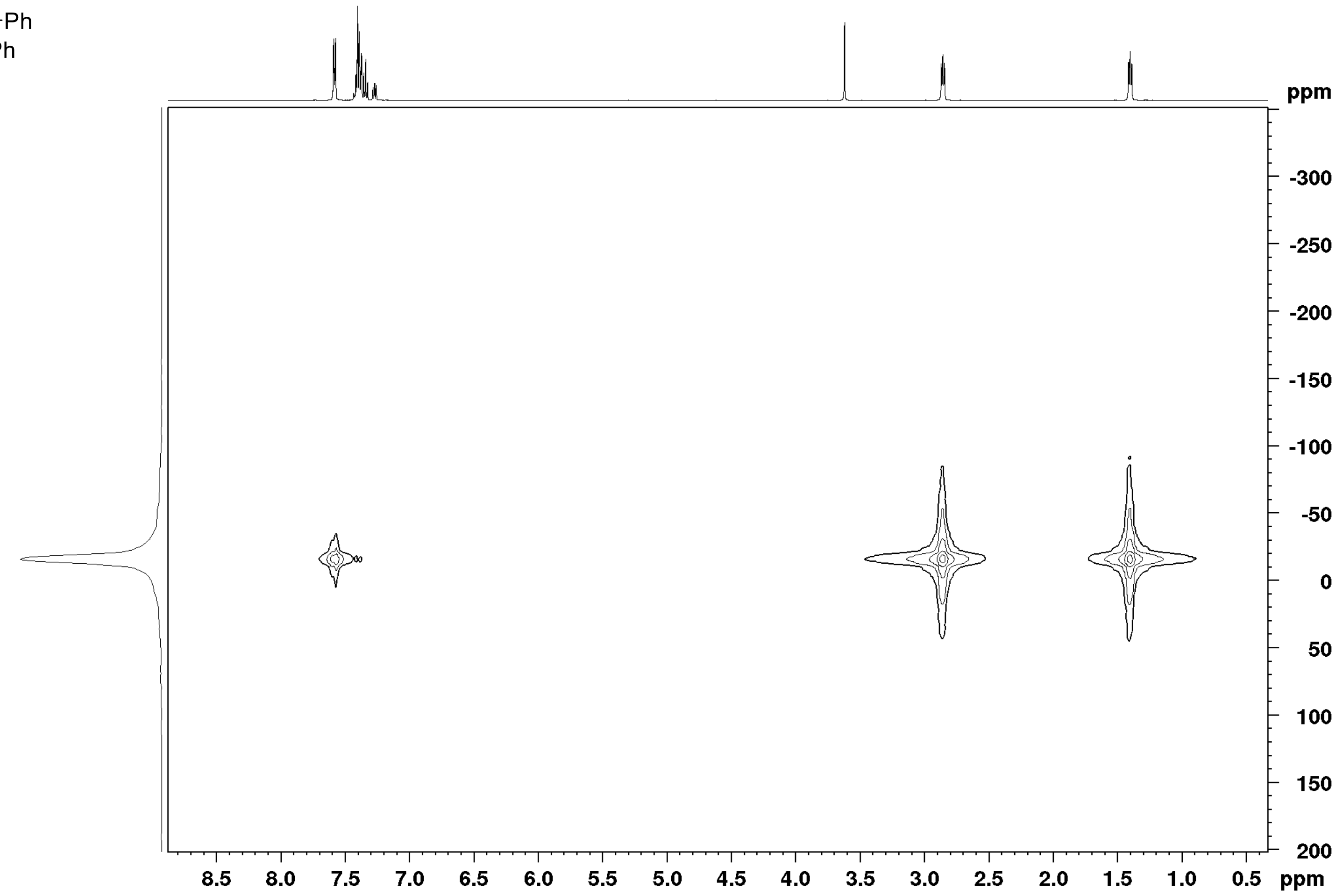

**Figure S8.** $^{1}H$ NMR spectrum (500 MHz, $CDCl_3$, 298 K) of **1-([1,1'-biphenyl]-4-ylmethyl)-4,4-diphenyl-1,4-azasilinane (3ba)**.

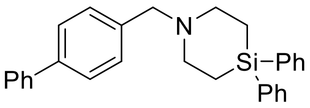


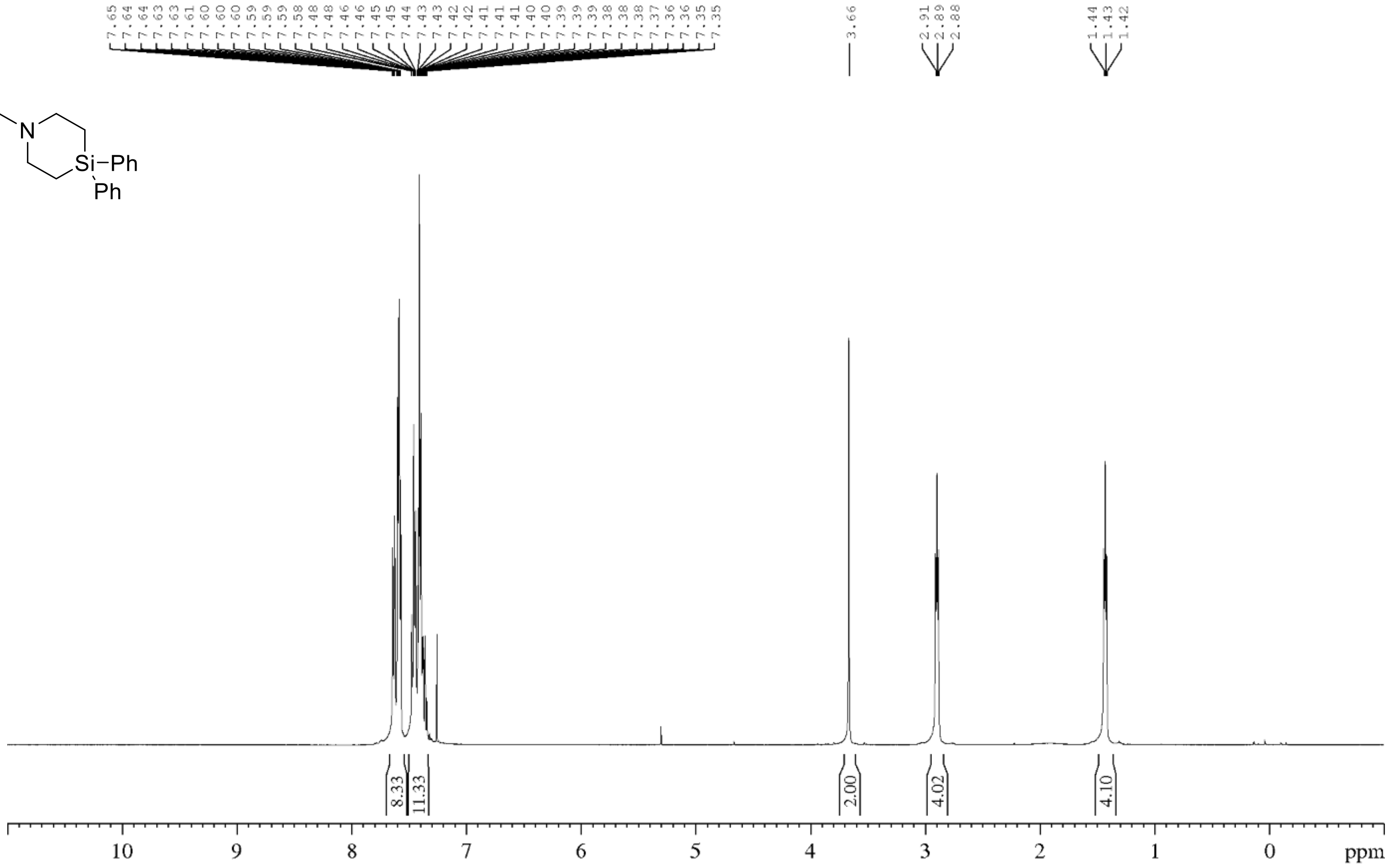

**Figure S9.** $^{13}C$ NMR spectrum (125 MHz, $CDCl_3$, 298 K) of **1-([1,1'-biphenyl]-4-ylmethyl)-4,4-diphenyl-1,4-azasilinane (3ba)**.

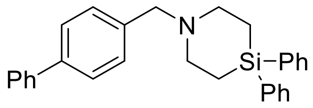


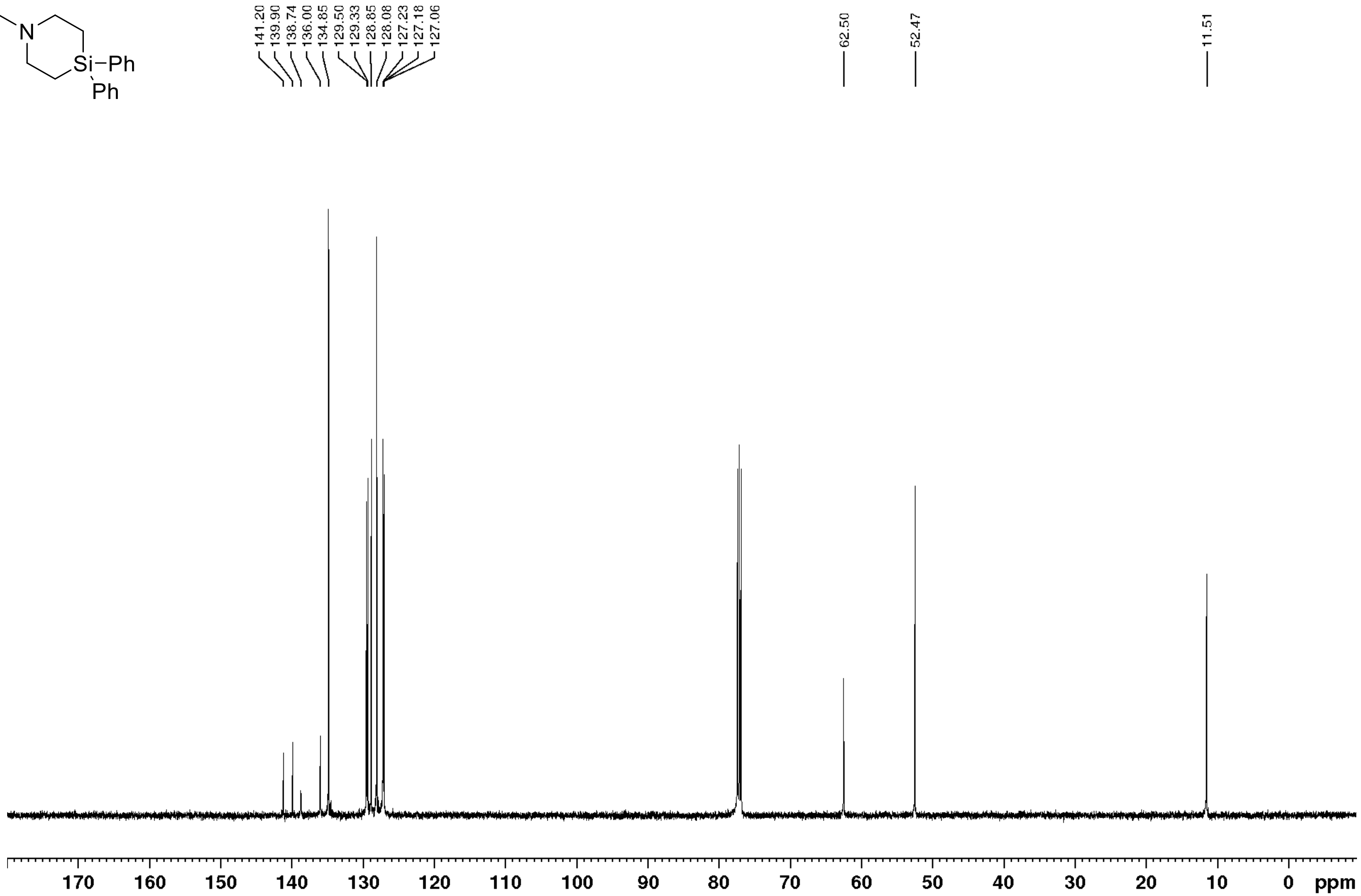

**Figure S10.** $^{1}H/^{29}Si$ HMQC NMR (500/99 MHz, $CDCl_3$, 298 K, optimized for $J$ = 7 Hz) of

**1-([1,1'-biphenyl]-4-ylmethyl)-4,4-diphenyl-1,4-azasilinane (3ba)**.

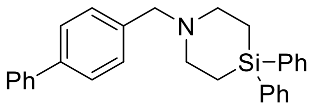


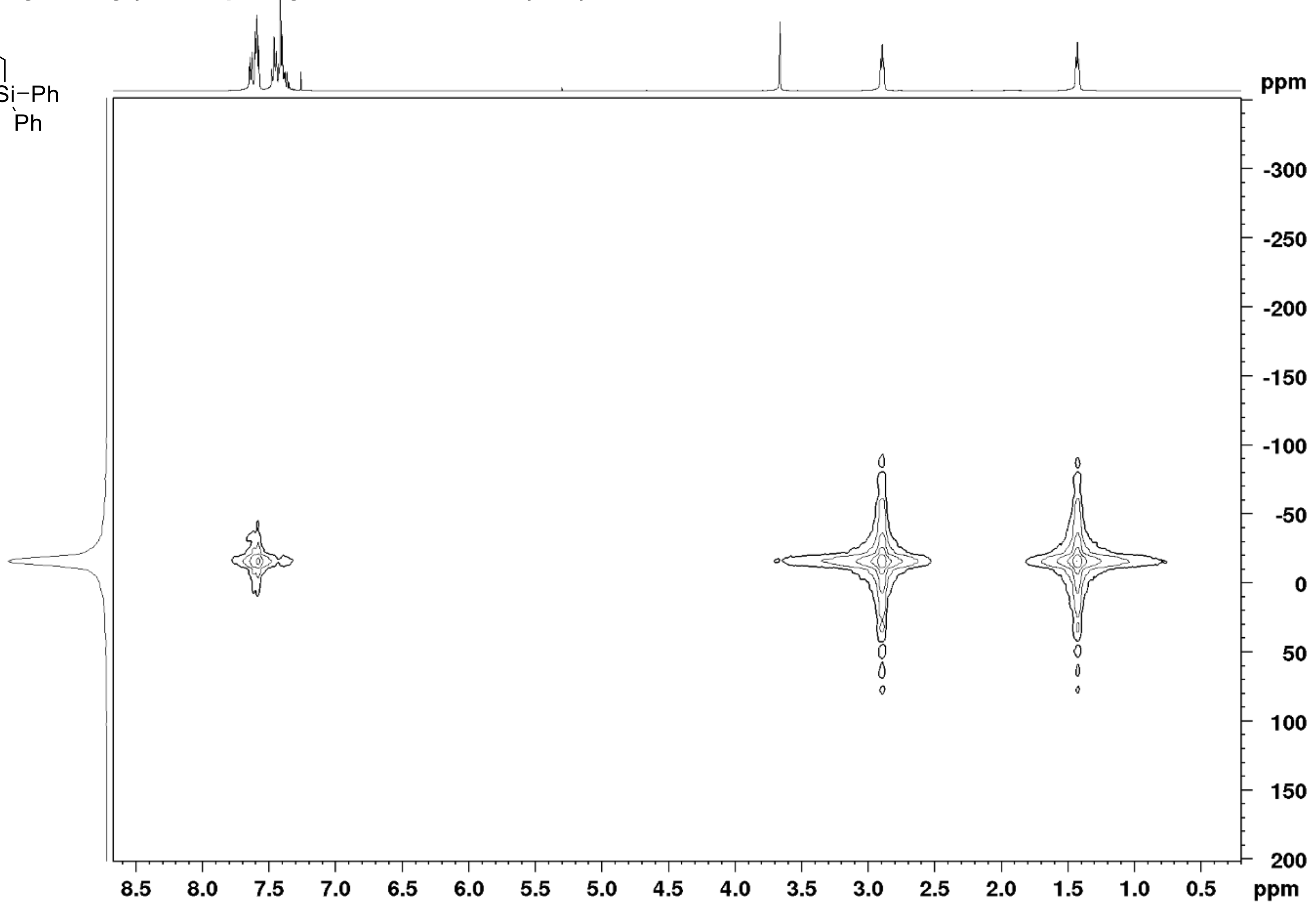

**Figure S11.** $^1$H NMR spectrum (500 MHz, $CDCl_3$, 298 K) of **1-(2-methylbenzyl)-4,4-diphenyl-1,4-azasilinane (3ca)**.

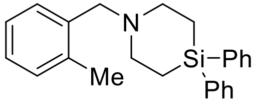


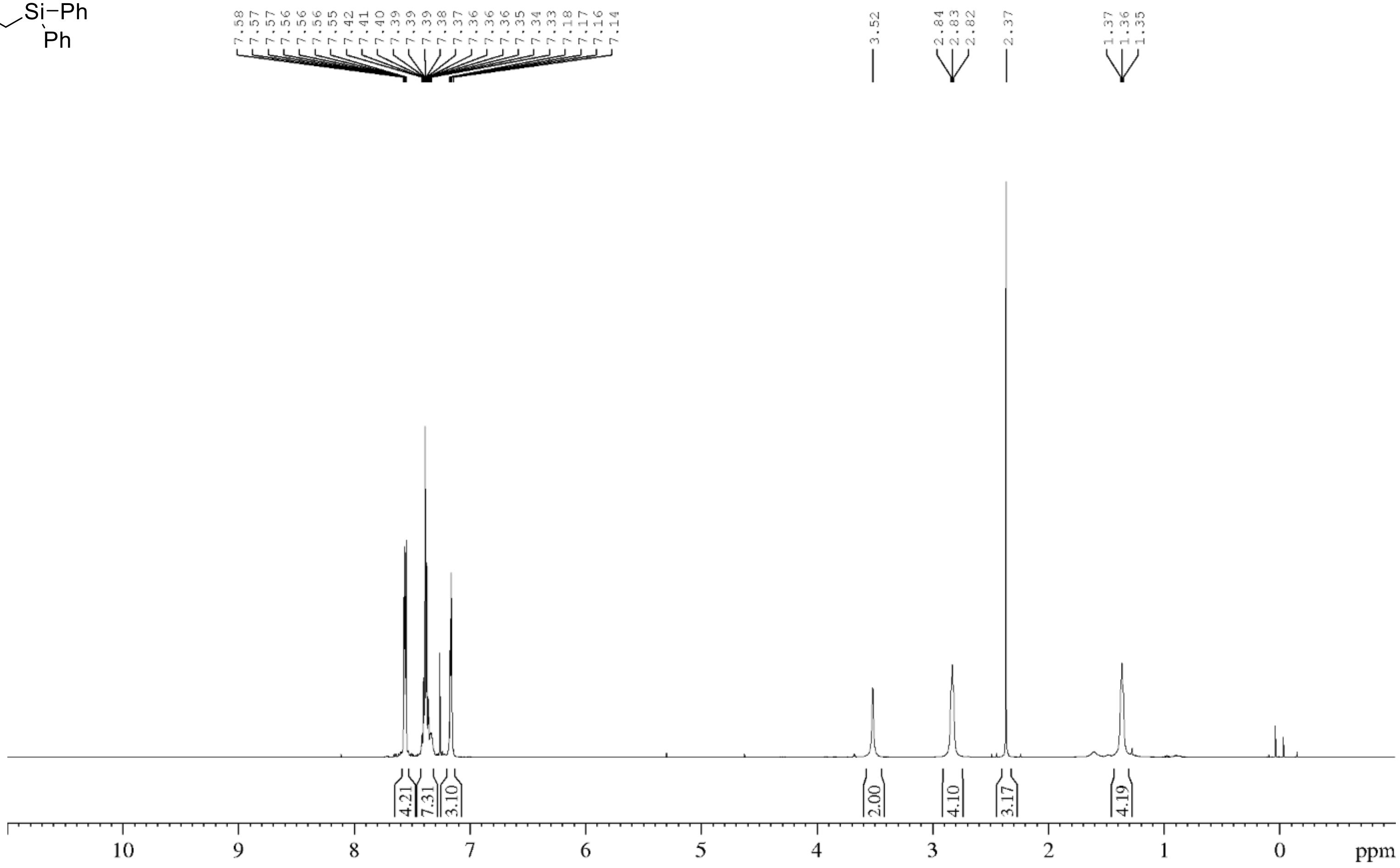

**Figure S12.** $^{13}C$ NMR spectrum (125 MHz, $CDCl_3$, 298 K) of **1-(2-methylbenzyl)-4,4-diphenyl-1,4-azasilinane (3ca)**.

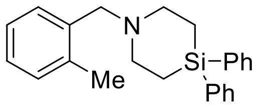


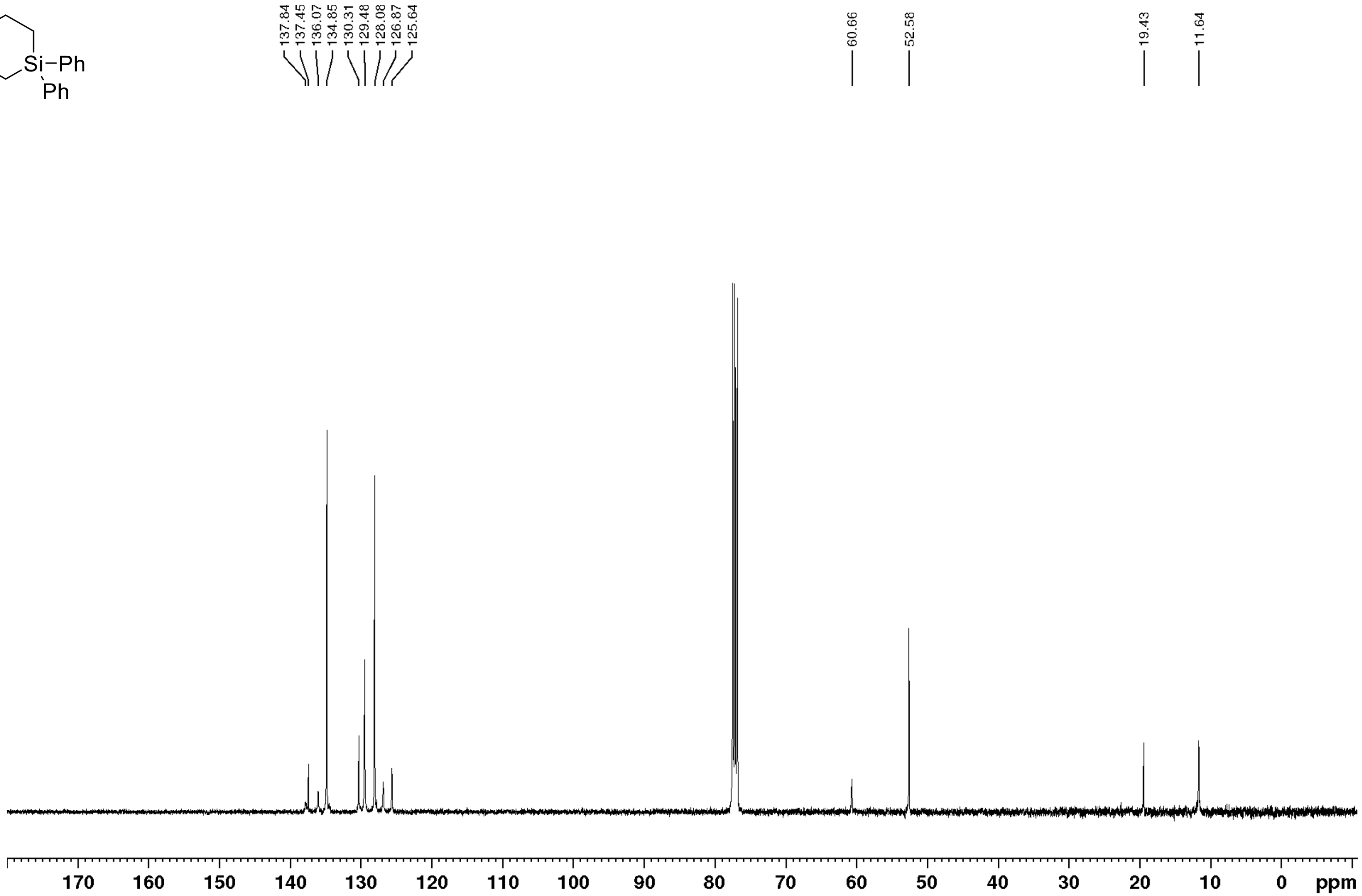

**Figure S13.** $^{1}H/^{29}Si$ HMQC NMR (500/99 MHz, $CDCl_3$, 298 K, optimized for $J$ = 7 Hz) of **1-(2-methylbenzyl)-4,4-diphenyl-1,4-azasilinane (3ca)**.

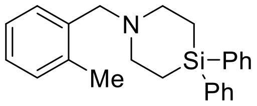


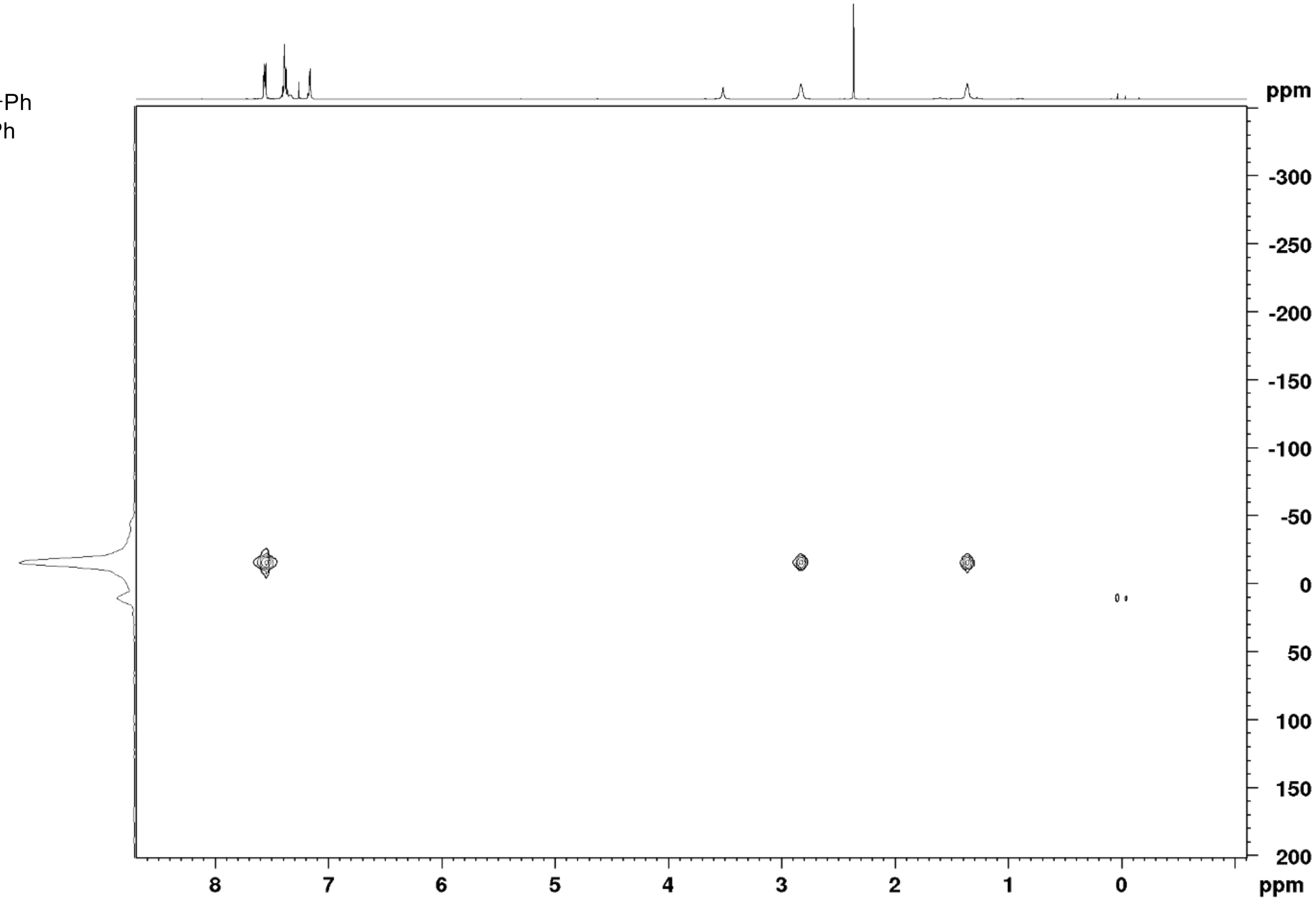

**Figure S14.** $^1$H NMR spectrum (500 MHz, $CDCl_3$, 298 K) of **1-(3-methylbenzyl)-4,4-diphenyl-1,4-azasilinane (3da)**.

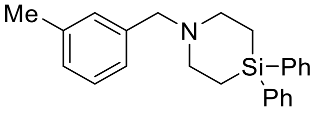


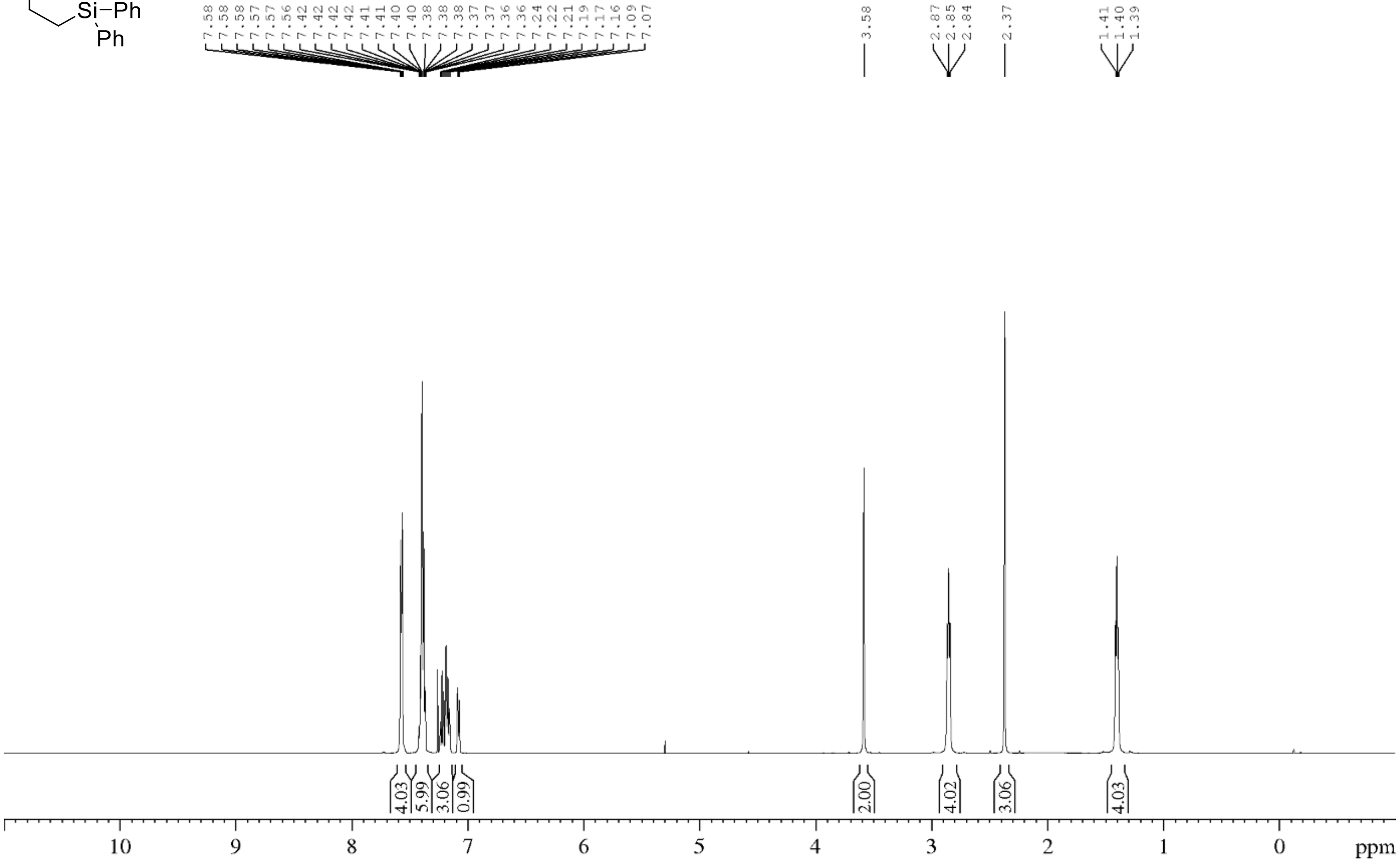

**Figure S15.** $^{13}$C NMR spectrum (125 MHz, $CDCl_3$, 298 K) of **1-(3-methylbenzyl)-4,4-diphenyl-1,4-azasilinane (3da)**.

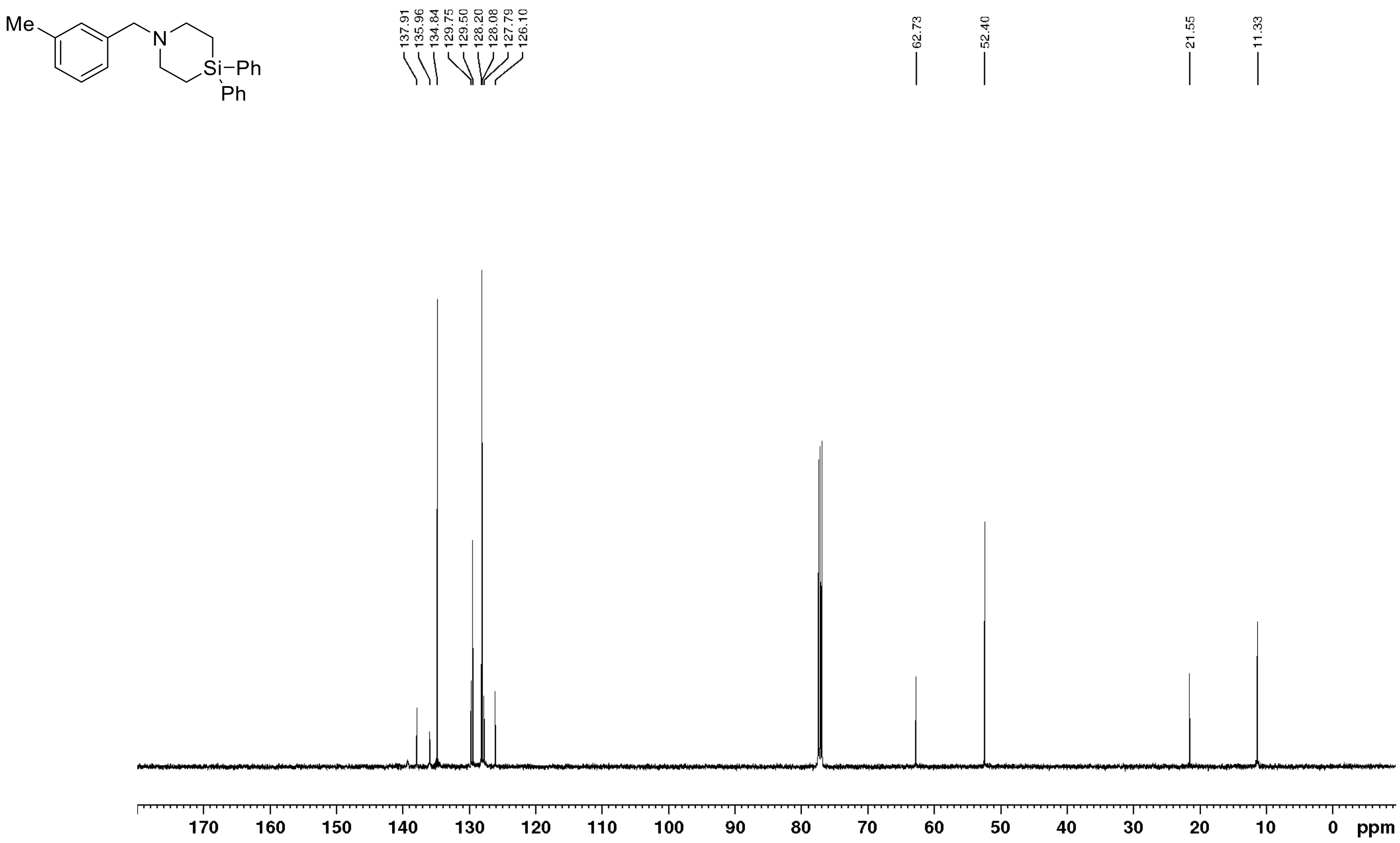

**Figure S16.** $^1H/^{29}Si$ HMQC NMR (500/99 MHz, $CDCl_3$, 298 K, optimized for $J$ = 7 Hz) of **1-(3-methylbenzyl)-4,4-diphenyl-1,4-azasilinane (3da)**.

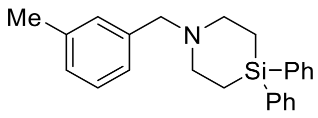


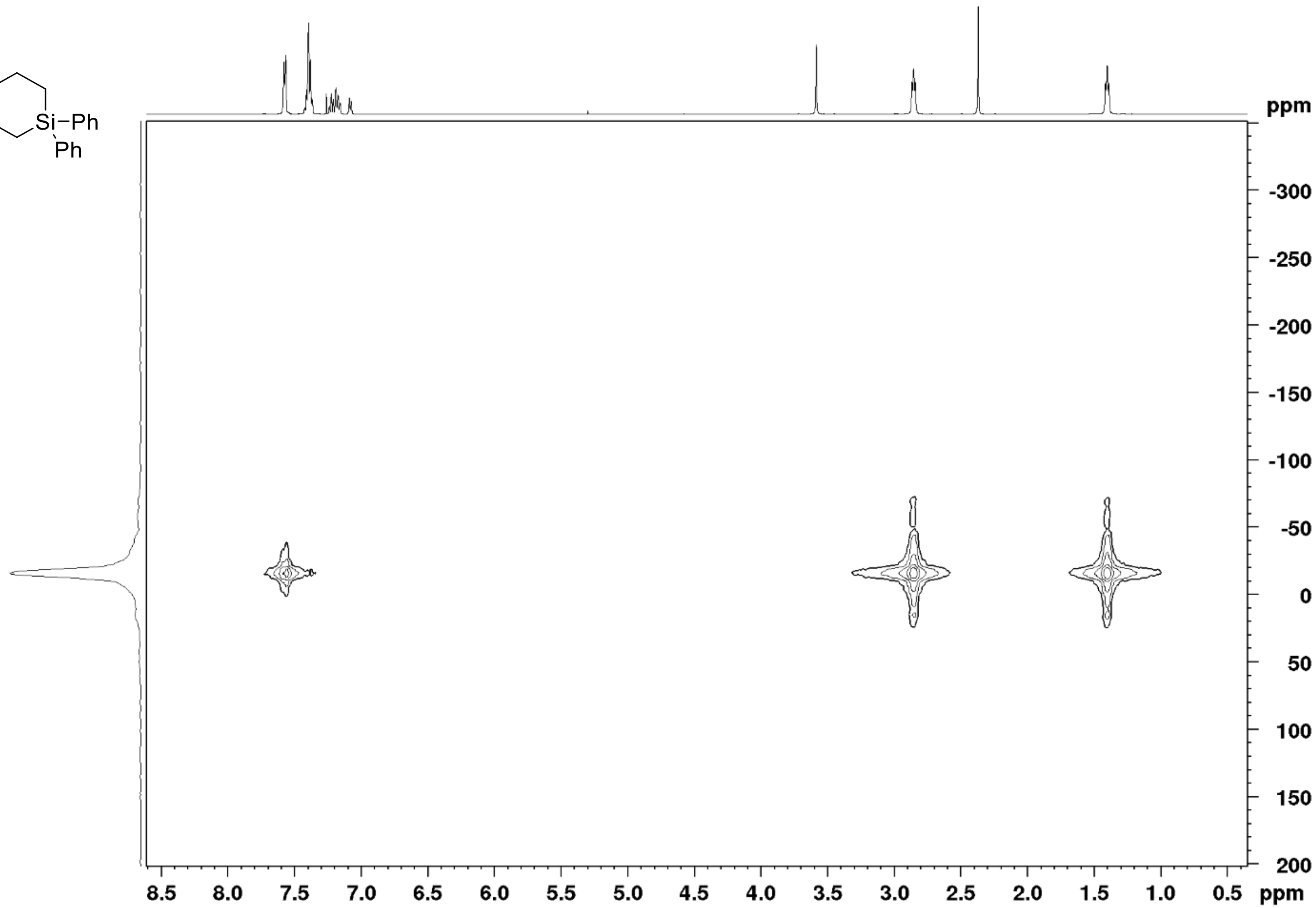

**Figure S17.** $^{1}H$ NMR spectrum (500 MHz, $CDCl_3$, 298 K) of **1-(4-methylbenzyl)-4,4-diphenyl-1,4-azasilinane (3ea)**.

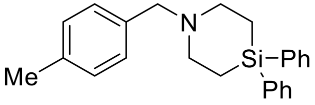


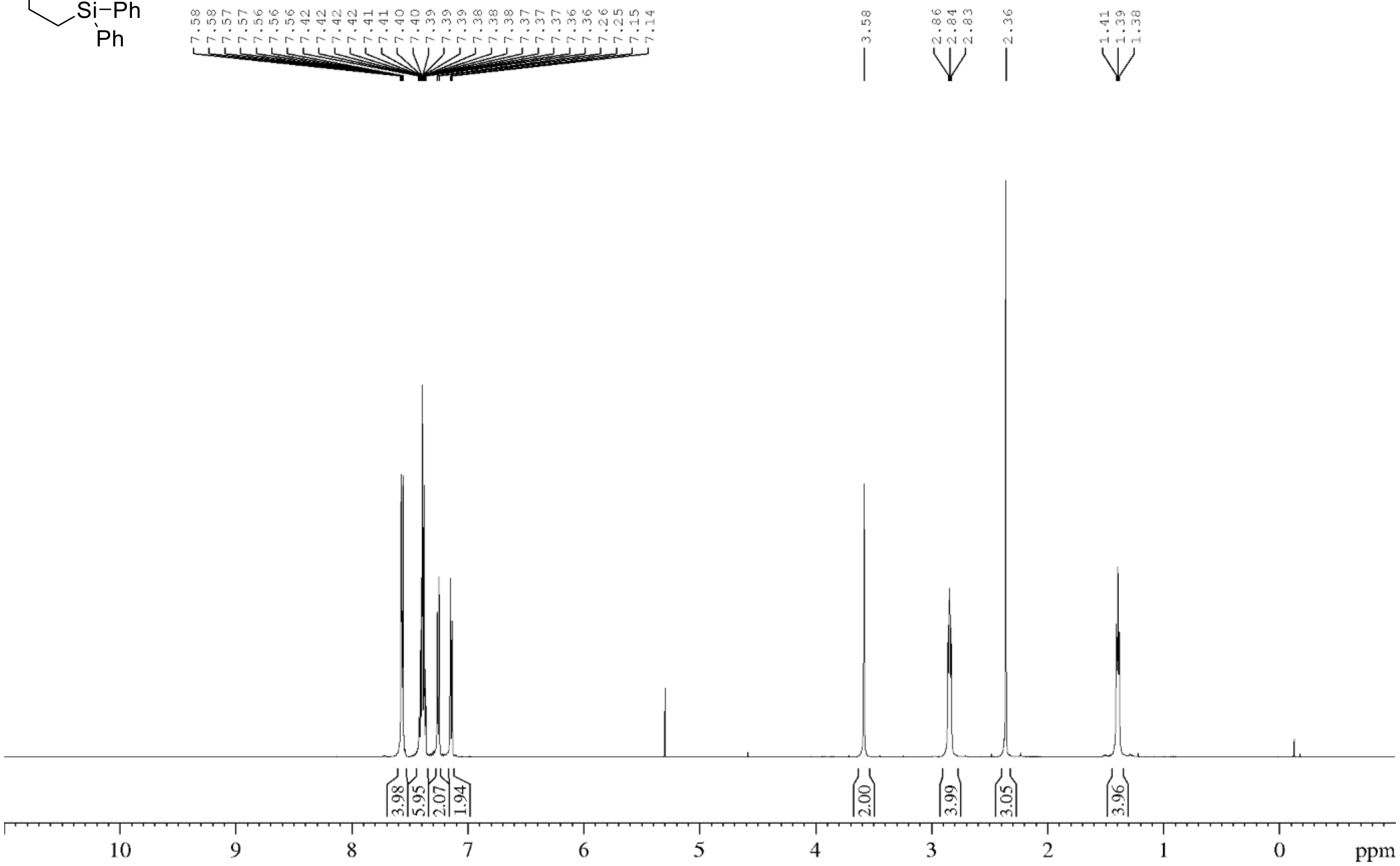

**Figure S18.** $^{13}$C NMR spectrum (125 MHz, $CDCl_3$, 298 K) of **1-(4-methylbenzyl)-4,4-diphenyl-1,4-azasilinane (3ea)**.

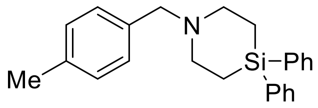


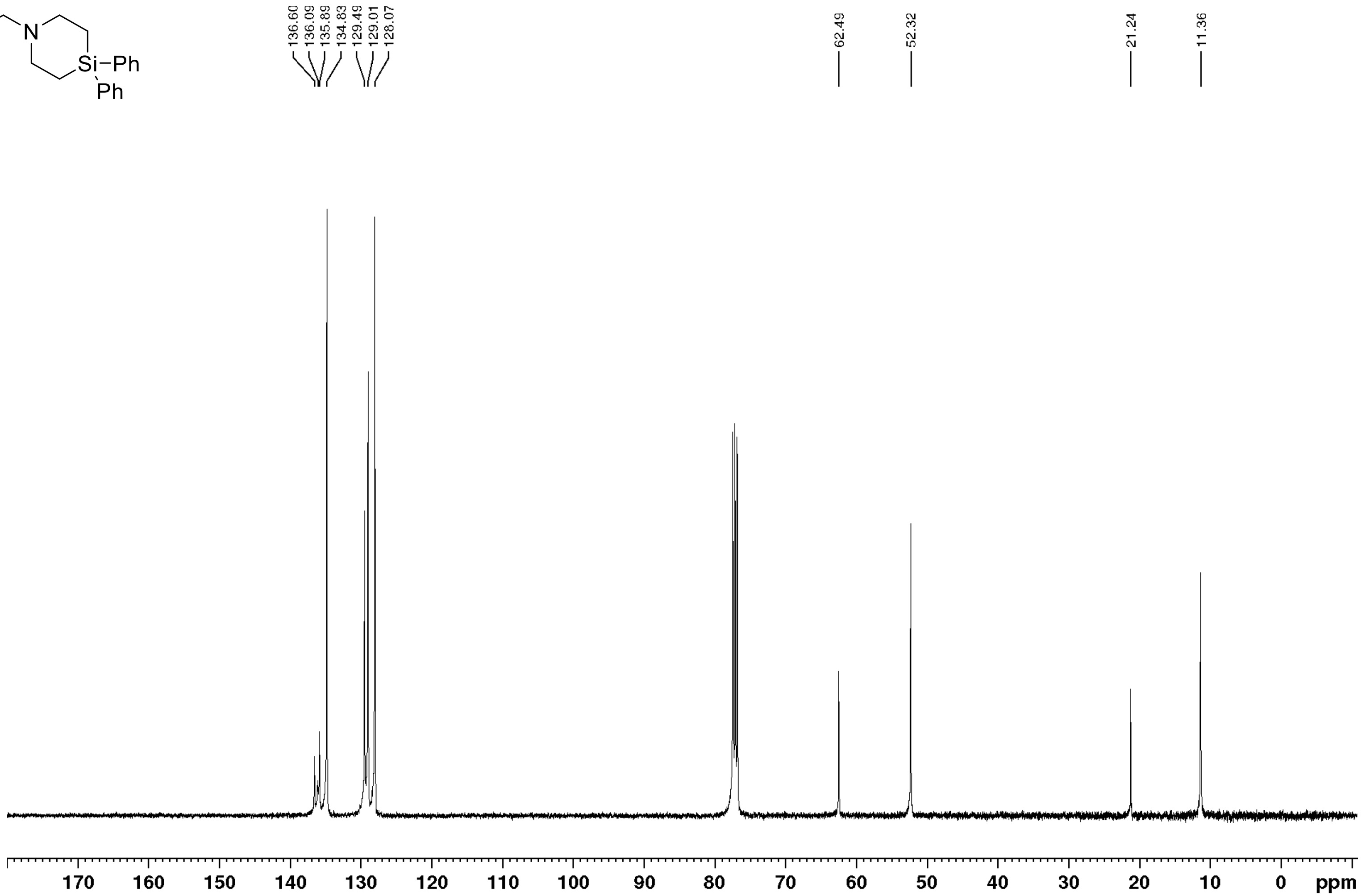

**Figure S19.** $^{1}H/^{29}Si$ HMQC NMR (500/99 MHz, $CDCl_3$, 298 K, optimized for $J$ = 7 Hz) of **1-(4-methylbenzyl)-4,4-diphenyl-1,4-azasilinane (3ea)**.

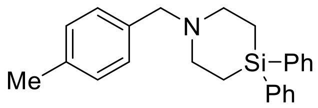


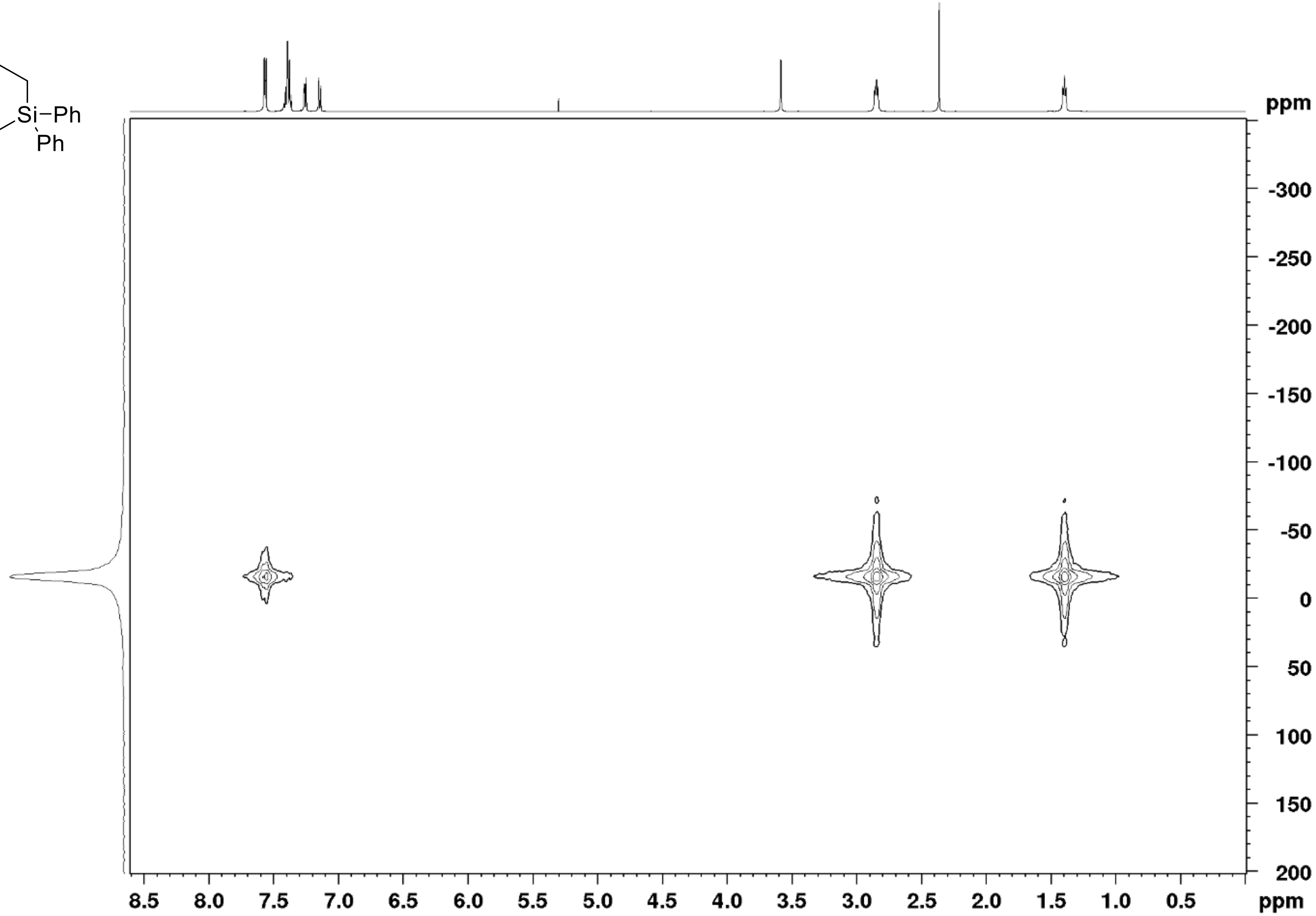

**Figure S20.** $^1$H NMR spectrum (500 MHz, $CDCl_3$, 298 K) of **1-(4-(*tert*-butyl)benzyl)-4,4-diphenyl-1,4-azasilinane (3fa)**.

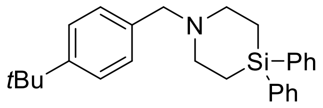


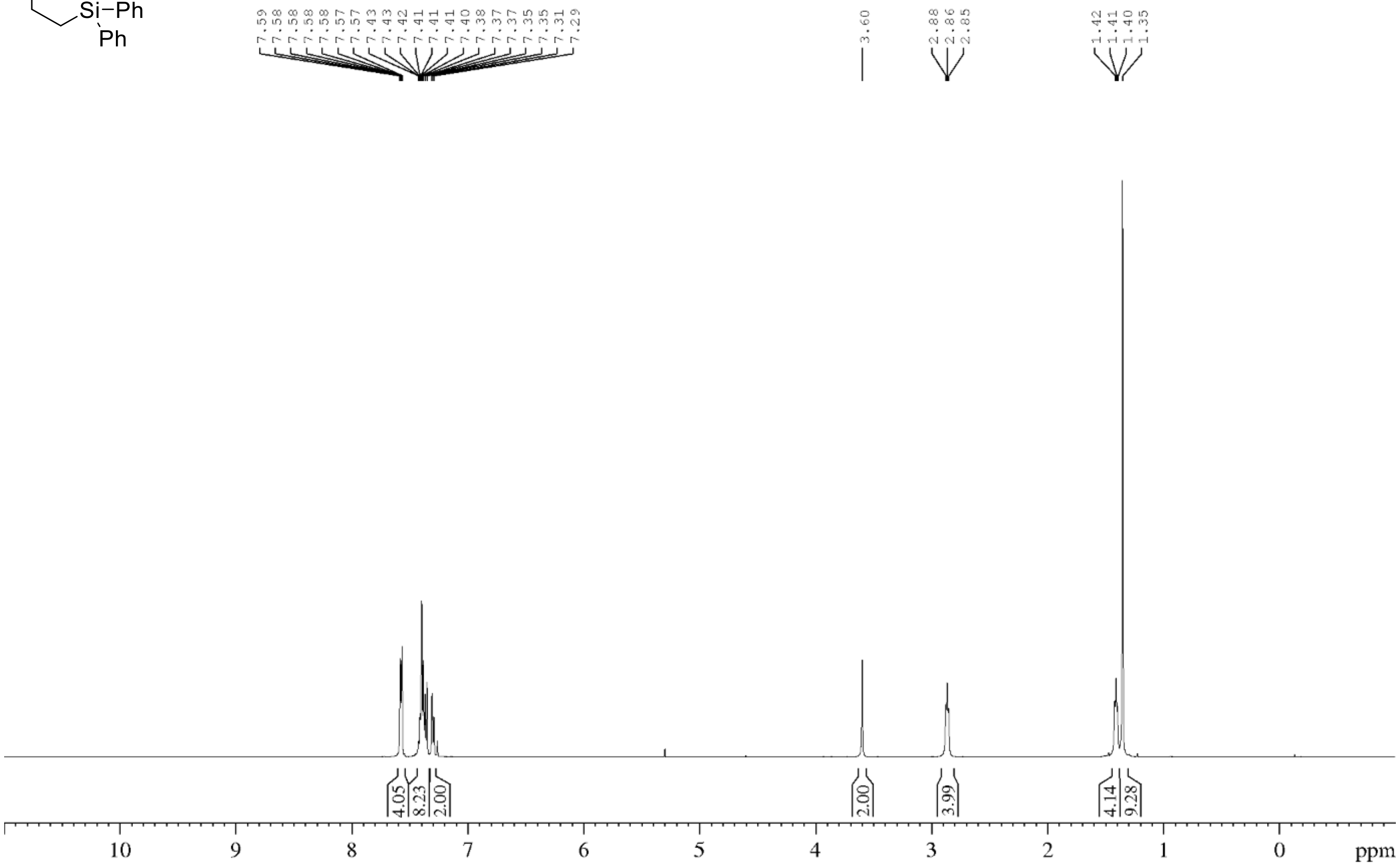

**Figure S21.** $^{13}$C NMR spectrum (125 MHz, $CDCl_3$, 298 K) of **1-(4-(*tert*-butyl)benzyl)-4,4-diphenyl-1,4-azasilinane (3fa)**.

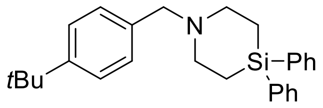


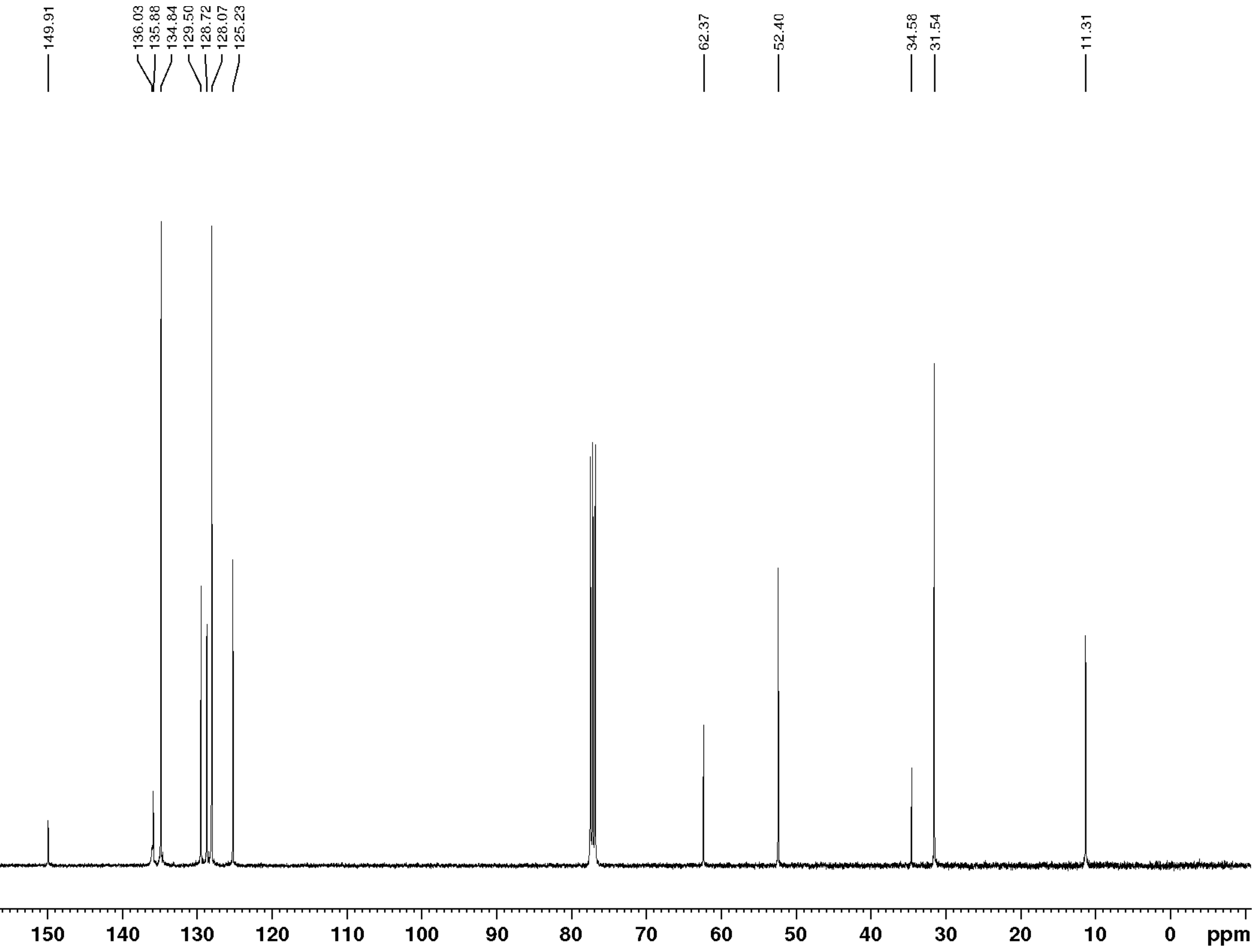

**Figure S22.** $^1$H/$^{29}$Si HMQC NMR (500/99 MHz, $CDCl_3$, 298 K, optimized for *J* = 7 Hz) of **1-(4-(*tert*-butyl)benzyl)-4,4-diphenyl-1,4-azasilinane (3fa)**.

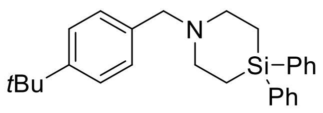


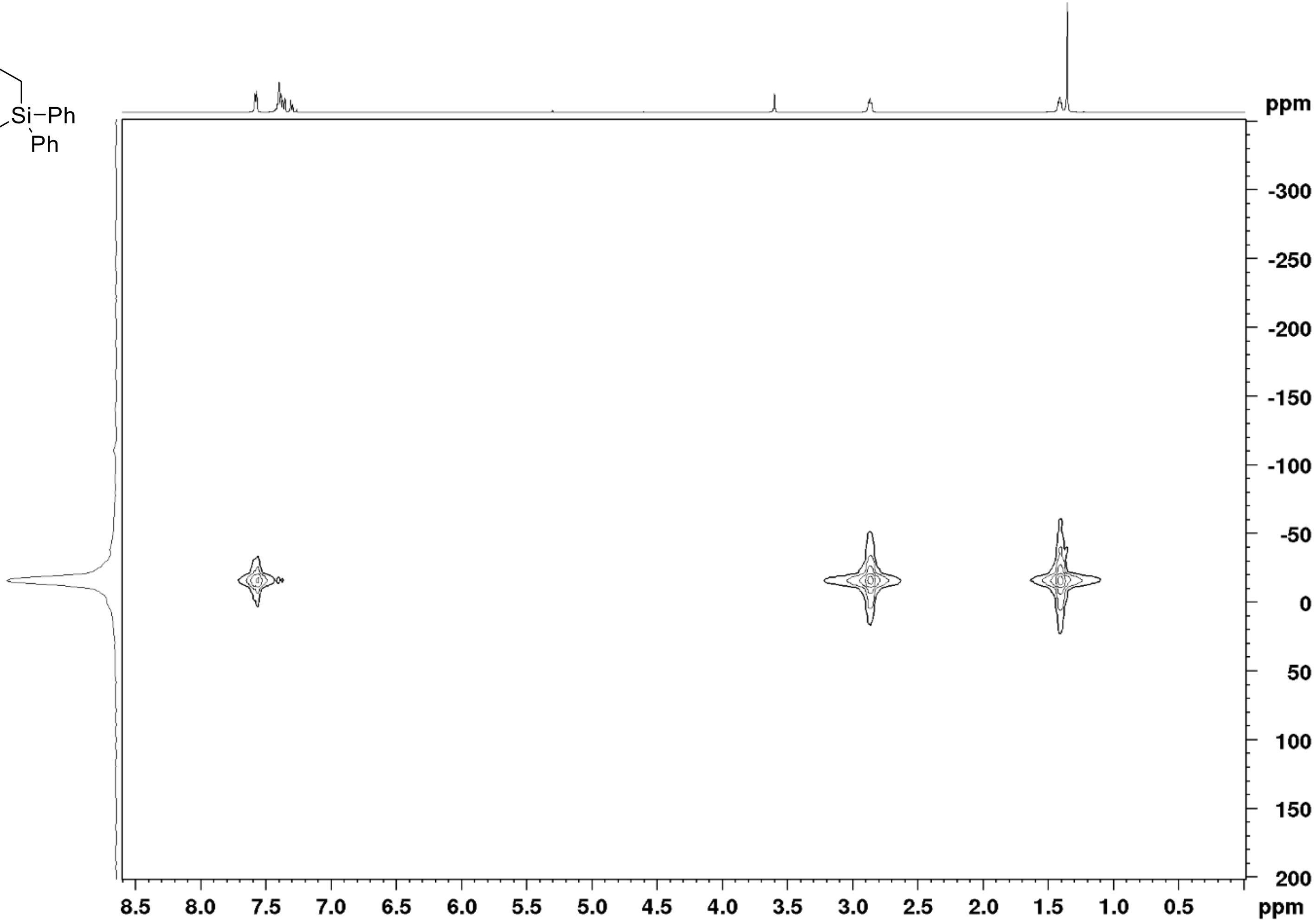

**Figure S23.** $^1$H NMR spectrum (500 MHz, $CDCl_3$, 298 K) of **1-(4-fluorobenzyl)-4,4-diphenyl-1,4-azasilinane (3ga)**.

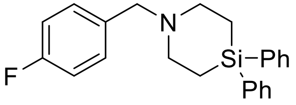


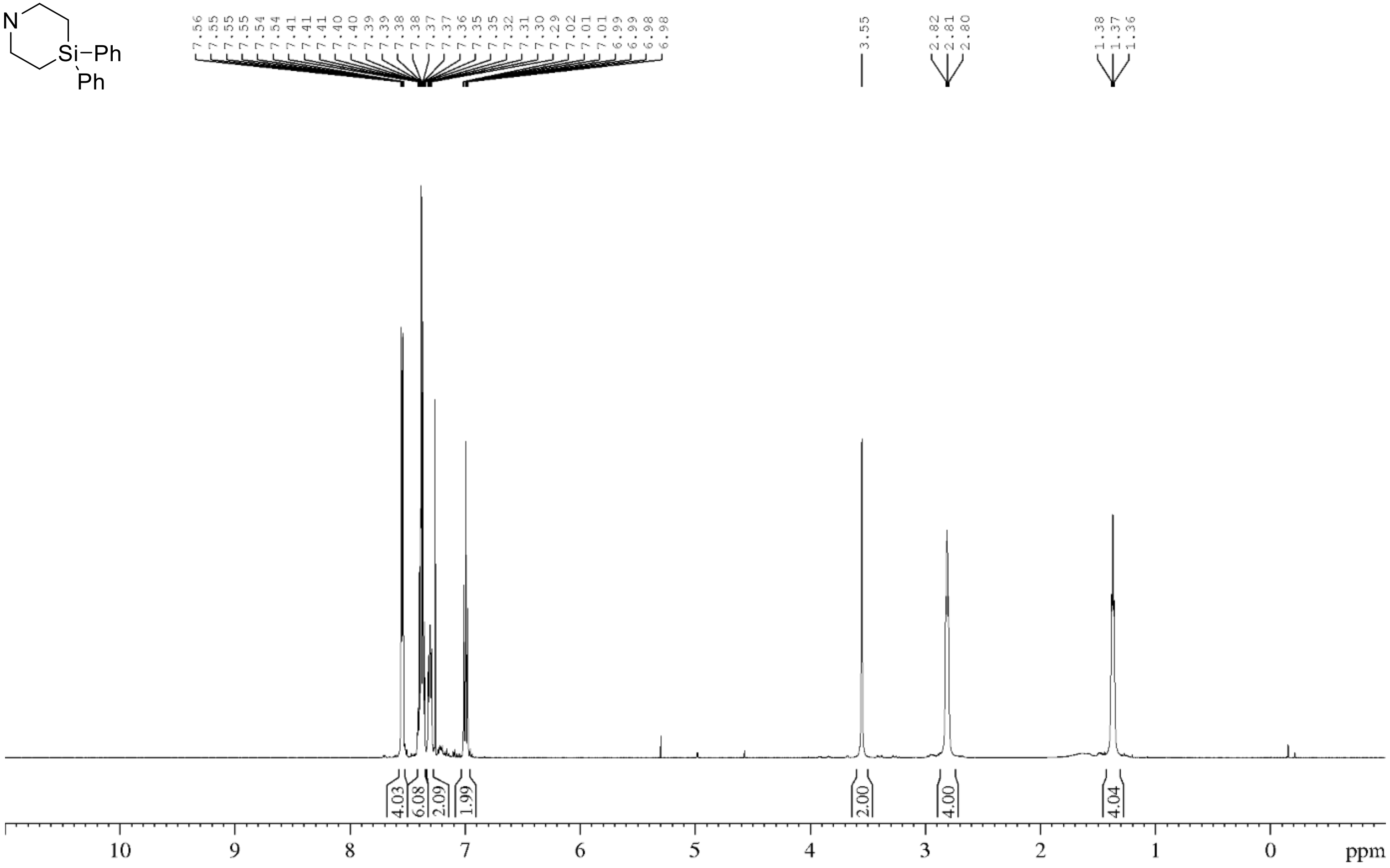

**Figure S24.** $^{13}C$ NMR spectrum (125 MHz, $CDCl_3$, 298 K) of **1-(4-fluorobenzyl)-4,4-diphenyl-1,4-azasilinane (3ga)**.

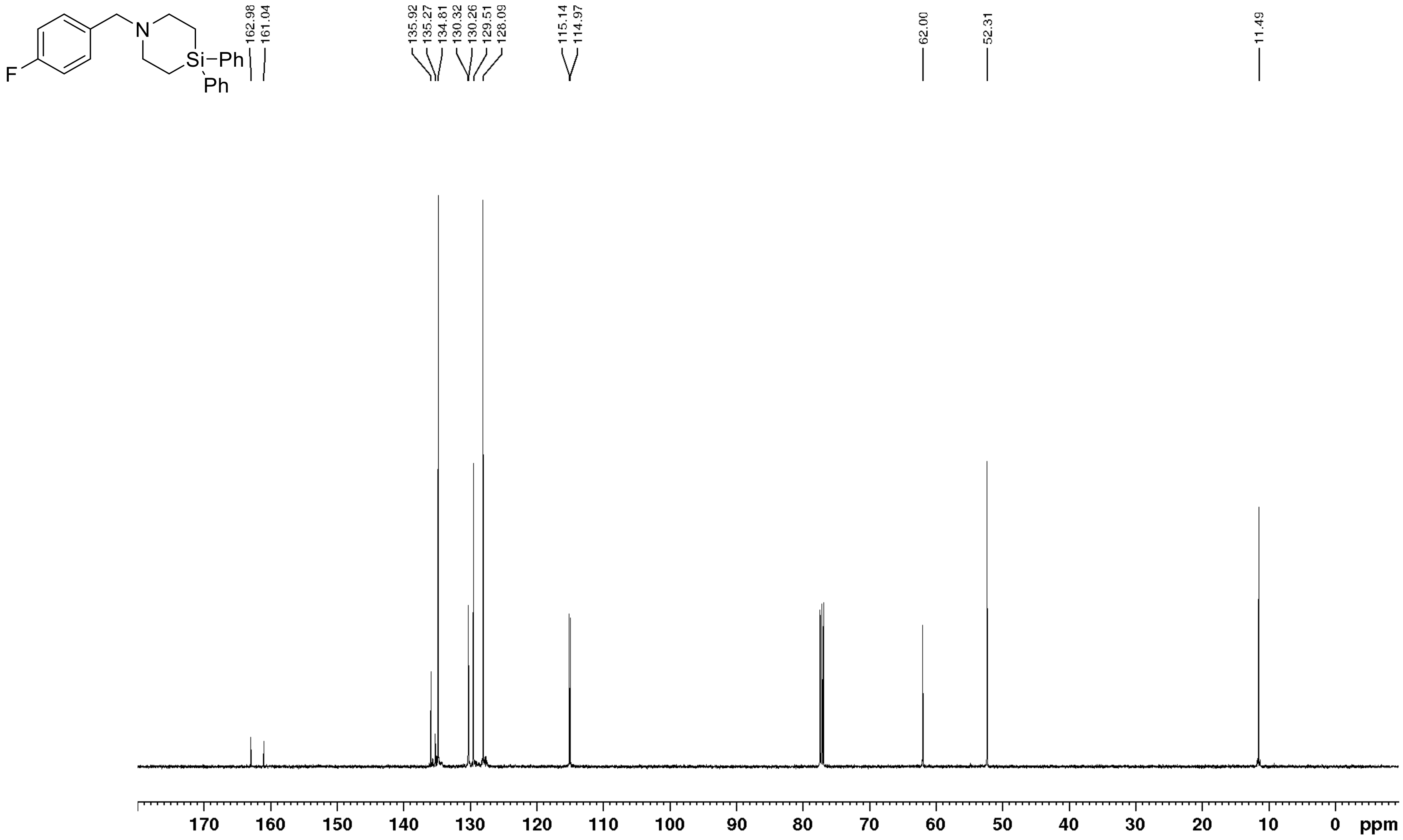

**Figure S25.** $^{19}F$ NMR spectrum (471 MHz, $CDCl_3$, 298 K) of **1-(4-fluorobenzyl)-4,4-diphenyl-1,4-azasilinane (3ga)**.

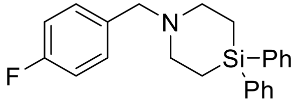

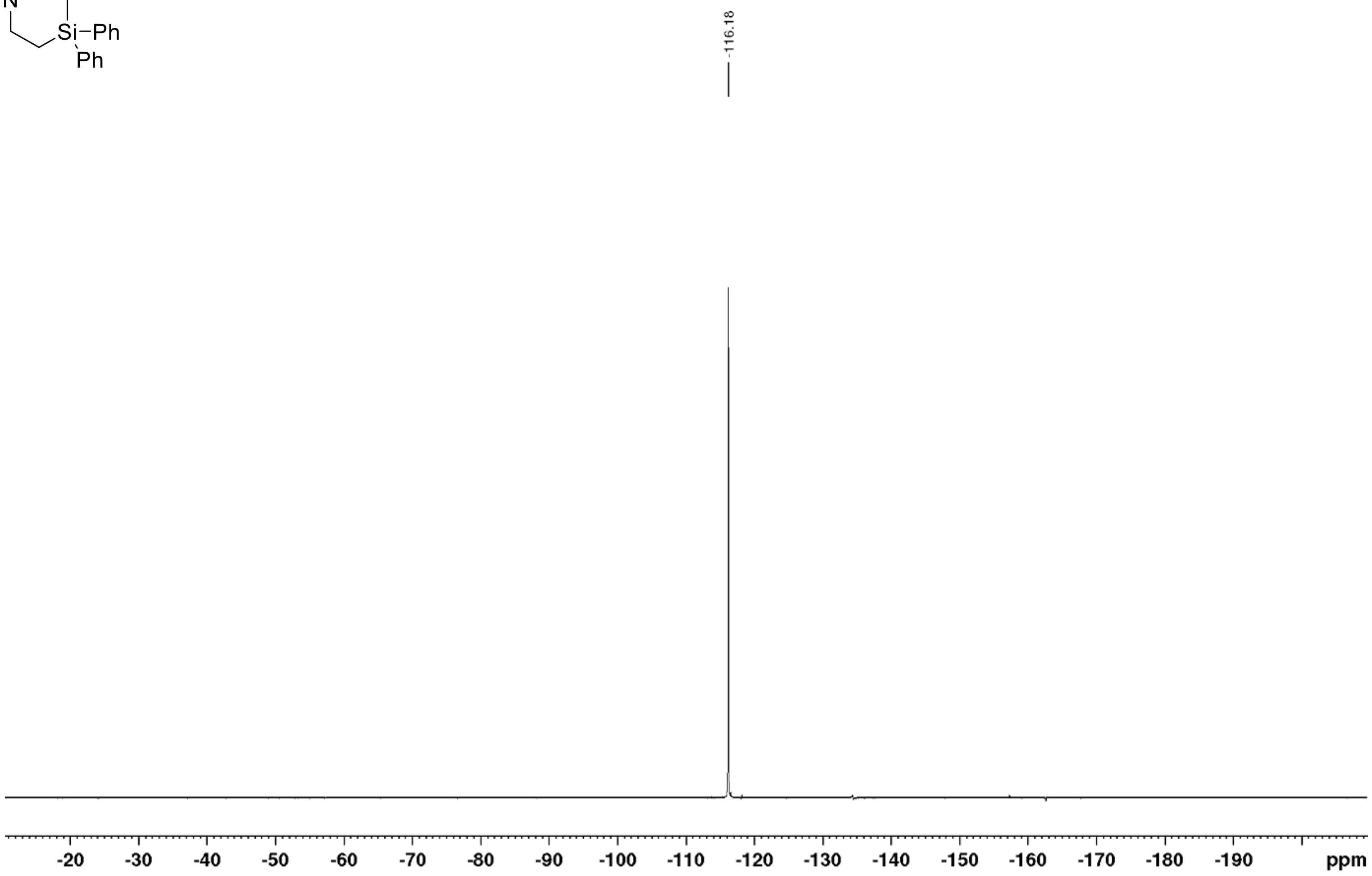

**Figure S26.** $^{1}$H/$^{29}$Si HMQC NMR (500/99 MHz, $CDCl_3$, 298 K, optimized for $J$ = 7 Hz) of **1-(4-fluorobenzyl)-4,4-diphenyl-1,4-azasilinane (3ga)**.

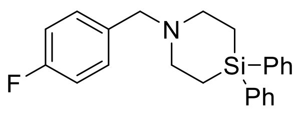

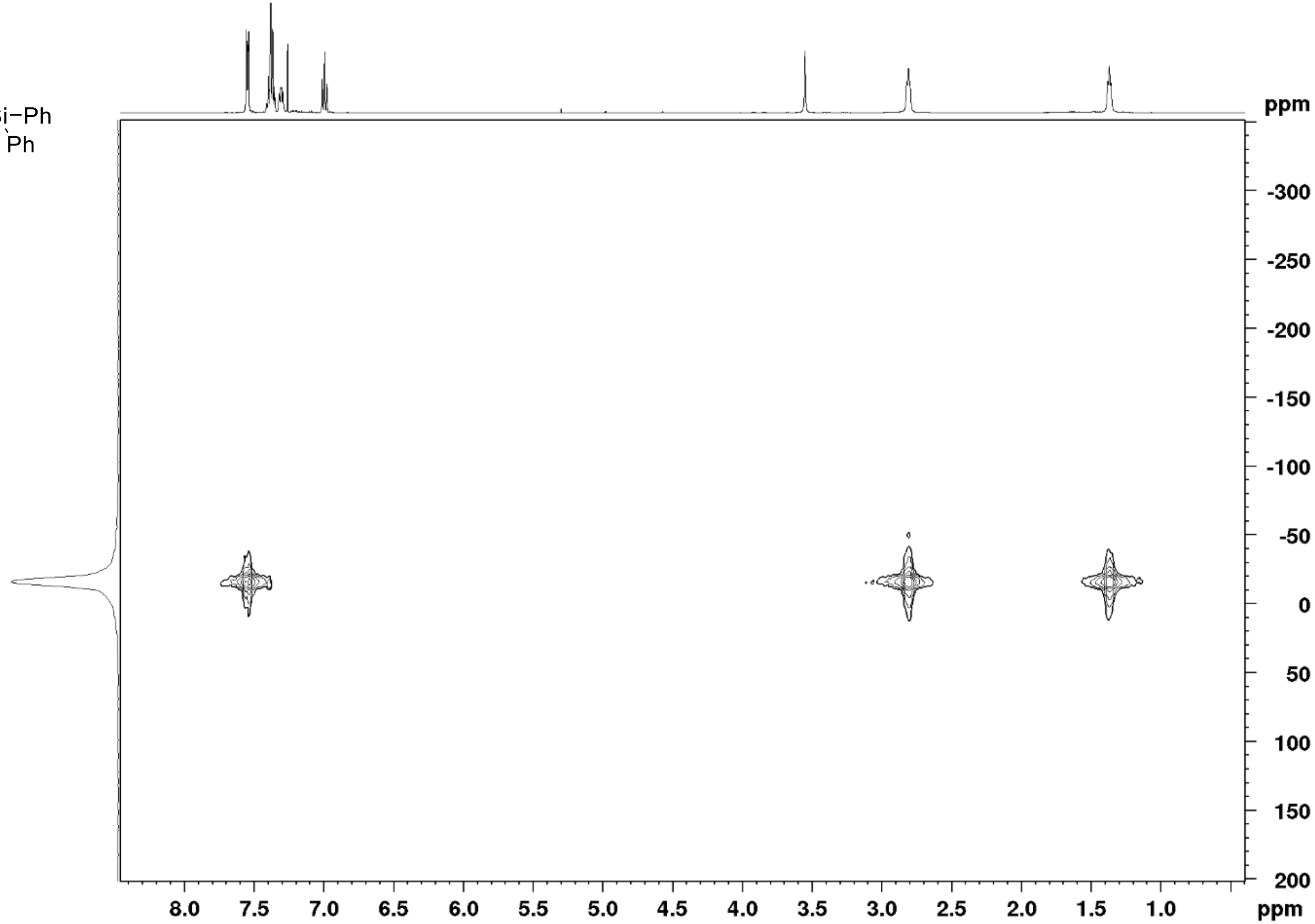

**Figure S27.** $^{1}$H NMR spectrum (500 MHz, $CDCl_3$, 298 K) of **1-(4-chlorobenzyl)-4,4-diphenyl-1,4-azasilinane (3ha)**.

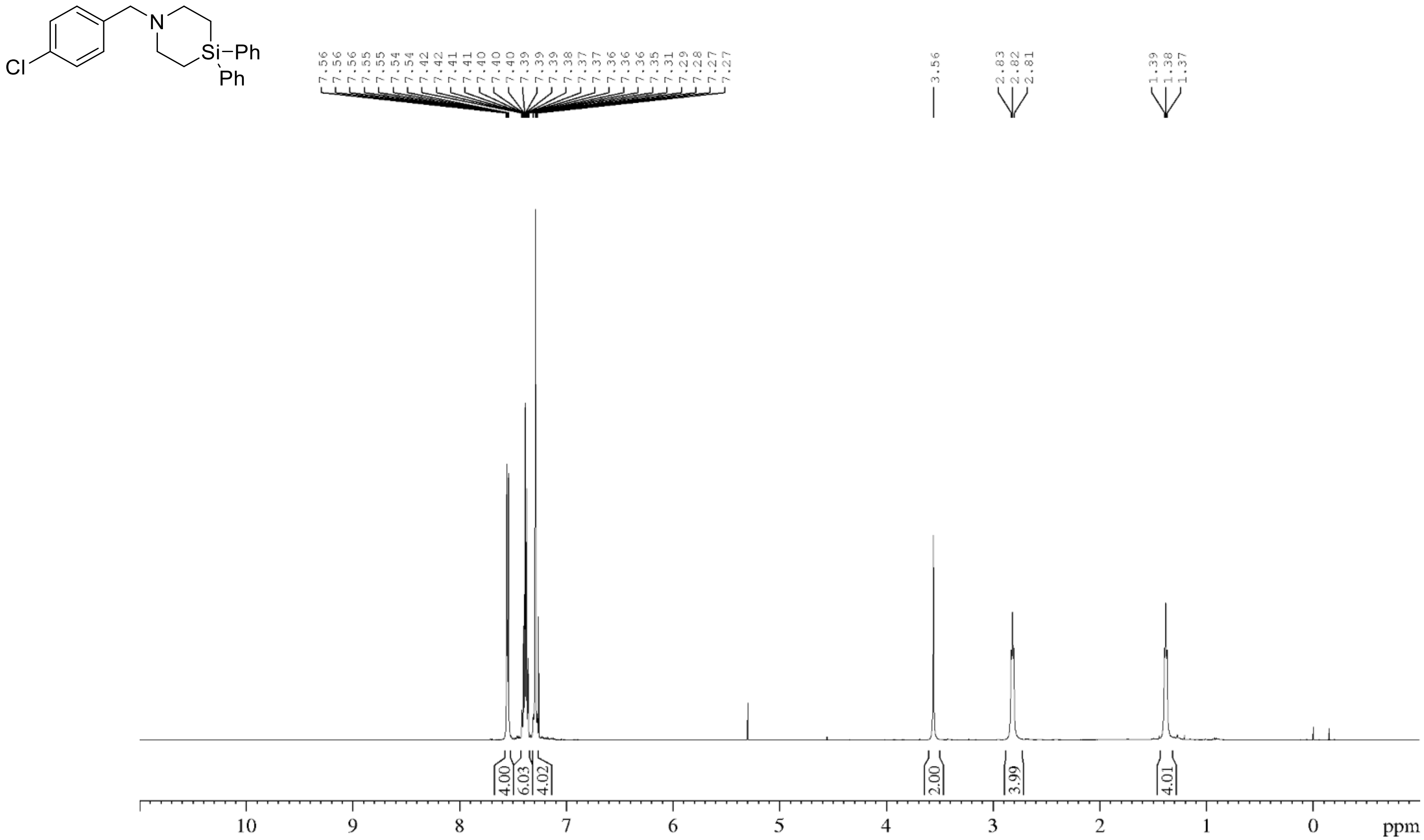

**Figure S28.** $^{13}C$ NMR spectrum (125 MHz, $CDCl_3$, 298 K) of **1-(4-chlorobenzyl)-4,4-diphenyl-1,4-azasilinane (3ha)**.

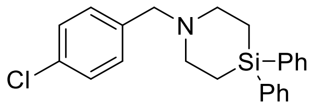


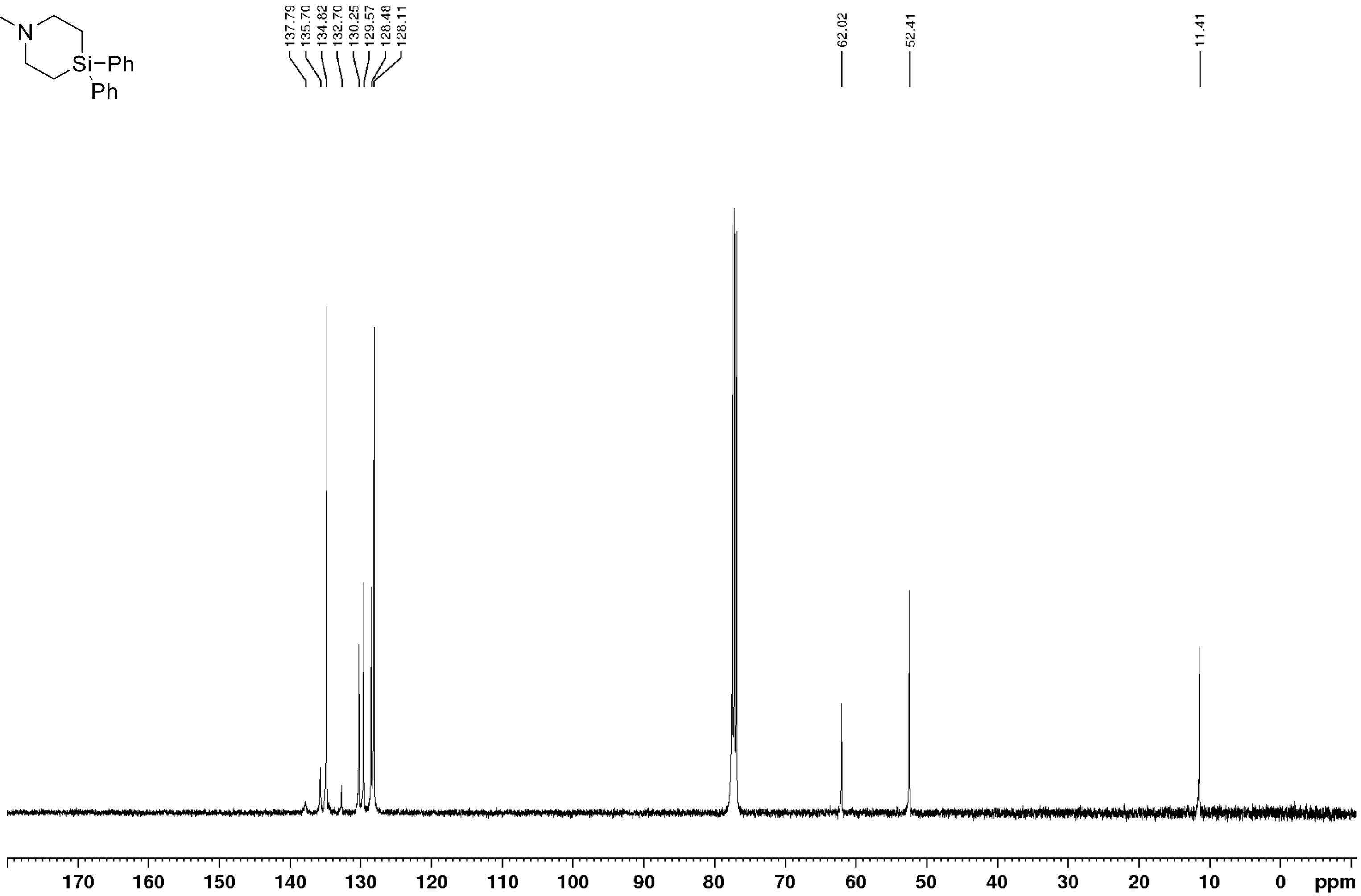

**Figure S29.** $^{1}H/^{29}Si$ HMQC NMR (500/99 MHz, $CDCl_3$, 298 K, optimized for $J$ = 7 Hz) of **1-(4-chlorobenzyl)-4,4-diphenyl-1,4-azasilinane (3ha)**.

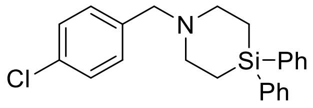


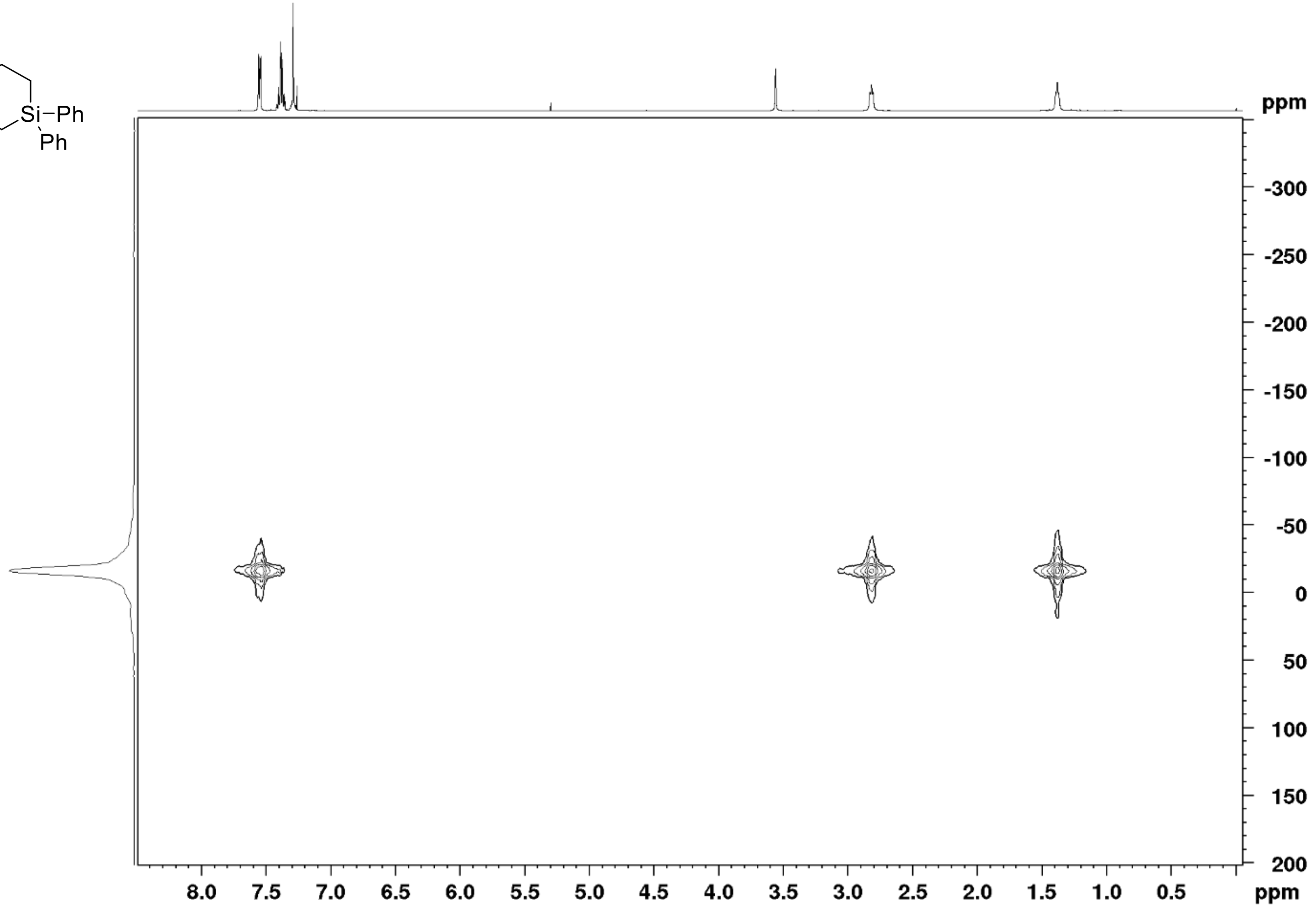

**Figure S30.** $^1$H NMR spectrum (500 MHz, $CDCl_3$, 298 K) of **1-(4-bromobenzyl)-4,4-diphenyl-1,4-azasilinane (3ia)**.

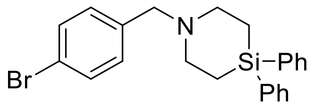

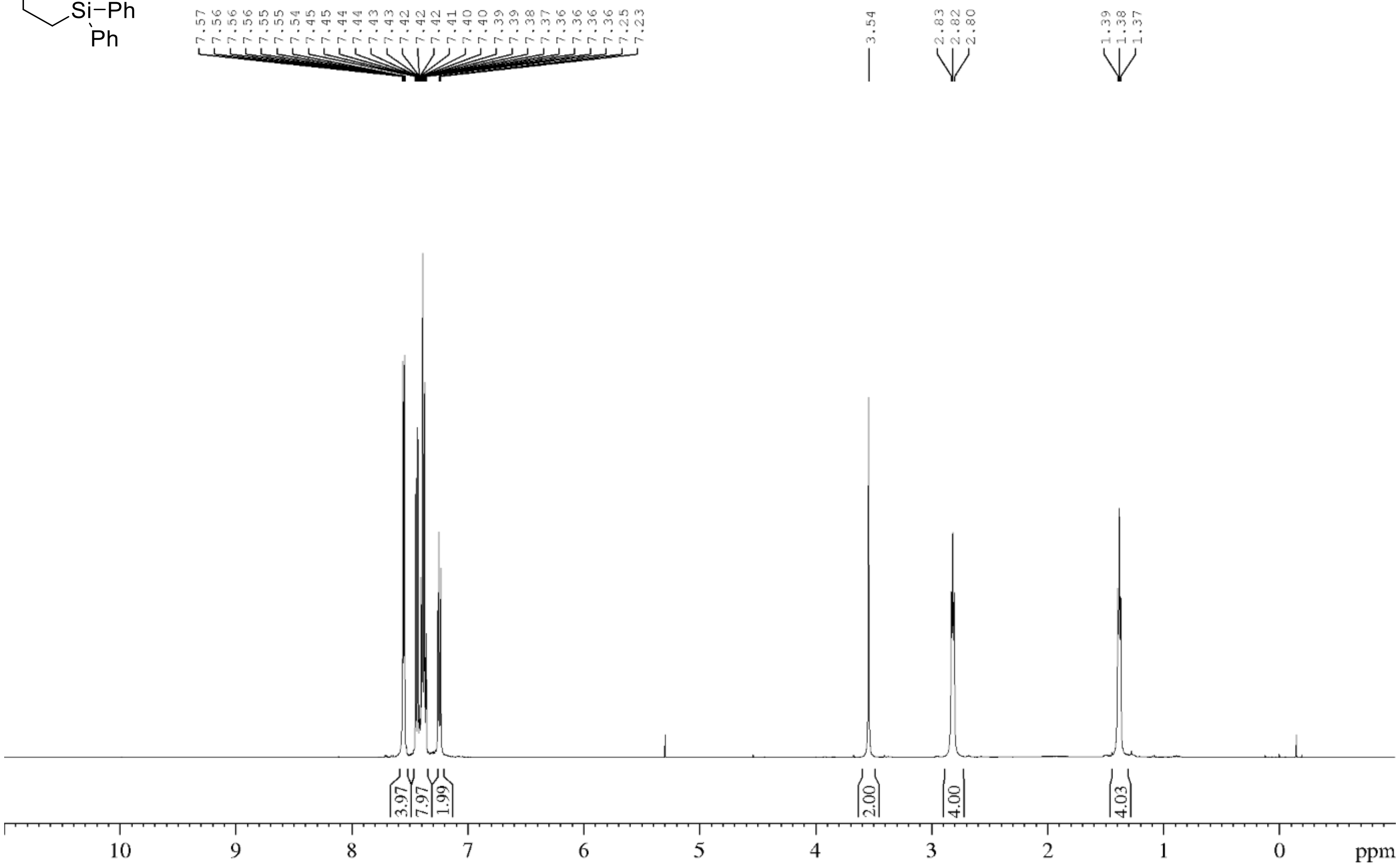

**Figure S31.** $^{13}$C NMR spectrum (125 MHz, $CDCl_3$, 298 K) of **1-(4-bromobenzyl)-4,4-diphenyl-1,4-azasilinane (3ia)**.

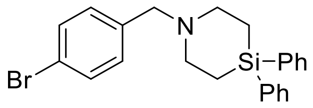


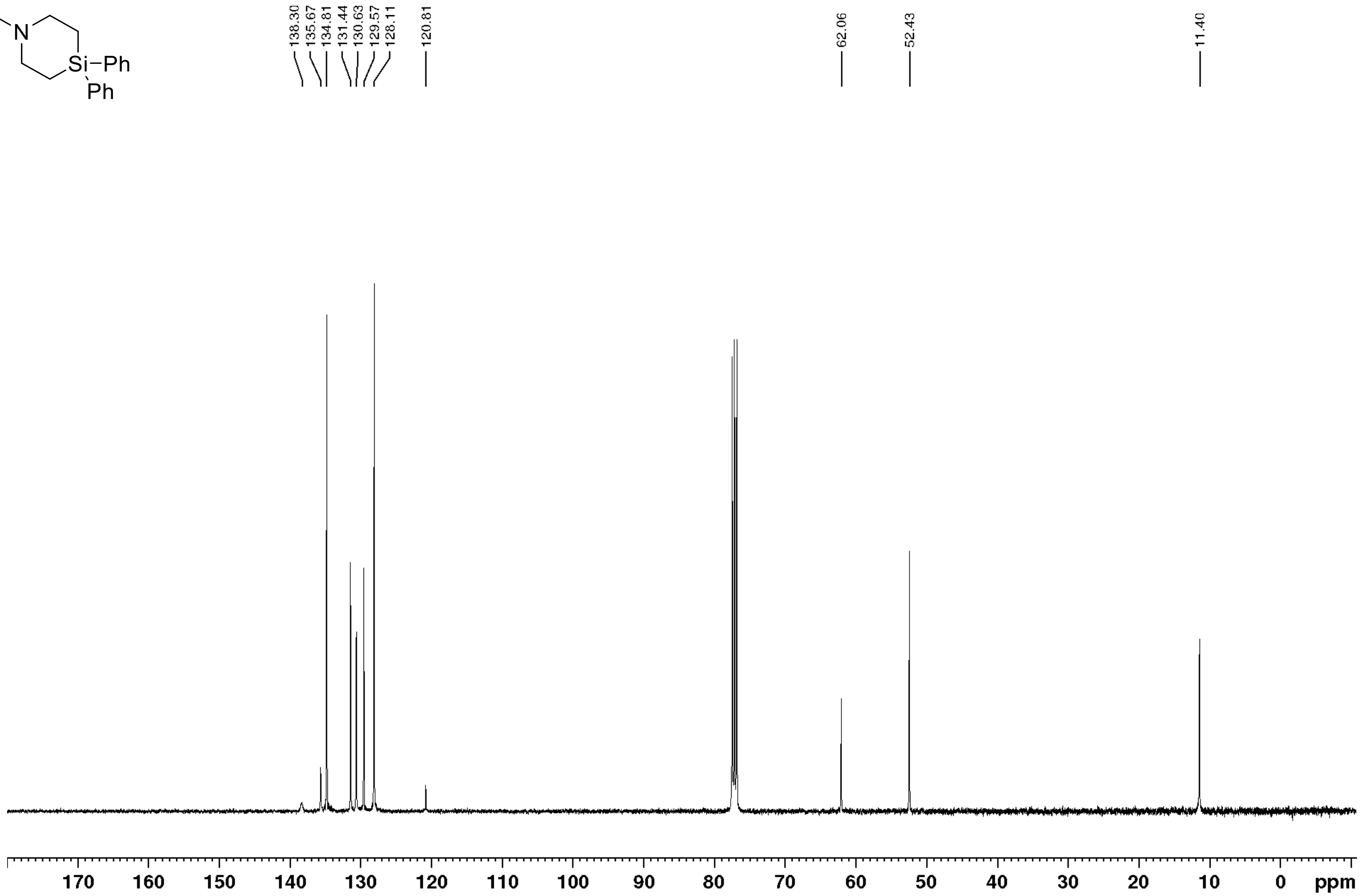

**Figure S32.** $^{1}H/^{29}Si$ HMQC NMR (500/99 MHz, $CDCl_3$, 298 K, optimized for $J$ = 7 Hz) of **1-(4-bromobenzyl)-4,4-diphenyl-1,4-azasilinane (3ia)**.

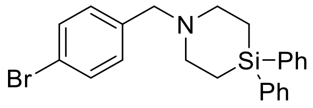


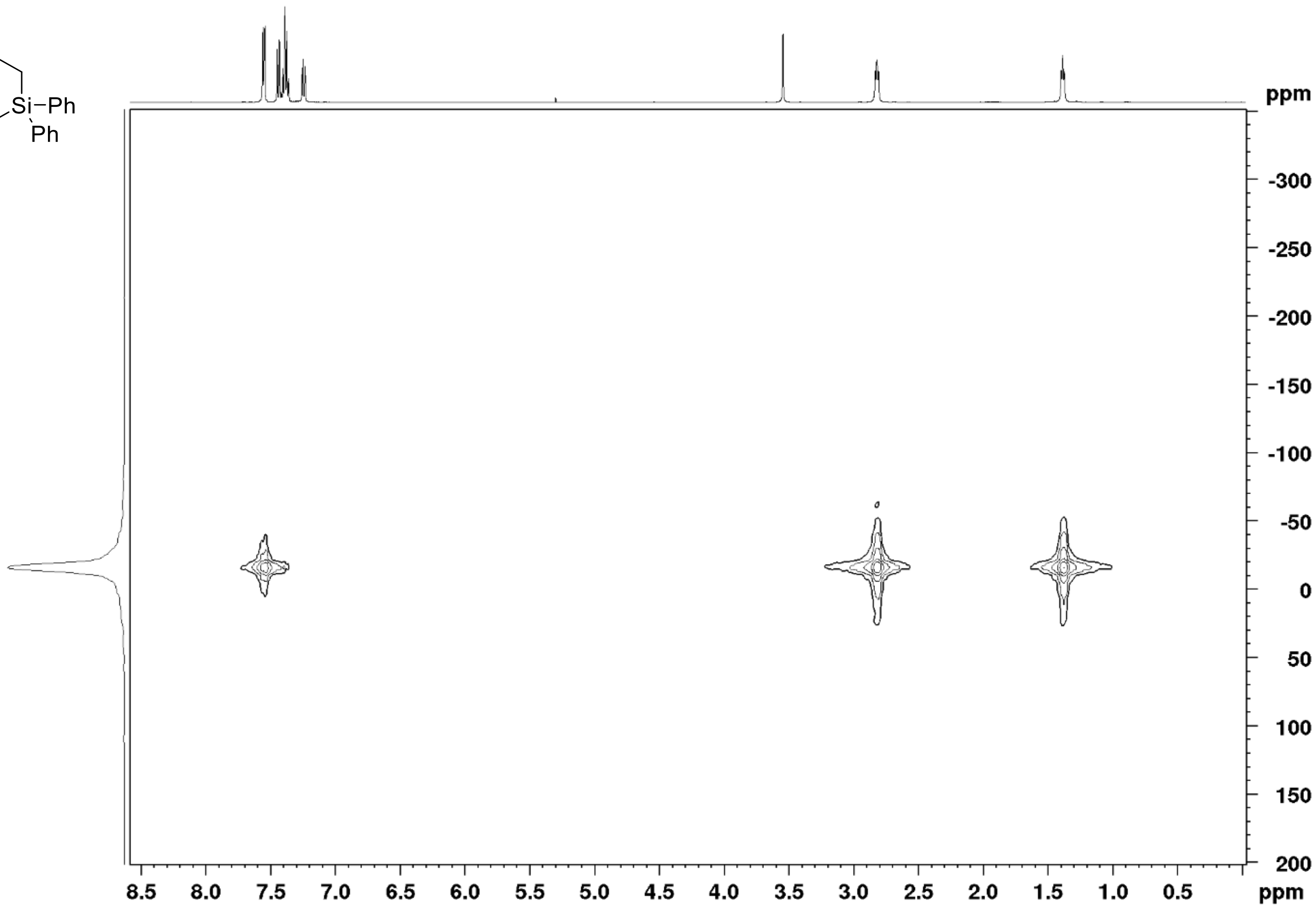

**Figure S33.** $^1$H NMR spectrum (500 MHz, $CDCl_3$, 298 K) of **1-(4-iodobenzyl)-4,4-diphenyl-1,4-azasilinane (3ja)**.

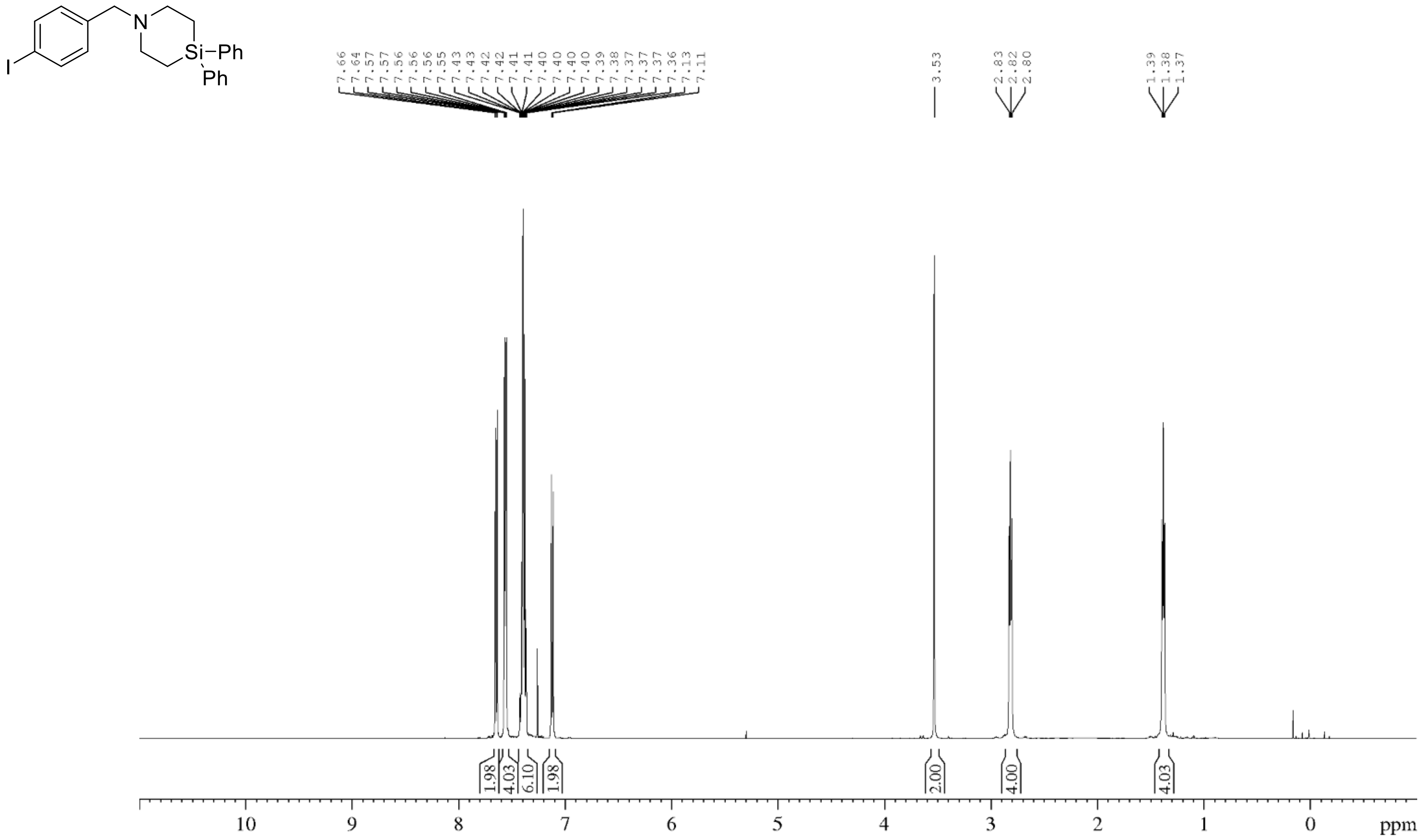

**Figure S34.** $^{13}C$ NMR spectrum (125 MHz, $CDCl_3$, 298 K) of **1-(4-iodobenzyl)-4,4-diphenyl-1,4-azasilinane (3ja)**.

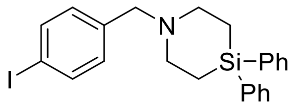


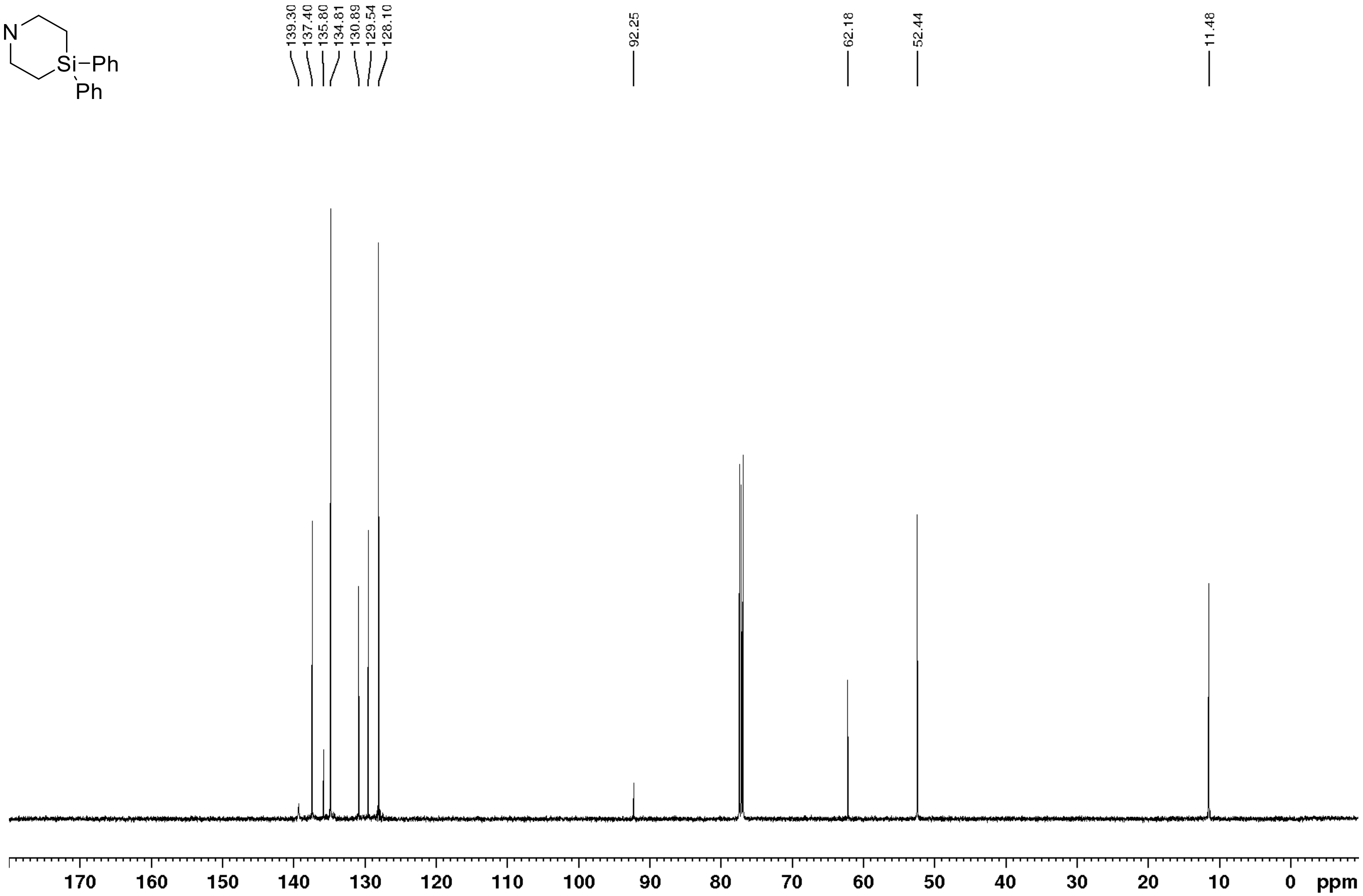

**Figure S35.** $^{1}H/^{29}Si$ HMQC NMR (500/99 MHz, $CDCl_3$, 298 K, optimized for $J$ = 7 Hz) of **1-(4-iodobenzyl)-4,4-diphenyl-1,4-azasilinane (3ja)**.

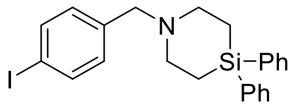


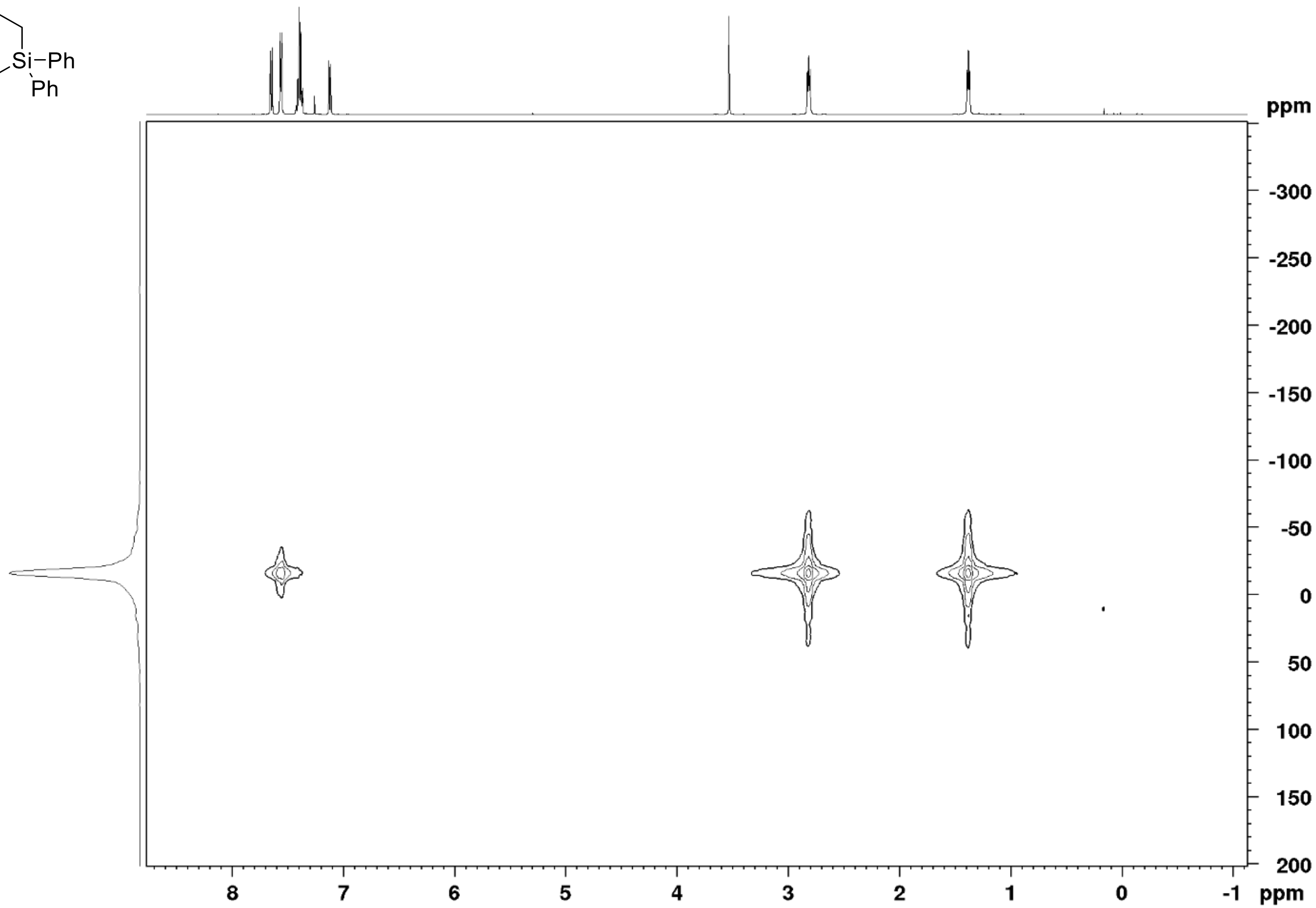

**Figure S36.** $^1$H NMR spectrum (500 MHz, $CDCl_3$, 298 K) of **4,4-diphenyl-1-(4-(trifluoromethyl)benzyl)-1,4-azasilinane (3ka)**.

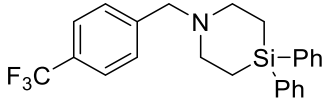


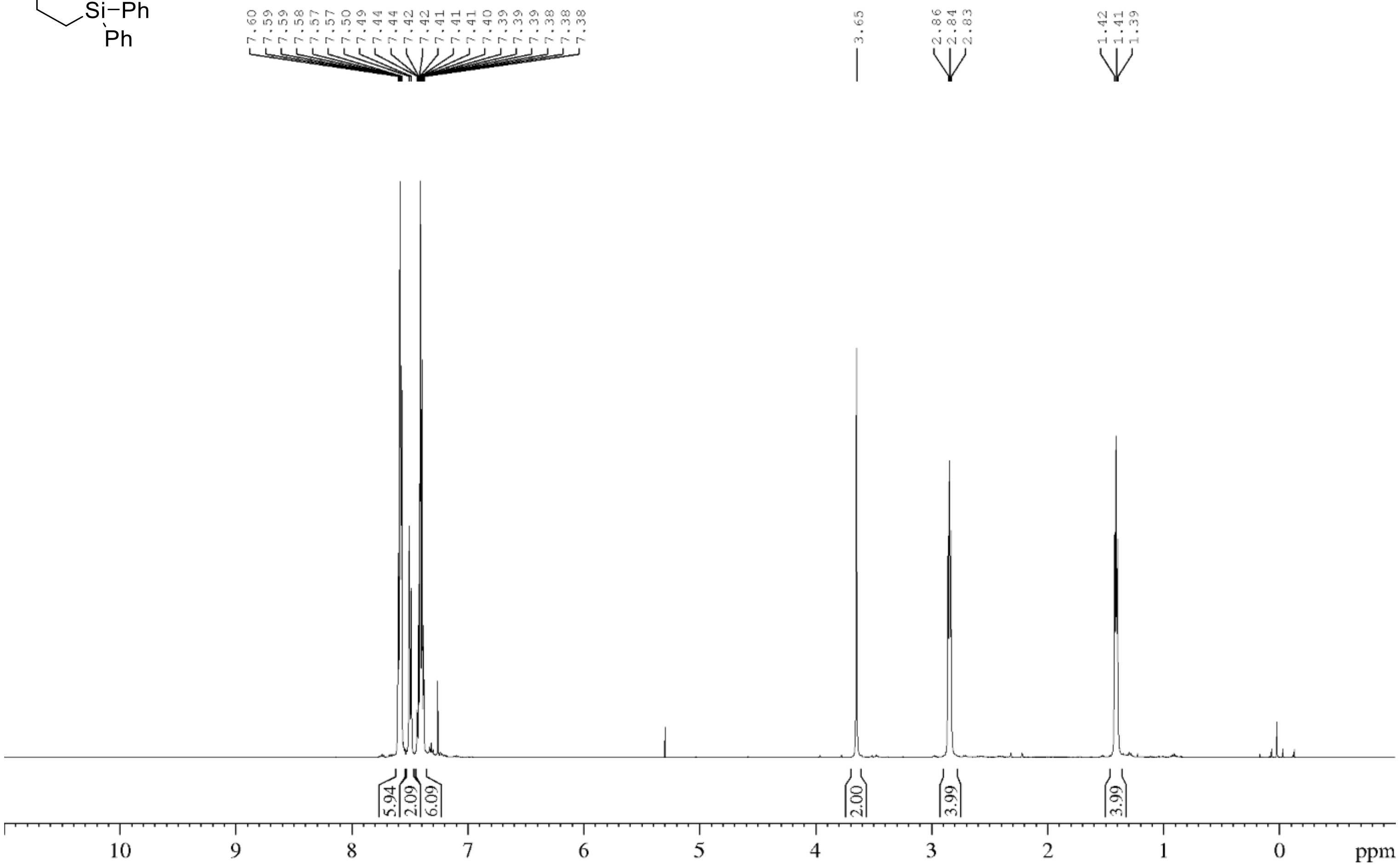

**Figure S37.** $^{13}$C NMR spectrum (125 MHz, $CDCl_3$, 298 K) of **4,4-diphenyl-1-(4-(trifluoromethyl)benzyl)-1,4-azasilinane (3ka)**.

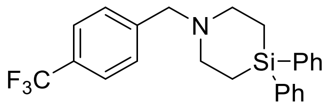


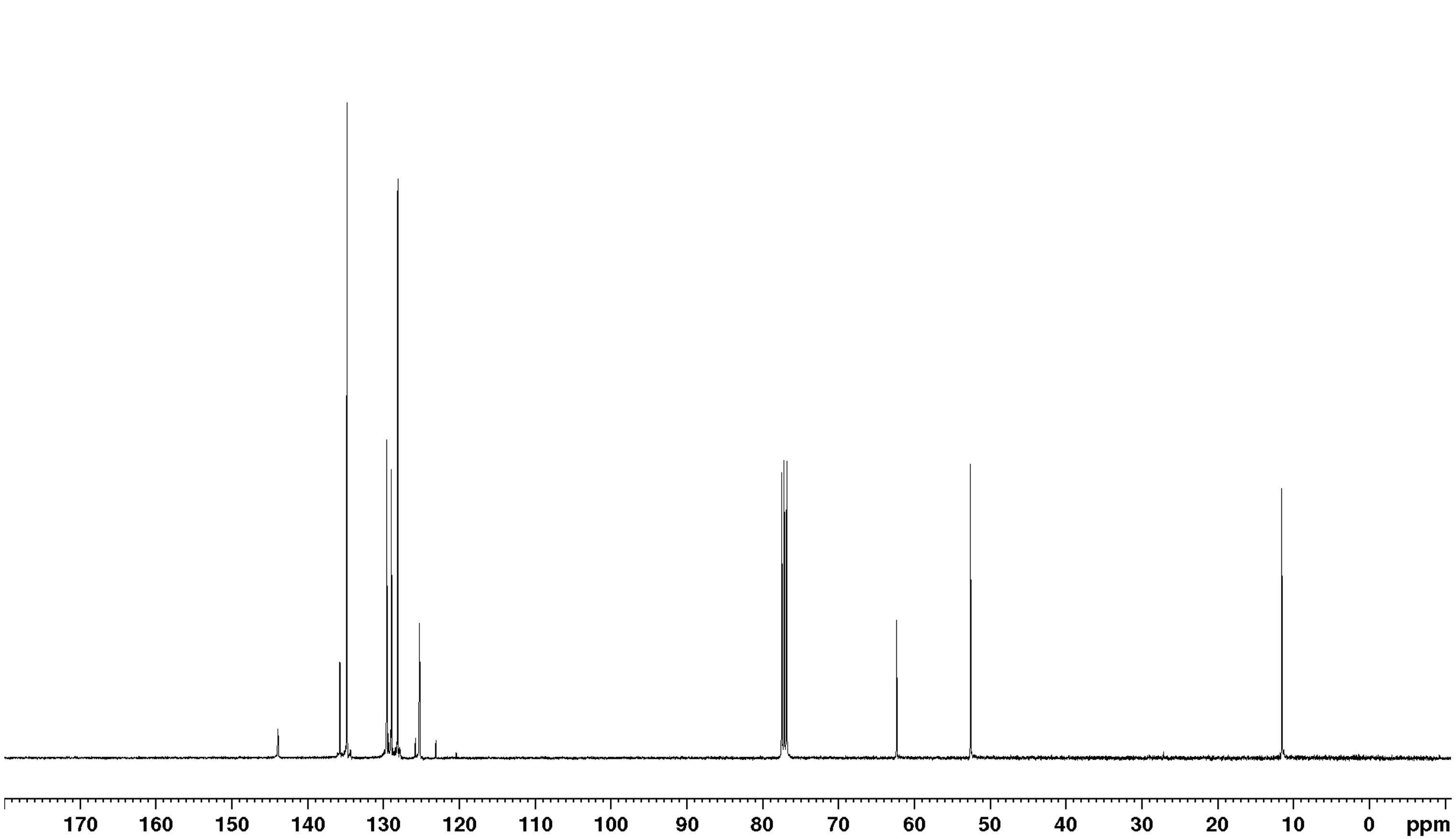

**Figure S38.** $^{19}$F NMR spectrum (471 MHz, $CDCl_3$, 298 K) of **4,4-diphenyl-1-(4-(trifluoromethyl)benzyl)-1,4-azasilinane (3ka)**.

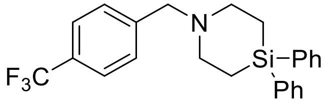

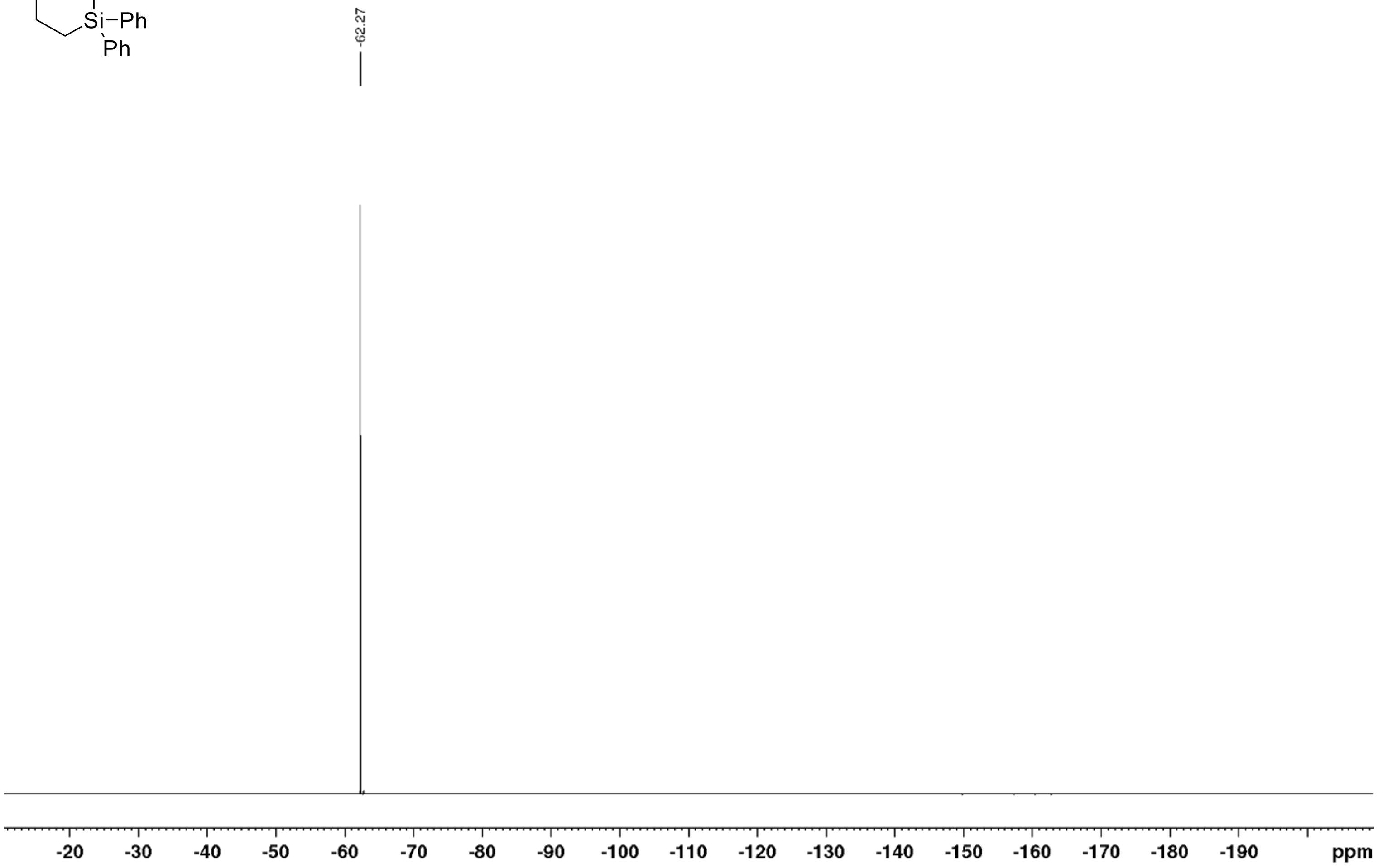

**Figure S39.** $^{1}H/^{29}Si$ HMQC NMR (500/99 MHz, $CDCl_3$, 298 K, optimized for $J$ = 7 Hz) of **4,4-diphenyl-1-(4-(trifluoromethyl)benzyl)-1,4-azasilinane (3ka)**.

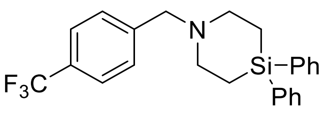


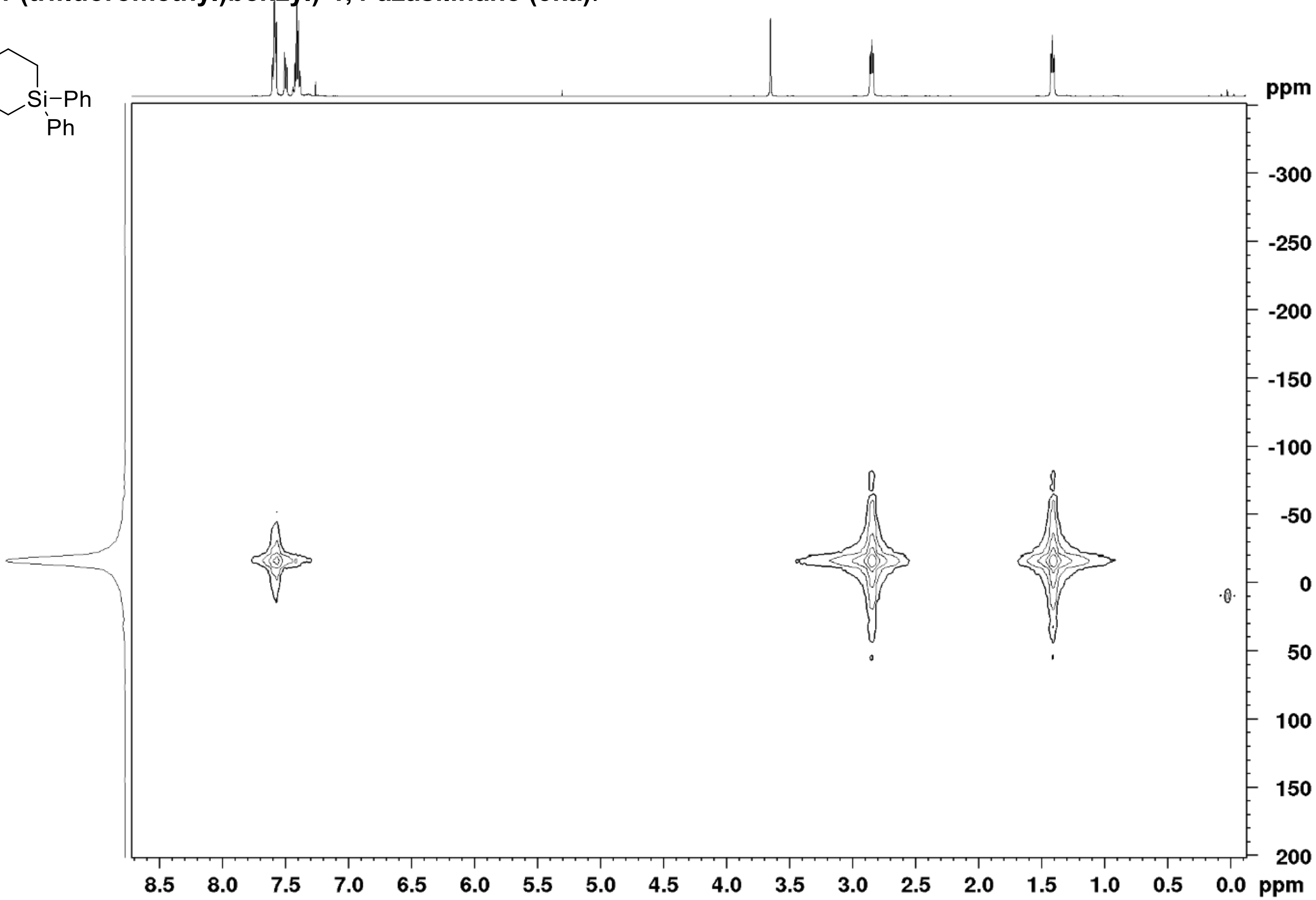

**Figure S40.** $^{1}H$ NMR spectrum (500 MHz, $CDCl_3$, 298 K) of **3-((4,4-diphenyl-1,4-azasilinan-1-yl)methyl)phenol (3la)**.

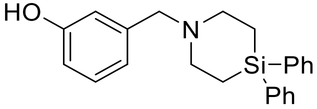


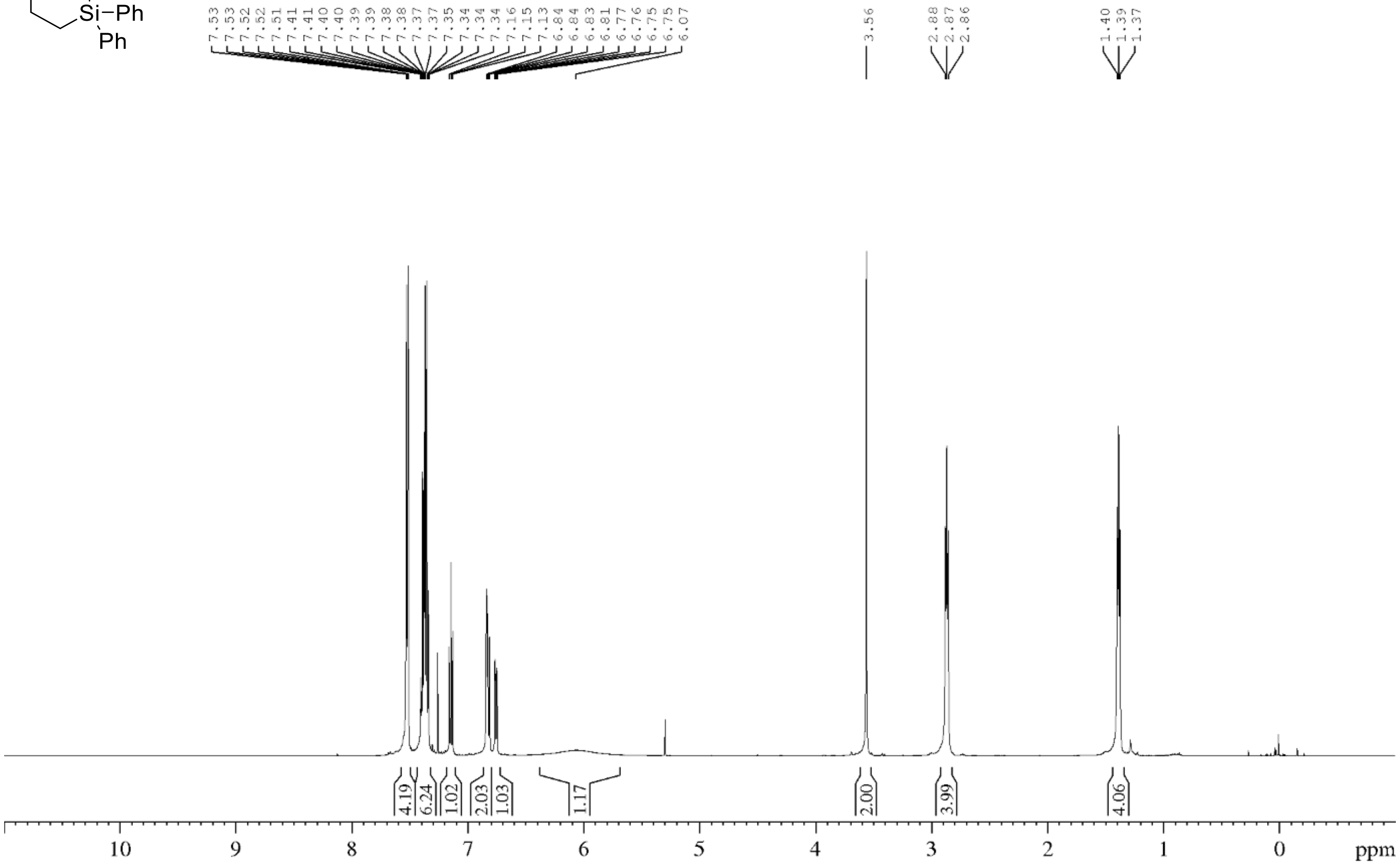

**Figure S41.** $^{13}$C NMR spectrum (125 MHz, $CDCl_3$, 298 K) of **3-((4,4-diphenyl-1,4-azasilinan-1-yl)methyl)phenol (3la)**.

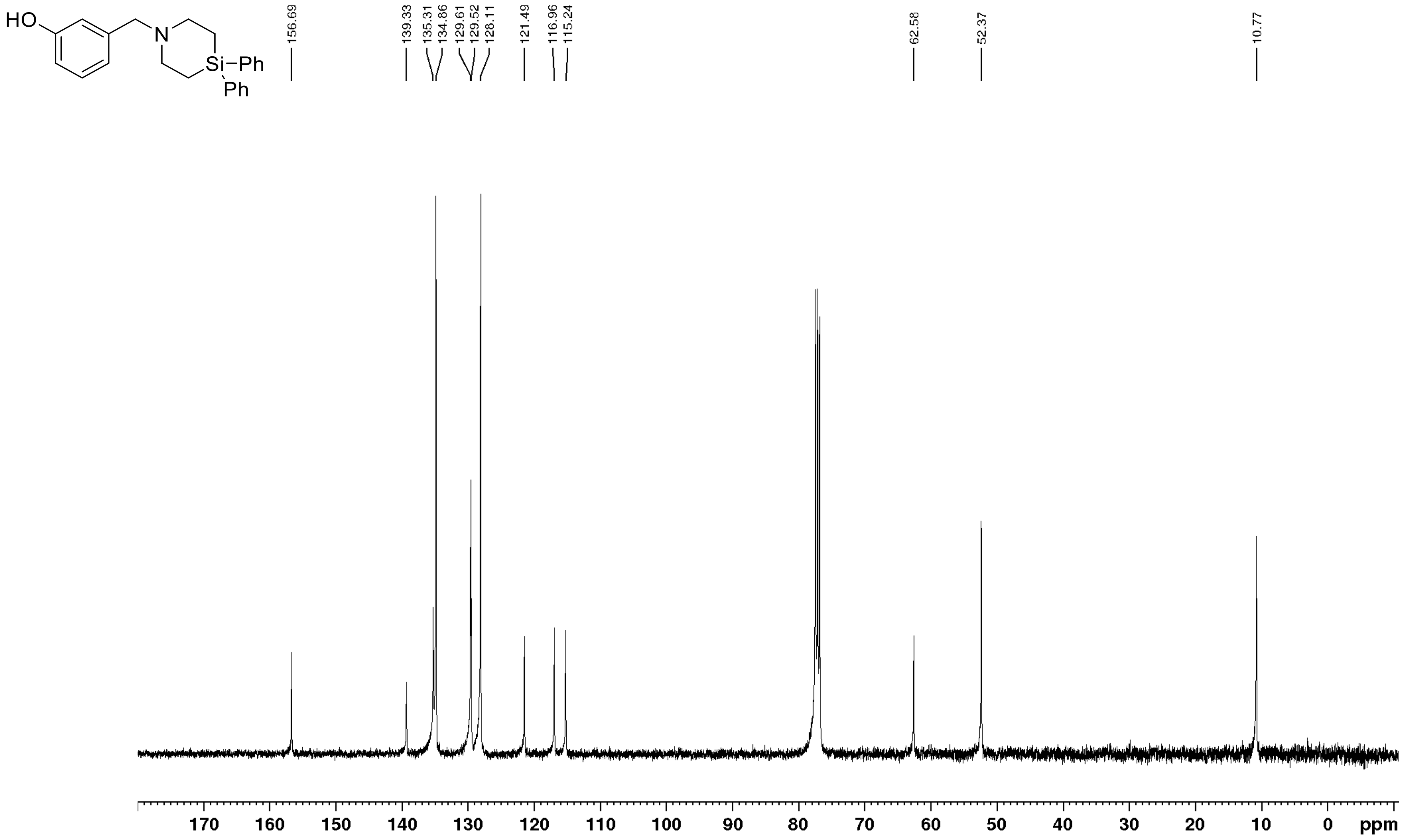

**Figure S42.** $^{1}H/^{29}Si$ HMQC NMR (500/99 MHz, $CDCl_3$, 298 K, optimized for $J$ = 7 Hz) of **3-((4,4-diphenyl-1,4-azasilinan-1-yl)methyl)phenol (3la)**.

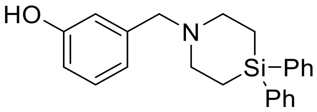


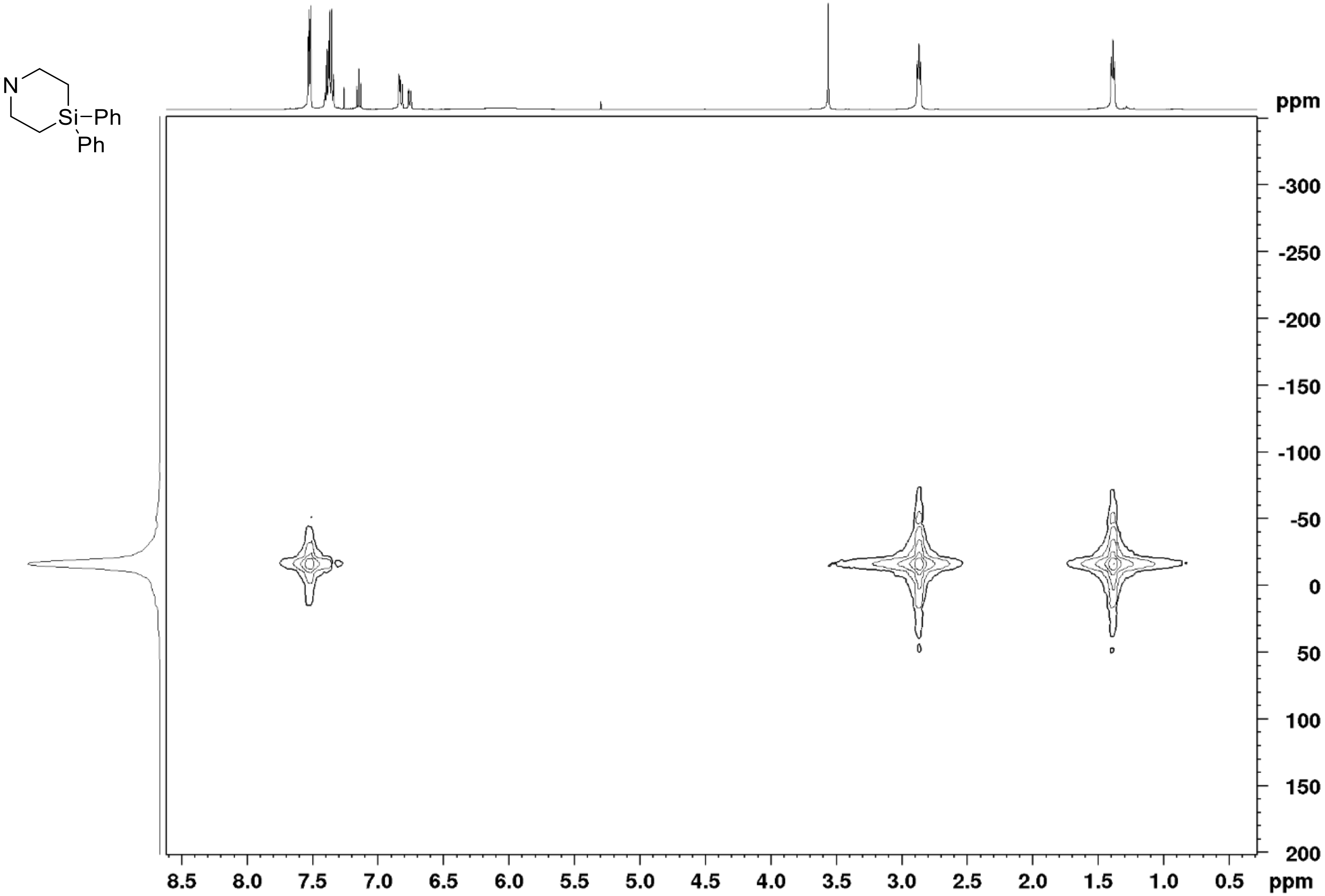

**Figure S43.** $^1H$ NMR spectrum (500 MHz, $CDCl_3$, 298 K) of **1-(naphthalen-2-ylmethyl)-4,4-diphenyl-1,4-azasilinane (3ma)**.

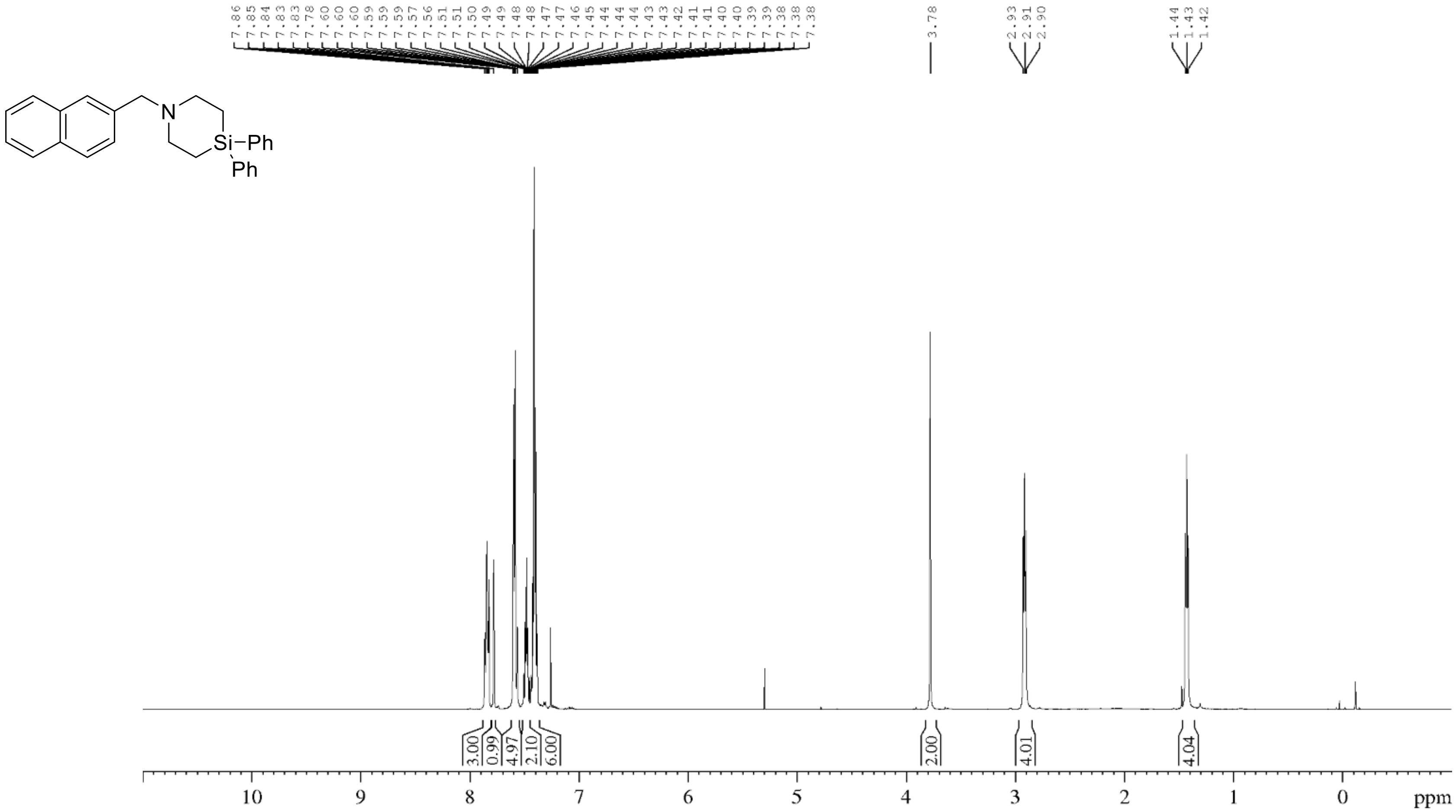

**Figure S44.** $^{13}C$ NMR spectrum (125 MHz, $CDCl_3$, 298 K) of **1-(naphthalen-2-ylmethyl)-4,4-diphenyl-1,4-azasilinane (3ma)**.

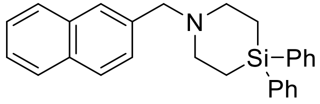


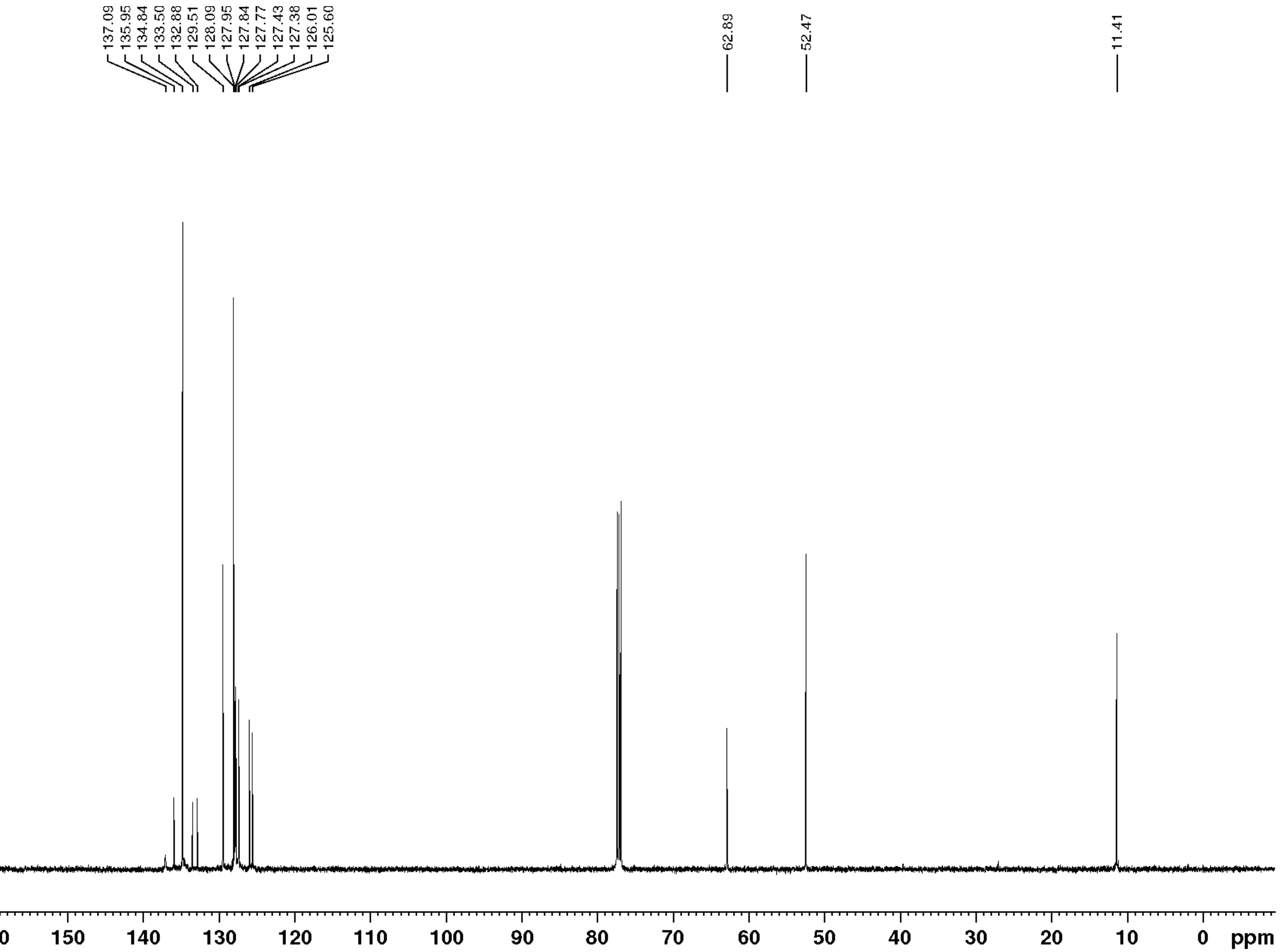

**Figure S45.** $^{1}H/^{29}Si$ HMQC NMR (500/99 MHz, $CDCl_3$, 298 K, optimized for $J$ = 7 Hz) of **1-(naphthalen-2-ylmethyl)-4,4-diphenyl-1,4-azasilinane (3ma)**.

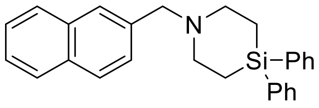


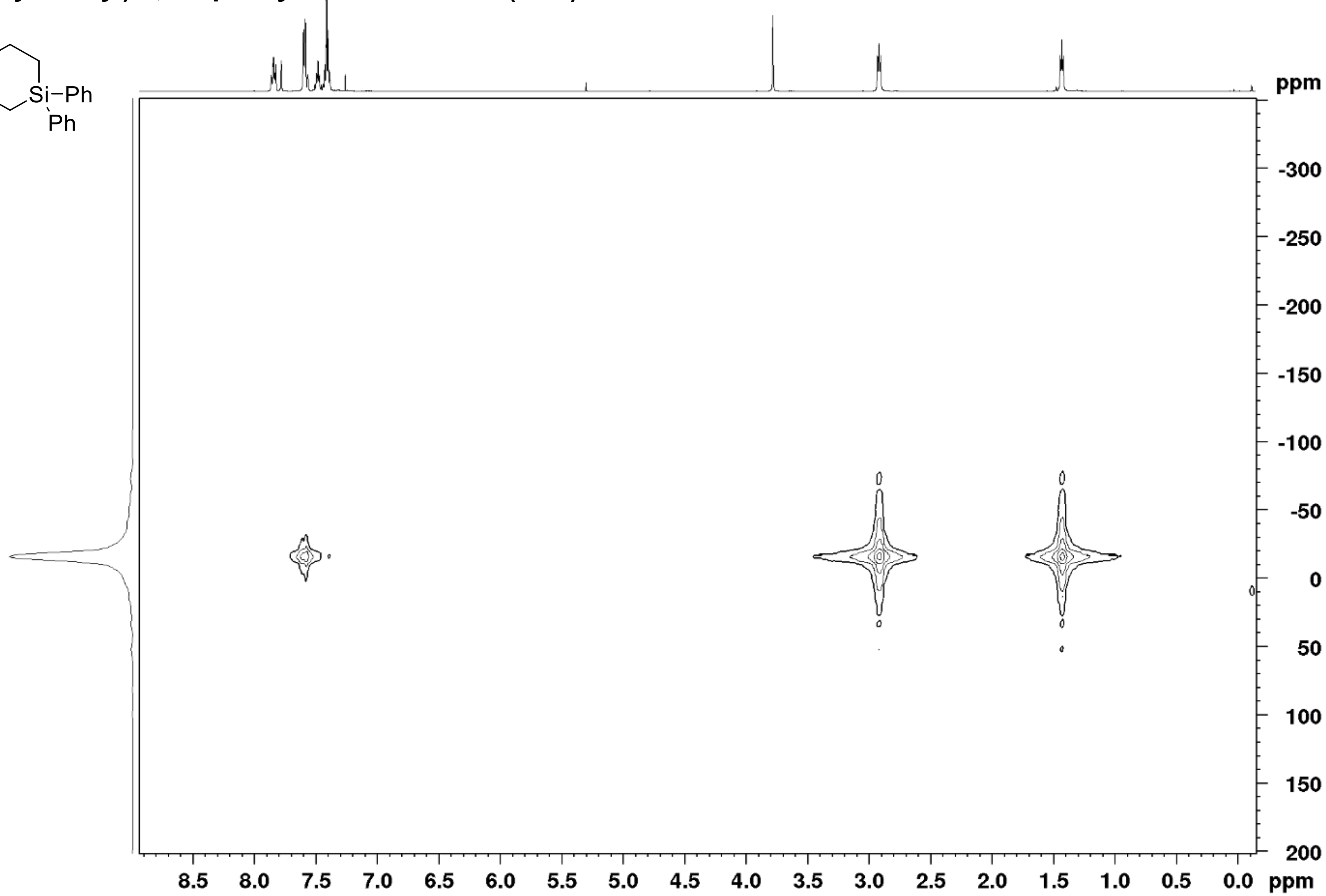

**Figure S46.** $^{1}$H NMR spectrum (500 MHz, $CDCl_3$, 298 K) of **1,4-bis((4,4-diphenyl-1,4-azasilinan-1-yl)methyl)benzene (3na)**.

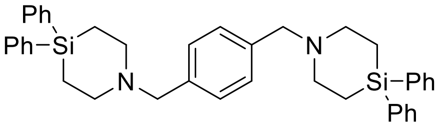

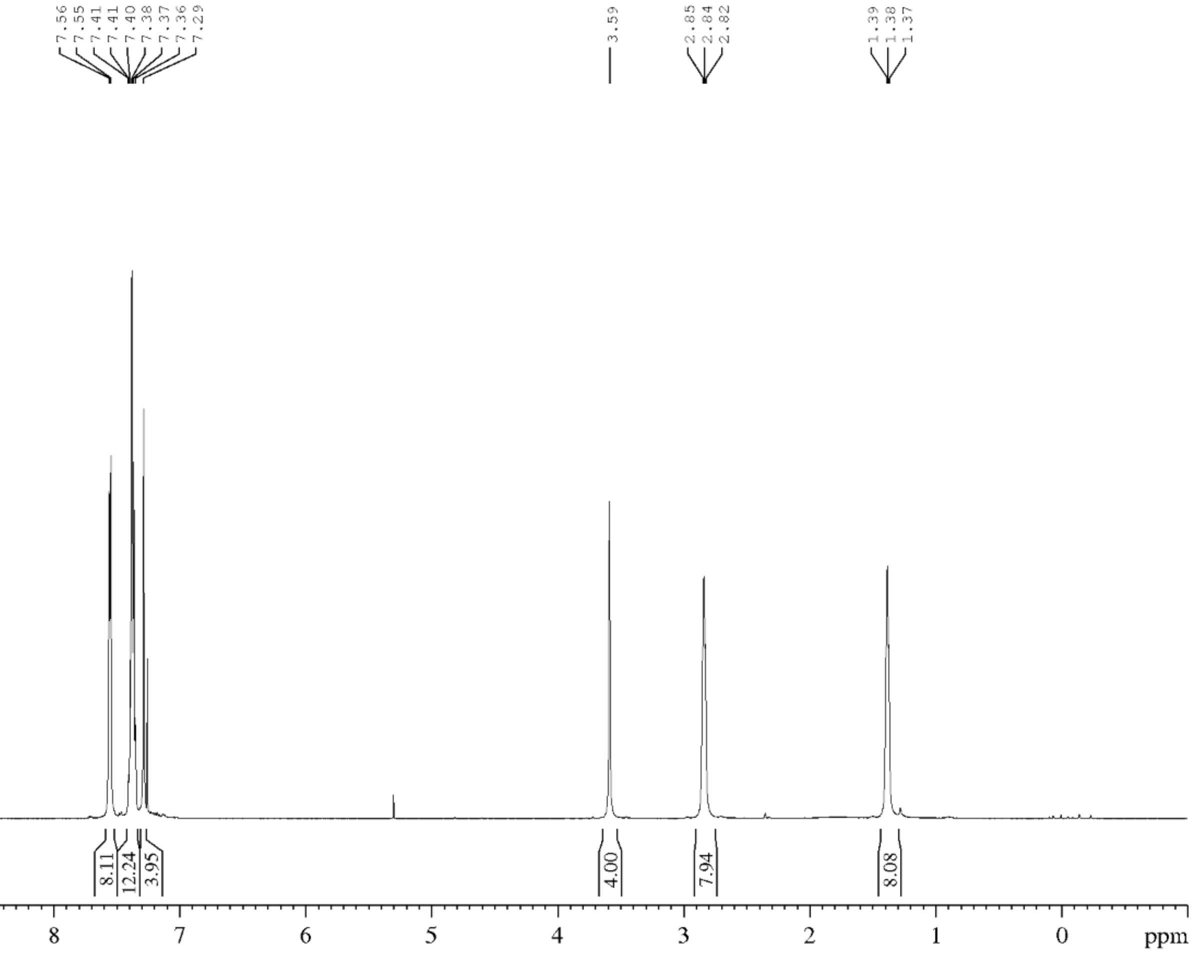

**Figure S47.** $^{13}C$ NMR spectrum (125 MHz, $CDCl_3$, 298 K) of **1,4-bis((4,4-diphenyl-1,4-azasilinan-1-yl)methyl)benzene (3na)**.

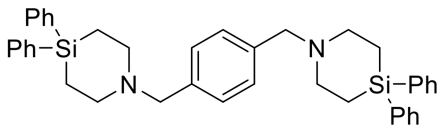

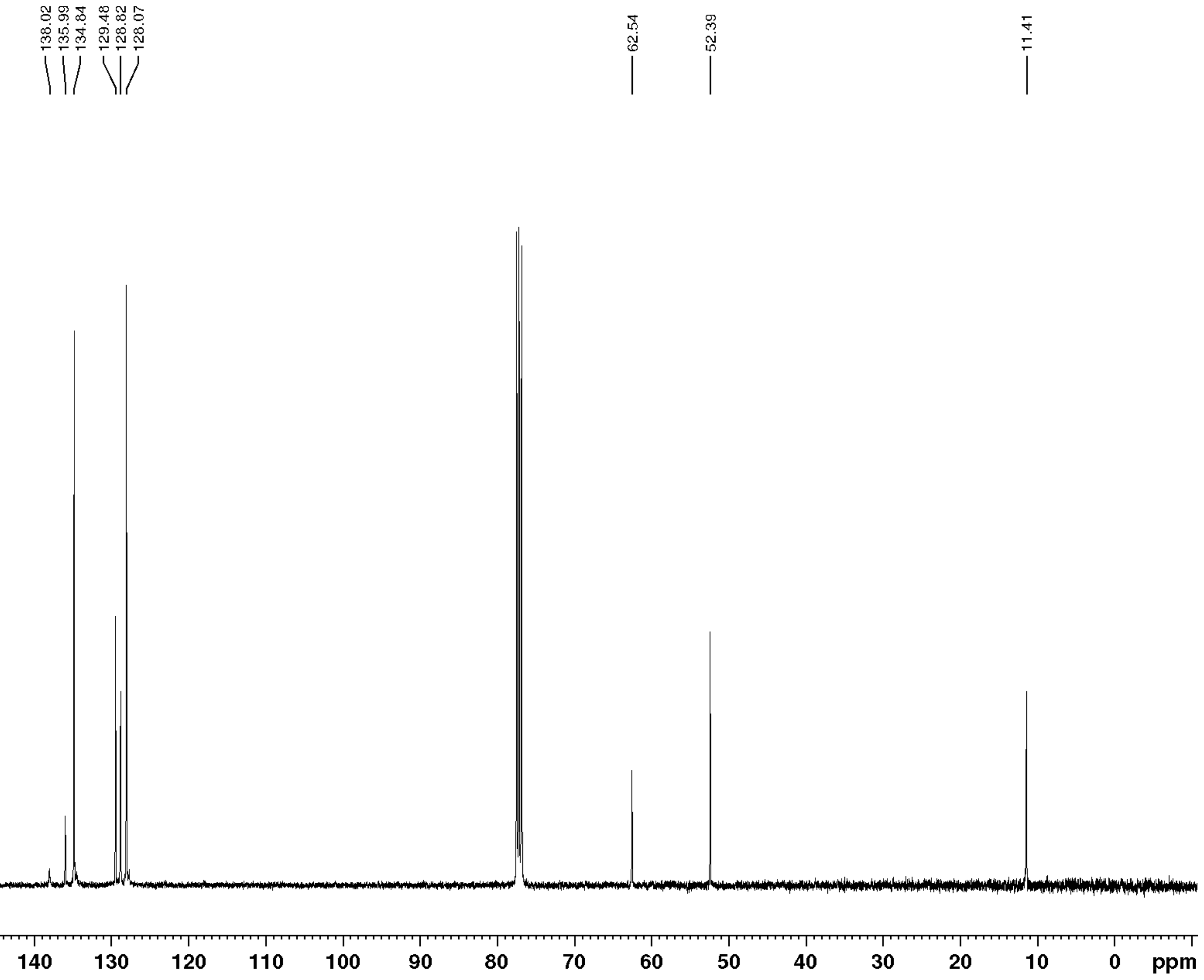

**Figure S48.** $^{1}H/^{29}Si$ HMQC NMR (500/99 MHz, $CDCl_3$, 298 K, optimized for $J$ = 7 Hz) of

**1,4-bis((4,4-diphenyl-1,4-azasilinan-1-yl)methyl)benzene (3na)**.

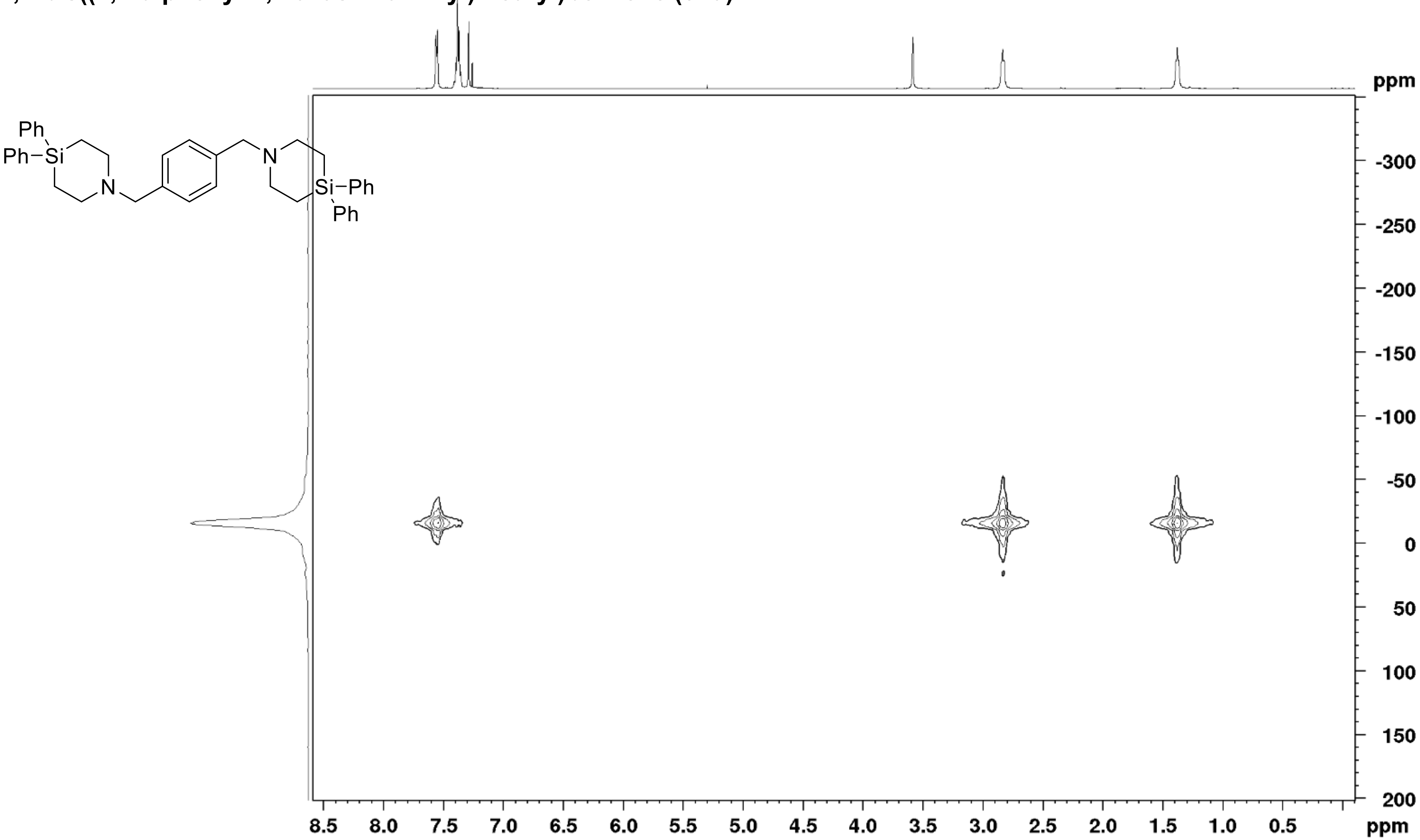

**Figure S49.** $^{1}$H NMR spectrum (500 MHz, $CDCl_3$, 298 K) of **1-cyclohexyl-4,4-diphenyl-1,4-azasilinane (3oa)**.

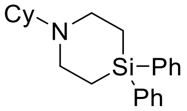

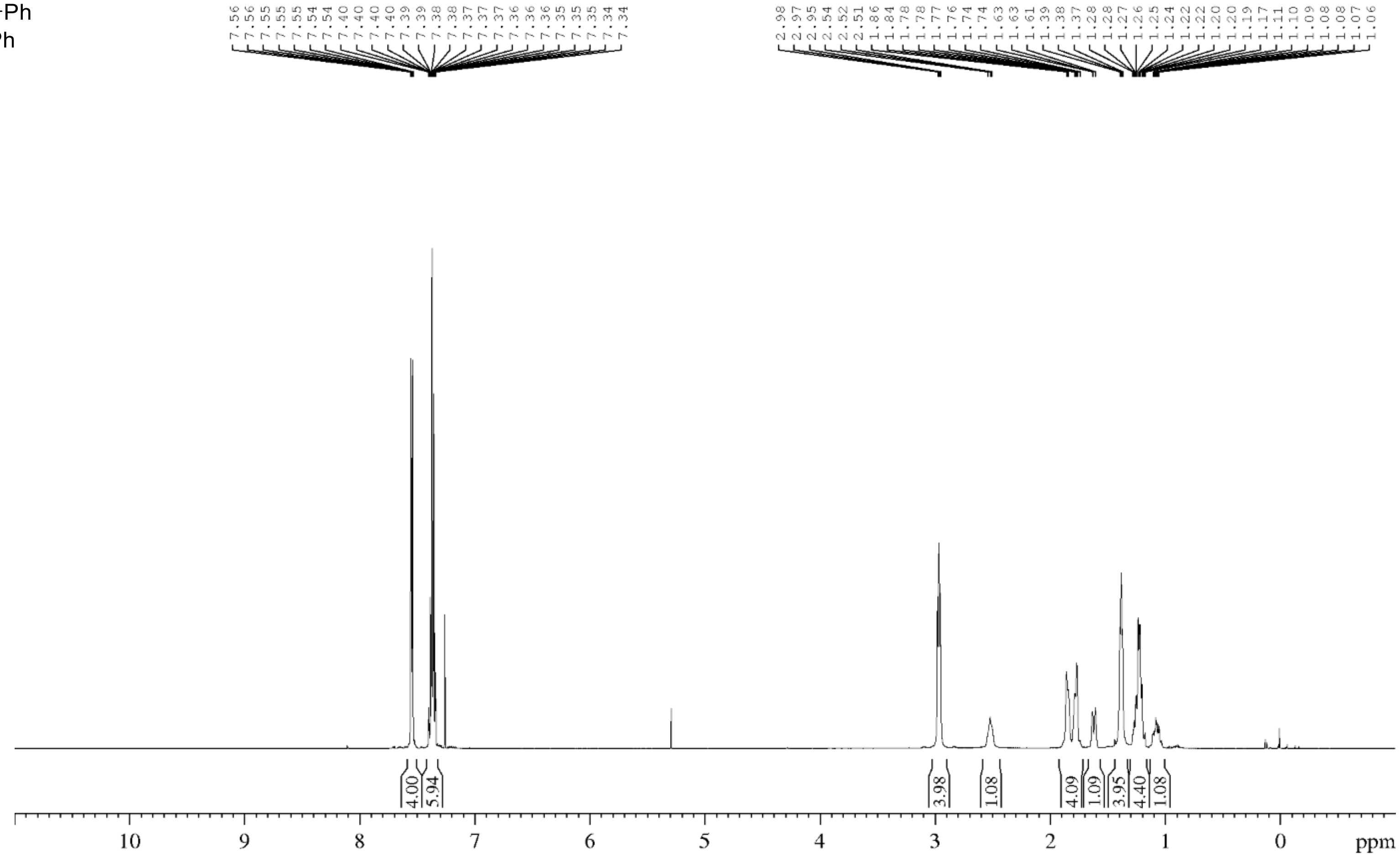

**Figure S50.** $^{13}C$ NMR spectrum (125 MHz, $CDCl_3$, 298 K) of **1-cyclohexyl-4,4-diphenyl-1,4-azasilinane (3oa)**.

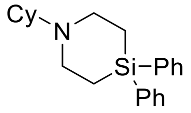


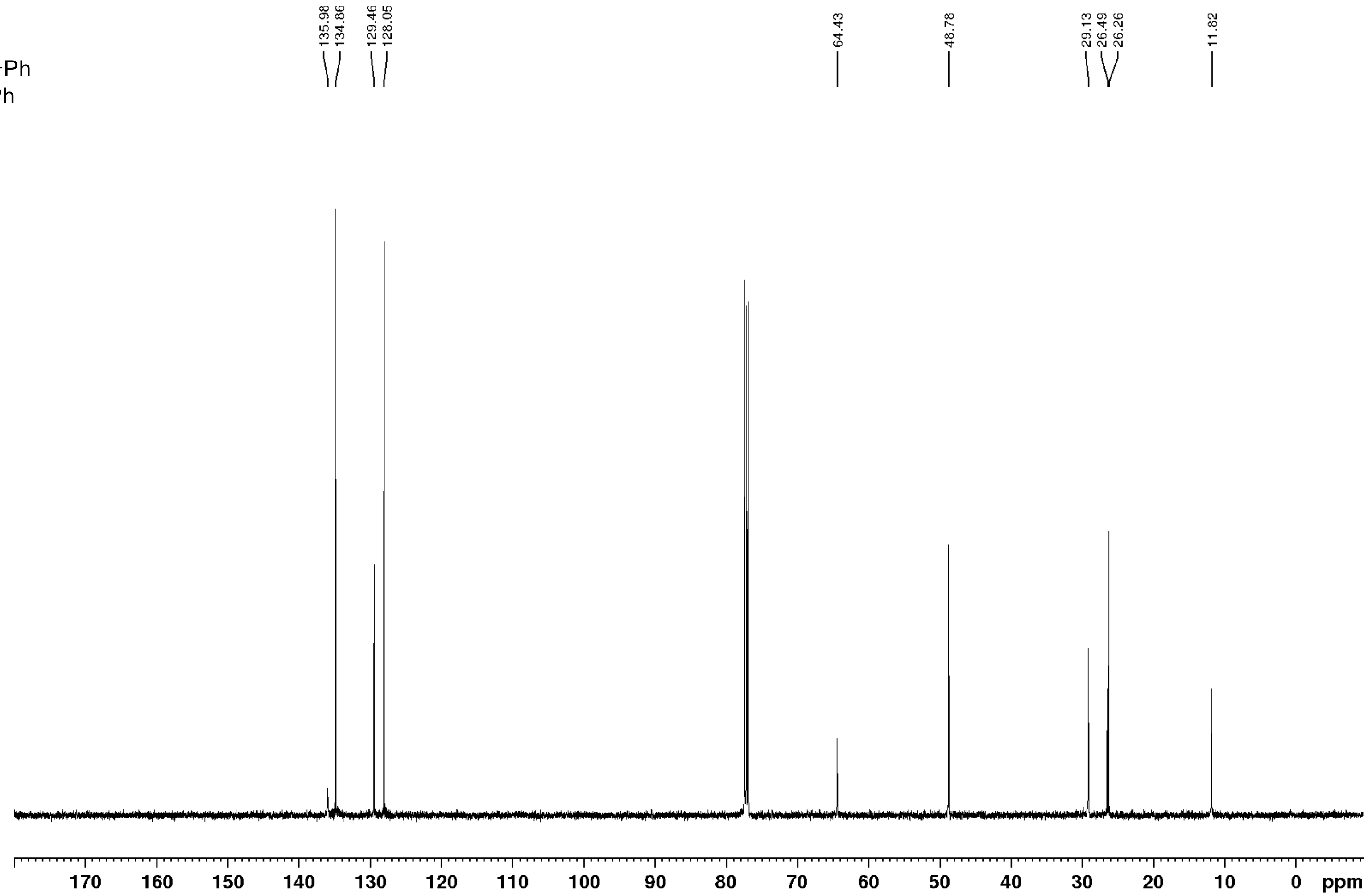

**Figure S51.** $^{1}H/^{29}Si$ HMQC NMR (500/99 MHz, $CDCl_3$, 298 K, optimized for $J$ = 7 Hz) of **1-cyclohexyl-4,4-diphenyl-1,4-azasilinane (3oa)**.

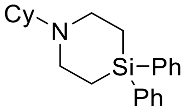


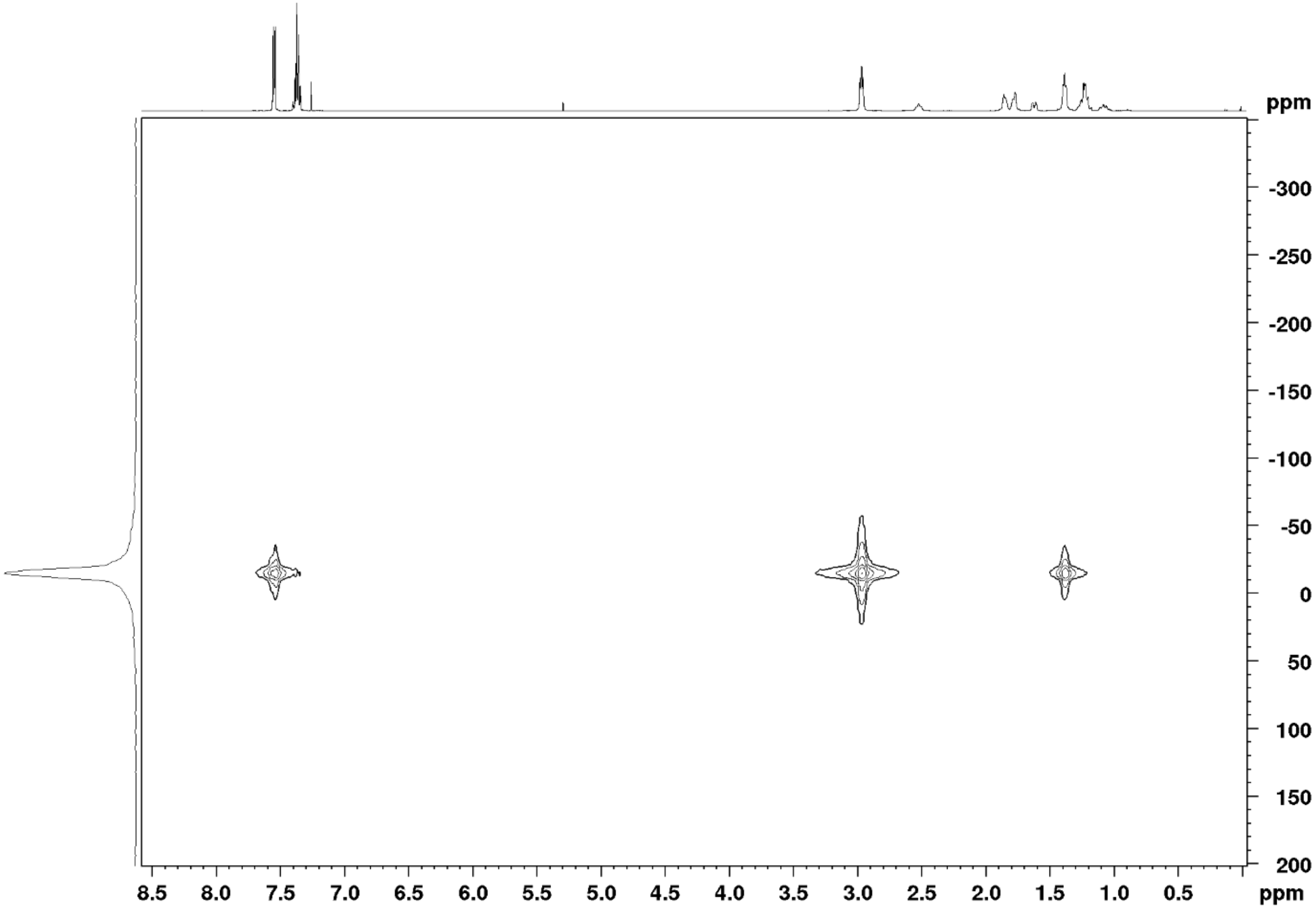

**Figure S52.** $^1$H NMR spectrum (500 MHz, $CDCl_3$, 298 K) of **1-ethyl-4,4-diphenyl-1,4-azasilinane (3pa)**.

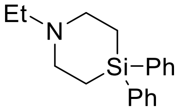


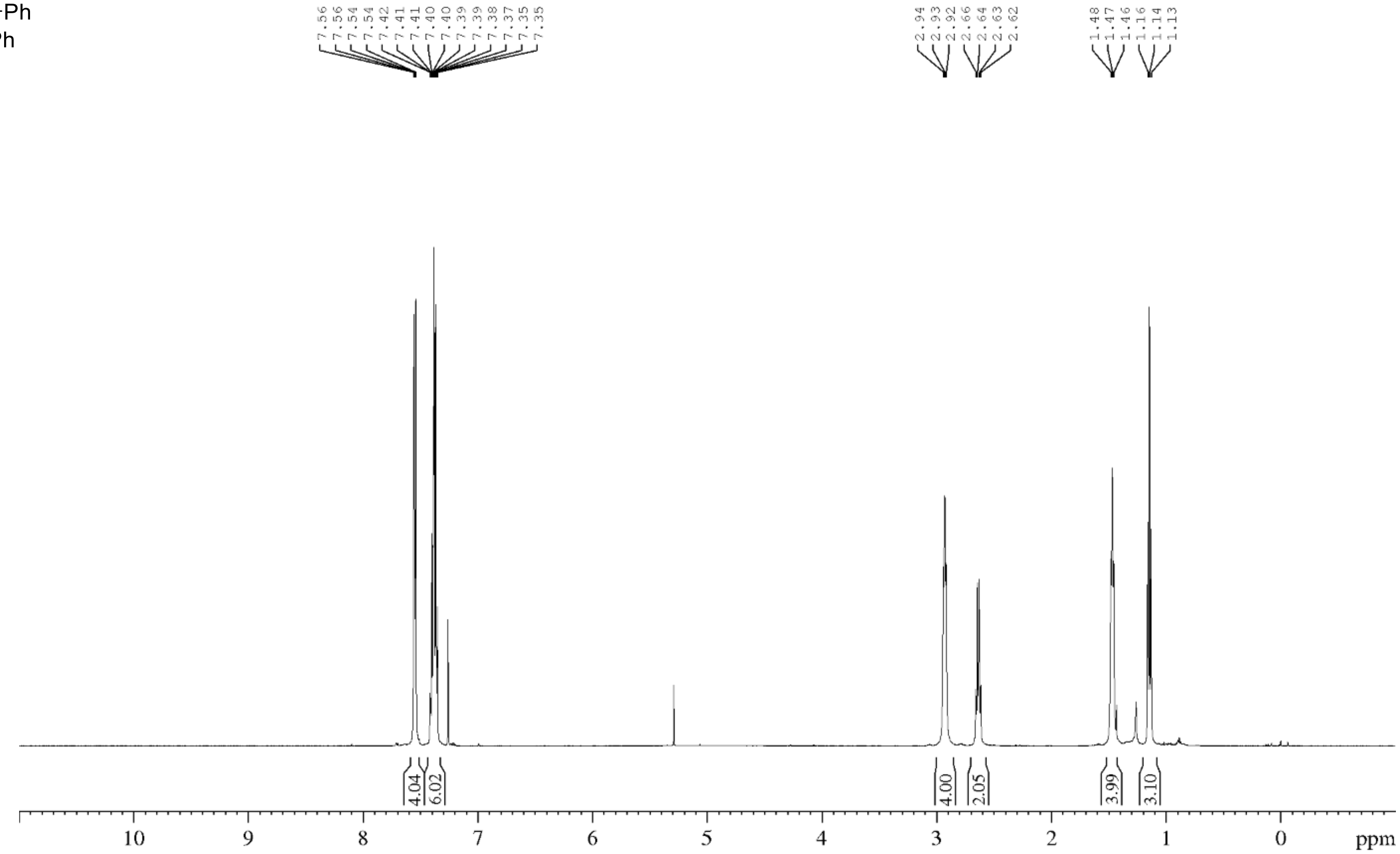

**Figure S53.** $^{13}$C NMR spectrum (125 MHz, $CDCl_3$, 298 K) of **1-ethyl-4,4-diphenyl-1,4-azasilinane (3pa)**.

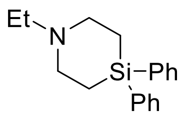


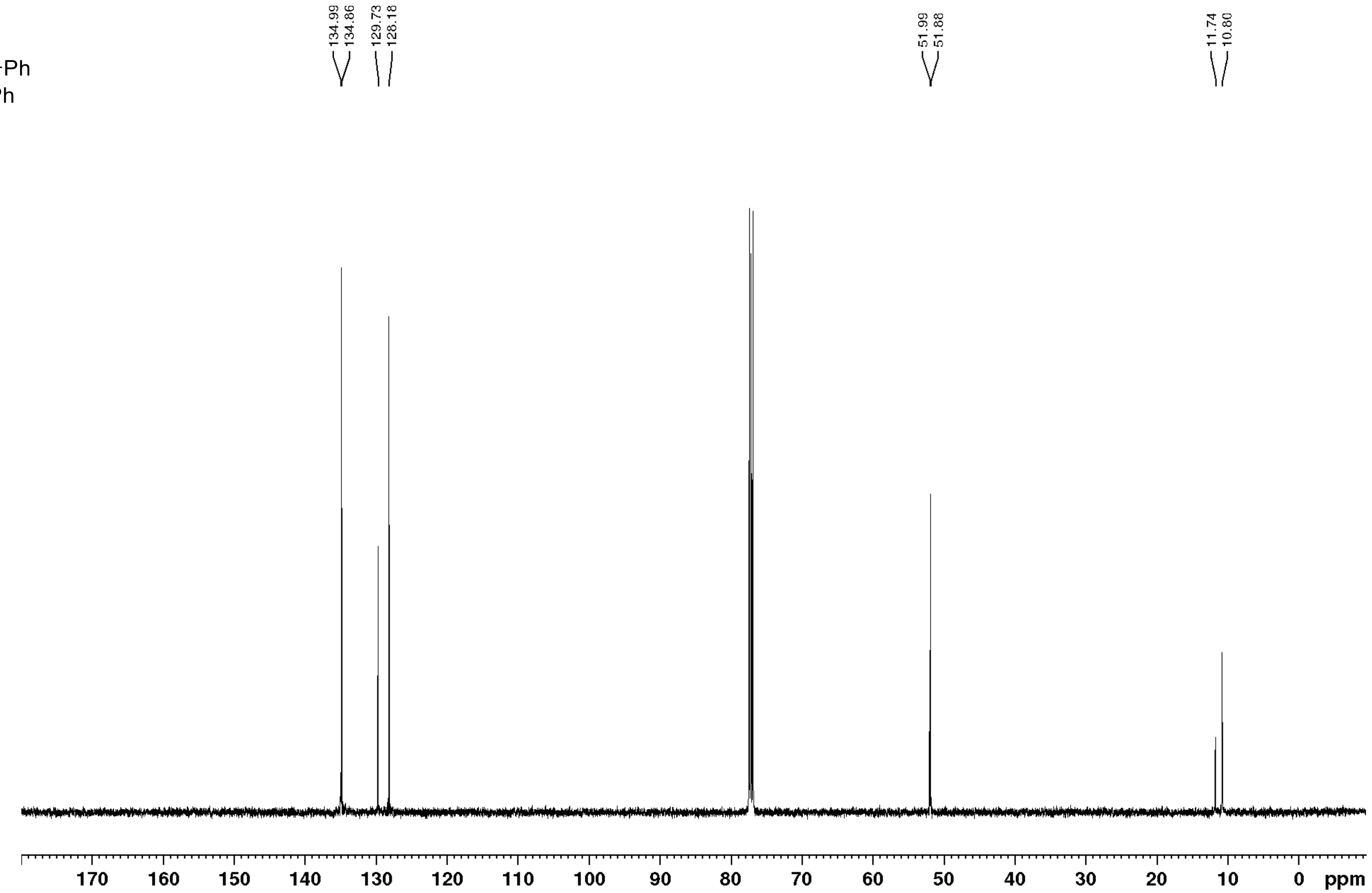

**Figure S54.** $^{1}H/^{29}Si$ HMQC NMR (500/99 MHz, $CDCl_3$, 298 K, optimized for $J$ = 7 Hz) of **1-ethyl-4,4-diphenyl-1,4-azasilinane (3pa)**.

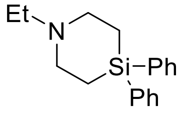


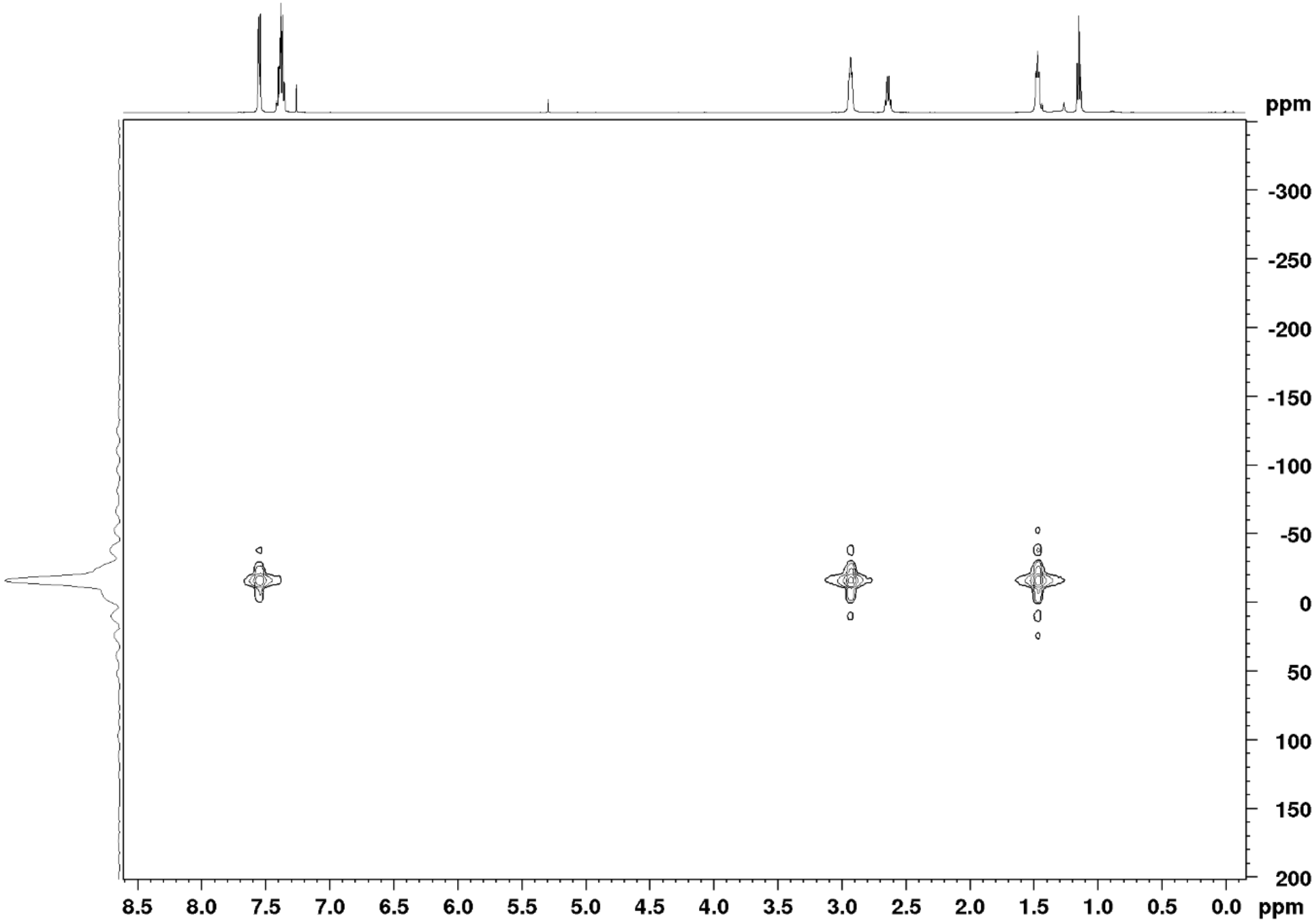

**Figure S55.** $^1$H NMR spectrum (500 MHz, $CDCl_3$, 298 K) of **1-methyl-4,4-diphenyl-1,4-azasilinane (3qa)**.

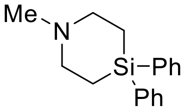


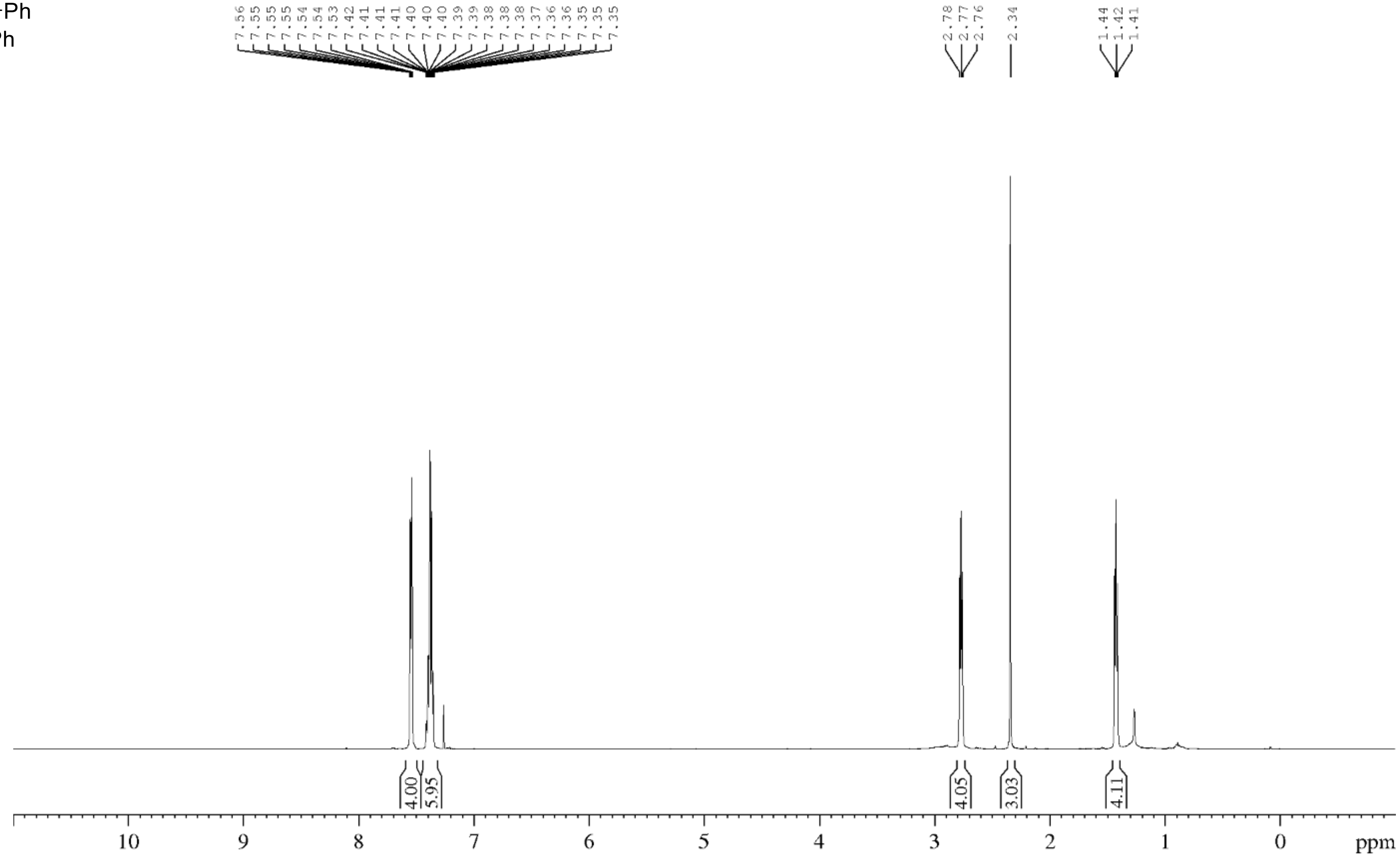

**Figure S56.** $^{13}C$ NMR spectrum (125 MHz, $CDCl_3$, 298 K) of **1-methyl-4,4-diphenyl-1,4-azasilinane (3qa)**.

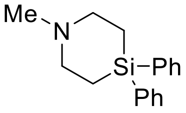


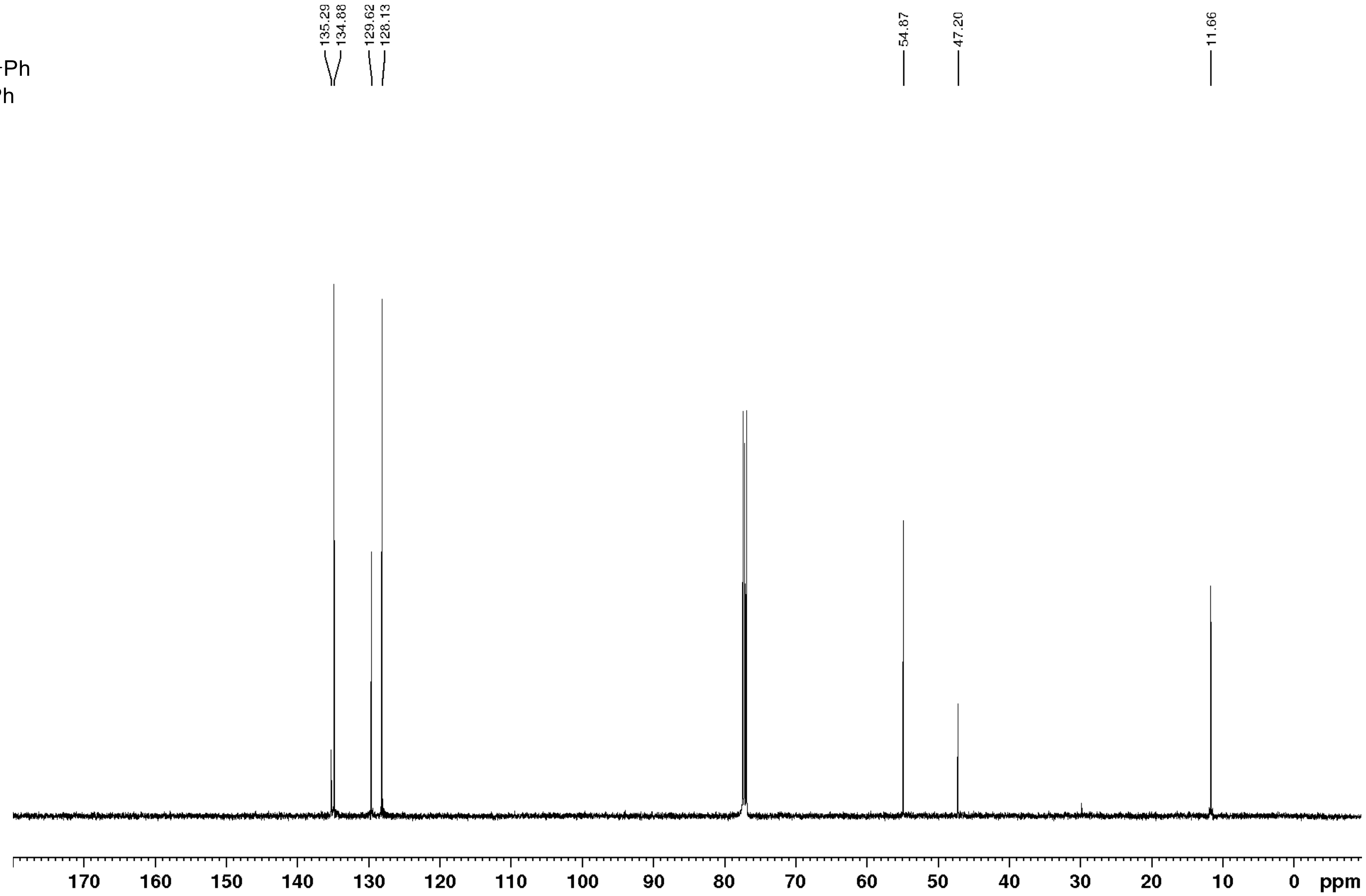

**Figure S57.** $^{1}H/^{29}Si$ HMQC NMR (500/99 MHz, $CDCl_3$, 298 K, optimized for $J$ = 7 Hz) of **1-methyl-4,4-diphenyl-1,4-azasilinane (3qa)**.

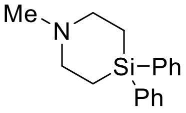


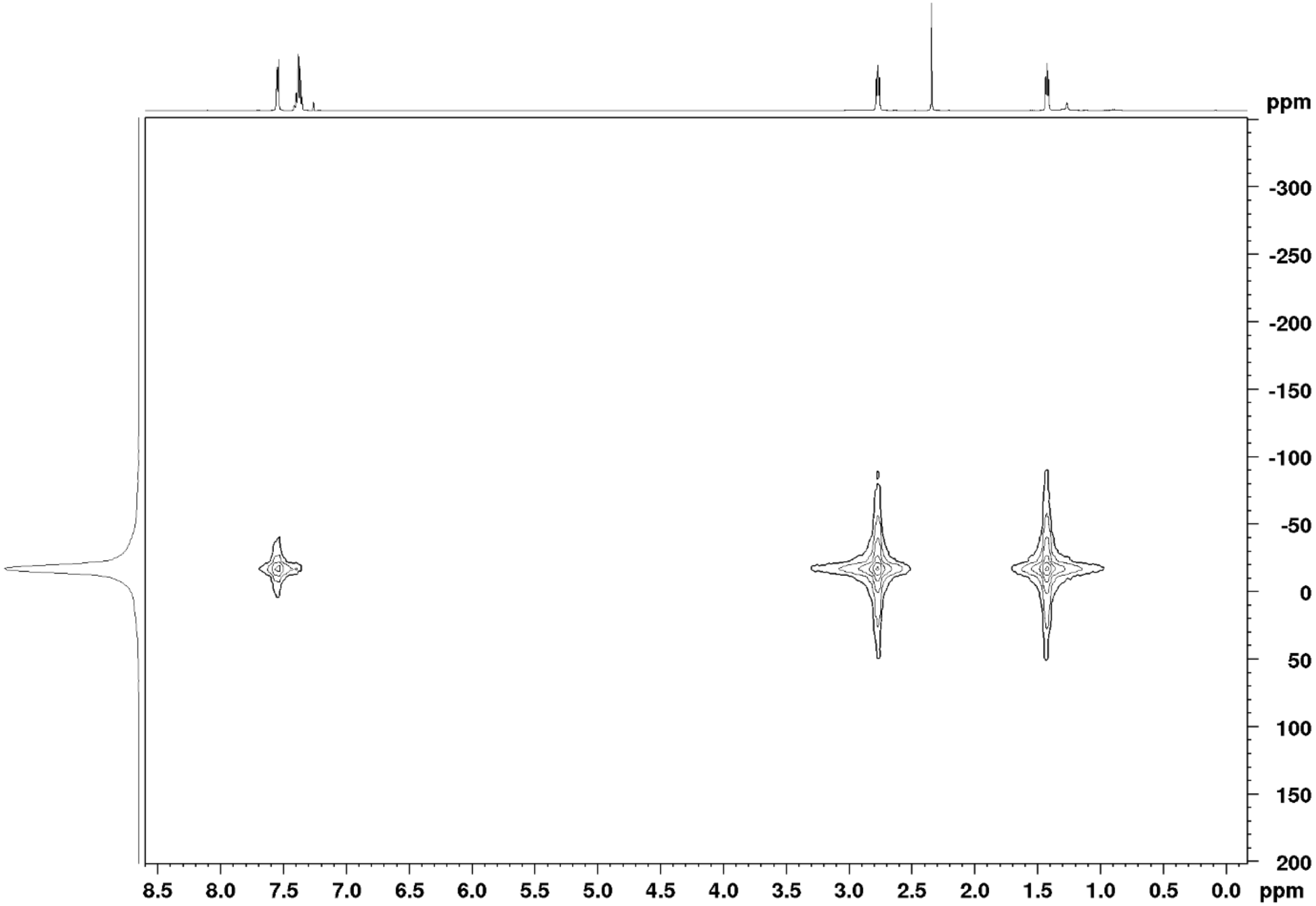

**Figure S58.** $^{1}$H NMR spectrum (500 MHz, $CDCl_3$, 298 K) of **1-benzyl-3-methyl-4,4-diphenyl-1,4-azasilinane (3ra)**.

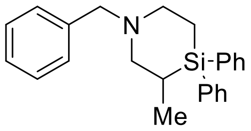


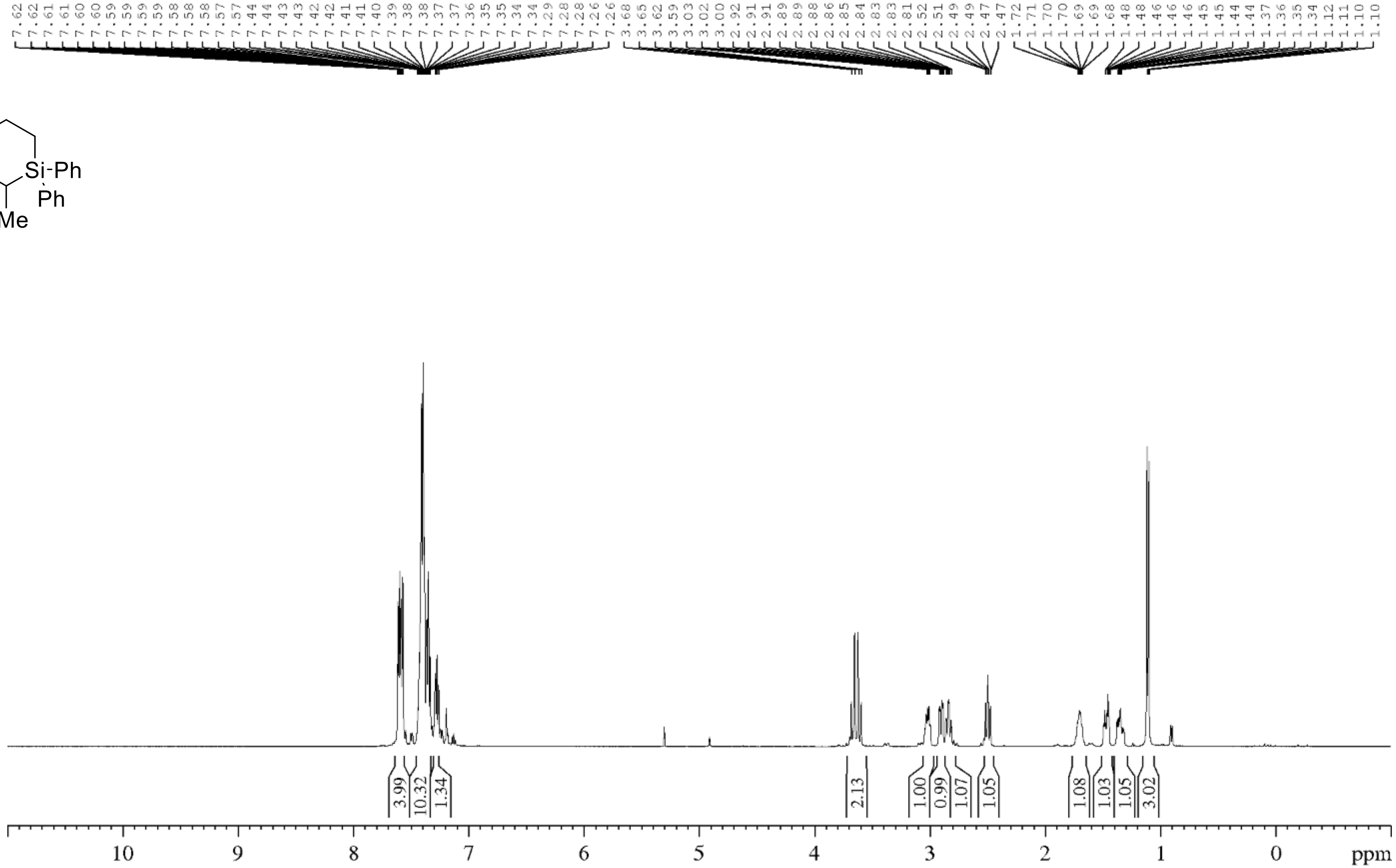

**Figure S59.** $^{13}C$ NMR spectrum (125 MHz, $CDCl_3$, 298 K) of **1-benzyl-3-methyl-4,4-diphenyl-1,4-azasilinane (3ra)**.

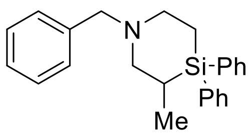


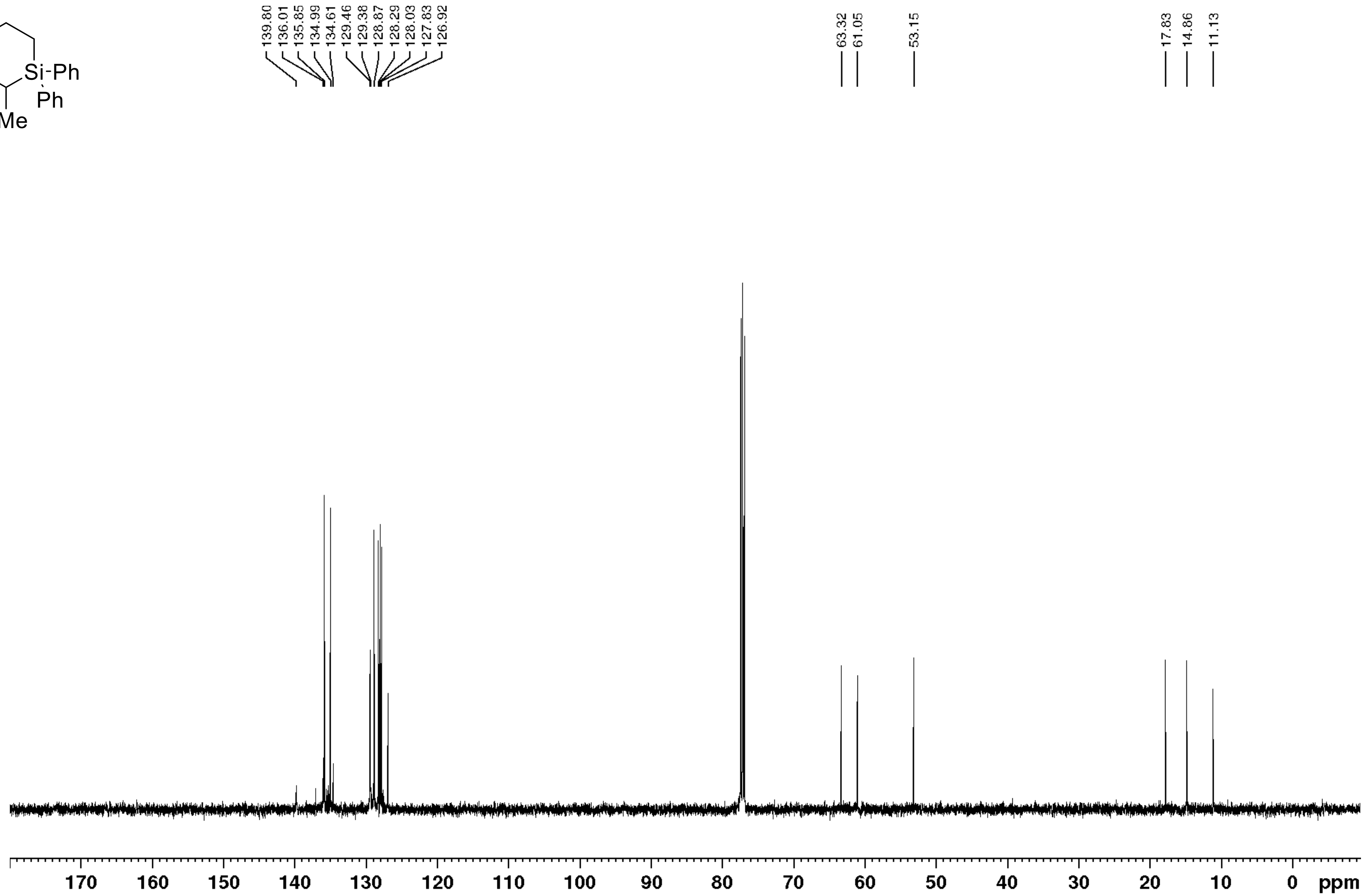

**Figure S60.** $^{1}H/^{29}Si$ HMQC NMR (500/99 MHz, $CDCl_3$, 298 K, optimized for $J$ = 7 Hz) of **1-benzyl-3-methyl-4,4-diphenyl-1,4-azasilinane (3ra)**.

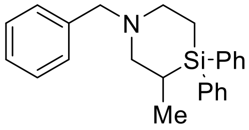


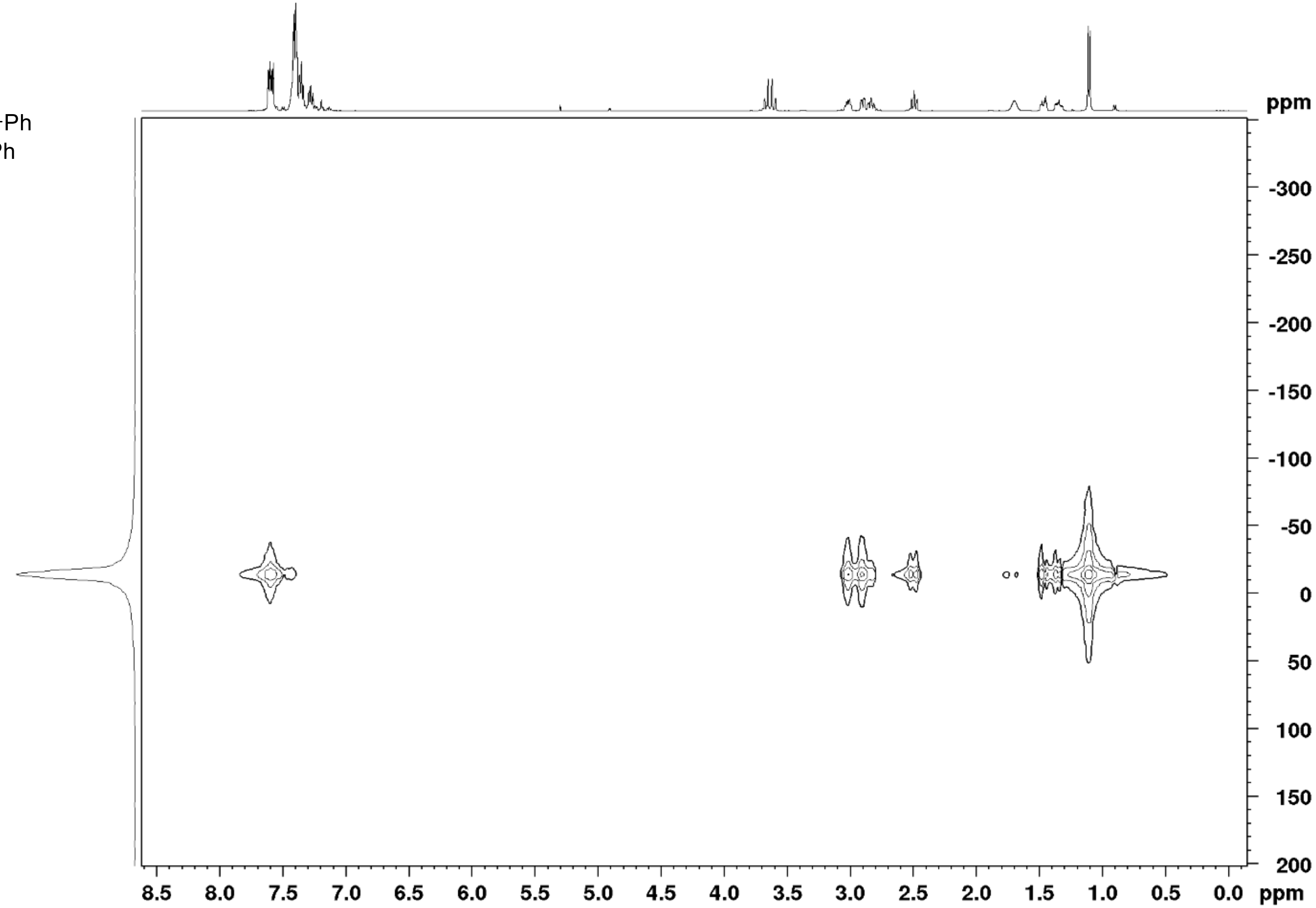

**Figure S61.** $^{1}$H NMR spectrum (500 MHz, $CDCl_3$, 298 K) of **1-benzyl-3-ethyl-4,4-diphenyl-1,4-azasilinane (3sa)**.

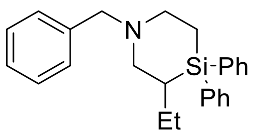


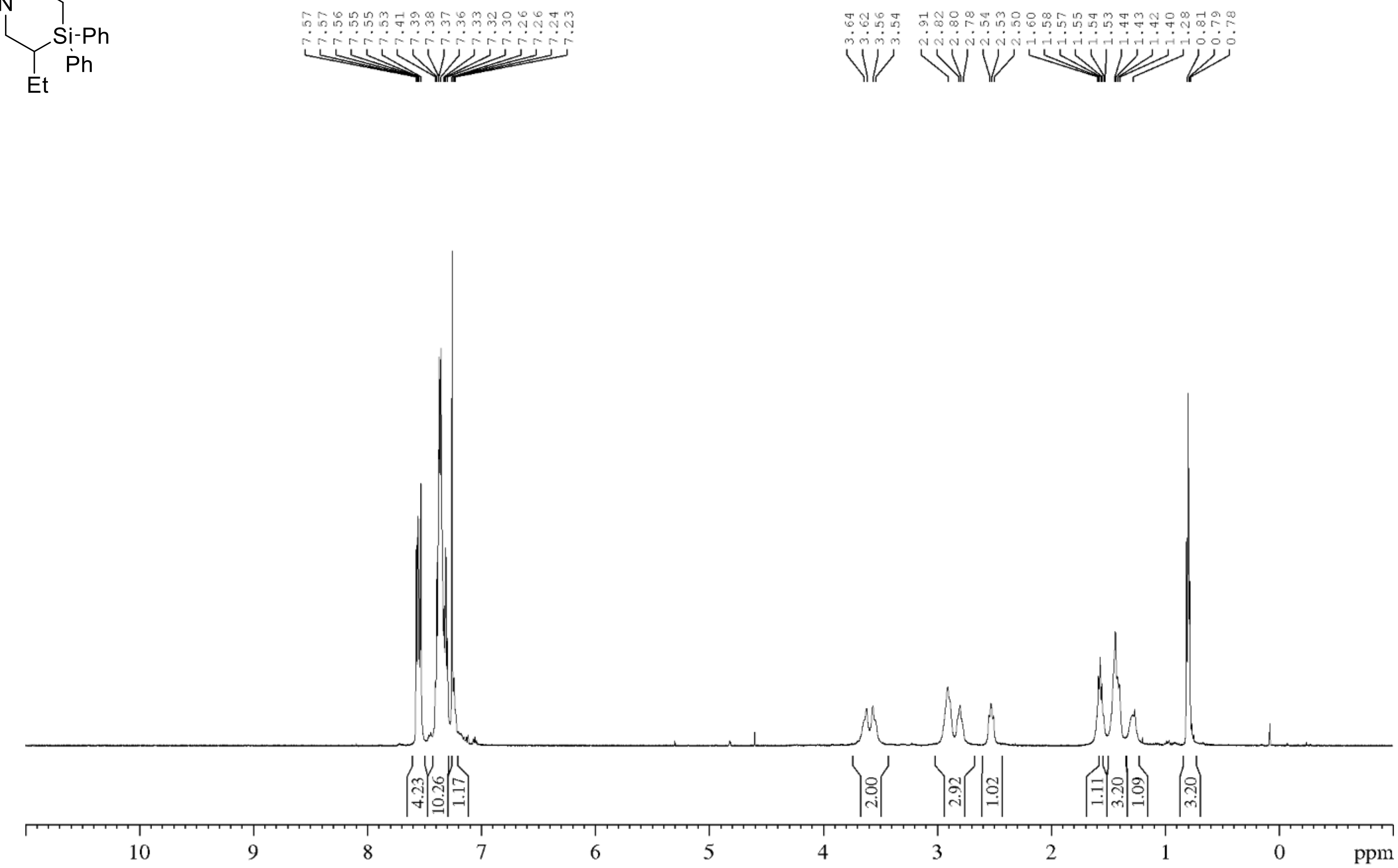

**Figure S62.** $^{13}C$ NMR spectrum (125 MHz, $CDCl_3$, 298 K) of **1-benzyl-3-ethyl-4,4-diphenyl-1,4-azasilinane (3sa)**.

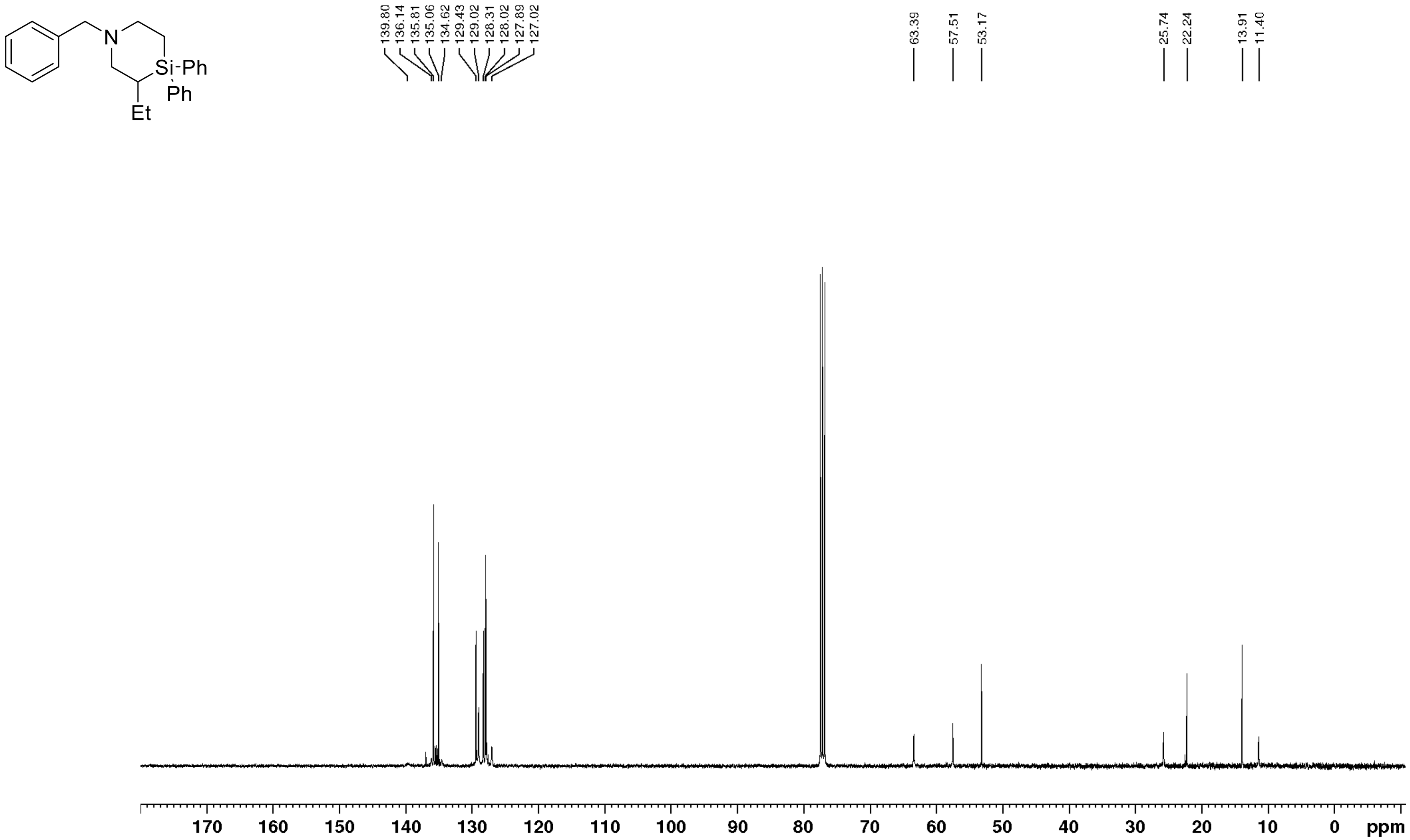

**Figure S63.** $^{1}H/^{29}Si$ HMQC NMR (500/99 MHz, $CDCl_3$, 298 K, optimized for $J$ = 7 Hz) of **1-benzyl-3-ethyl-4,4-diphenyl-1,4-azasilinane (3sa)**.

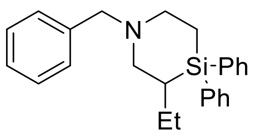


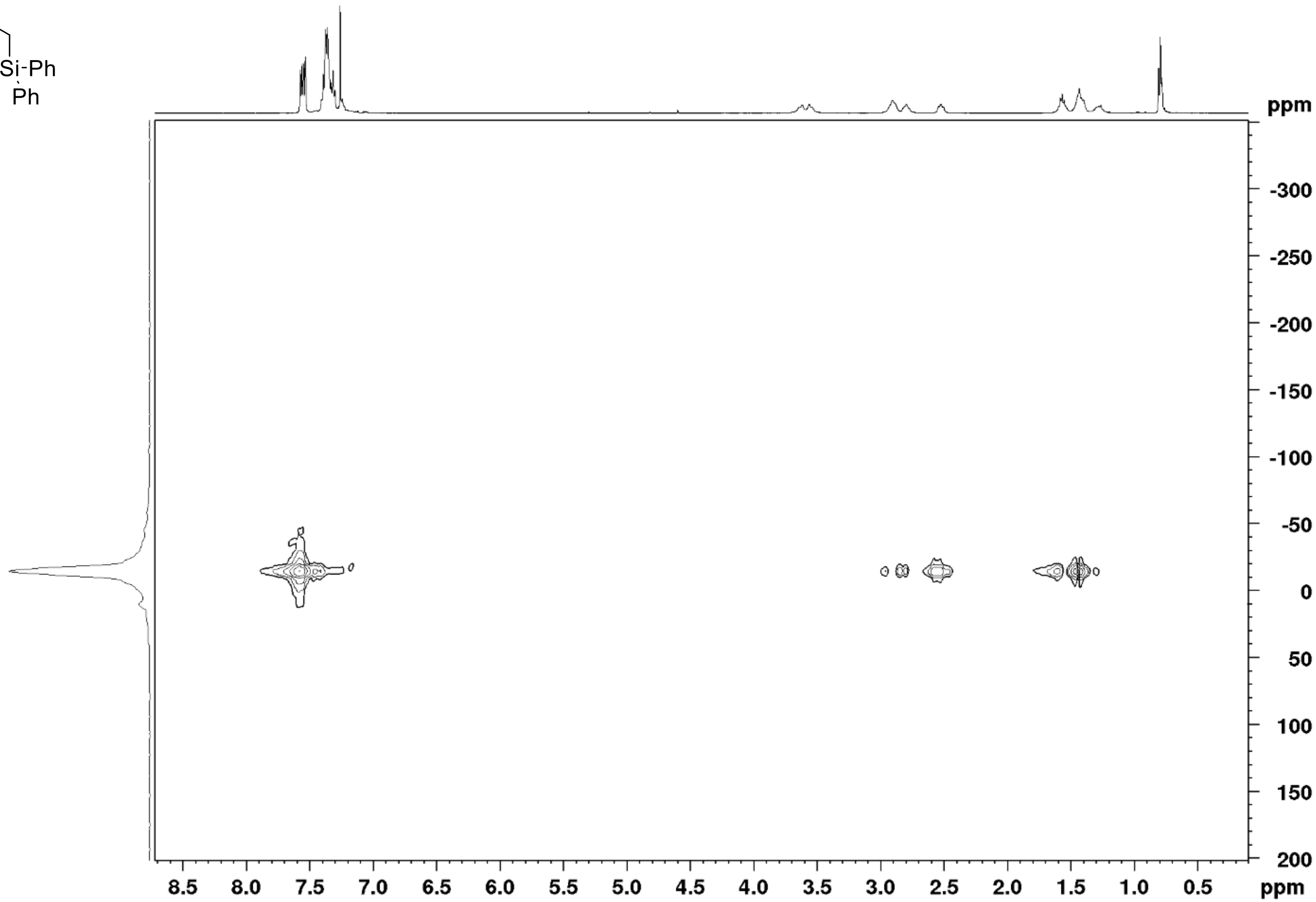

**Figure S64.** $^1$H NMR spectrum (500 MHz, $CDCl_3$, 298 K) of **3,5-dimethyl-4,4-diphenyl-1-propyl-1,4-azasilinane (3ta) (d.r. = 58:42)**.

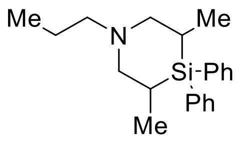

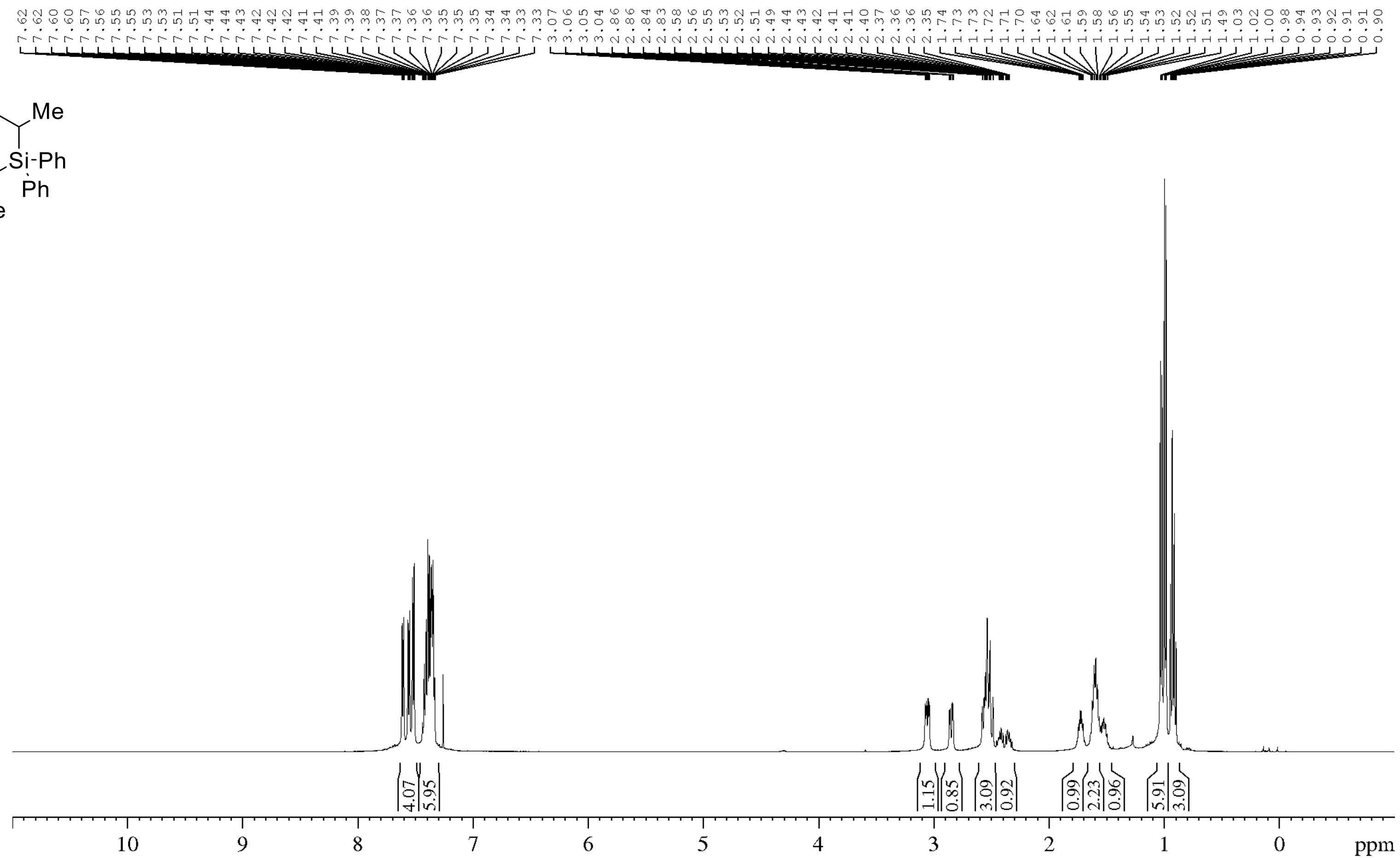

**Figure S65.** $^{13}C$ NMR spectrum (125 MHz, $CDCl_3$, 298 K) of **3,5-dimethyl-4,4-diphenyl-1-propyl-1,4-azasilinane (3ta) (d.r. = 58:42)**.

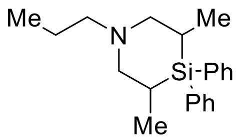


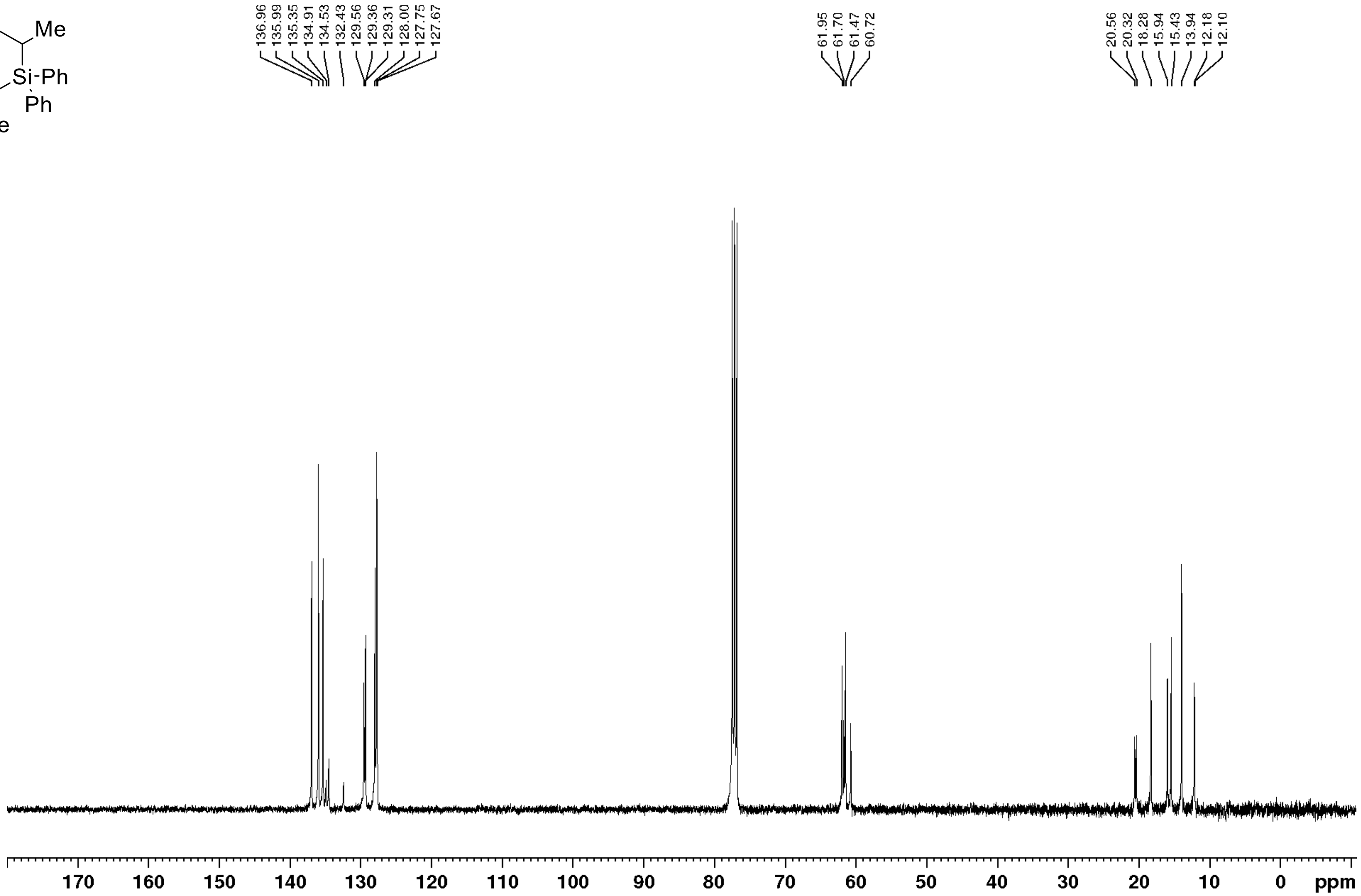

**Figure S66.** $^1H/^{29}Si$ HMQC NMR (500/99 MHz, $CDCl_3$, 298 K, optimized for $J$ = 7 Hz) of **3,5-dimethyl-4,4-diphenyl-1-propyl-1,4-azasilinane (3ta) (d.r. = 58:42)**.

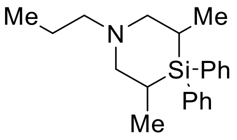


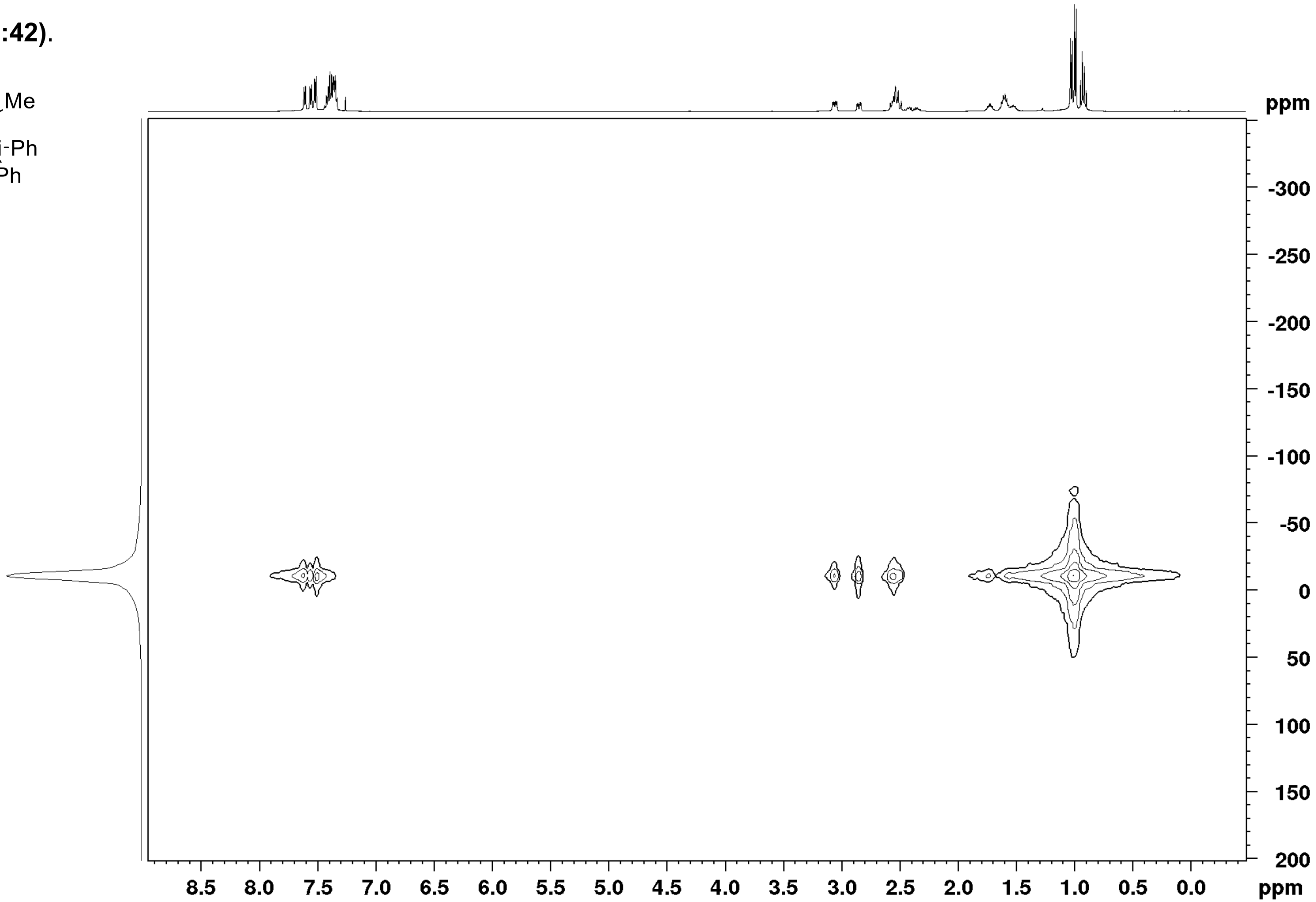

**Figure S67.** $^{1}$H NMR spectrum (500 MHz, $CDCl_3$, 298 K) of **1-ethyl-6-methyl-4,4-diphenyl-1,2,3,4-tetrahydrobenzo[*b*][1,4]azasiline (4ua)**.

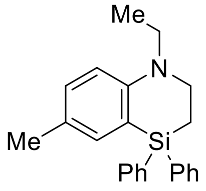


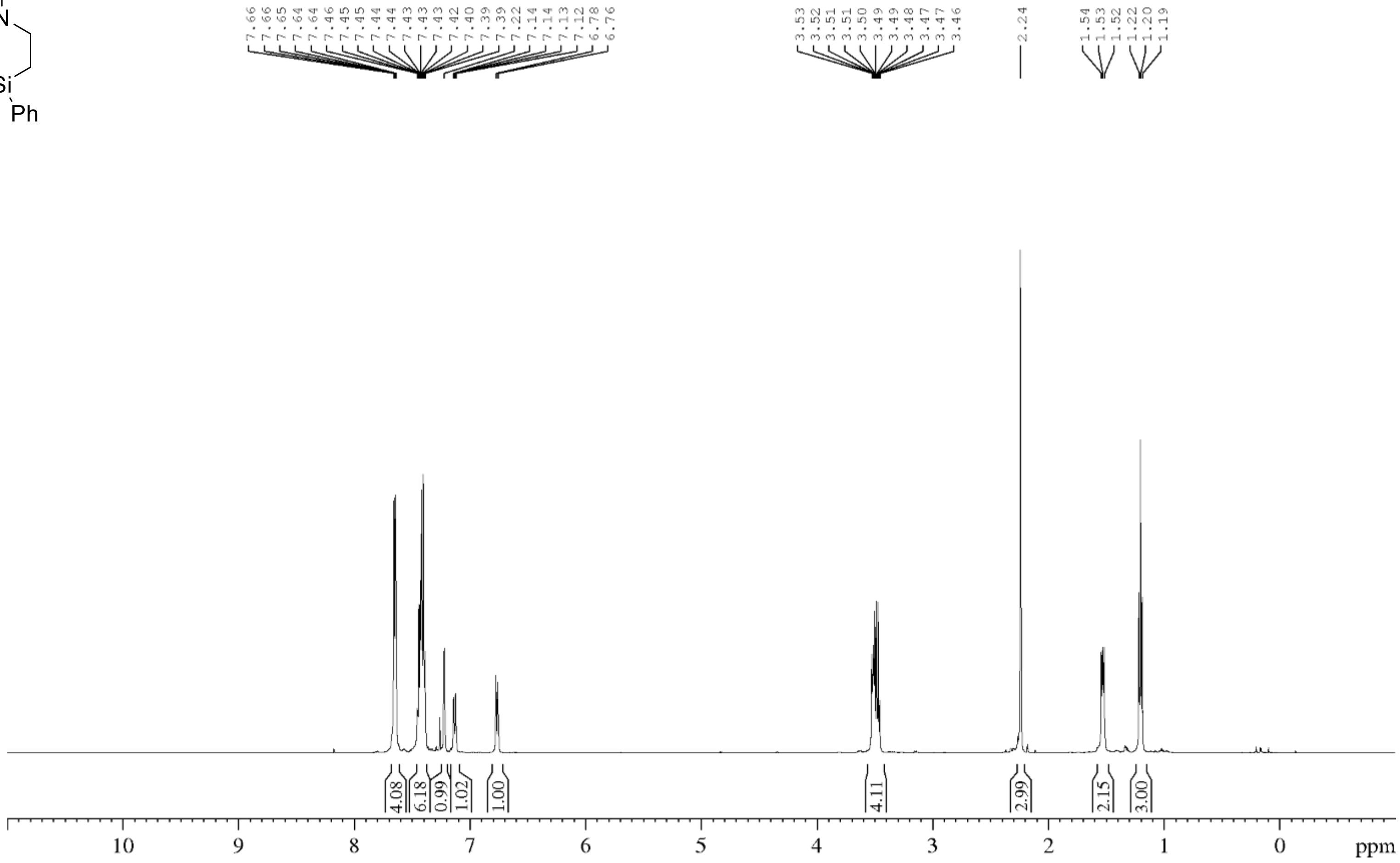

**Figure S68.** $^{13}C$ NMR spectrum (125 MHz, $CDCl_3$, 298 K) of **1-ethyl-6-methyl-4,4-diphenyl-1,2,3,4-tetrahydrobenzo[*b*][1,4]azasiline (4ua)**.

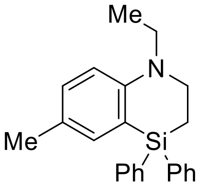


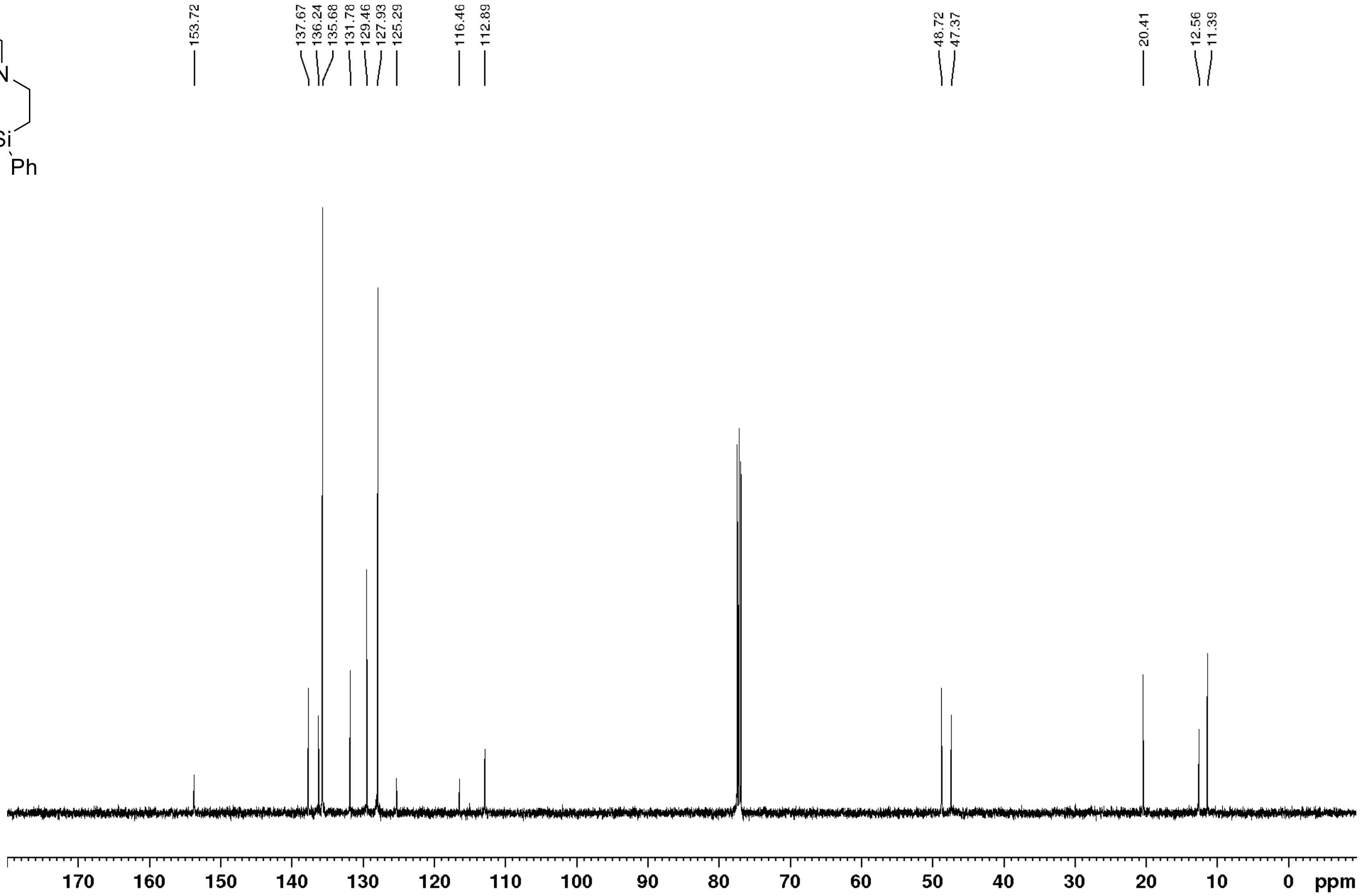

**Figure S69.** $^{1}H/^{29}Si$ HMQC NMR (500/99 MHz, $CDCl_3$, 298 K, optimized for $J$ = 7 Hz) of

**1-ethyl-6-methyl-4,4-diphenyl-1,2,3,4-tetrahydrobenzo[*b*][1,4]azasiline (4ua)**.

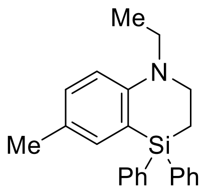


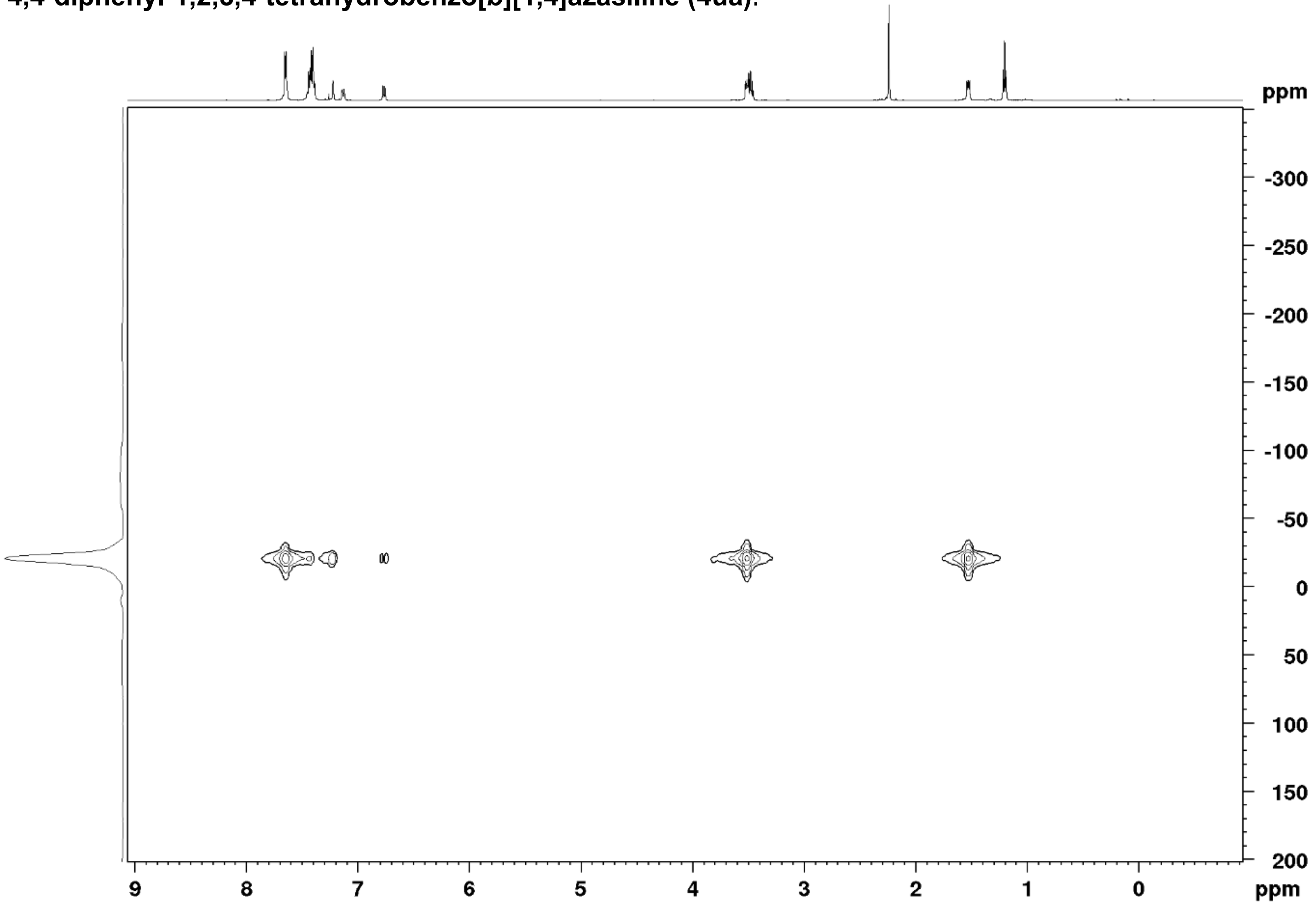

**Figure S70.** $^{1}$H NMR spectrum (500 MHz, $CDCl_3$, 298 K) of

**8-methyl-1,1,6,6-tetraphenyl-2,3,5,6-tetrahydro-1*H*,4*H*-3a-aza-1,6-disilaphenalene (5ua)**.

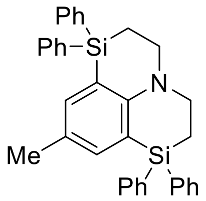

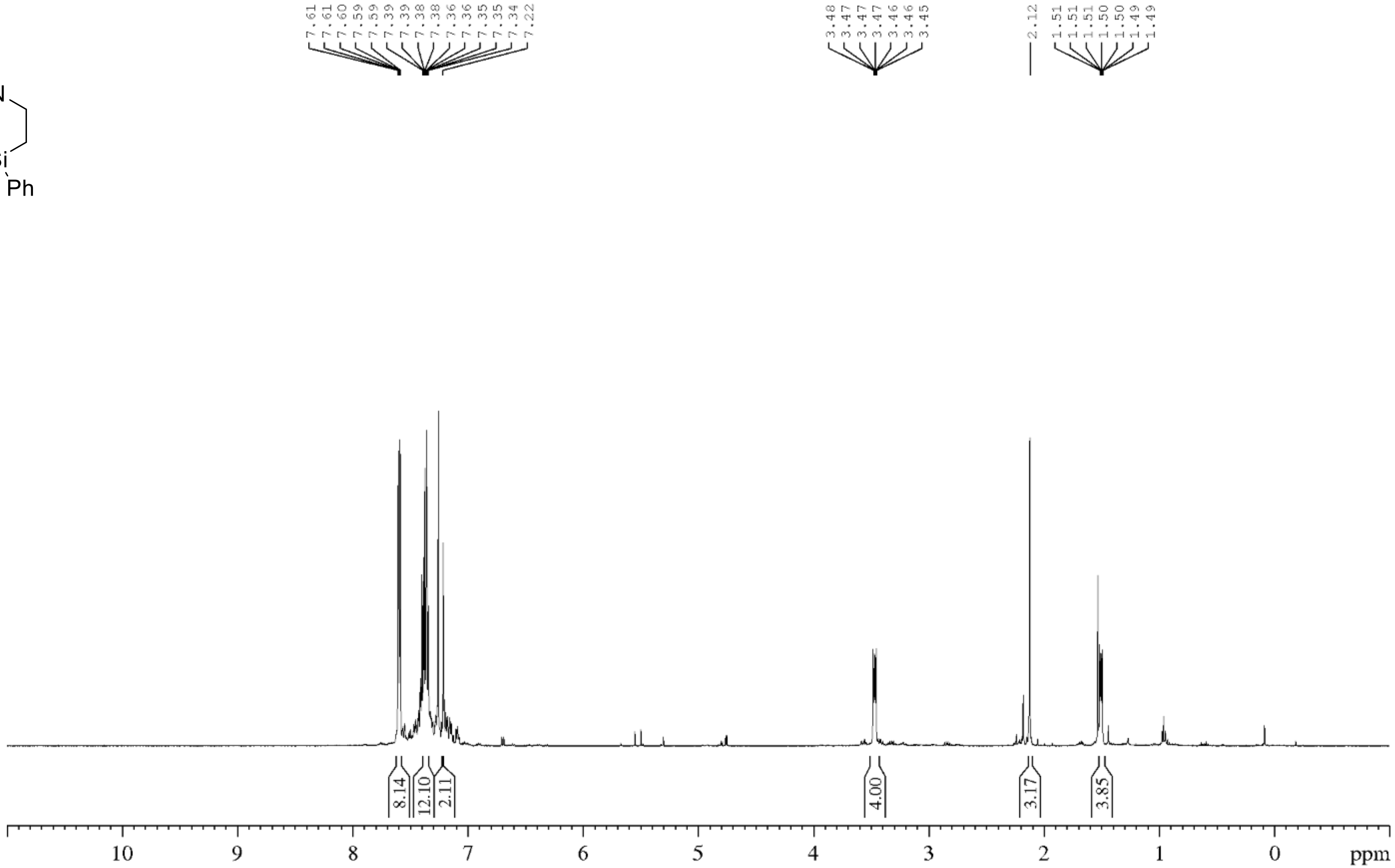

**Figure S71.** $^{13}$C NMR spectrum (125 MHz, $CDCl_3$, 298 K) of

**8-methyl-1,1,6,6-tetraphenyl-2,3,5,6-tetrahydro-1*H*,4*H*-3a-aza-1,6-disilaphenalene (5ua)**.

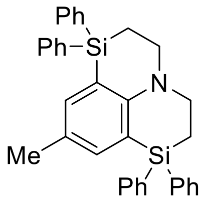


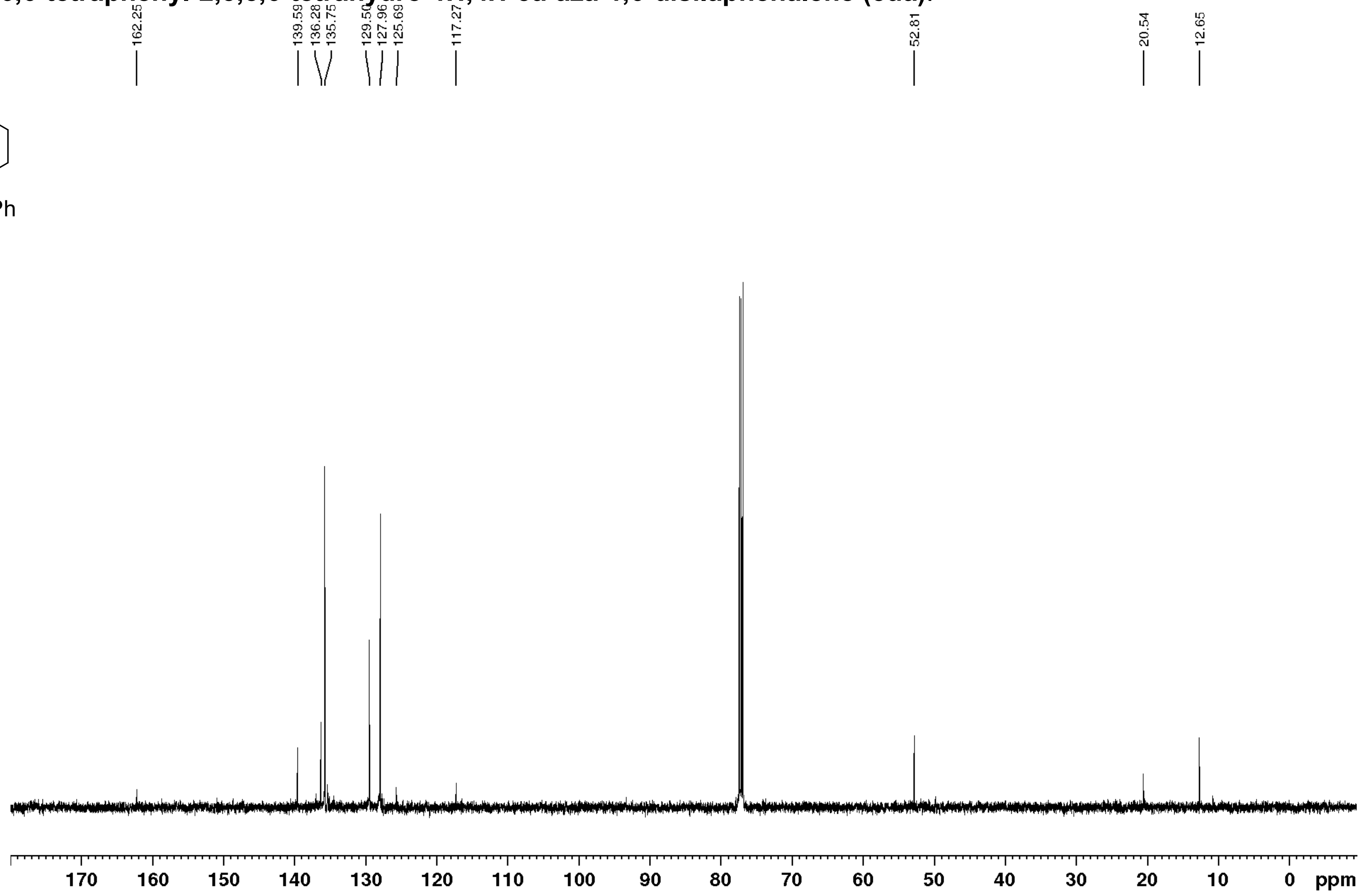

**Figure S72.** $^{1}H/^{29}Si$ HMQC NMR (500/99 MHz, $CDCl_3$, 298 K, optimized for $J$ = 7 Hz) of **8-methyl-1,1,6,6-tetraphenyl-2,3,5,6-tetrahydro-1*H*,4*H*-3a-aza-1,6-disilaphenalene (5ua)**.

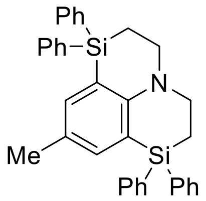

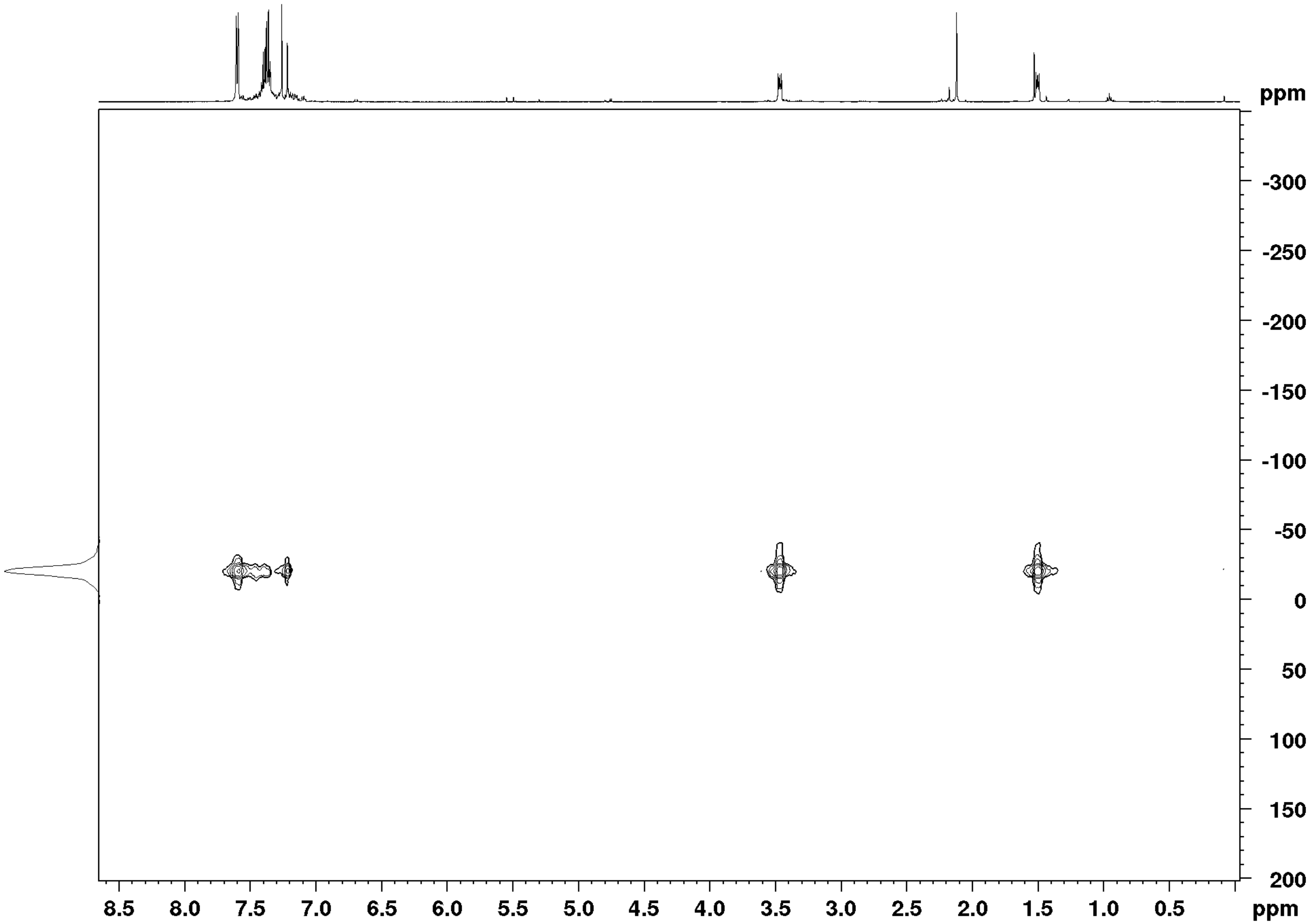

**Figure S73.** $^{1}H$ NMR spectrum (500 MHz, $CDCl_3$, 298 K) of **1-benzyl-4,4-di-*p*-tolyl-1,4-azasilinane (3ab)**.

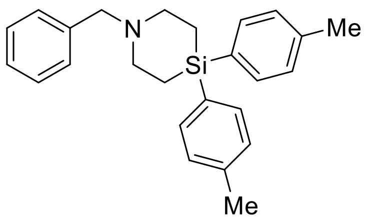


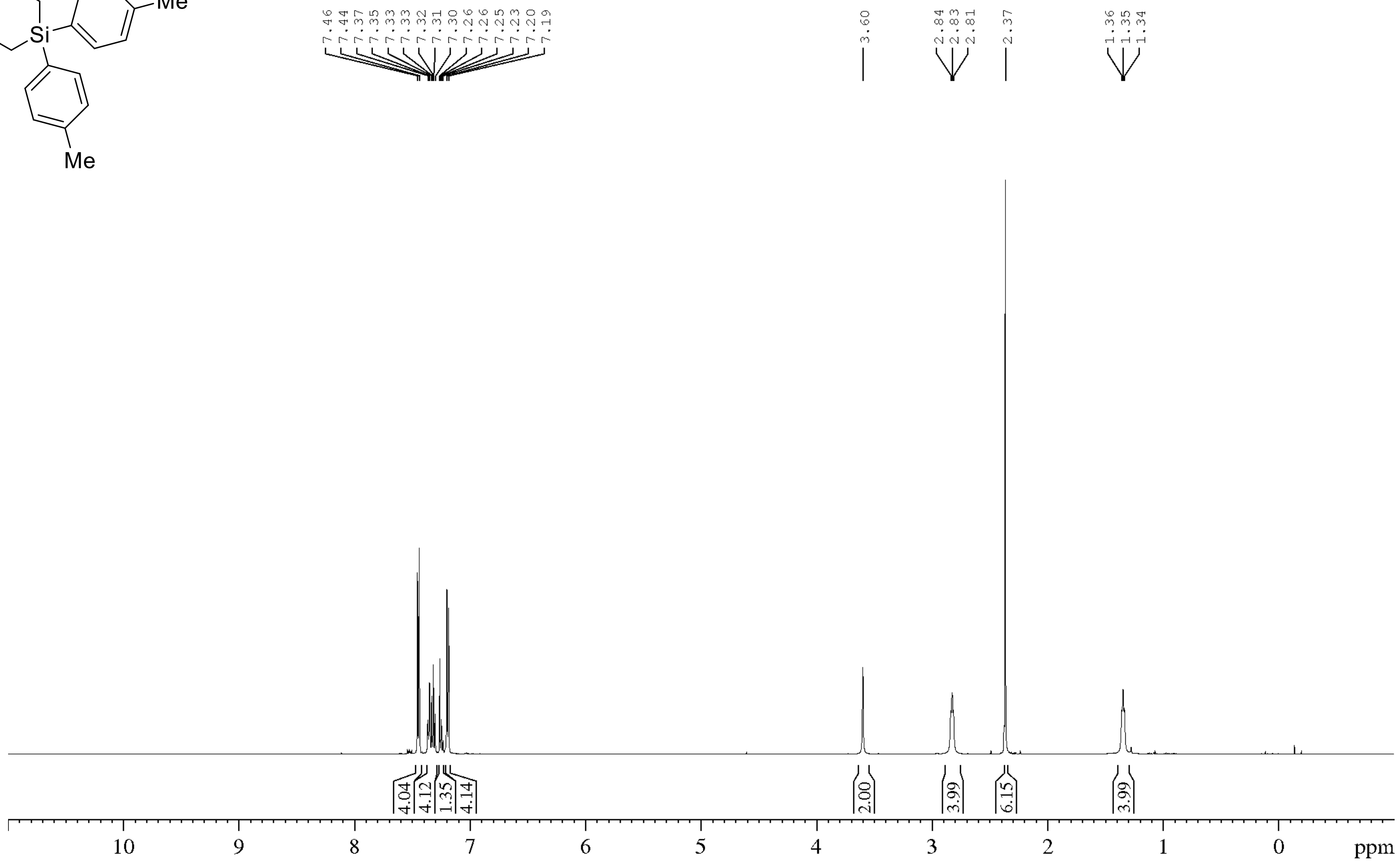

**Figure S74.** $^{13}$C NMR spectrum (125 MHz, $CDCl_3$, 298 K) of **1-benzyl-4,4-di-*p*-tolyl-1,4-azasilinane (3ab)**.

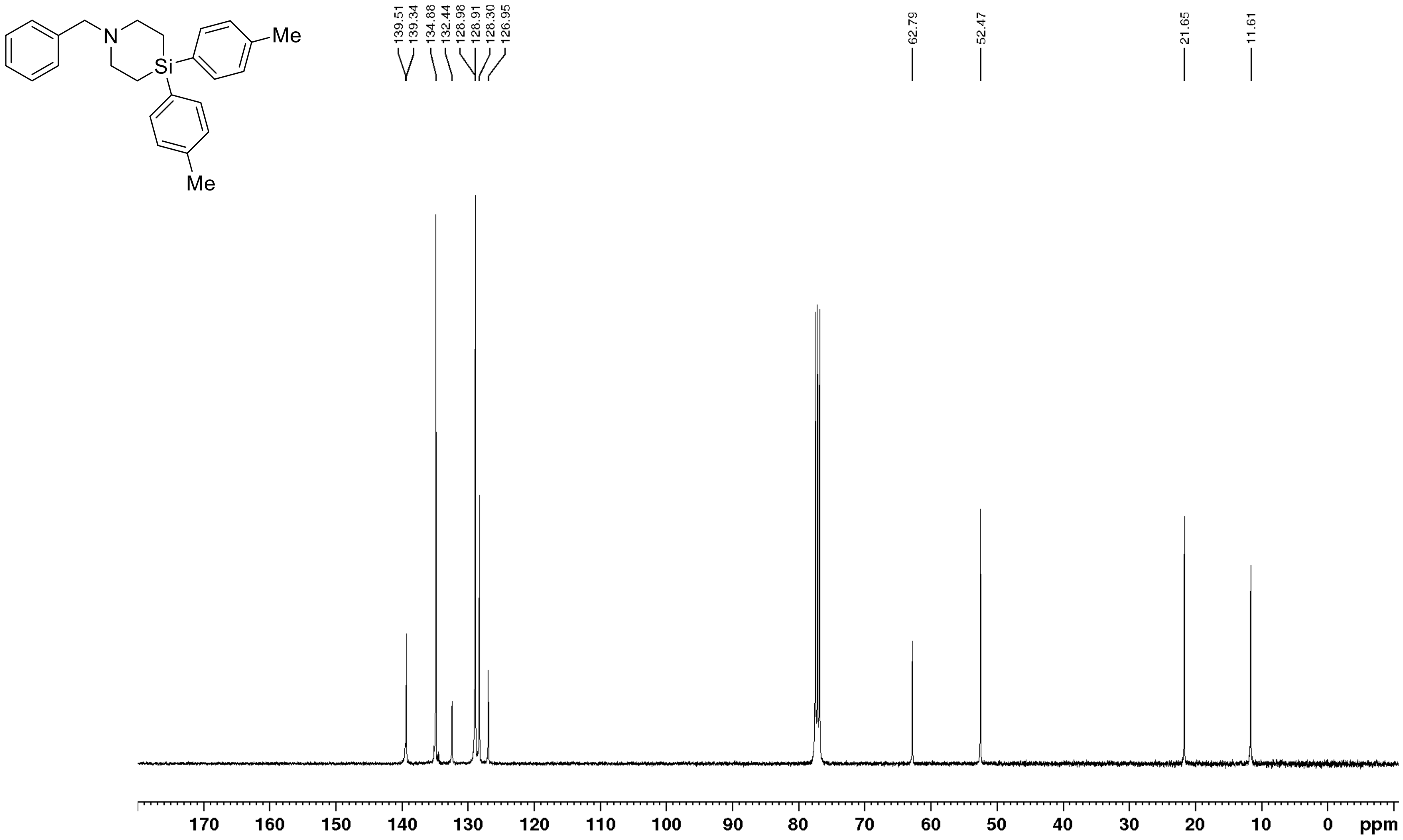

**Figure S75.** $^{1}H/^{29}Si$ HMQC NMR (500/99 MHz, $CDCl_3$, 298 K, optimized for $J$ = 7 Hz) of **1-benzyl-4,4-di-*p*-tolyl-1,4-azasilinane (3ab)**.

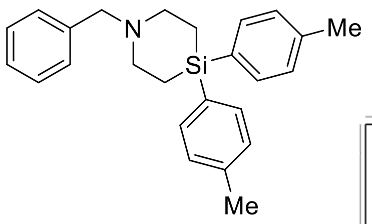


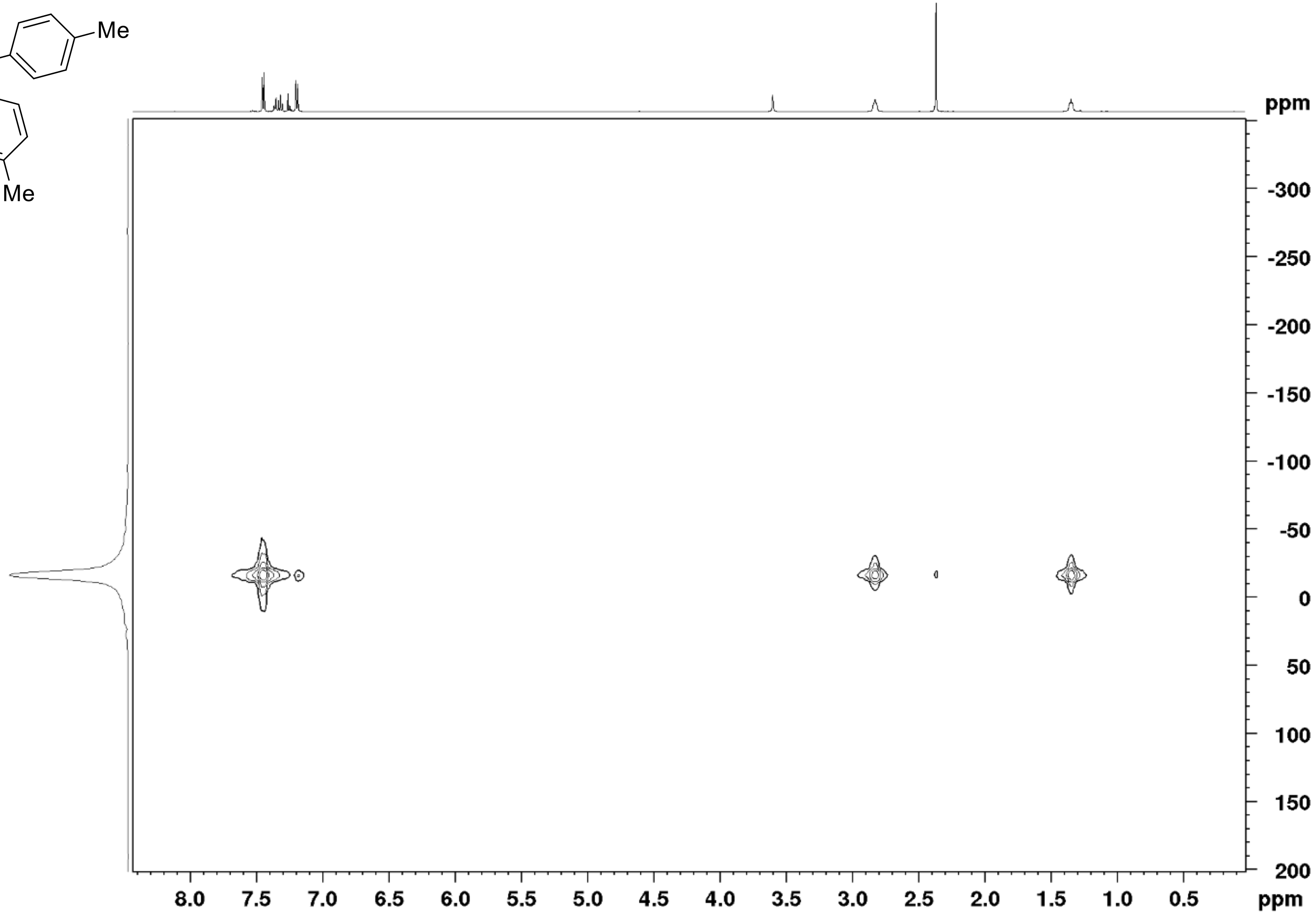

**Figure S76.** $^1$H NMR spectrum (500 MHz, $CDCl_3$, 298 K) of **1-benzyl-4,4-bis(4-(*tert*-butyl)phenyl)-1,4-azasilinane (3ac)**.

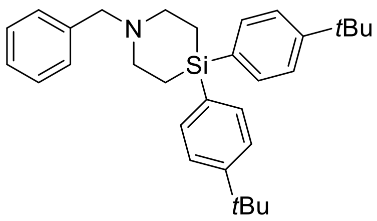


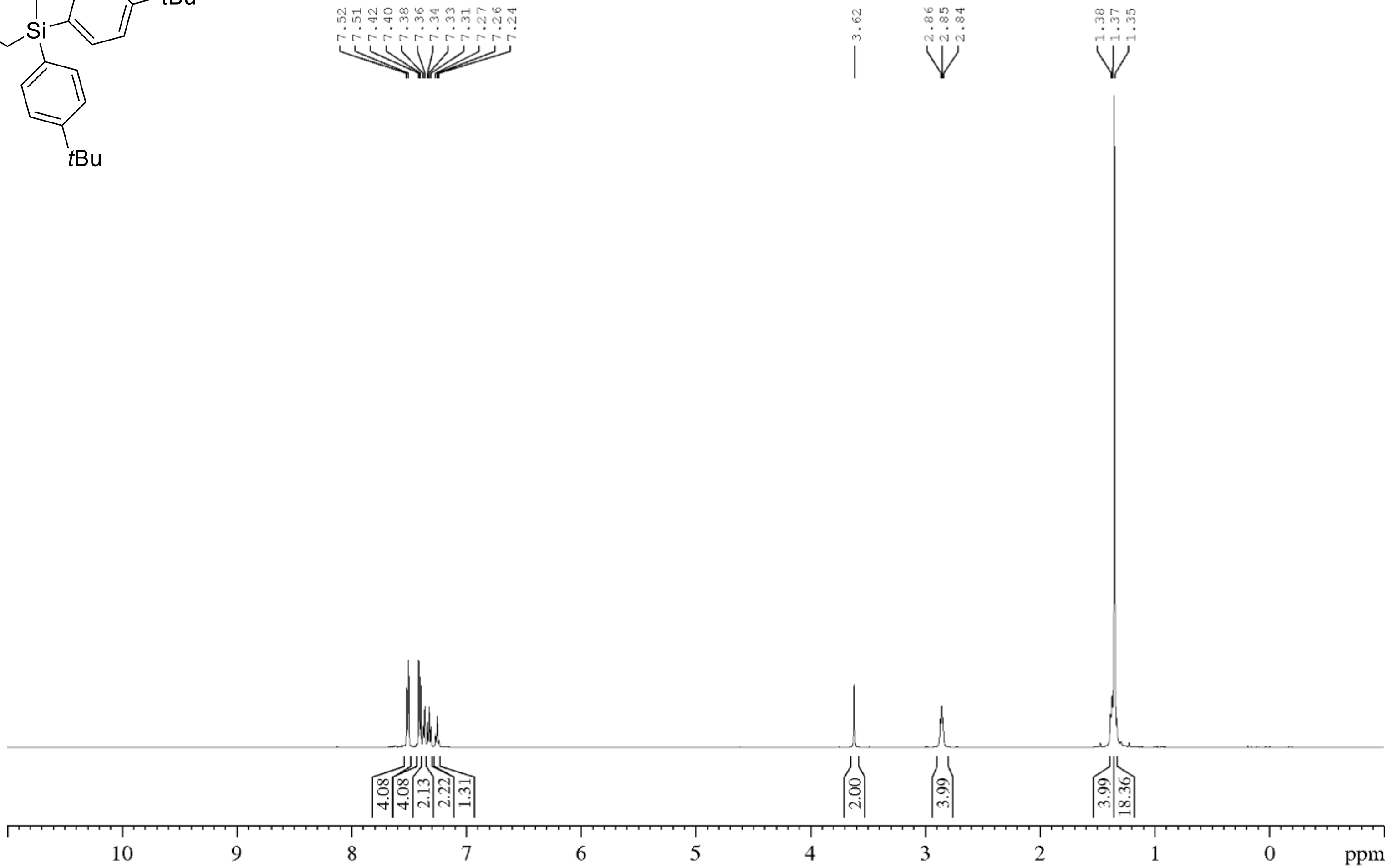

**Figure S77.** $^{13}$C NMR spectrum (125 MHz, $CDCl_3$, 298 K) of **1-benzyl-4,4-bis(4-(*tert*-butyl)phenyl)-1,4-azasilinane (3ac)**.

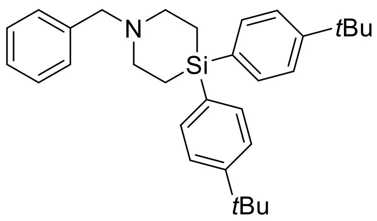


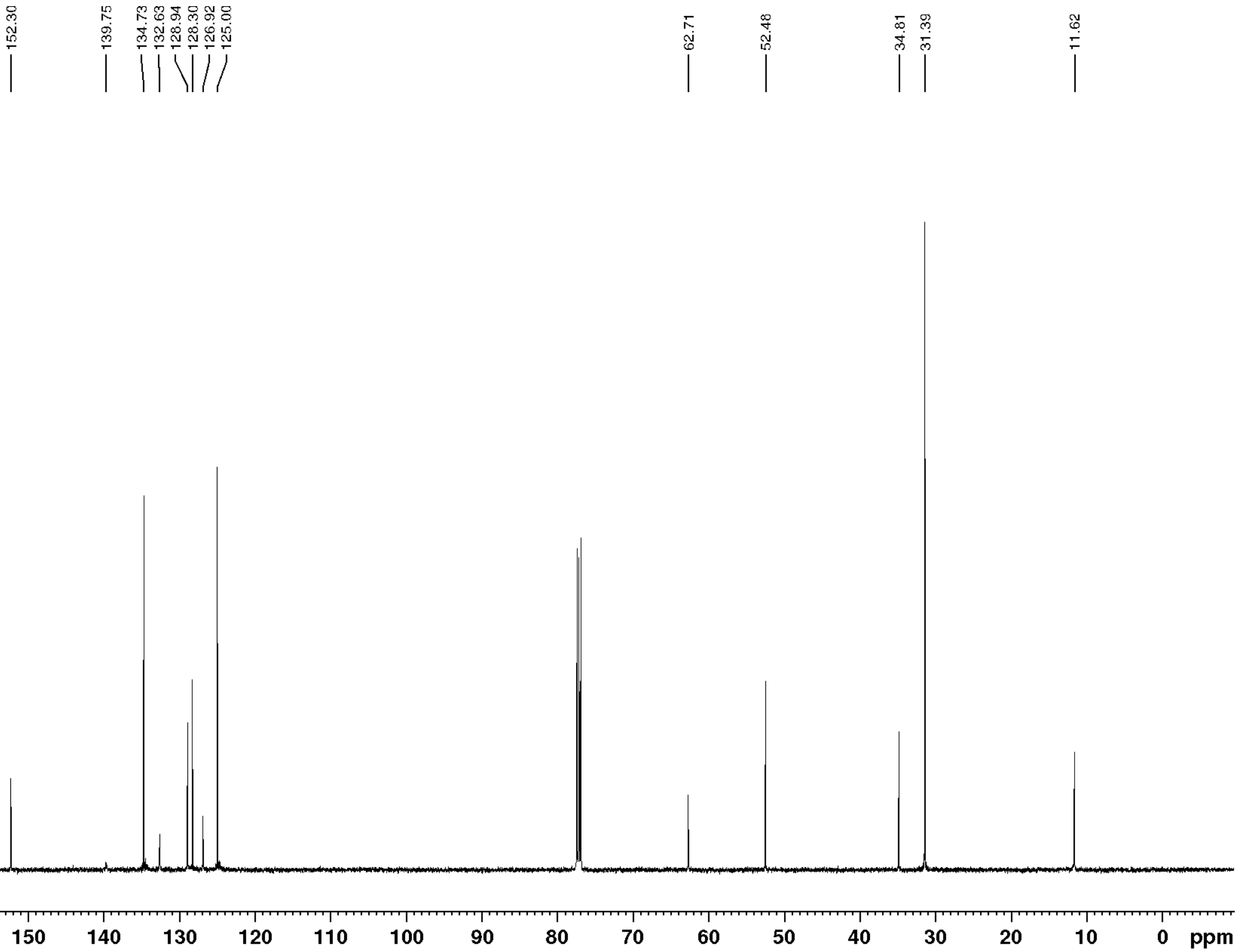

**Figure S78.** $^{1}H/^{29}Si$ HMQC NMR (500/99 MHz, $CDCl_3$, 298 K, optimized for $J$ = 7 Hz) of

**1-benzyl-4,4-bis(4-(*tert*-butyl)phenyl)-1,4-azasilinane (3ac)**.

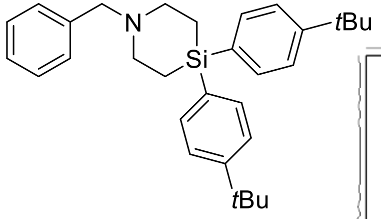


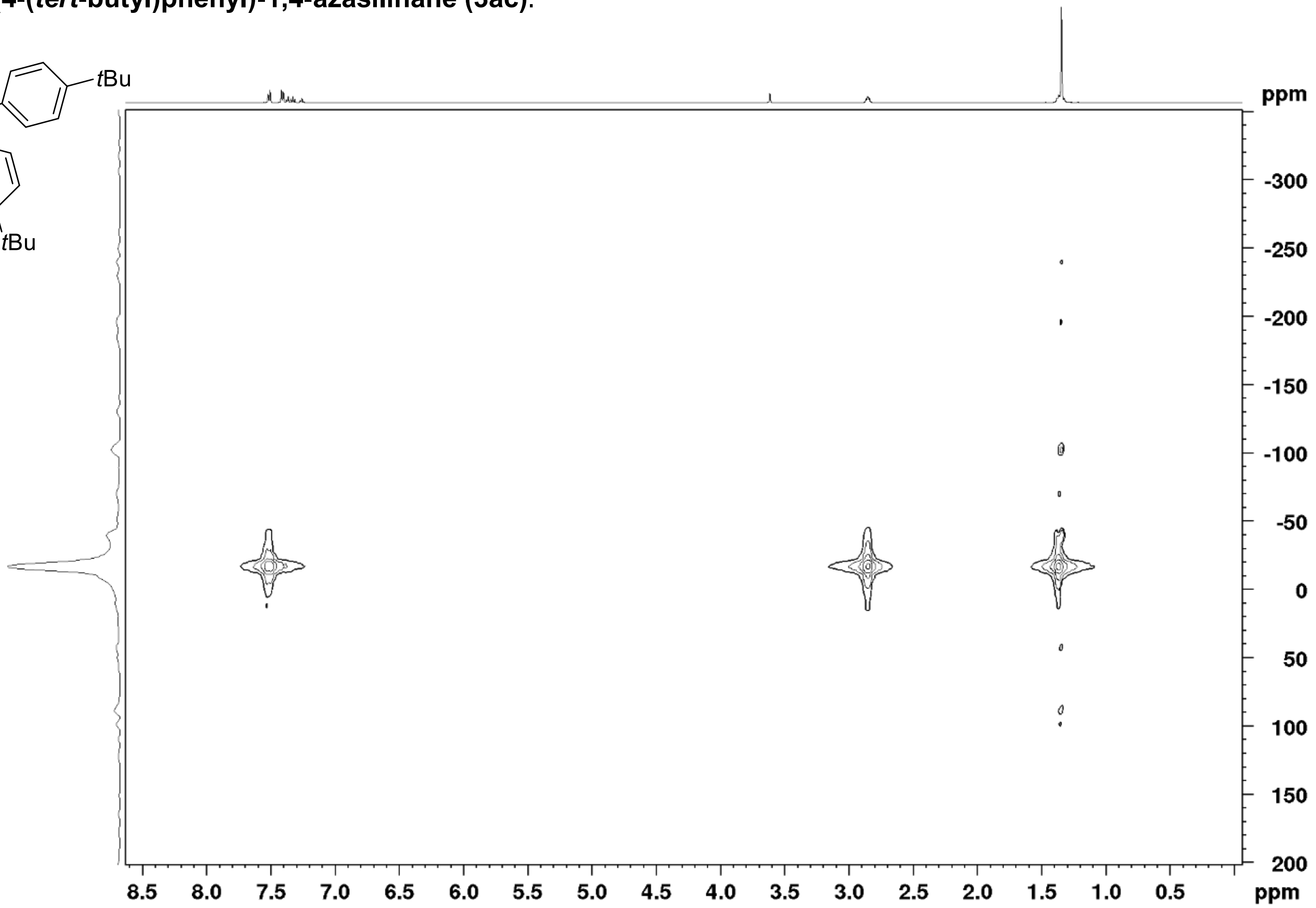

**Figure S79.** $^1$H NMR spectrum (500 MHz, $CDCl_3$, 298 K) of **1-benzyl-4,4-bis(4-fluorophenyl)-1,4-azasilinane (3ad)**.

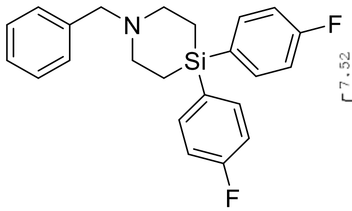


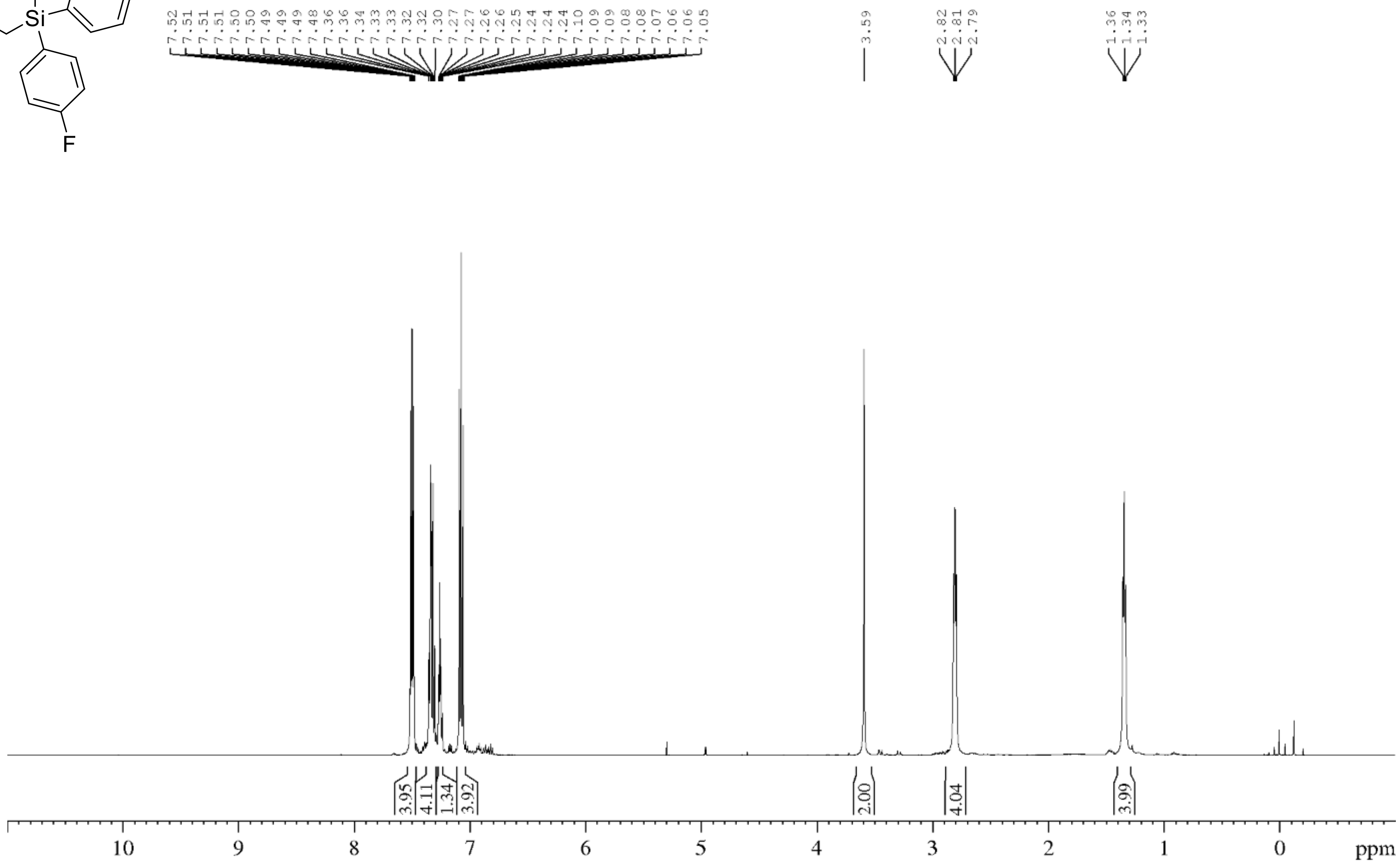

**Figure S80.** $^{13}$C NMR spectrum (125 MHz, $CDCl_3$, 298 K) of **1-benzyl-4,4-bis(4-fluorophenyl)-1,4-azasilinane (3ad)**.

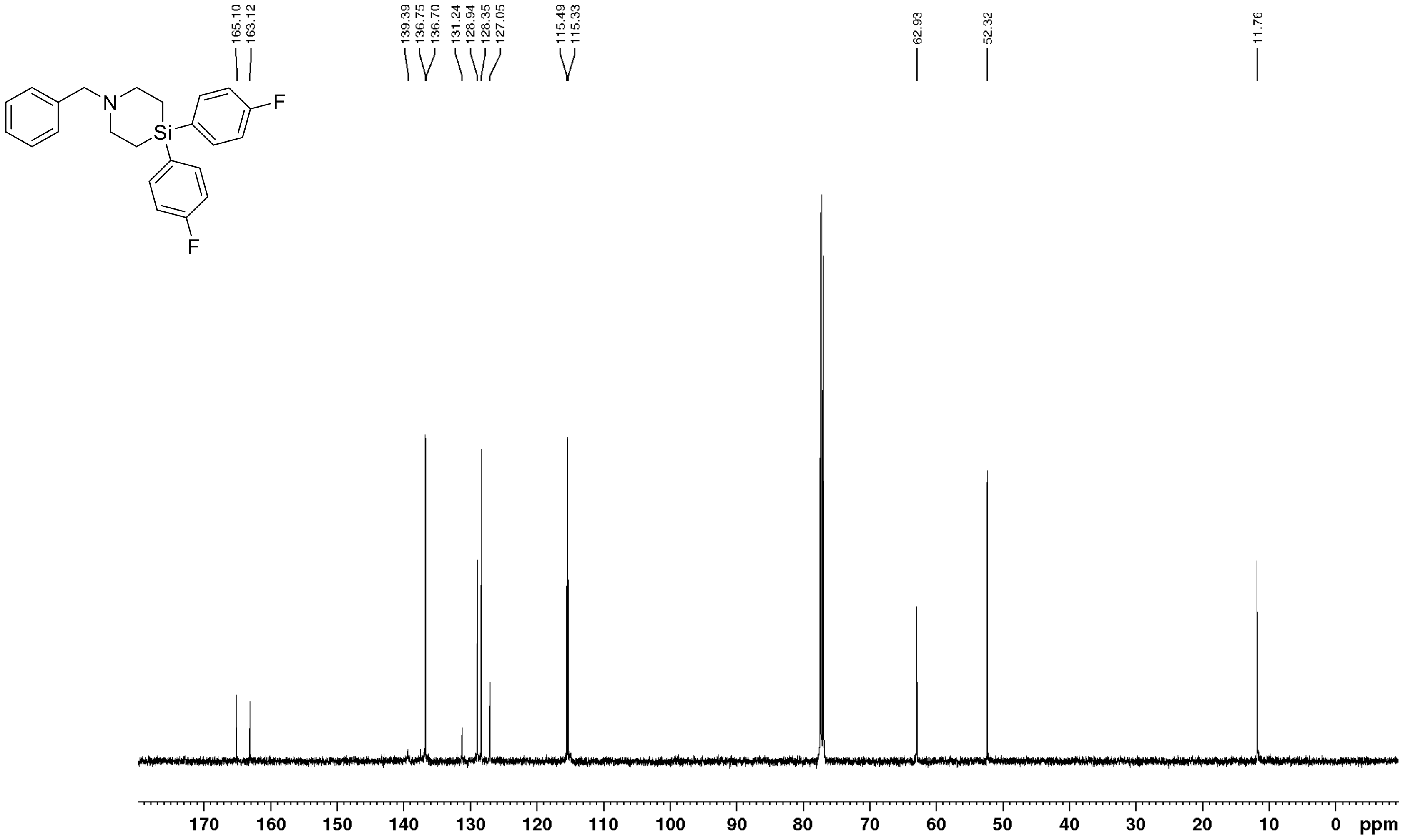

**Figure S81.** $^{19}$F NMR spectrum (471 MHz, $CDCl_3$, 298 K) of **1-benzyl-4,4-bis(4-fluorophenyl)-1,4-azasilinane (3ad)**.

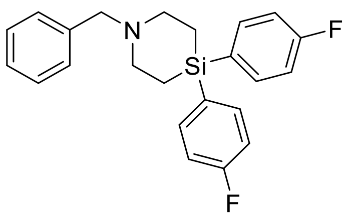


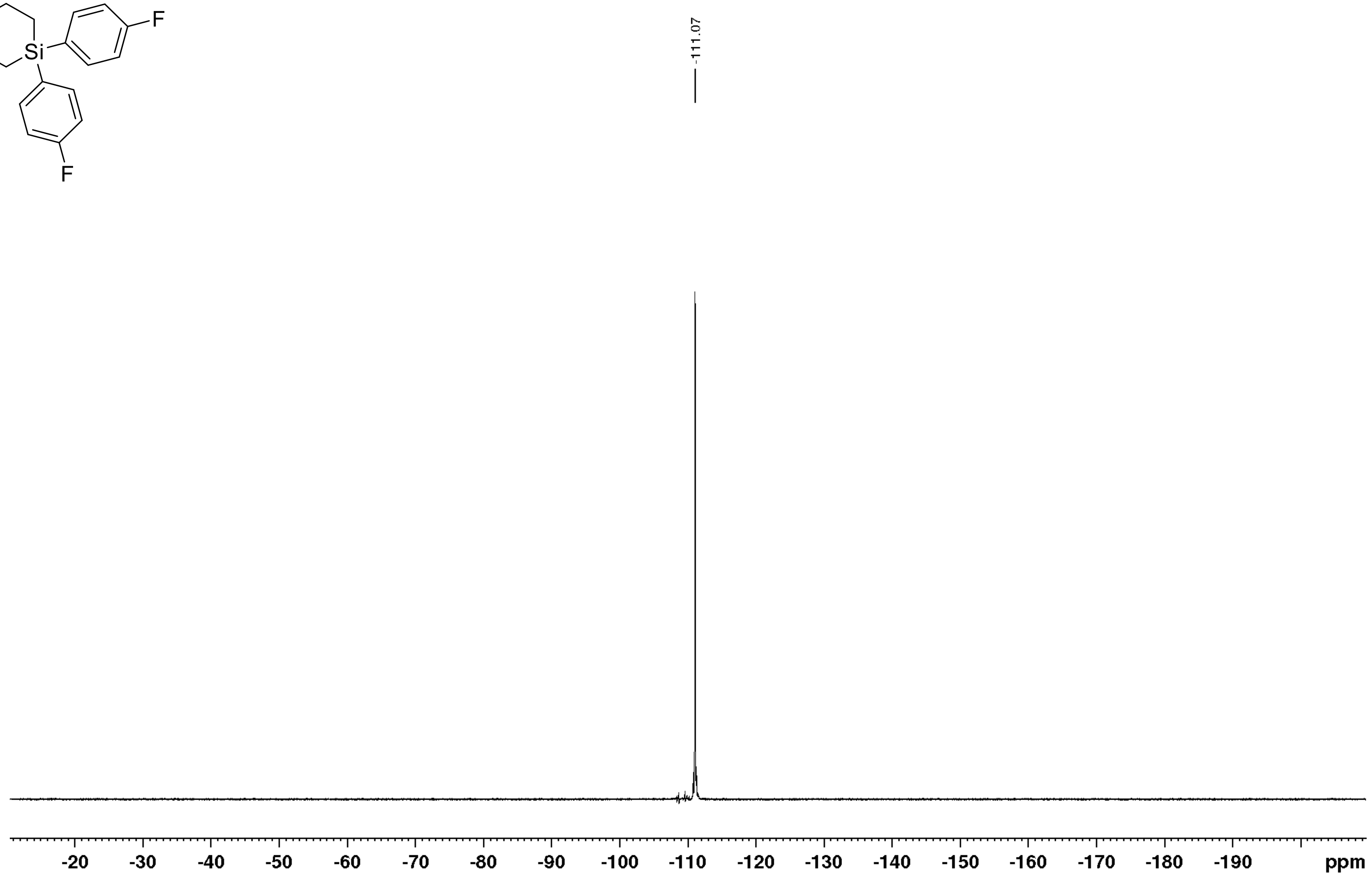

**Figure S82.** $^{1}H/^{29}Si$ HMQC NMR (500/99 MHz, $CDCl_3$, 298 K, optimized for $J$ = 7 Hz) of **1-benzyl-4,4-bis(4-fluorophenyl)-1,4-azasilinane (3ad)**.

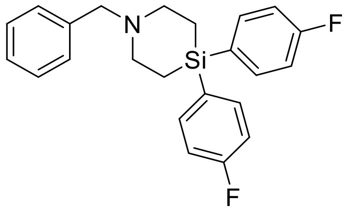


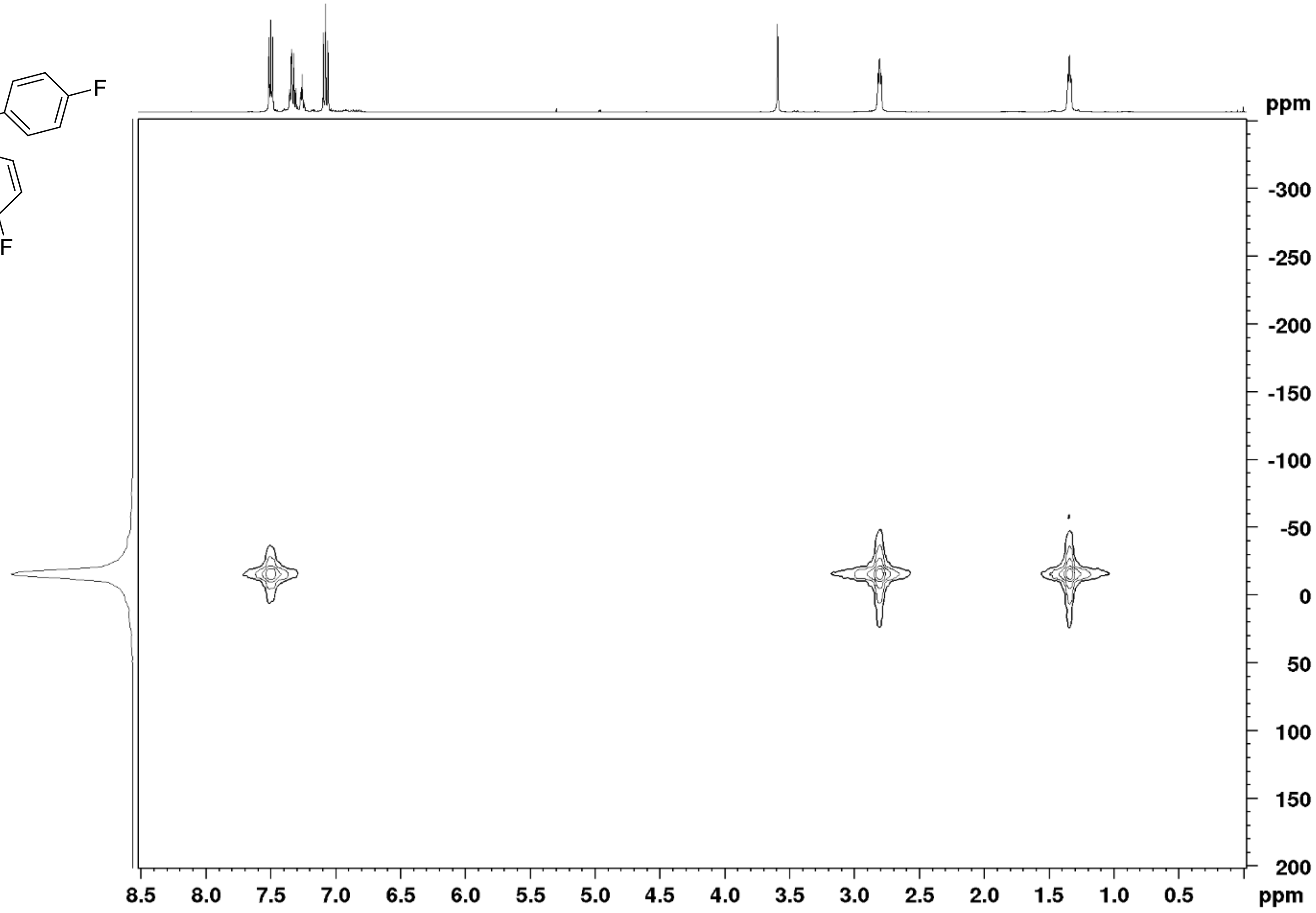

**Figure S83.** $^1$H NMR spectrum (500 MHz, $CDCl_3$, 298 K) of **1-benzyl-4-(naphthalen-1-yl)-4-phenyl-1,4-azasilinane (3ae)**.

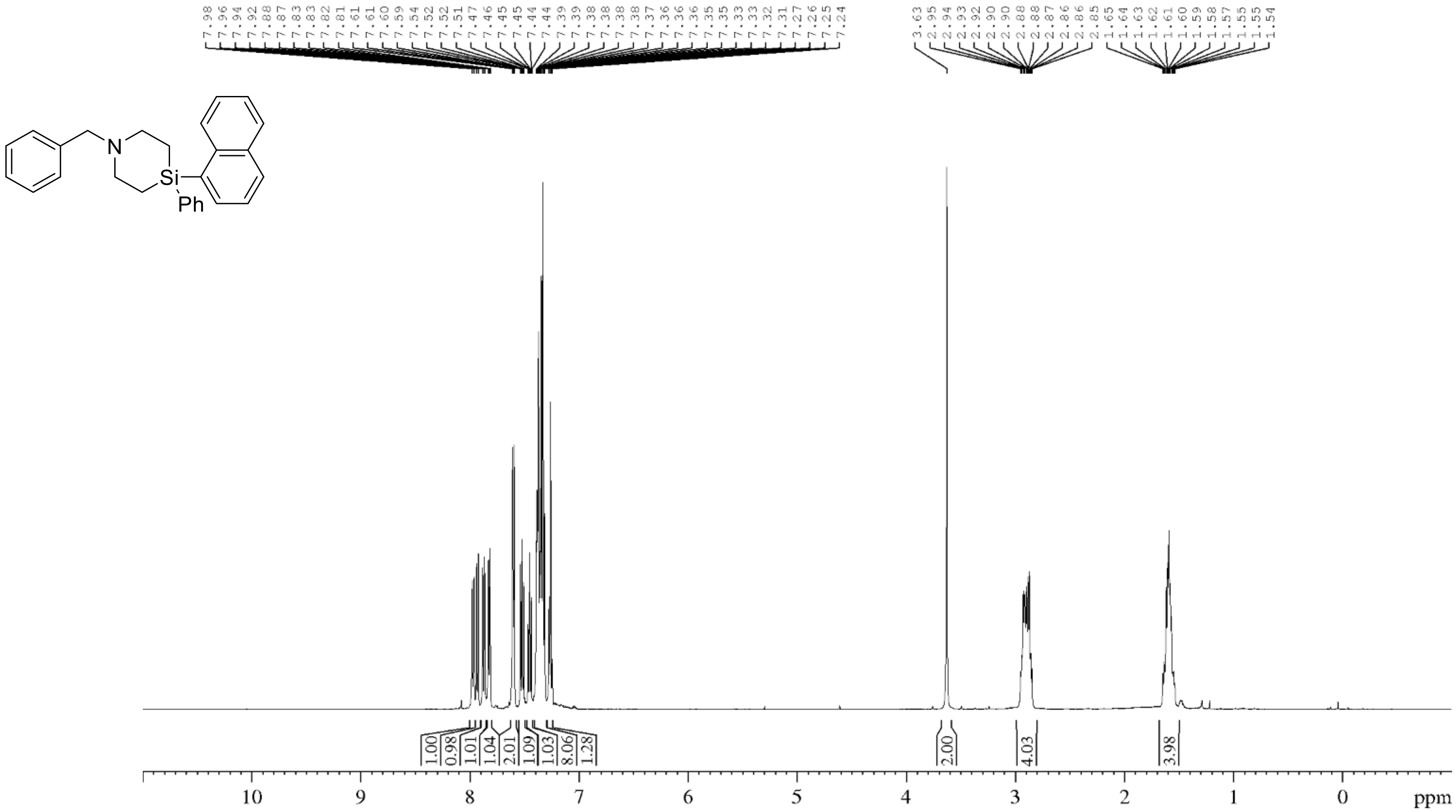

**Figure S84.** $^{13}C$ NMR spectrum (125 MHz, $CDCl_3$, 298 K) of **1-benzyl-4-(naphthalen-1-yl)-4-phenyl-1,4-azasilinane (3ae)**.

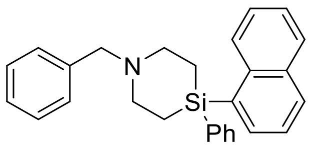


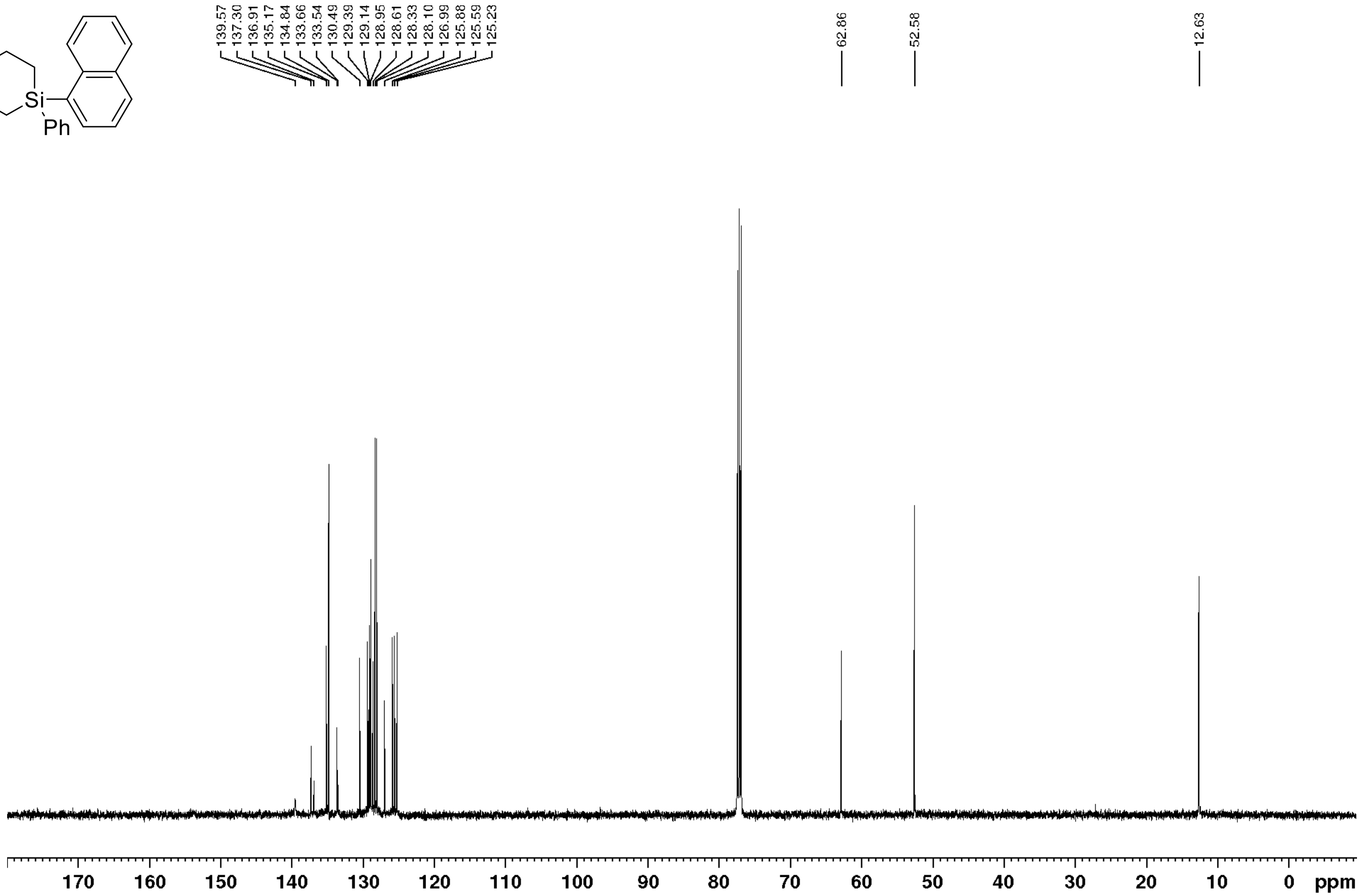

**Figure S85.** $^{1}H/^{29}Si$ HMQC NMR (500/99 MHz, $CDCl_3$, 298 K, optimized for $J$ = 7 Hz) of

**1-benzyl-4-(naphthalen-1-yl)-4-phenyl-1,4-aza-silinane (3ae)**.

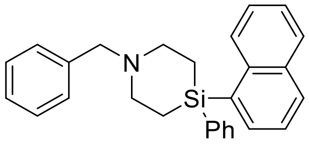


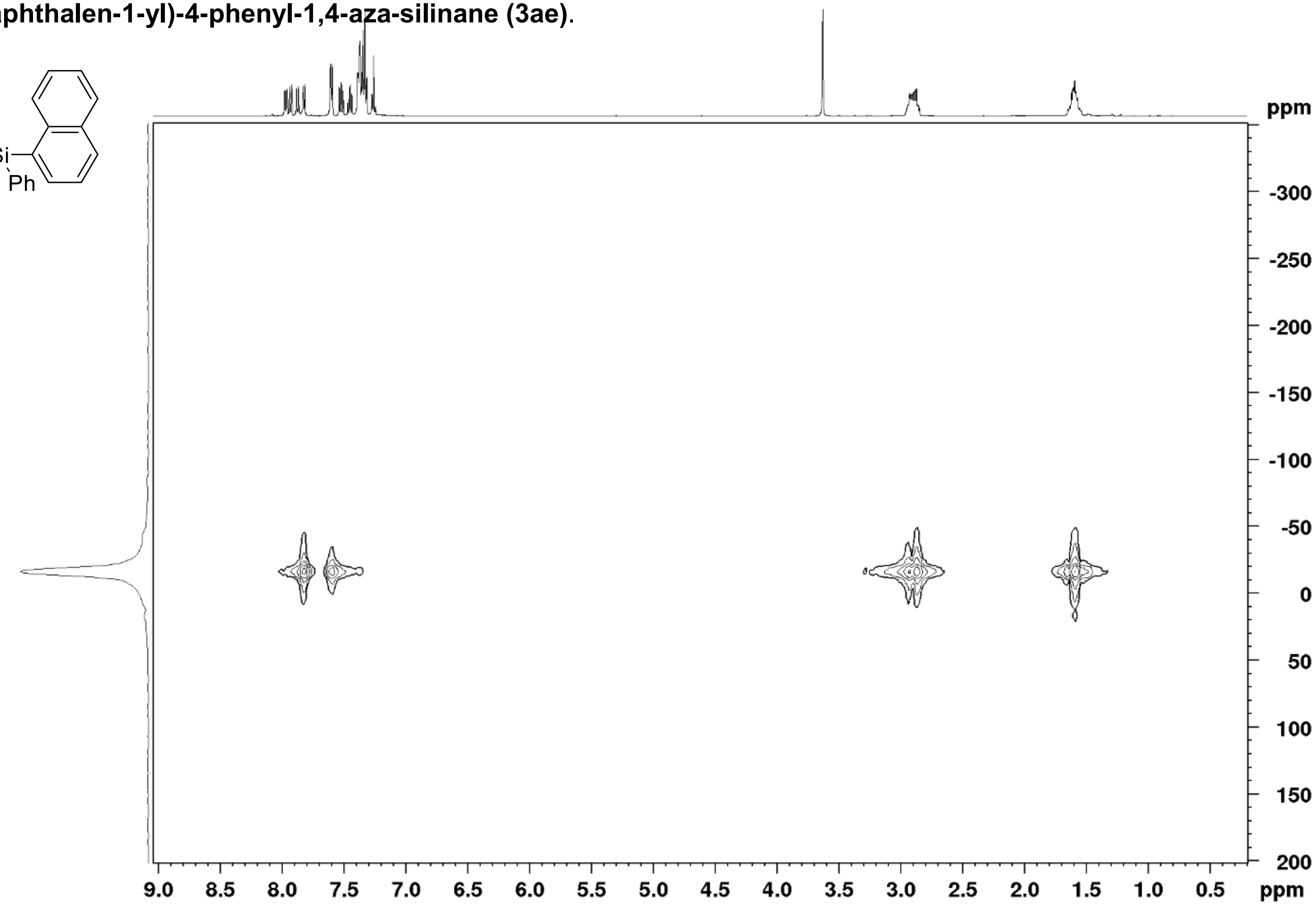

**Figure S86.** $^1$H NMR spectrum (500 MHz, $CDCl_3$, 298 K) of **1-benzyl-4-methyl-4-phenyl-1,4-azasilinane (3ag)**.

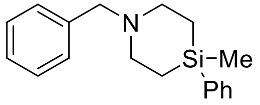

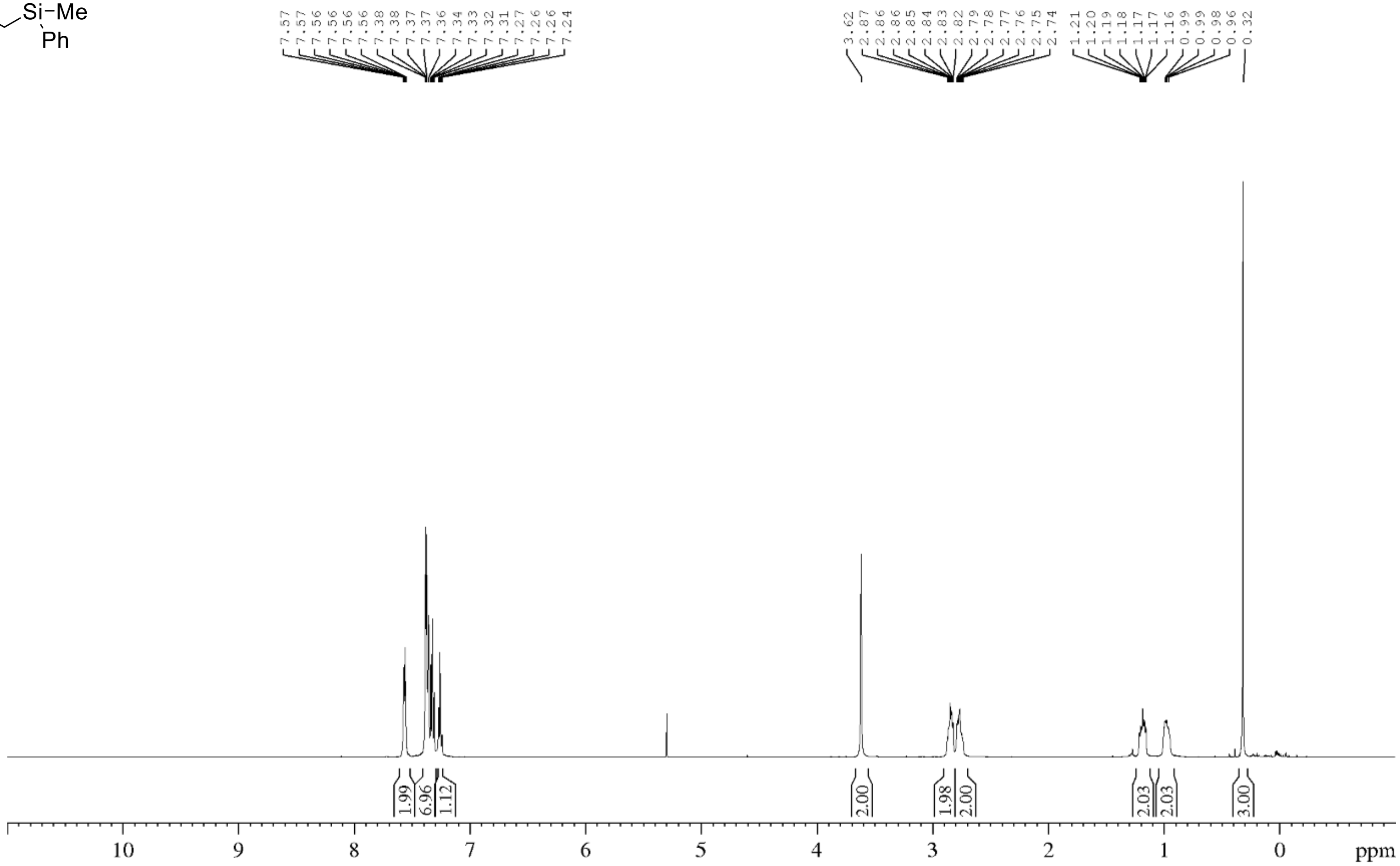

**Figure S87.** $^{13}$C NMR spectrum (125 MHz, $CDCl_3$, 298 K) of **1-benzyl-4-methyl-4-phenyl-1,4-azasilinane (3ag)**.

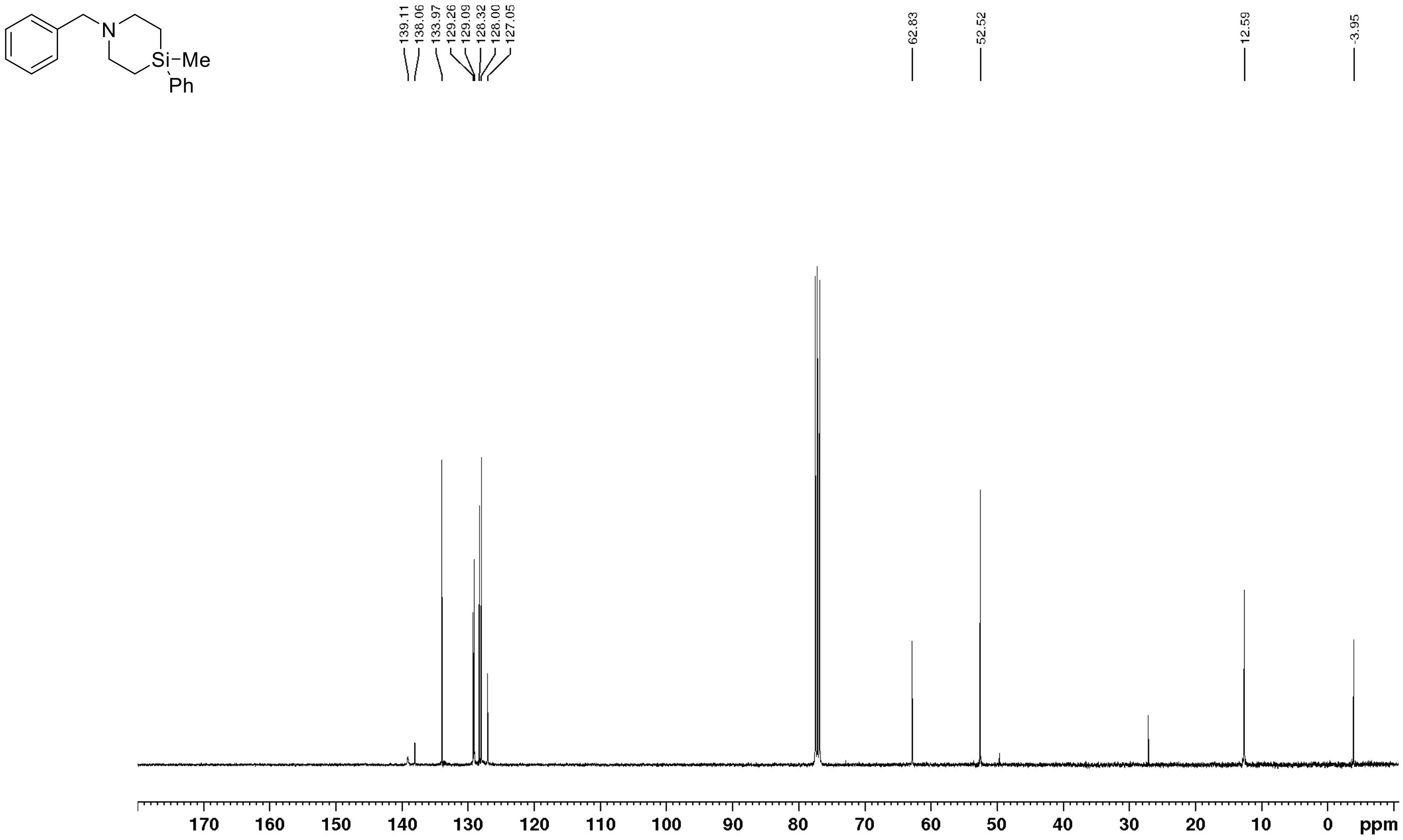

**Figure S88.** $^{1}H/^{29}Si$ HMQC NMR (500/99 MHz, $CDCl_3$, 298 K, optimized for $J$ = 7 Hz) of **1-benzyl-4-methyl-4-phenyl-1,4-azasilinane (3ag)**.

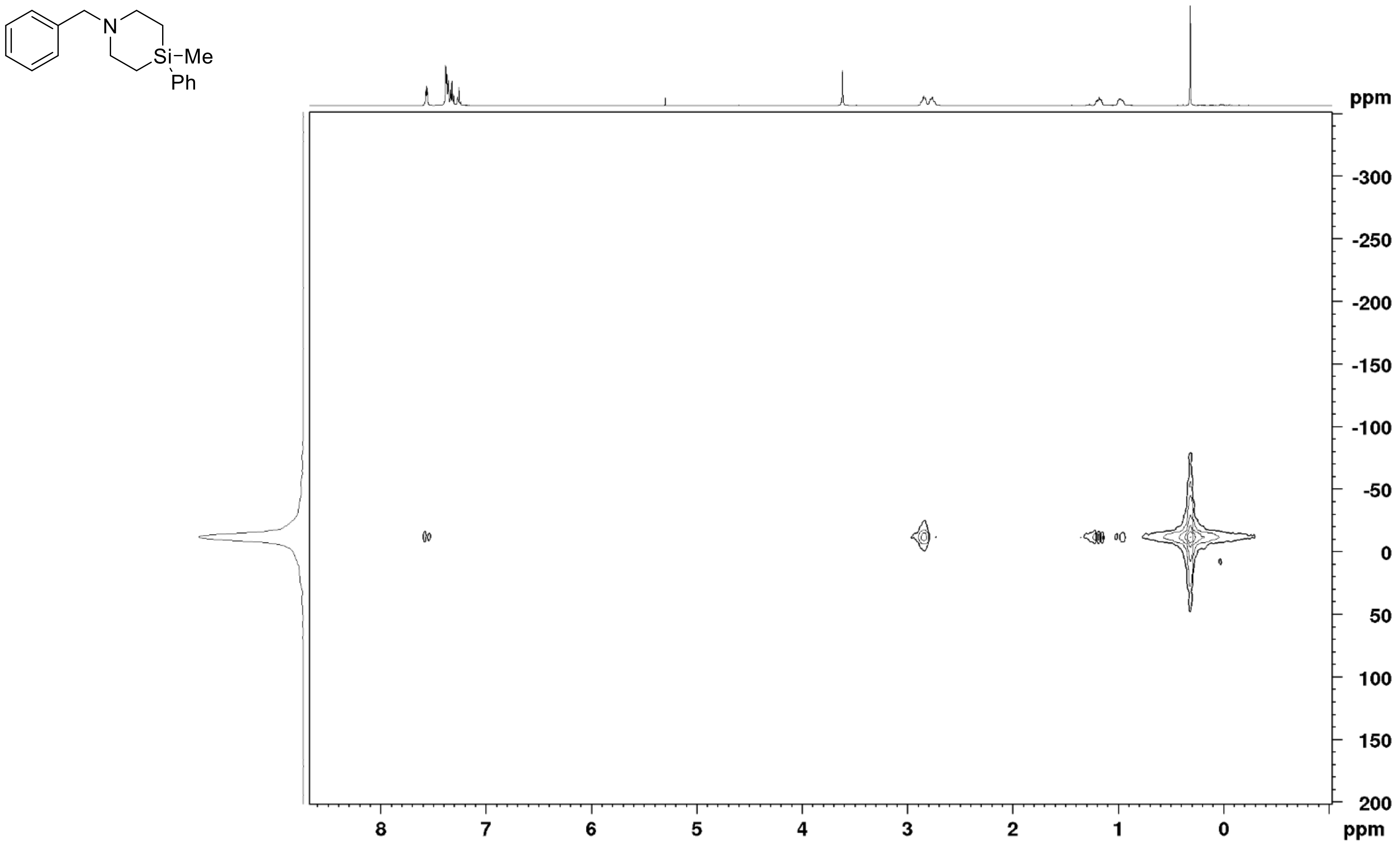

**Figure S89.** $^{1}$H NMR spectrum (500 MHz, $CDCl_3$, 298 K) of **1-benzyl-4,4-diethyl-1,4-azasilinane (3ai)**.

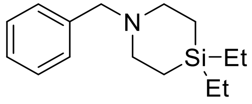


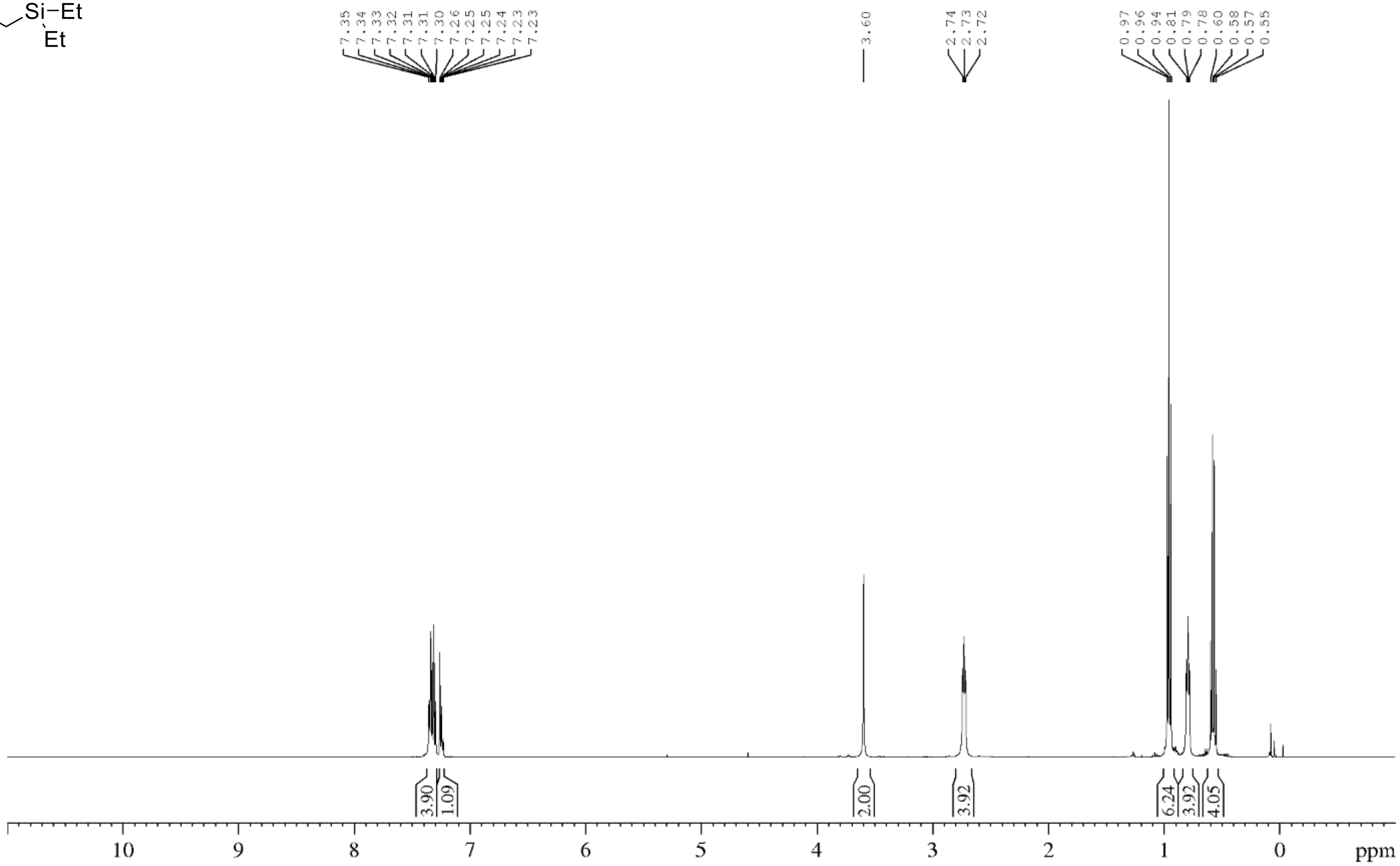

**Figure S90.** $^{13}C$ NMR spectrum (125 MHz, $CDCl_3$, 298 K) of **1-benzyl-4,4-diethyl-1,4-azasilinane (3ai)**.

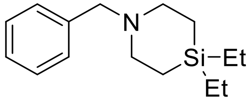


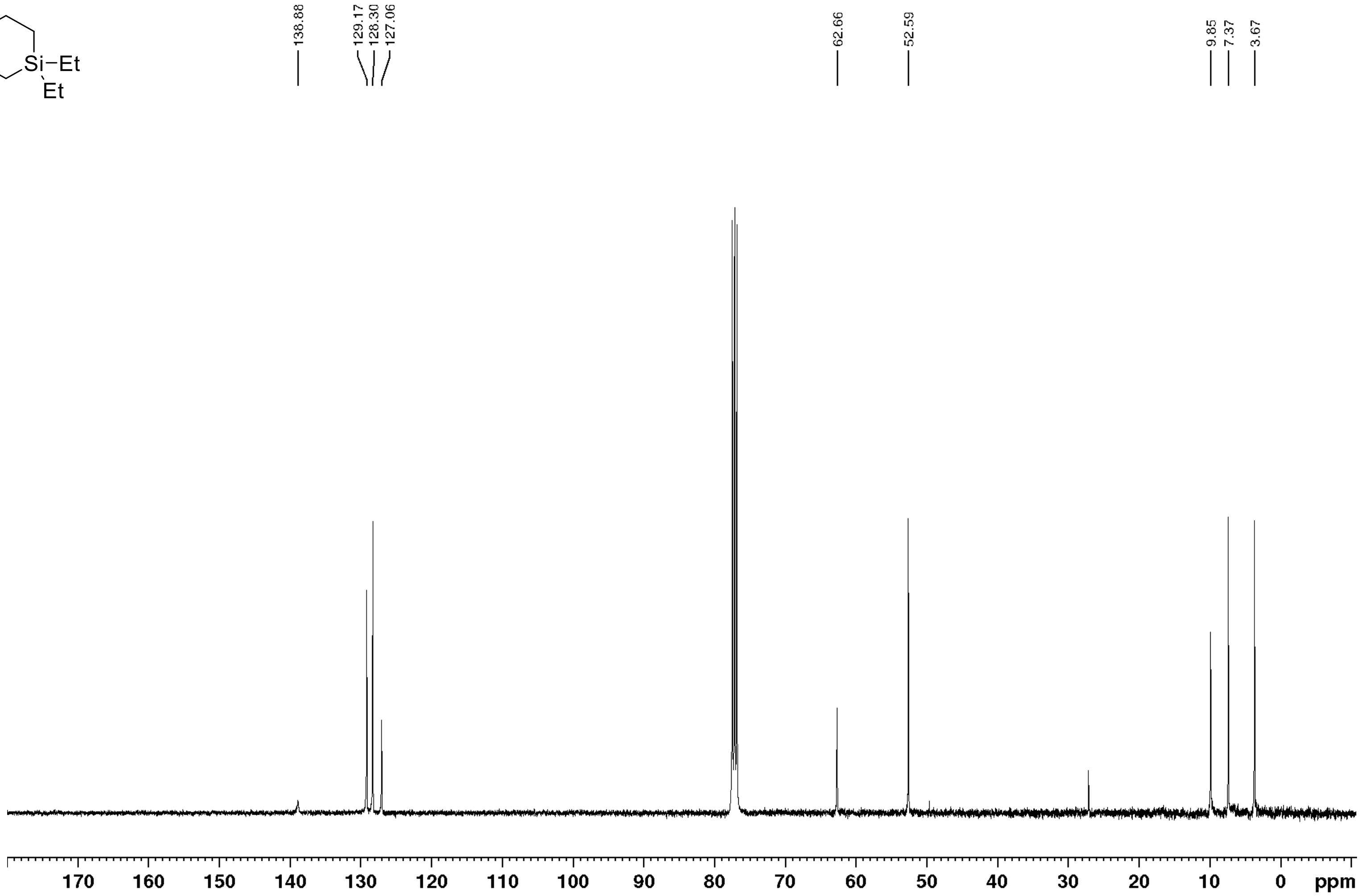

**Figure S91.** $^{1}H/^{29}Si$ HMQC NMR (500/99 MHz, $CDCl_3$, 298 K, optimized for $J$ = 7 Hz) of **1-benzyl-4,4-diethyl-1,4-azasilinane (3ai)**.

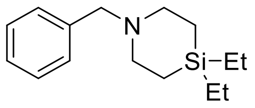


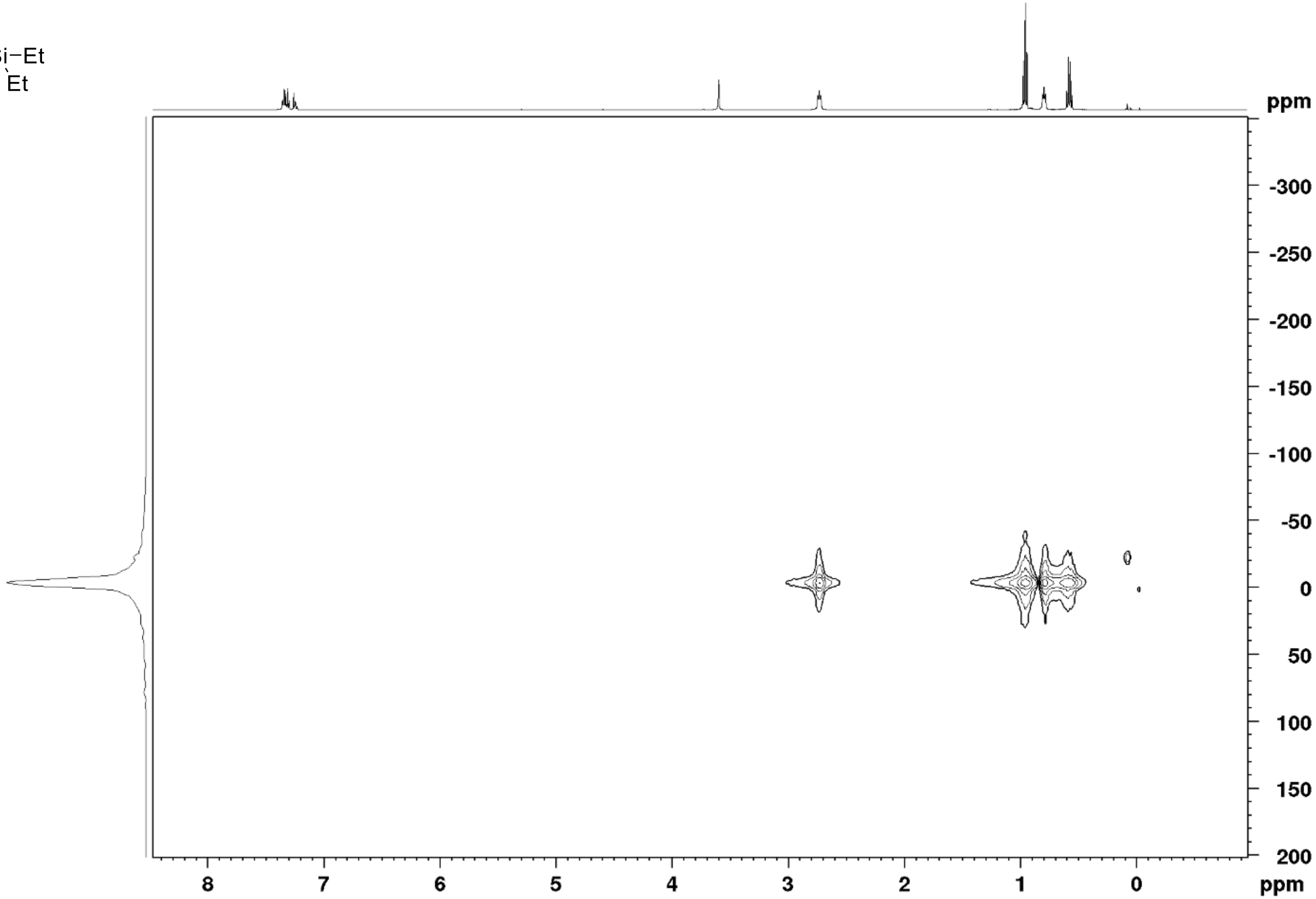

**Figure S92.** $^1$H NMR spectrum (500 MHz, $CDCl_3$, 298 K) of **4,4-diphenyl-1,4-azasilinane hydrochloride (6)**.

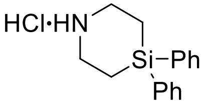


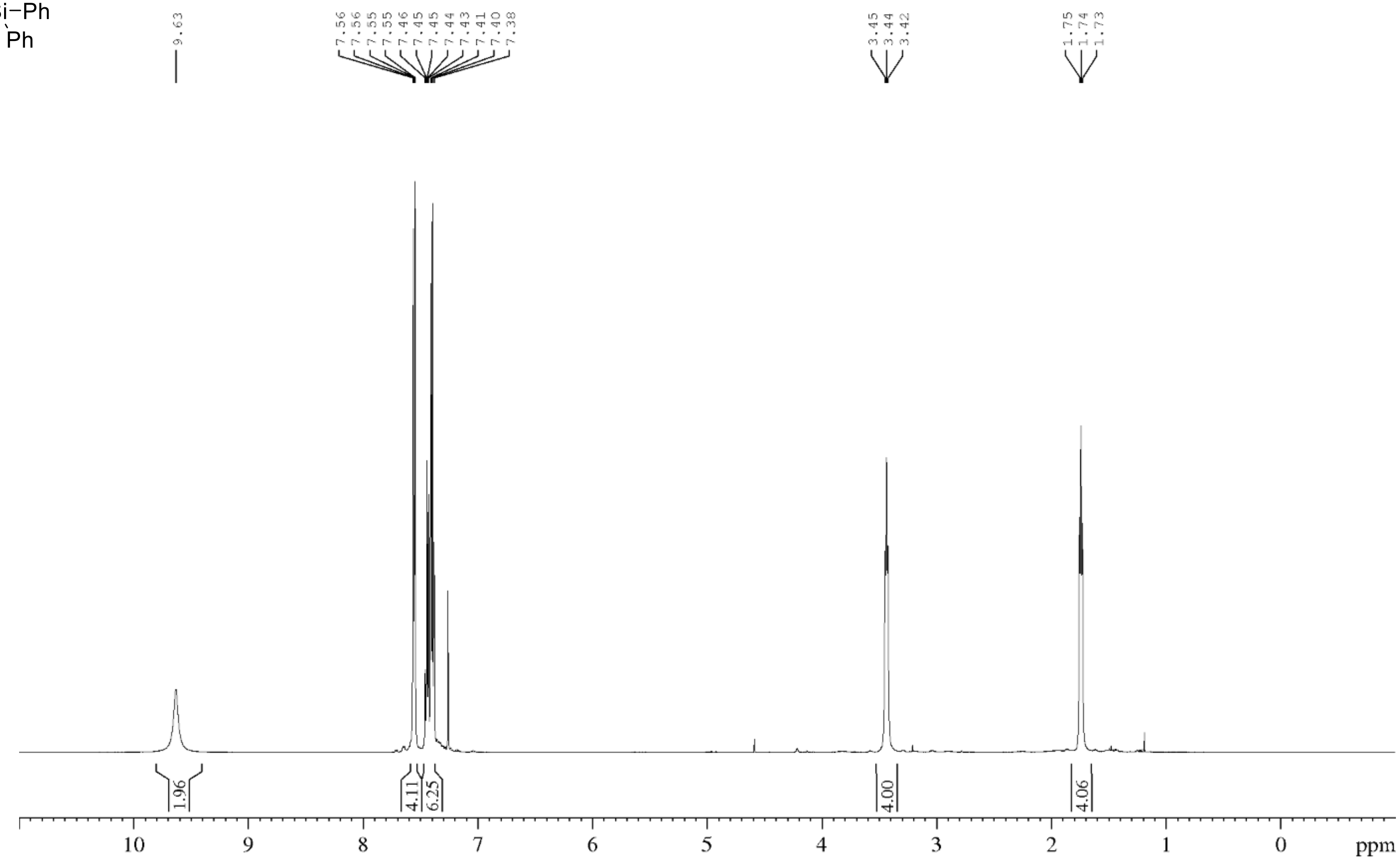

**Figure S93.** $^{13}C$ NMR spectrum (125 MHz, $CDCl_3$, 298 K) of **4,4-diphenyl-1,4-azasilinane hydrochloride (6)**.

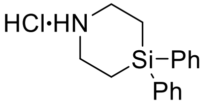


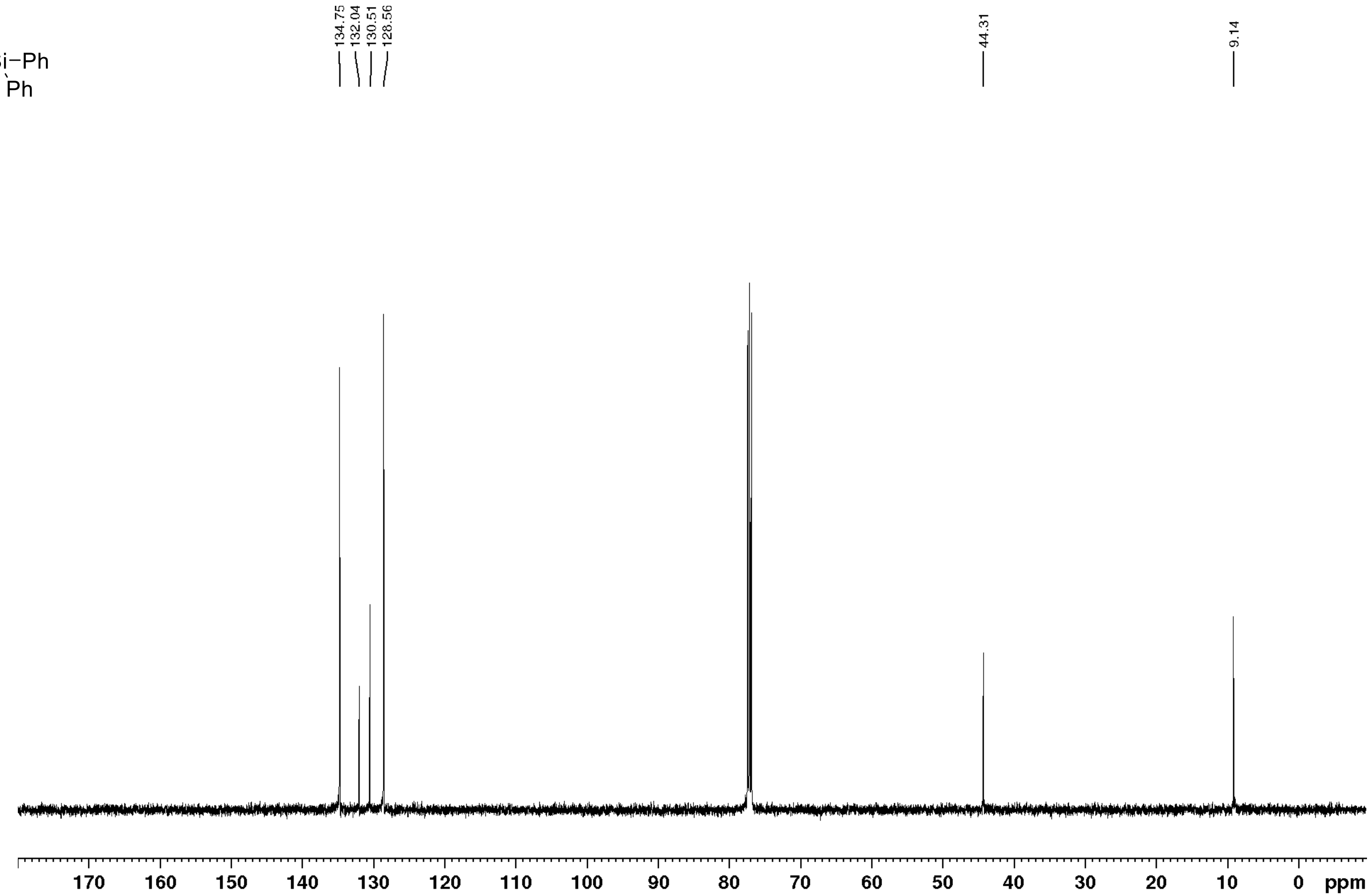

**Figure S94.** $^{1}H/^{29}Si$ HMQC NMR (500/99 MHz, $CDCl_3$, 298 K, optimized for *J* = 7 Hz) of **4,4-diphenyl-1,4-azasilinane hydrochloride (6)**.

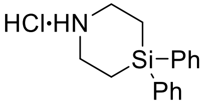


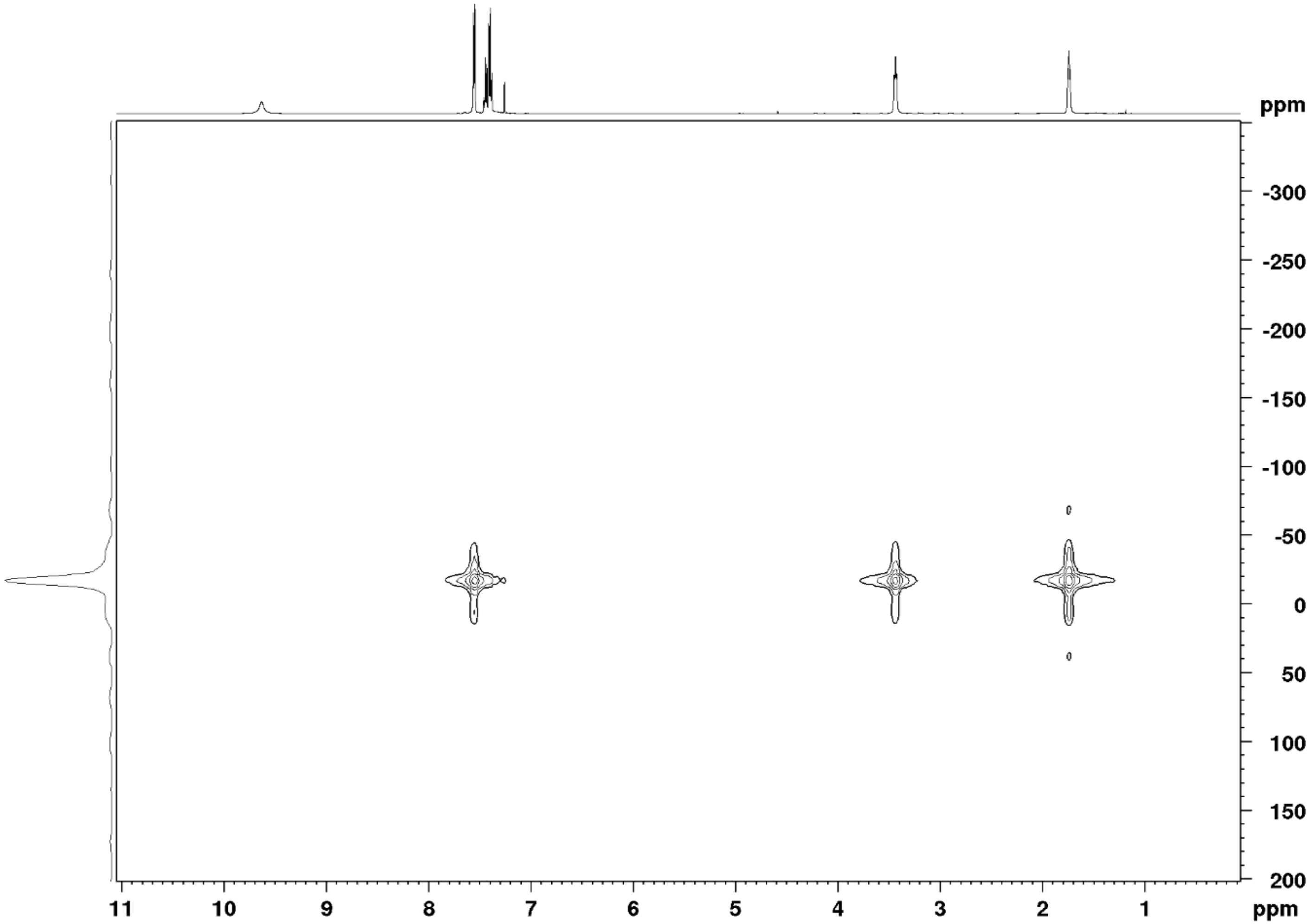

**Figure S95.** $^{1}$H NMR spectrum (500 MHz, $CDCl_3$, 298 K) of **(4,4-diphenyl-1,4-azasilinan-1-yl)(phenyl)methanone (7)**.

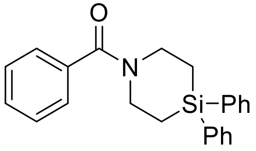


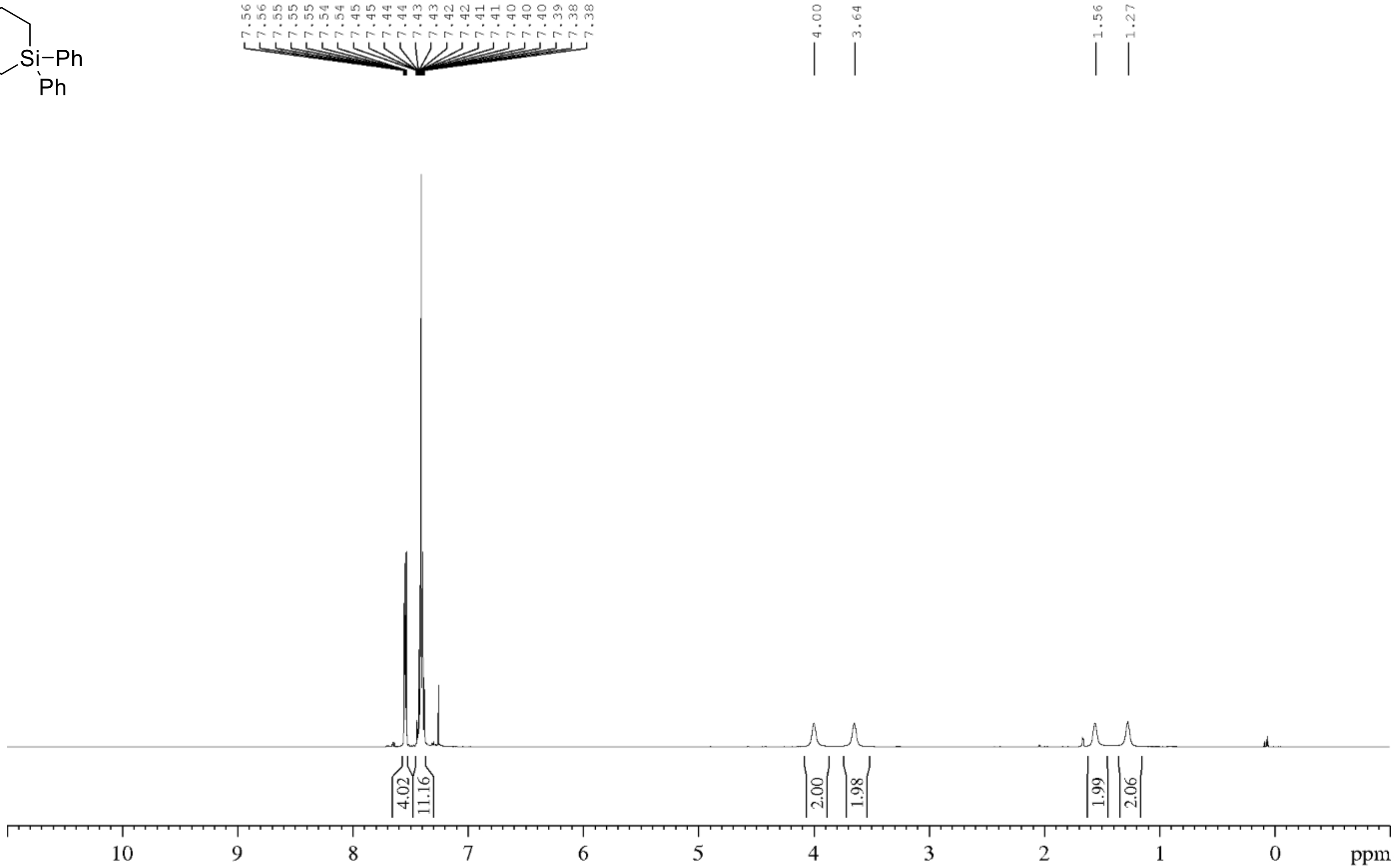

**Figure S96.** $^{13}$C NMR spectrum (125 MHz, $CDCl_3$, 298 K) of **(4,4-diphenyl-1,4-azasilinan-1-yl)(phenyl)methanone (7)**.

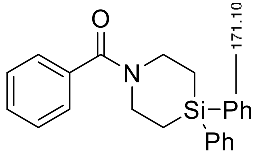

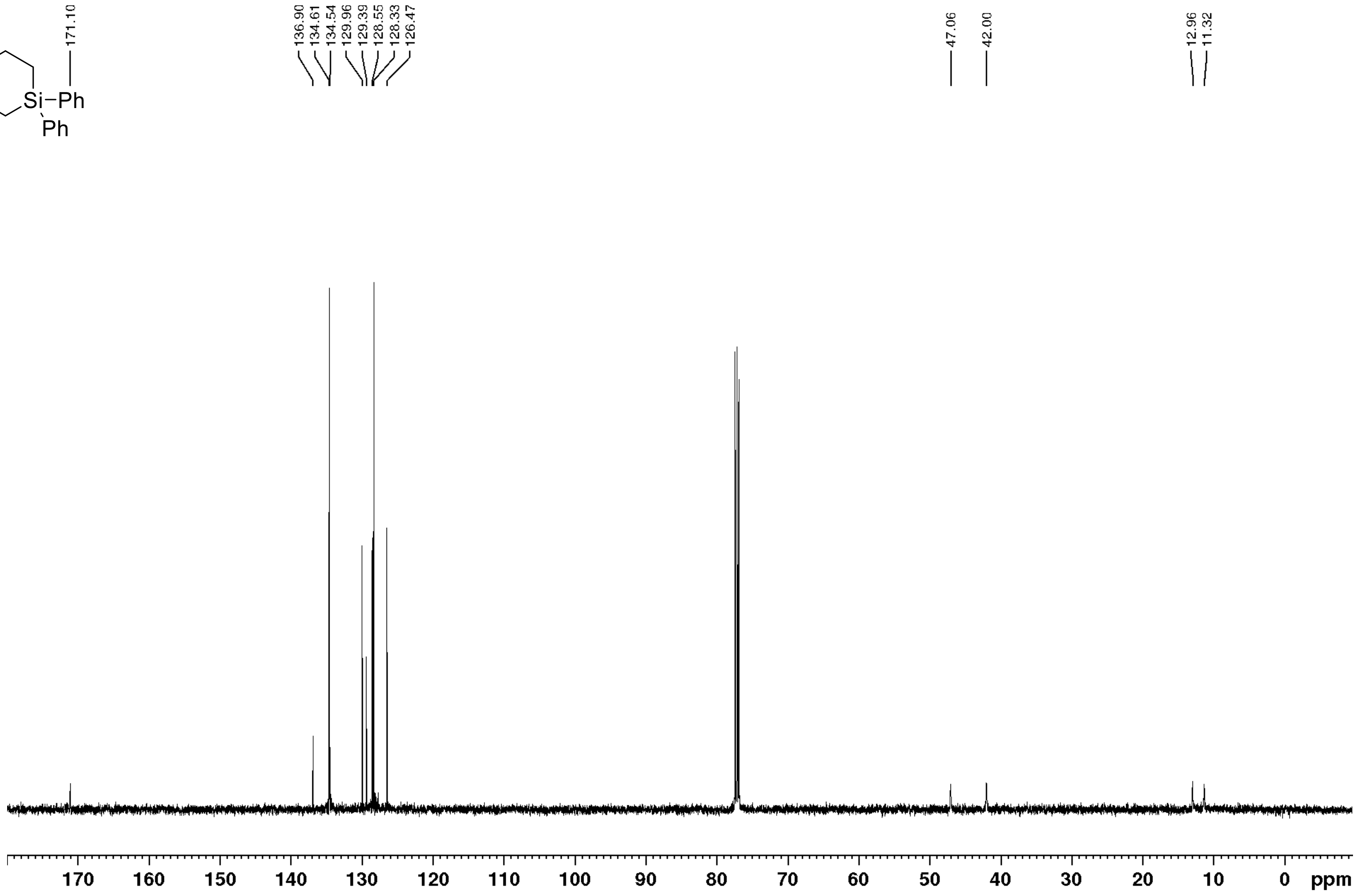

**Figure S97** $^{1}H/^{29}Si$ HMQC NMR (500/99 MHz, $CDCl_3$, 298 K, optimized for *J* = 7 Hz) of **(4,4-diphenyl-1,4-azasilinan-1-yl)(phenyl)methanone (7)**.

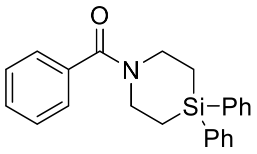


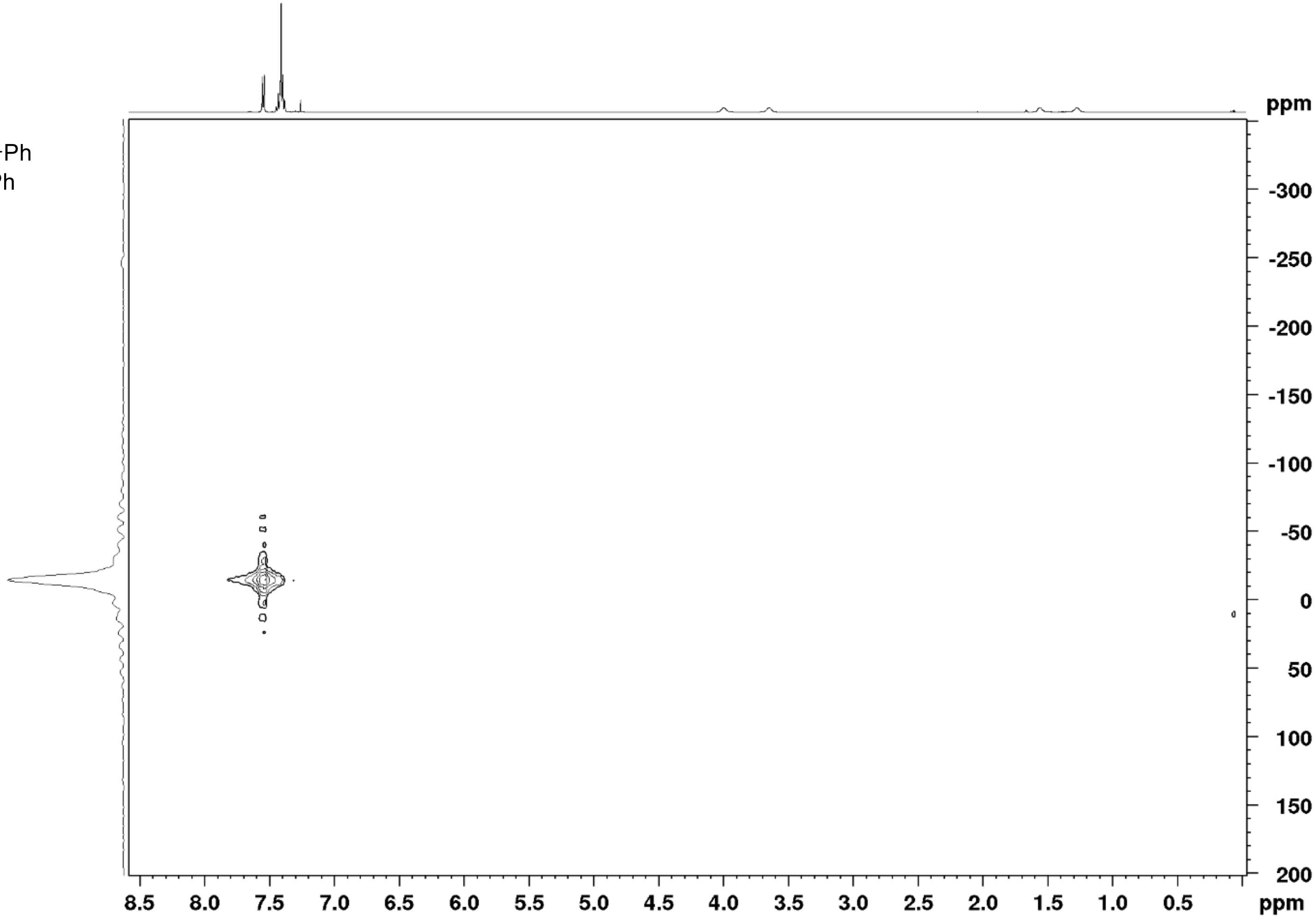

**Figure S98.** $^{1}$H NMR spectrum (500 MHz, $CDCl_3$, 298 K) of **4,4-diphenyl-1-(phenylmethyl-d)-1,4-azasilinane-2,6-*d*$_2$ (3aa-*d*$_3$)**.

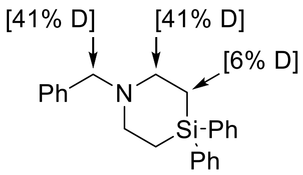


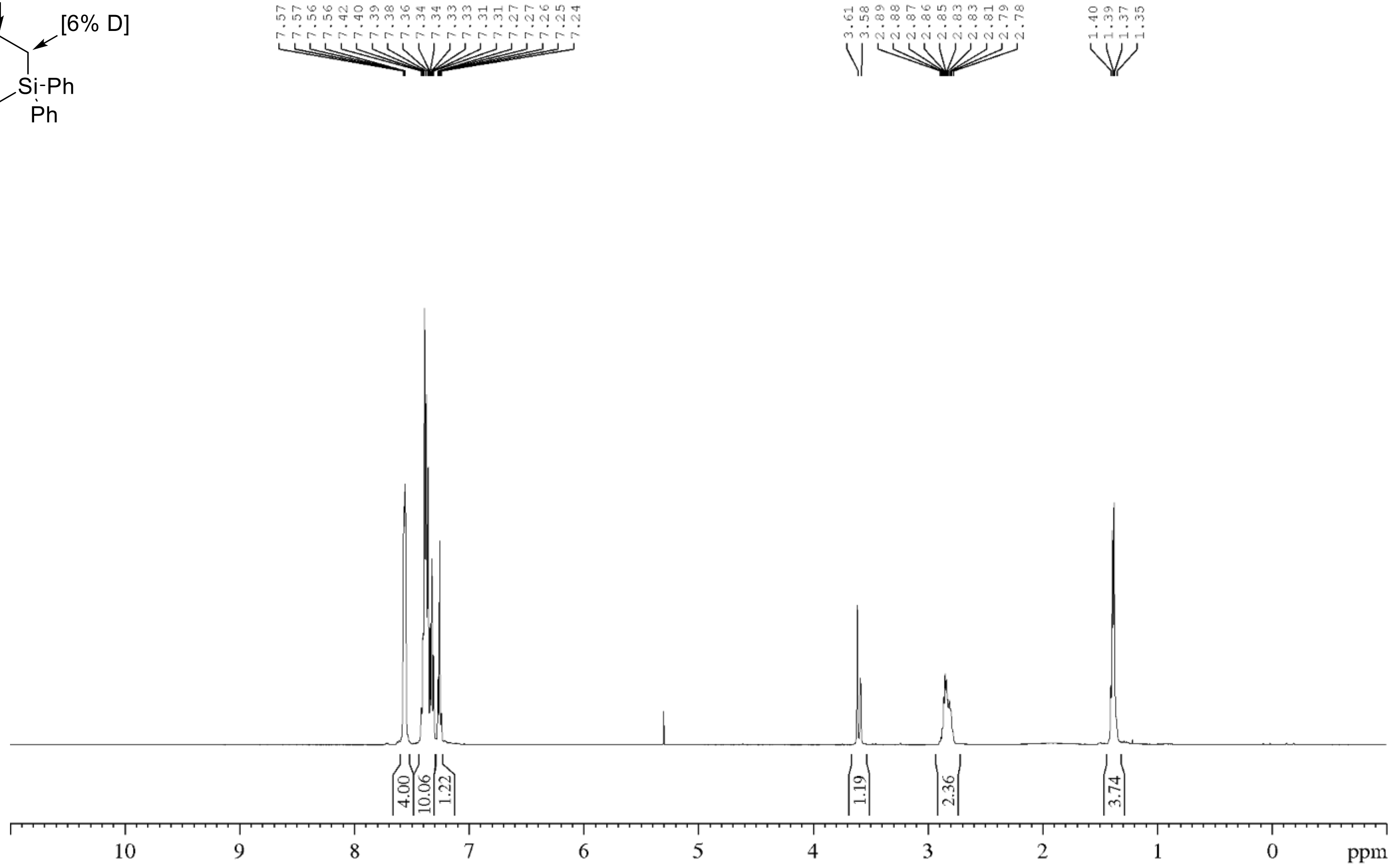

**Figure S99.** $^{13}$C NMR spectrum (125 MHz, $CDCl_3$, 298 K) of **4,4-diphenyl-1-(phenylmethyl-d)-1,4-azasilinane-2,6-$d_2$ (3aa-$d_3$)**.

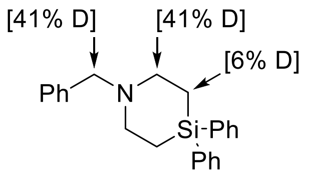


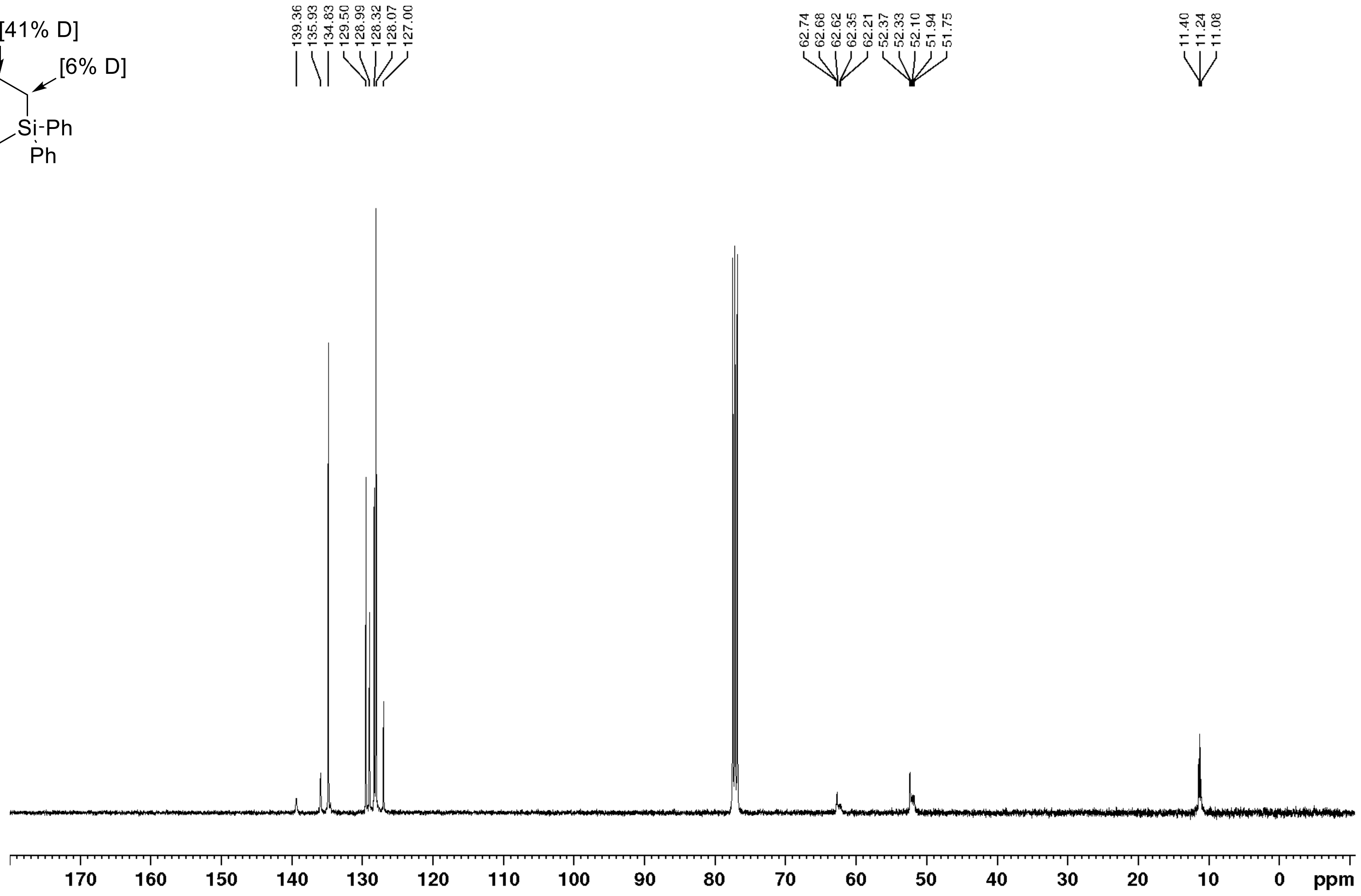

**Figure S100.** $^{1}H/^{29}Si$ HMQC NMR (500/99 MHz, $CDCl_3$, 298 K, optimized for $J$ = 7 Hz) of

**4,4-diphenyl-1-(phenylmethyl-d)-1,4-azasilinane-2,6-$d_2$ (3aa-$d_3$).**

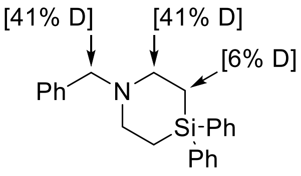


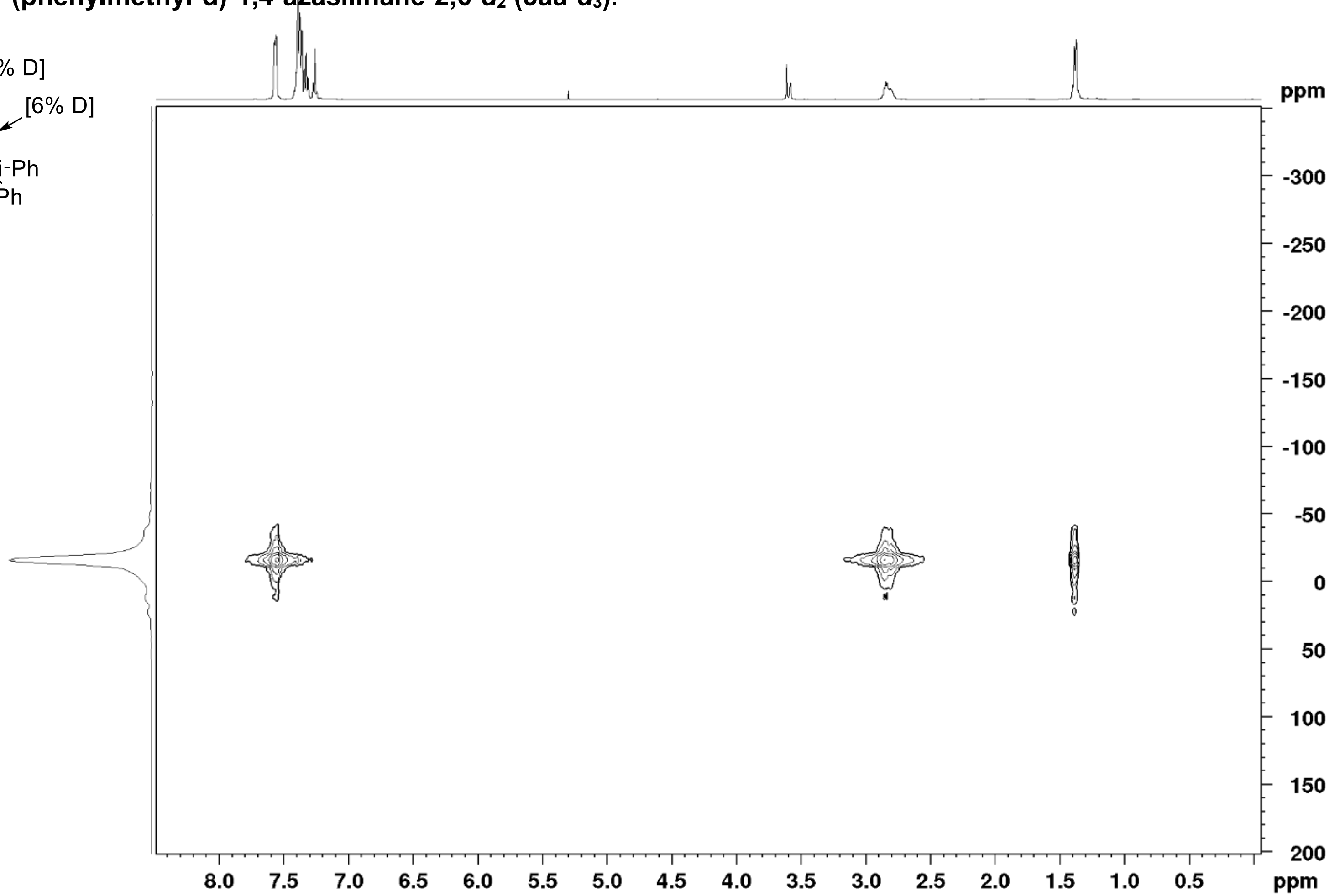

**Figure S101.** $^{1}H$ NMR spectrum (500 MHz, $CDCl_3$, 298 K) of ***N*-benzyl-*N*-butylbutan-1-amine-*d*$_3$ (1v-*d*$_3$)**.

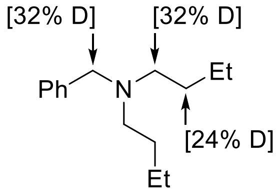


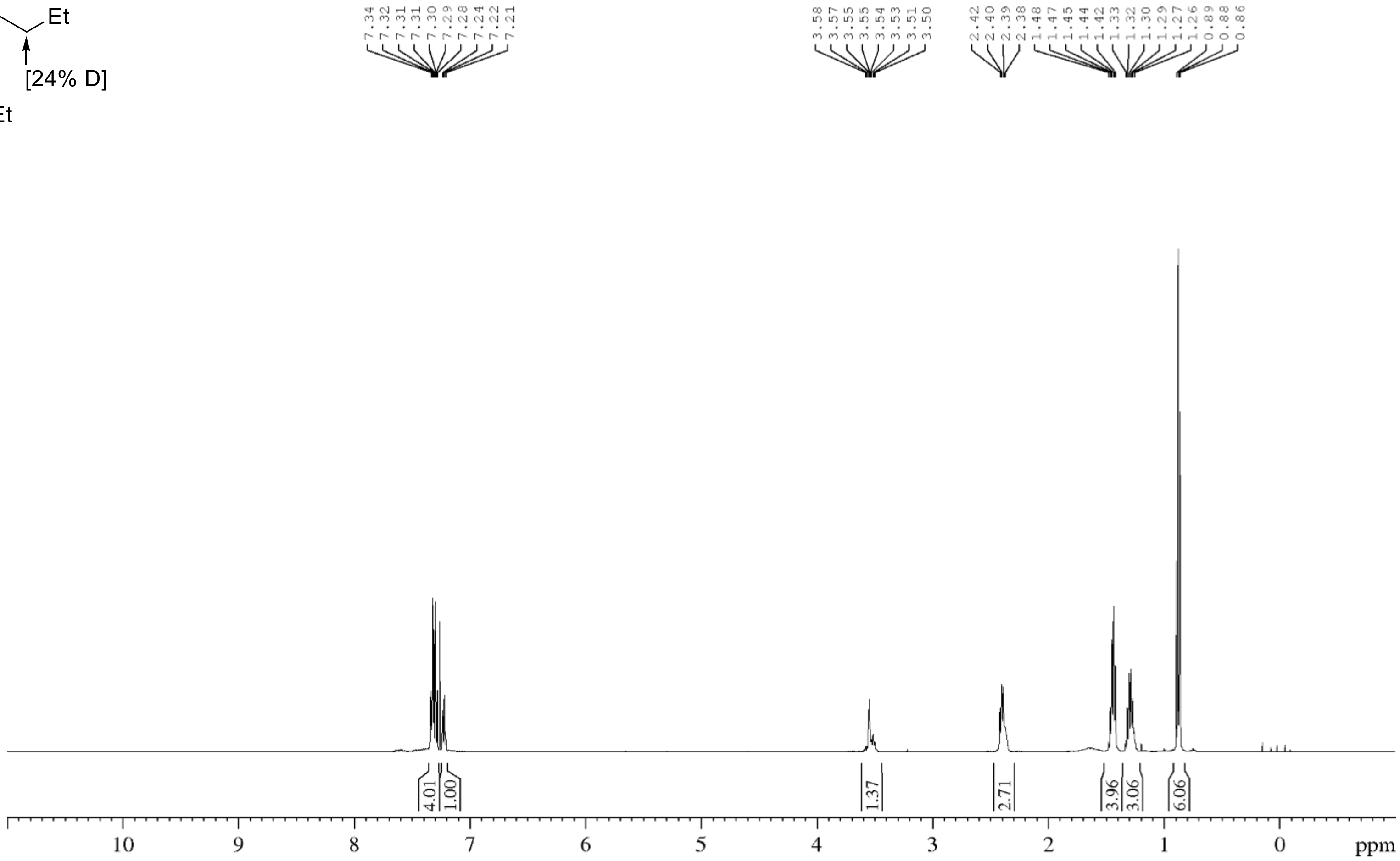

**Figure S102.** $^{13}$C NMR spectrum (125 MHz, $CDCl_3$, 298 K) of ***N*-benzyl-*N*-butylbutan-1-amine-*d*$_3$ (1v-*d*$_3$)**..

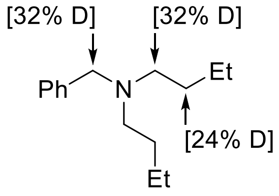


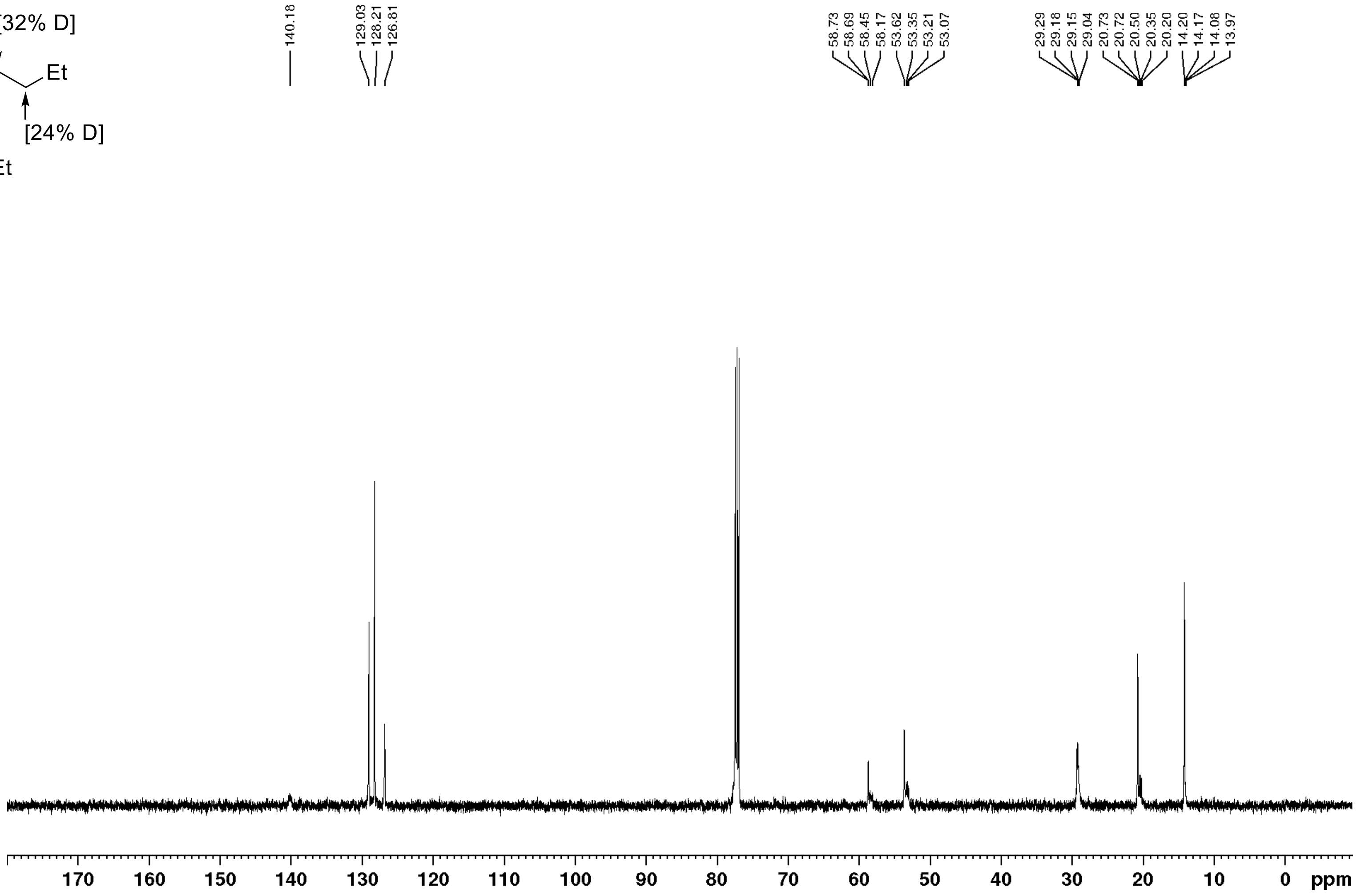

**Figure S103.** $^{1}H$ NMR spectrum (500 MHz, $CDCl_3$, 298 K) of the stoichiometric reaction of **1a** and $B(C_6F_5)_3$ (1:1).

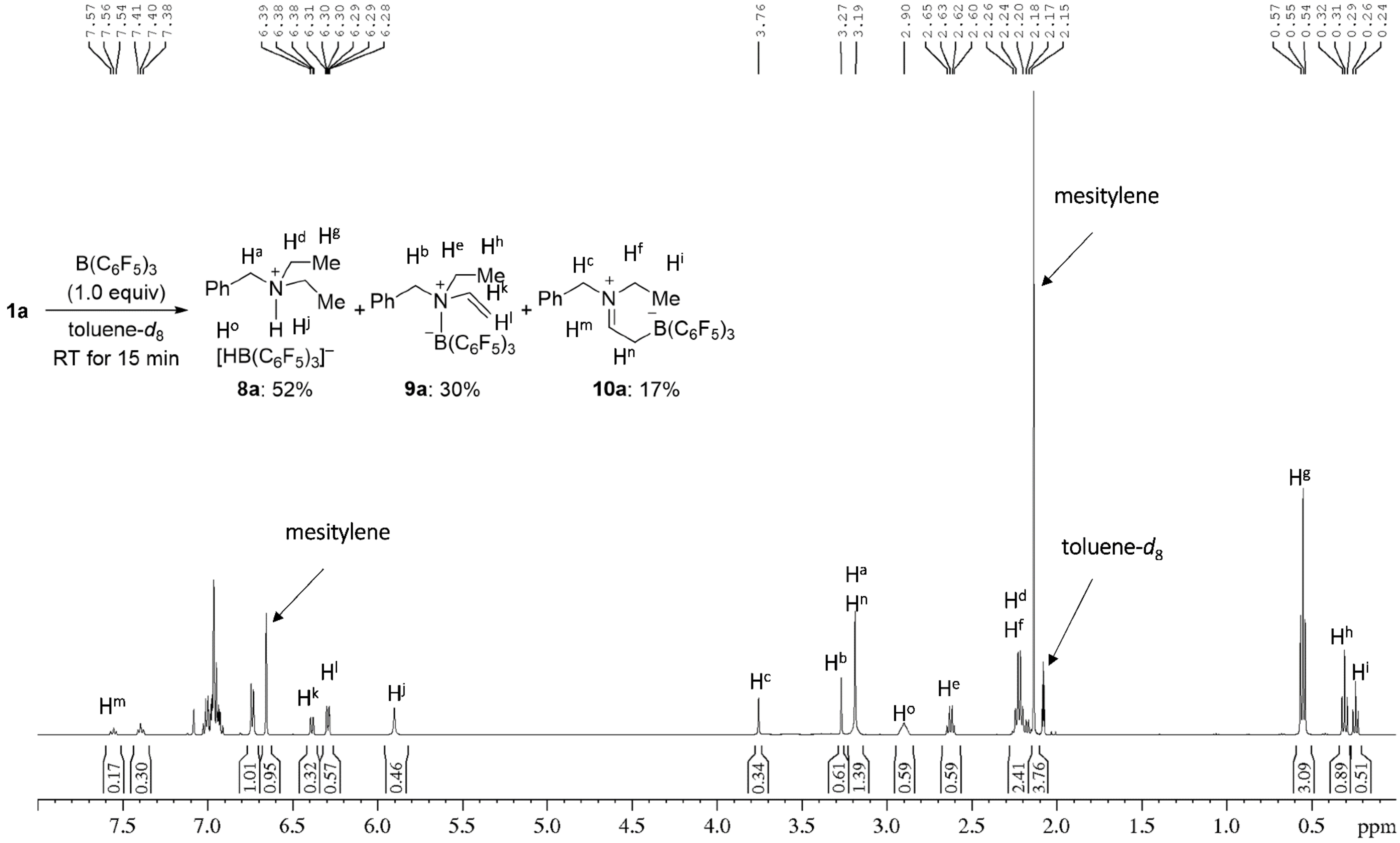

**Figure S104.** $^{11}B$ NMR spectrum (161 MHz, 1,2-$C_6D_4Cl_2$, 298 K) of the stoichiometric reaction of **1a** and $B(C_6F_5)_3$ (1:1).

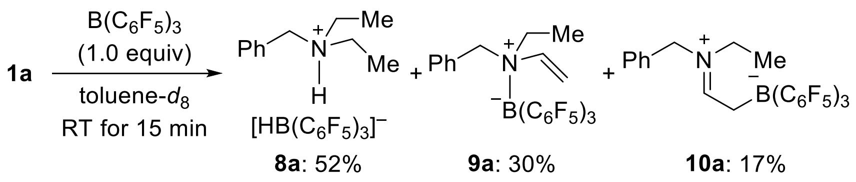


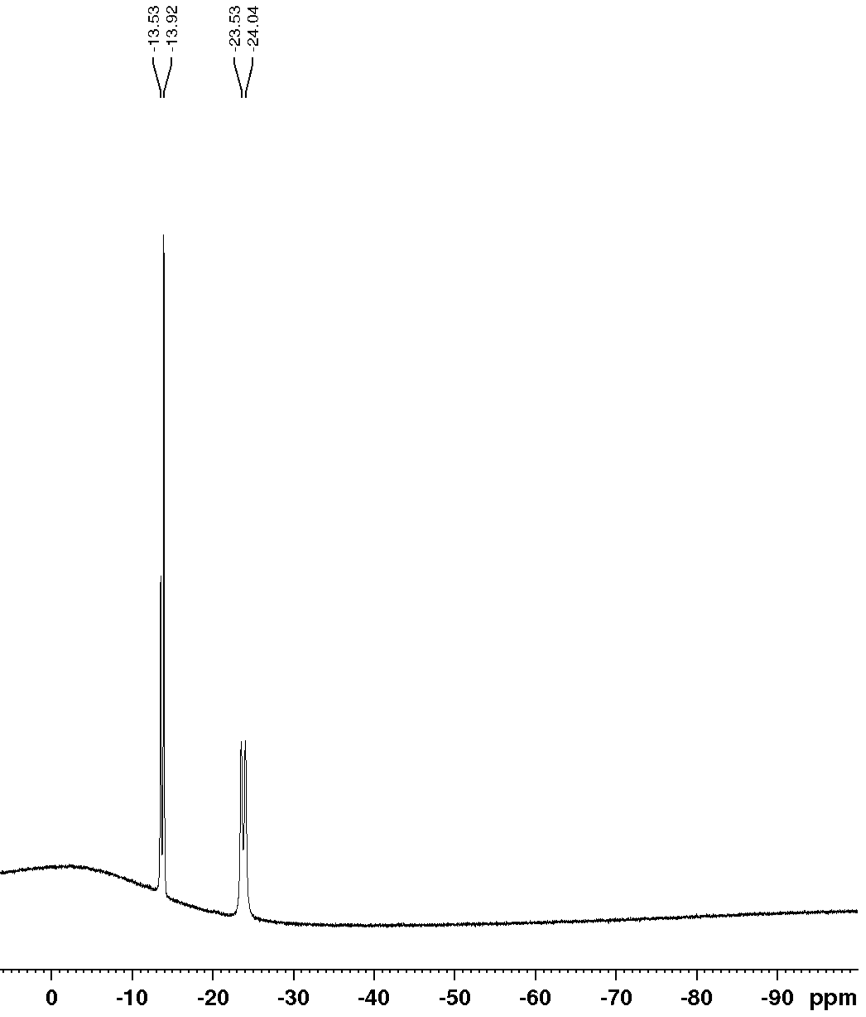

**Figure S105.** $^{19}F$ NMR spectrum (471 MHz, 1,2-$C_6D_4Cl_2$, 298 K) of the stoichiometric reaction of **1a** and $B(C_6F_5)_3$ (1:1).

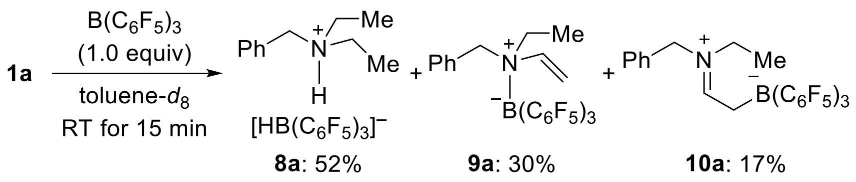


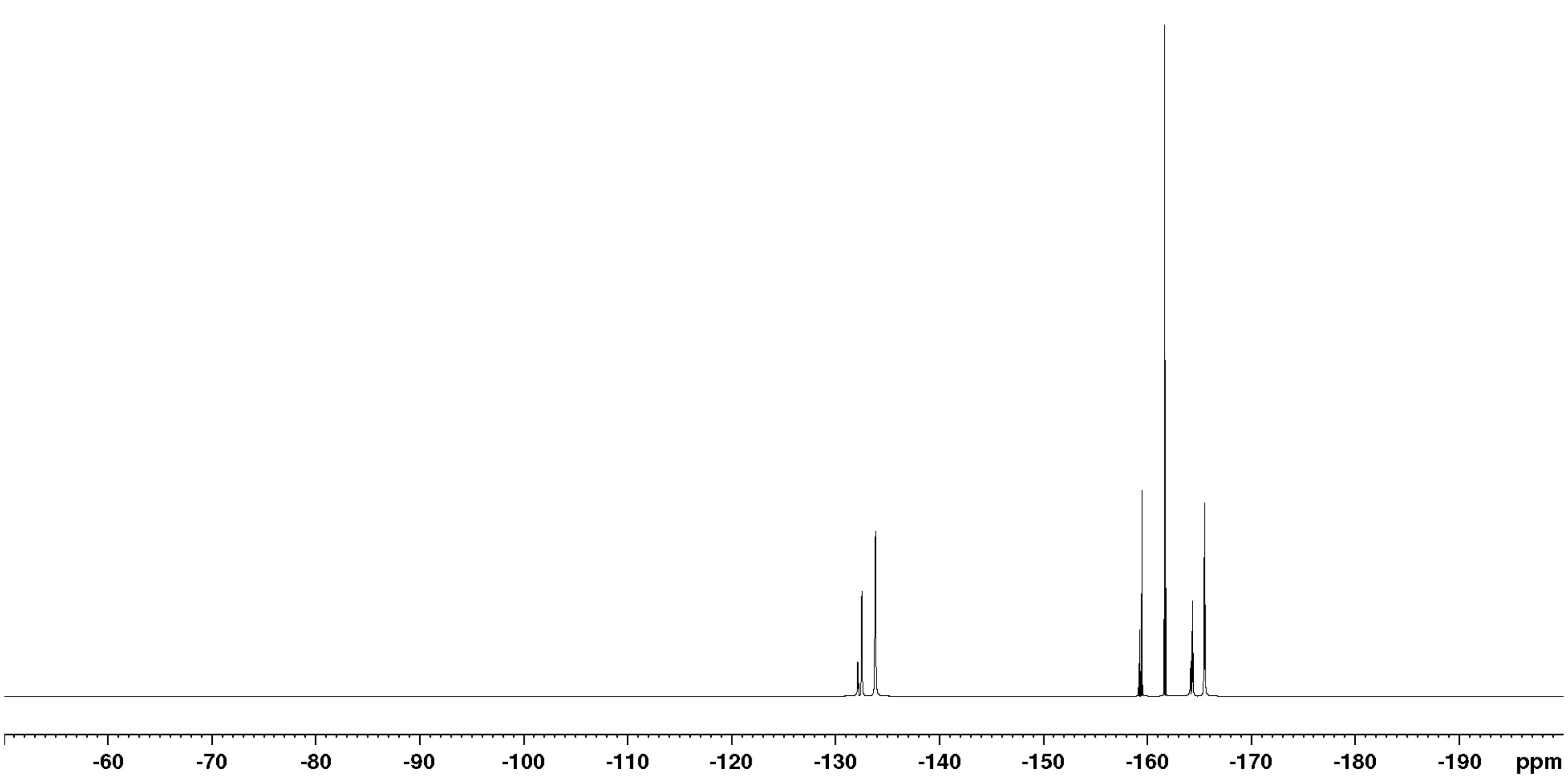

**Figure S106.** $^1$H NMR spectrum (500 MHz, $CDCl_3$, 298 K) of the stoichiometric reaction of **1a**, $Me_3SiOTf$, and $B(C_6F_5)_3$ (1:1:1).

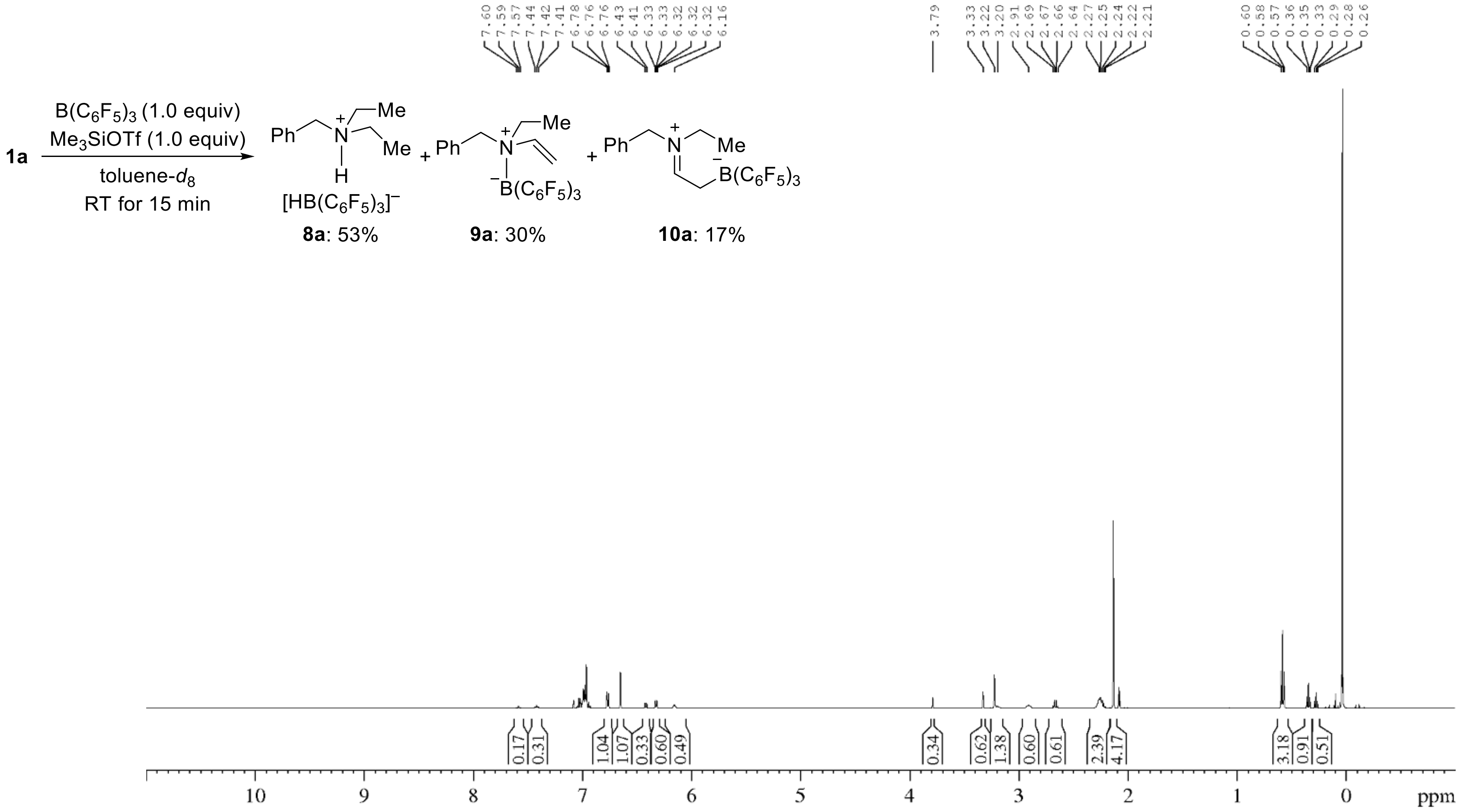

**Figure S107.** $^{11}B$ NMR spectrum (161 MHz, 1,2-$C_6D_4Cl_2$, 298 K) of the stoichiometric reaction of **1a**, $Me_3SiOTf$, and $B(C_6F_5)_3$ (1:1:1).

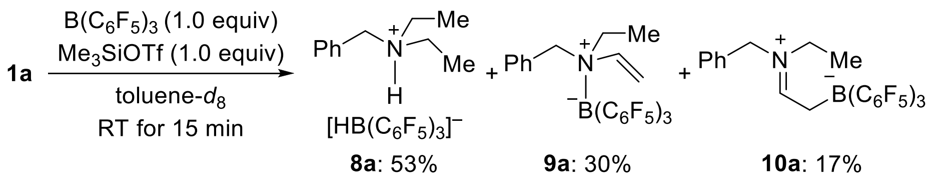


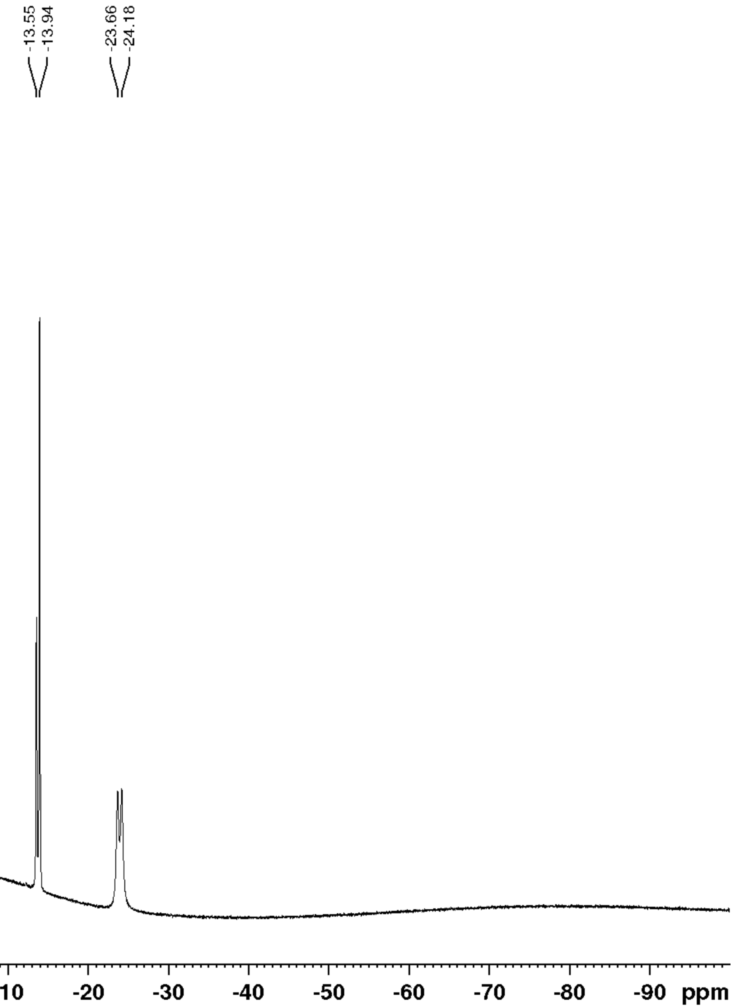

**Figure S108.** $^{19}F$ NMR spectrum (471 MHz, 1,2-$C_6D_4Cl_2$, 298 K) of the stoichiometric reaction of **1a**, $Me_3SiOTf$, and $B(C_6F_5)_3$ (1:1:1).

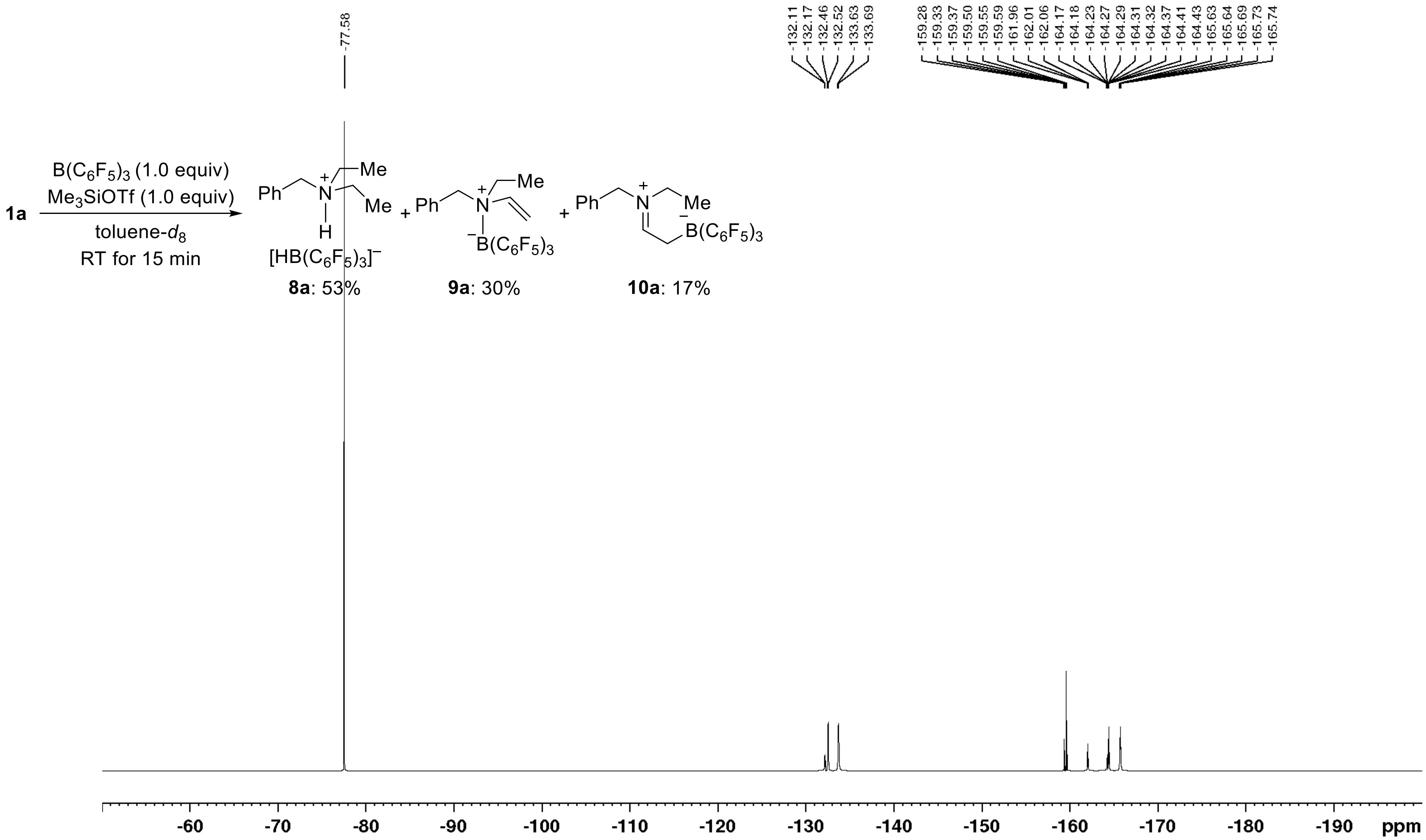

## 8 References


[1] P. D. Prince, M. J. Bearpark, G. S. McGrady, J. W. Steed, *Dalton Trans.* **2008**, 271–282.

[2] X. Yang, C. Wang, *Angew. Chem. Int. Ed.* **2018**, *57*, 923–928; *Angew. Chem.* **2018**, *130*, 935–940.

[3] a) S. J. Aspin, S. Taillemaud, P. Cyr, A. B. Charette, *Angew. Chem. Int. Ed.* **2016**, *55*, 13833–13837; *Angew. Chem.* **2016**, *128*, 14037–14041; b) G. Zhan, H.-L. Teng, Y. Luo, S.-J. Lou, M. Nishiura, Z. Hou, *Angew. Chem. Int. Ed.* **2018**, *57*, 12342–12346; *Angew. Chem.* **2018**, *130*, 12522–12526.

[4] N. T. Tran, T. Min, A. K. Franz, *Chem. Eur. J.* **2011**, *17*, 9897–9900.

[5] N. Gandhamsetty, S. Park, S. Chang, *J. Am. Chem. Soc.* **2015**, *137*, 15176–15184.

[6] S. E. Varjosaari, V. Skrypai, P. Suating, J. J. M. Hurley, A. M. D. Lio, T. M. Gilbert, M. J. Adler, *Adv. Synth. Catal.* **2017**, *359*, 1872–1878.

[7] S. R. Tamang, A. Singh, D. Bedi, A. R. Bazkiaei, A. A. Warner, K. Glogau, C. McDonald, D. K. Unruh, M. Findlater, *Nat. Catal.* **2020**, *3*, 154–162.

[8] W. Yao, H. Fang, Q. He, D. Peng, G. Liu, Z. Huang, *J. Org. Chem.* **2019**, *84*, 6084–6093.

[9] C. Li, K.-f. Wan, F.-y. Guo, Q.-h. Wu, M.-l. Yuan, R.-x. Li, H.-y. Fu, X.-l. Zheng, H. Chen, *J. Org. Chem.* **2019**, *84*, 2158–2168.

[10] H. Seo, M. H. Katcher, T. F. Jamison, *Nat. Chem.* **2017**, *9*, 453–456.

[11] a) R. Tacke, R. Bertermann, C. Burschka, S. Dörrich, M. Fischer, B. Müller, G. Meyerhans, D. Schepmann, B. Wünsch, I. Arnason, R. Bjornsson, *ChemMedChem* **2012**, *7*, 523–532; b) B. Seetharamsingh, R. Ramesh, S. S. Dange, P. V. Khairnar, S. Singhal, D. Upadhyay, S. Veeraraghavan, S. Viswanadha, S. Vakkalanka, D. S. Reddy, *Acs Med. Chem. Lett.* **2015**, *6*, 1105–1110.

[12] M. Fischer, R. Tacke, *Organometallics* **2013**, *32*, 7181–7185.

[13] D. S.Reddy, R. Ramesh, S. S. Balamkundu, PCT Int. Appl., WO2014097322A1.
